# Multiplatform comparisons and annotation of structural variants highlight the utility of the T2T reference genome in human diagnostics

**DOI:** 10.1093/gigascience/giag027

**Published:** 2026-03-09

**Authors:** Jakub Savara, Tomas Novosad, Petr Gajdos, Anna Petrackova, Marek Behalek, Jirina Manakova, Filip Ctvrtlik, Jiri Minarik, Tomas Papajik, Eva Kriegova

**Affiliations:** Department of Immunology, Faculty of Medicine and Dentistry, Palacký University Olomouc and University Hospital Olomouc, Hnevotinska 3, 77900 Olomouc, Czech Republic; Department of Computer Science, Faculty of Electrical Engineering and Computer Science, VSB-Technical University of Ostrava, 17. listopadu 2172/15, 70800 Ostrava, Czech Republic; Department of Computer Science, Faculty of Electrical Engineering and Computer Science, VSB-Technical University of Ostrava, 17. listopadu 2172/15, 70800 Ostrava, Czech Republic; Department of Computer Science, Faculty of Electrical Engineering and Computer Science, VSB-Technical University of Ostrava, 17. listopadu 2172/15, 70800 Ostrava, Czech Republic; Department of Immunology, Faculty of Medicine and Dentistry, Palacký University Olomouc and University Hospital Olomouc, Hnevotinska 3, 77900 Olomouc, Czech Republic; Department of Computer Science, Faculty of Electrical Engineering and Computer Science, VSB-Technical University of Ostrava, 17. listopadu 2172/15, 70800 Ostrava, Czech Republic; Department of Immunology, Faculty of Medicine and Dentistry, Palacký University Olomouc and University Hospital Olomouc, Hnevotinska 3, 77900 Olomouc, Czech Republic; Department of Radiology, Faculty of Medicine and Dentistry, Palacký University Olomouc and University Hospital Olomouc, Zdravotníků 248/7, 77900 Olomouc, Czech Republic; Department of Hemato-oncology, Faculty of Medicine and Dentistry, Palacký University Olomouc and University Hospital Olomouc, Zdravotníků 248/7, 77900 Olomouc, Czech Republic; Department of Hemato-oncology, Faculty of Medicine and Dentistry, Palacký University Olomouc and University Hospital Olomouc, Zdravotníků 248/7, 77900 Olomouc, Czech Republic; Department of Immunology, Faculty of Medicine and Dentistry, Palacký University Olomouc and University Hospital Olomouc, Hnevotinska 3, 77900 Olomouc, Czech Republic

**Keywords:** next-generation sequencing, structural variants, annotations, LongReadChecker (LoReC) toolkit, long-read technology

## Abstract

**Background:**

Structural variants (SVs) are increasingly recognized as key contributors to human diseases. However, our understanding of SVs in health and disease is limited, mainly due to their structural complexity and variable length in individuals, as well as limitations inherent to the available genomic technologies and reference genome used.

**Results:**

To systematically evaluate SVs across human whole-genome samples using hg38/GRCh38 and gapless T2T-CHM13 references, we introduced an innovative multiplatform approach, LongReadChecker (LoReC), which advances SV comparison and annotation based on distance variance, intersection, gene overlap, and the closest SV in the clinical database. Comparison of the performance in detecting SVs from public and our own whole-genome datasets from short-read sequencing (SRS), available long-read sequencing (LRS) platforms, and optical genome mapping (OGM) revealed that most SVs detected by SRS were confirmed by LRS, but LRS can identify twice as many SVs (25,000 SVs/genome) with greater read mapping accuracy. Our LongReadChecker (LoReC) analysis further highlights the utility of the T2T-CHM13 reference in SV detection, as 20% more deletions and 20% less insertions were detected compared with hg38/GRCh38, which was particularly evident in long-read datasets. Since 80% of the SVs detected by LRS/SRS are smaller than 0.5 kbp, OGM did not detect them.

**Conclusions:**

Our study revealed that introducing distance variance, intersection, gene overlap, and the closest SV in the clinical database may help compare and annotate SVs in diagnostics. Our data showed that LRS, together with T2T-CHM13 gapless sequences, can improve the diagnostics of patients with human diseases when SRS fails to identify the cause.

## Graphical Abstract

**Graphical Abstract:**
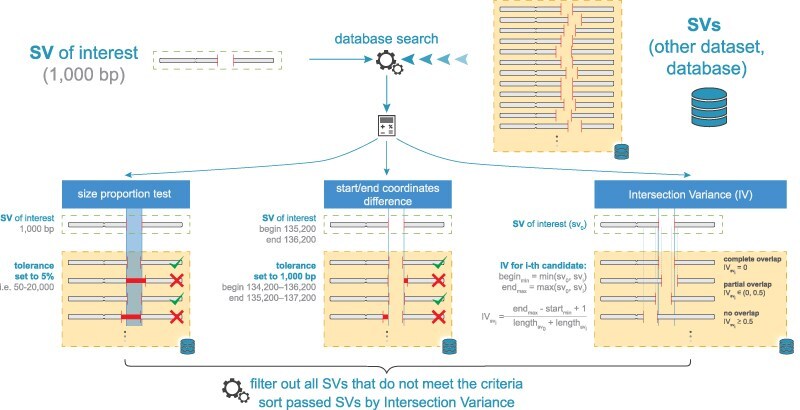

## Introduction

Structural variants (SVs) are a major source of human genetic diversity and arise from the breakdown and rejoining of DNA fragments, which can lead to the loss, gain, and rearrangement of genes and regulatory elements [1]. Since SVs are larger than 50 bp and can affect thousands to millions of nucleotides [2, 3], they are expected to have a strong effect on transcriptional regulation in health and disease [4]. Structural variant detection is of particular importance, as they are responsible for more than 25% of all rare protein truncations in a genome and are associated with many diseases [5, 6]. However, SV characterization and functional interrogation have largely lagged behind single-nucleotide variations and small insertions and deletions (INDELs), mainly due to their structural complexity and variable length in individuals as well as limitations inherent to the available genomic technologies [7]. Traditionally, SVs in the human genome have been detected using array-based methods or locus-specific assays for targeted regions [8].

Currently, great progress is being made in the detection of SVs using short-read sequencing (SRS), which remains essential due to practical and cost considerations in clinical diagnostics [9]. Novel genomic technologies for SV detection are also being rapidly developed. Among them, long-read sequencing (LRS) has demonstrated a high potential for detecting SVs [10] through longer reads and increased accuracy compared with SRS [1]. The increasing throughput, lower prices, and portability of LRS technologies increase the potential of LRS introduction into diagnostic testing, particularly for patients with genetic disorders with negative results using SRS [11, 12]. However, accurate and precise identification of SVs in specific samples and/or across samples is challenging [13]. In addition, data on the performance and comparison of currently available genomic technologies, the clinical utility of the novel human reference assembly T2T-CHM13 (T2T), and clinical databases for the annotation of detected SVs are incomplete.

Therefore, this study focused on conducting a comprehensive comparison of SVs detected from whole-genome datasets, both public and our own, obtained using SRS, LRS from currently available technologies, and optical genome mapping (OGM). To achieve this, we introduced an innovative bioinformatics approach, LongReadChecker (LoReC) [14], enabling comparisons of SVs across whole-genome datasets, technologies, and reference genomes, including their annotations using clinical genomic databases. This multiplatform approach revolutionizes the comparisons of SVs by introducing key parameters, such as distance variance, intersection, and gene overlap between datasets, thus advancing their comparison and annotation across samples or technologies or clinical databases. Our study further highlights the utility of T2T reference and long-read technologies in clinical and research applications.

## Materials and Methods

### Public and our own human whole-genome datasets

This study evaluated whole-genome datasets from a human DNA standard (NA12878 cell line, also known as HG001; B-lymphocyte; female; healthy; Genome in a Bottle Consortium [GIAB]) [15] and a breast cancer cell line (SKBR3) [16] obtained by (i) traditional SRS (Illumina), (ii) true LRS on a single-molecule real-time platform from Pacific Biosciences (LRS-PacBio), (iii) true LRS from Oxford Nanopore Technologies (LRS-ONT), (iv) synthetic LRS from transposase enzyme-linked LRS (LRS-TELL-Seq; Universal Sequencing Technology), (v) synthetic LRS from Illumina Complete Long-Reads (LRS-ICLR; Illumina), and (vi) synthetic LRS from the 10× assay (LRS-10×; 10× Genomics). All synthetic LRSs were sequenced on an Illumina short-read platform. In addition, OGM from Bionano Genomics was used (Supplementary Table S1). The principles of the LRS technologies used are described elsewhere [17]. Our own datasets from SRS, LRS-TELL-Seq, LRS-ICLR, and OGM were obtained for 2 diagnostic samples, P3 (pheochromocytoma adrenal medulla tissue, man, 63 years) and S48 (enriched CD138^+^ myeloma cells from bone marrow aspirate, woman, 39 years, IgG lambda, stage IIIA, ISS II), as well as for the NA12878 cell line. Raw sequencing data for SKBR3 cell lines for different technologies are available within the SRA under BioProject PRJNA476239 [16]. Raw sequencing data for NA12878 are available within the GIAB FTP release and under BioProject PRJNA200694 [15, 18].

For our own analysis, the high-molecular-weight DNA from tumor tissues and cells from cell lines was isolated from agarose plugs as reported previously [19]. Next-generation sequencing (NGS) libraries were prepared according to the manufacturer’s recommendations for LRS-ICLR, LRS-TELL-Seq, and SRS TruSeq DNA PCR-Free (Illumina) and sequenced (150-bp paired-end reads) on a NovaSeq 6000 system (Illumina). In addition, OGM labeling and measurements using the Bionano Saphyr instrument (Bionano Genomics) were performed as reported previously [19]. The sequence depth was approximately 30× for LRS and SRS and 300× for OGM.

### Bioinformatic processing of whole-genome datasets from different short-read sequencing and long-read sequencing platforms

To minimize the difference in precision, recall, and F1-score metrics using different callers/aligners, we used the LRS aligner Minimap2 [20] and the SV caller Sniffles2 (v2.2) software [21] for true LRS and the LongRanger software (v2.2.2) [22] for synthetic LRS analyses, which provided a strong basis for SV pipeline calling in LRS [23]. Moreover, this combination is the basis for the Illumina DRAGEN analysis of Illumina LRS-ICLR, an approach that was also compared in this study. For SRS datasets, the BWA aligner software (v0.7.17) [24] and the Manta structural variant caller (v1.6.0) [25] were used. Raw data from OGM were analyzed using Bionano Access (v1.8) software by Bionano Genomics, and the *de novo* assembly pipeline was performed using Bionano Solve tools (v3.8) (Bionano Genomics). The hg38 (GRCh38.p14) and T2T-CHM13 (v2.0) human reference genomes were used. Gene and pseudogene coordinates, names, and biotypes (e.g., protein coding) are based on MANE transcripts from RefSeq NCBI annotations (version 110); in all analyses, the Y chromosome and alternate (ALT) contigs were excluded. The list of medically relevant genes is derived from the DisGeNET [26] database (DisGeNET v20.1), which includes information on gene/variant–disease associations (VDAs) originating from ClinVar, the GWAS Catalog, UniProt, GAD, and BeFree data [26]. The selection of medically relevant genes is based on gene–disease associations (GDAs) and VDAs, GDA/VDA *>*0.5, and evidence index *>*0.8, indicating that most publications support GDA/VDA [26].

### Comparison of structural variants using the LongReadChecker toolkit

**Figure 1:**
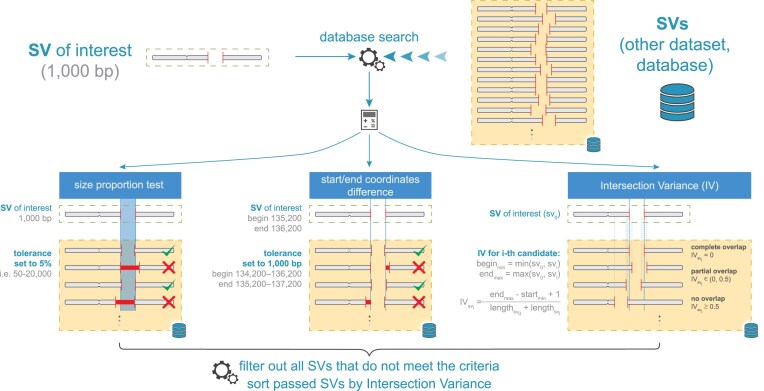
Comparison and annotation of structural variants (SVs) detected in the short-read and long-read sequencing datasets in clinical diagnostics using the LoReC toolkit. Due to the high variability in SV breakpoints, 3 parameters are needed for the SV comparison with SVs from clinical databases, such as the distance in base pairs between the sample SV and the database SV (start and end coordinate difference), the intersection (intersection factor), and the minimum overlap between the sample SV(s) and the database SV(s) (size proportion).

For comparison of SVs across different samples, technologies, and databases, we designed our own LoReC toolkit, containing 2 tools: the LoReC comparator (source file: variant call format, vcf) and LoReC coverage (source file: mapped reads in binary format, bam). The LoReC comparator can find the closest SV detected by another technology, database, or reference genome for each SV across the whole genome or region(s) of interest. For each comparison, the following parameters need to be established: (i) the distance variance threshold (the accepted difference in base pairs between the start and end coordinates between each SV in dataset 1 and the nearest SV in dataset 2, expressed as the sum of the 2 differences), (ii) the intersection factor (the overlap between each SV in dataset 1 and the nearest SV in dataset 2; 0 = 100% overlap, 0–0.5 = partial overlap, *>*0.5 = no overlap), and (iii) the minimal size proportion (the percentage of the size in base pairs between each SV in dataset 1 and the nearest SV in dataset 2; e.g., 5% means that the SVs in dataset 1 encompass at least 5% of the SVs in dataset 2 or vice versa). To compare SVs in the same regions detected by different technologies, which may differ in size and coordinates because of the different principles of the method, the LoReC toolkit lists the closest SV of the same type (e.g., deletion, insertion) from dataset 2 and its coordinates, distance, intersection factor, and size proportion compared with the SV from dataset 1. A distance variance threshold of 1,000 bp was used for a comparison between different NGS platforms and 50,000 bp between NGS and OGM, and an intersection factor of 0 to 0.5 and a minimum size fraction of 5% were used to indicate SVs that overlap and are very similar; different parameters can be used for filtering (Fig. 1). To visually inspect the SVs of interest, the Samplot tool was used [27].

**Figure 2:**
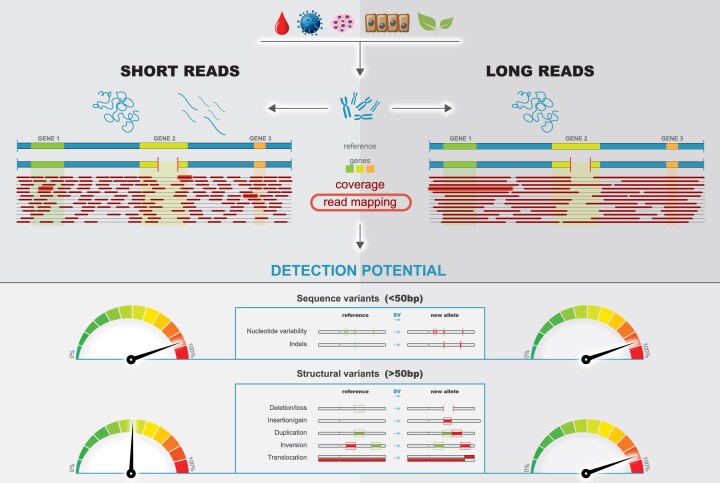
Principles of short-read sequencing (SRS) and long-read sequencing (LRS) and their detection potential for sequence and structural variants. An example of the coverage and read mapping for a heterozygous gene deletion is shown; LRS requires DNA with a high-molecular-weight. The lower diagram shows the detection potential for sequence and structural variants using SRS and LRS.

The LoReC coverage tool is able to provide coverage of the gene/region of interest, including statistics (mean, median, min, max, and Q1 and Q3 coverages), filtering reads based on the mapping quality, and visualization of the regions of interest. The LoReC coverage outputs are as follows: (i) coverage across regions of interest or across the whole genome based on the coordinates and gene names given in a region file, (ii) coverage calculations based on the read mapping quality (MAPQ) threshold value, which is able to filter out the reads that map to multiple regions or those of poor quality (MAPQ0 = high probability that a read is mapped to multiple locations with an equal score, MAPQ1 = high probability that a read is mapped to at least 2 locations, MAPQ50 = 99.999% probability that a read is mapped to a unique region; Fig. 2), and (iii) visualization of the regions of interest specified in the region files, which allows a comparison of multiple technologies, different samples, or reference genomes. Low-coverage genes are those in which Q1 coverage was below 25% of the mean genome coverage of the sample.

**Figure 3:**
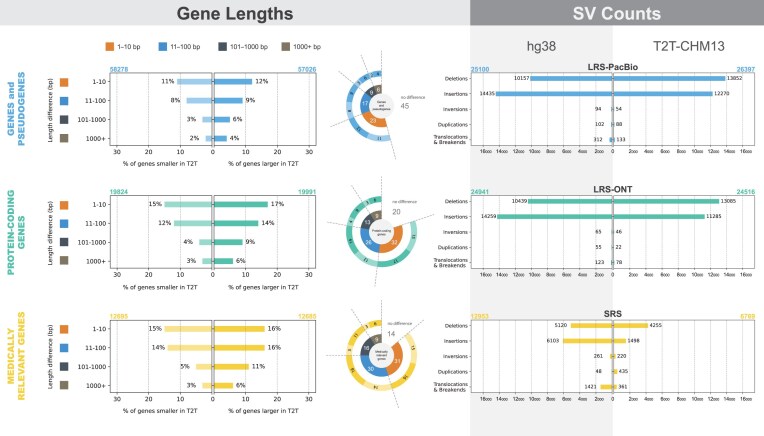
Comparison of gene lengths and the number of structural variants (SVs) in the NA12878 cell line using hg38 and T2T-CHM13 references. The differences in the number and lengths of genes and pseudogenes, protein-coding genes, and medically relevant genes for both references are presented; genes were annotated based on RefSeq NCBI (version 110, based on gene IDs and MANE transcript), excluding genes on the Y chromosome and ALT contigs. The number of SVs detected by long-read sequencing (LRS-PacBio, LRS-ONT) and short-read sequencing (SRS) on both references is shown on the right.

### Annotation of structural variants using the LongReadChecker toolkit

Another functionality of the LoReC comparator is to annotate detected SVs based on the annotation file(s) from dbVar (NCBI) [28, 29]. As the current dbVar_common and ClinVar databases are based on hg38, SV annotations were performed primarily on this reference. For experimental purposes, dbVar_common and ClinVar annotation files were converted from hg38 to the CHM13-T2T reference genome using BCFtools/liftover [30]. The following parameters were established for SV comparisons: a threshold of ±1,000 bp in the distance between the start and end coordinates of the SVs compared, an intersection factor threshold of 0.5, and a minimal size proportion of 1%. First, the detected SVs are compared with SVs included in the dbVar_common database of common SVs found with a frequency *>*1% in the population (e.g., nstd186, NCBI Curated Common Structural Variants) or any other vcf file of interest and must fulfill the setup criteria to be marked as PASS. Second, detected SVs not found in the dbVar_common database are compared with the ClinVar SV database (e.g., nstd102, Clinical Structural Variants) or vcf of interest, and for SVs present in the database, the clinical significance of SVs is reported (e.g., pathogenic, likely pathogenic, variant of uncertain significance [VUS], likely benign, benign). For each SV, LoReC provides the main annotation (ClinVar accession ID) with the lowest intersection factor (=highest overlap) that passed the selected filters. In addition, LoReC provides all passed ClinVar accession IDs that meet filtering criteria for further investigation. For each gene, it is possible to filter its SVs in output tables generated by the LoReC toolkit. For SVs not found in the ClinVar SV database and with an overlap of at least 1 gene, additional information is reported through the LoReC toolkit based on the NCBI annotation file, which includes the biotype, gene description, and gene/VDAs present in DisGeNET, allowing filtering based on these features [31].

## Results

### True long-read sequencing technologies have superior performance in detecting structural variants

To evaluate the performance of the currently available LRS technologies and SRS, whole-genome sequencing datasets for the NA12878 healthy [15] and SKBR3 breast cancer [16] cell lines and 2 diagnostic tissue samples, P3 (pheochromocytoma tissue) and S48 (multiple myeloma bone marrow aspirate), both public and our own (Supplementary Table S1), were compared using different LRS and SRS platforms. Among the tested platforms were the following: (i) 2 true LRS technologies (LRS-PacBio and LRS-ONT); (ii) 3 synthetic linked-read LRS approaches (LRS-ICLR, LRS-10×, and LRS-TELL-Seq), sequenced on the short-read Illumina platform; (iii) SRS on Illumina; and (iv) OGM from Bionano Genomics. For comparisons of detected SVs, as well as their types, coordinates, sizes, intersection factors, size proportions, and coverage between different technologies, reference genomes (hg38/GRCh38.p14; T2T-CHM13, v2.0), and annotations of detected SVs according to clinical databases, a LoReC toolkit was used (Fig. 1). For more details on functionalities, see the Materials and Methods.

Using hg38, approximately 25,000 SVs per genome were detected using LRS-PacBio and LRS-ONT, 14,000–15,000 using LRS-ICLR, 10,000–12,500 using LRS-10×, 12,500–15,000 using SRS, and 4,000 using OGM. Insertions were the most common SVs detected by all technologies: 14,000 per genome using LRS-PacBio and LRS-ONT, 5,000–6,000 using LRS-ICLR, 6,000–7,000 using SRS, and 2,500 using OGM. Insertions were not detected in the LRS-TELL-Seq and LRS-10× datasets, as the LongRanger pipeline available to analyze synthetic reads cannot call insertions. Deletions were the second most common SVs: 10,500 per genome using LRS-PacBio and LRS-ONT, 9,000–10,000 using LRS-ICLR, 3,500–5,000 using LRS-10×, 5,000–6,000 using SRS, and 1,250 using OGM. Regarding other SVs, such as inversions, duplications, and breakends/translocations, their counts varied across samples and genomes, ranging from 240 to 1,430 per genome, depending on the technology (Supplementary Table S2, Fig. 3, Supplementary Fig. S1). Selected SVs from the OGM and LRS datasets were verified using fluorescence immunophenotyping and interphase cytogenetics as a tool for the investigation of neoplasms (FICTION), arrayCGH, and/or targeted SRS.

When comparing available technologies, most deletions and insertions ($\sim$80%–95%) detected by LRS-ONT were confirmed by LRS-PacBio, whereas higher concordance ($\sim$95%) was observed using the PacBio high-fidelity (HiFi) sequencing mode and lower concordance ($\sim$80%) using continuous long reads. However, less than 50% were detected by SRS and 55%–71% by LRS-ICLR. Furthermore, LRS-TELL-Seq and LRS-10× confirmed approximately 25%–50% of the deletions detected by LRS-ONT/LRS-PacBio, but it was not possible to call insertions from synthetic reads. Regarding inversions, duplications, and breakends/translocations, the best overlap was observed between LRS-PacBio and LRS-ONT, and a moderate overlap was identified using synthetic LRS and SRS (Supplementary Tables S3 and S4).

### Most structural variants detected by short-read sequencing were confirmed by long-read sequencing

Next, we compared the SVs detected by SRS, the most widely used platform today in clinical diagnostics, with SVs detected by LRS-ONT, LRS-PacBio, and synthetic LRS. Most of the deletions and insertions detected by SRS (10,000–12,500 per genome) were smaller than 0.5 kbp ($\sim$80%) and were confirmed by LRS-PacBio/LRS-ONT ($\sim$90%). Although LRS-ICLR was similar to true LRS in detecting deletions, it had less precision for insertions ($\sim$50%), as this technology is based on SRS. Notably, SRS failed to detect approximately 50% of the SVs detected by LRS (Supplementary Tables S3 and S5).

### True long-read sequencing and short-read sequencing technologies have superior performance over optical genome mapping

We also compared LRS and SRS with OGM, a nonsequencing technology based on the labeling of high-molecular-weight DNA using fluorophore tags on specific sequence motifs. As OGM does not detect SVs smaller than 0.5 kbp or SVs in genomic regions that lack specific sequence motifs, OGM detected a lower number of SVs than LRS or SRS (hg38: 3,946 vs. 24,941 vs. 12,953; T2T-CHM13: 3,082 vs. 24,516 vs. 6,769). Most ($\sim$80%) deletions and insertions detected by OGM were confirmed by LRS-ONT and LRS-PacBio; SRS confirmed approximately 35% of the insertions and approximately 50%–60% of the deletions detected by OGM (Supplementary Tables S3 and S6). Comparison of OGM with LRS and SRS showed that OGM does not provide the exact coordinates of individual SVs, and their position may be substantially different from the coordinates detected by LRS and SRS (Supplementary Fig. S2).

### True long reads map with high probability to unique regions compared with short reads

Next, we evaluated the MAPQ [24], a measure of the probability that a read is misplaced, for different LRS and SRS technologies. For MAPQ0, a default setting in the current SRS/LRS aligners and variant callers that allows read mapping to multiple regions, most genes were covered by all SRS/LRS technologies (Fig. 2, Supplementary Fig. S3, Supplementary Table S7). To eliminate problematic regions with misplaced reads, current SRS pipelines often mask these repetitive dark regions. When stricter MAPQ1 and MAPQ50 (associated with a lower probability of misplaced reads than MAPQ0) were applied, many regions of the genome were not covered in the SRS datasets, including many protein-coding genes and medically relevant genes (Fig. 2, Supplementary Fig. S3, Supplementary Table S7). In LRS-PacBio HiFi datasets, most reads were mapped with high probability to a unique region, as demonstrated by applying MAPQ1/MAPQ50. Regarding LRS-ONT, mapping to multiple regions is less probable because of the very long reads (up to Mbp); however, many reads are low quality, resulting in less accuracy in mapping (Fig. 2, Supplementary Fig. S3, Supplementary Table S7).

### T2T-CHM13 reference improves the analysis of structural variants for long-read sequencing and short-read sequencing datasets

To understand the added value of the gapless T2T-CHM13 reference with the currently used hg38, SVs in known genes and pseudogenes, protein-coding genes, and medically relevant genes and their lengths were compared for the LRS and SRS datasets, excluding the Y chromosome and ALT contigs (Fig. 3, Supplementary Table S7). For comparison between references, unique gene IDs from RefSeq NCBI annotations (version 110) using the main MANE transcript were used, which also enables comparison of gene paralogs. Using the T2T-CHM13 reference, the number of deletions increased by more than 20%, and the number of insertions decreased by more than 20% using the LRS-ONT and LRS-PacBio datasets compared with hg38 (Fig. 3, Supplementary Fig. S1, Supplementary Table S2). Using SRS, approximately 10% fewer deletions and 80% fewer insertions were detected using T2T-CHM13 than using hg38 (Fig. 3, Supplementary Fig. S1, Supplementary Table S2). In the unique regions of T2T-CHM13, numerous translocations, deletions, and other SVs were detected in the telomere, centromere, and subcentromere regions in all analyzed datasets (Supplementary Tables S4–S6, Supplementary Fig. S4).

**Figure 4:**
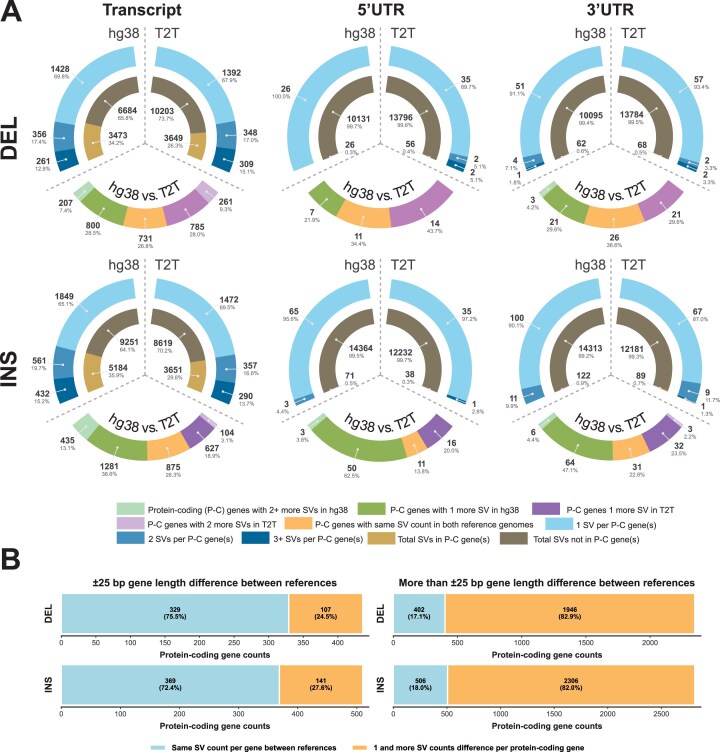
Comparison of detected structural variants (SVs) in the NA12878 cell line located in (A) protein-coding sequences (MANE transcripts) and 5′-UTR and 3′-UTR regions for both hg38 and T2T-CHM13 (T2T) reference genomes and (B) genes with length differences within ±25 bp and greater than ±25 bp between reference genomes hg38 and T2T-CHM13.

Of the detected SVs, approximately 27%–33% were located in the protein-coding sequences and approximately 0.5% in each of the 5′-UTR and 3′-UTR regions, with the remainder in the noncoding regions (Fig. 4, Supplementary Table S8). The T2T-CHM13 reference also refined the length of the genes. Comparing T2T-CHM13 and hg38 references for 54,553 genes and pseudogenes that overlap based on the gene ID (Fig. 3, Supplementary Table S7), 45.6% were the same length, 23.7% differed by 1–10 bp, 16.8% by 11–100 bp, 8.2% by 101–1,000 bp, and 5.6% by more than 1,000 bp. Regarding protein-coding genes (medically relevant genes), 19.9% (14.3%) were the same length, 31.7% (31.0%) differed by 1–10 bp, 26.7% (30.2%) by 11–100 bp, 13.3% (15.8%) by 101–1,000 bp, and 8.4% (8.8%) by more than 1,000 bp (Fig. 3). For genes of similar length (±25 bp), minimal differences in SV counts were observed between the 2 references, whereas larger differences in length were associated with substantial differences in SV counts (Fig. 4, Supplementary Table S8). Among those with the largest differences in length were *GRK1* [32] and *LPA* [33], and many other genes, such as *SMN1&2* [34], *DUX4*, and *HLA-DRB5* or *GBA* and its pseudogene *GBAP1*, were disassembled, not correctly assembled, or highly similar in hg38 (Supplementary Fig. S5, Supplementary Fig. S6, Supplementary Table S7). Furthermore, an additional 167 protein-coding genes were annotated in T2T-CHM13 than in hg38 (Supplementary Table S7). To complement the added value of T2T-CHM13, we marked the genes found in the discrepant regions between hg19 and hg38 [35, 36] (Supplementary Table S7).

### Annotation of structural variants in clinical databases is challenging

To annotate the SVs detected by the tested technologies, we used a LoReC comparator toolkit to compare the detected SVs with the dbVar_common (NCBI dbVar Curated Common Structural Variants, GnomAD, and other sources, ID: nstd186) [28] and ClinVar (ID: nstd102) clinical datasets [37]. The dbVar_common database includes SVs that occur with a frequency of > 1% in the population. As the current ClinVar database is based on hg38, annotations of SVs were primarily performed using this reference. Given that SV coordinates may vary from individual to individual, the following parameters need to be defined prior to each analysis: (i) the distance variance threshold (acceptable difference in base pairs between the SV of interest and the SV in the clinical database), (ii) the intersection factor (overlap between the SV of interest and the nearest SV in the clinical database), and (iii) the minimum size fraction (minimum proportion of the SV of interest and the nearest SV in the clinical database) (see the Materials and Methods for details). All SVs were also evaluated using the AnnotSV tool [38] (Table 1, Fig. 1).

**Table 1:** Annotations of deletions detected in whole-genome datasets for the NA12878 and SKBR3 cell lines and P3 and S48 diagnostic samples by SRS and LRS technologies employing (A) LoReC using dbVar_common and ClinVar and (B) AnnotSV, both for the hg38 reference.

All		(A) LoReC*							(B) AnnotSV			
Sample	Technology	Total	dbVar_common	ClinVar	Pathogenic/	VUS	Benign/	Not	Total	Benign	Pathogenic/	VUS
		DEL			likely		likely	annotated	DEL+BND		likely	
					pathogenic		benign				pathogenic	
**NA12878**	SRS	5,120	3,332 (65%)	594 (12%)	29/11	62	461/19	1,734 (34%)	5,525	4,598 (83%)	32/6	889 (16%)
	LRS-PacBio	10,157	4,696 (46%)	1,085 (11%)	41/19	91	879/39	5,220 (51%)	10,157	6,030 (59%)	7/6	4,114 (41%)
	LRS-ONT	10,439	4,692 (45%)	1,090 (11%)	42/21	90	883/36	5,519 (53%)	10,439	5,893 (56%)	2/7	4,537 (43%)
	LRS-ICLR	9,412	4,018 (43%)	832 (9%)	42/18	75	655/25	5,199 (55%)	9,412	4,767 (51%)	2/4	4,639 (49%)
**SKBR3**	SRS	2,920	2,261 (78%)	549 (19%)	65/9	91	358/17	570 (20%)	2,890	2,373 (82%)	46/32	457 (16%)
	LRS-PacBio	9,097	4,168 (46%)	932 (10%)	62/15	98	710/33	4,693 (52%)	9,097	6,095 (67%)	30/10	2,962 (33%)
	LRS-ONT	10,983	4,343 (40%)	979 (9%)	64/18	98	748/32	6,402 (58%)	10,983	7,183 (65%)	29/15	3,756 (34%)
**P3**	SRS	5,018	3,222 (64%)	580 (12%)	28/8	72	437/22	1,741 (35%)	5,478	4,463 (81%)	36/4	975 (18%)
	LRS-ICLR	9,166	3,899 (43%)	756 (8%)	35/16	82	579/30	5,095 (56%)	9,166	4,486 (49%)	0/3	4,677 (51%)
**S48**	SRS	5,632	3,480 (62%)	652 (12%)	39/12	82	480/24	2,069 (37%)	6,199	5,035 (81%)	84/6	1,074 (17%)
	LRS-ICLR	9,279	3,970 (43%)	811 (9%)	49/13	89	613/29	5,102 (55%)	9,279	4,715 (51%)	5/6	4,553 (49%)

BND, breakends; DEL, deletion; LRS-ICLR, synthetic long-read sequencing by Illumina—complete long-read technology on the Illumina platform; LRS-ONT, true long-read sequencing by Oxford Nanopore Technologies; LRS-PacBio, true long-read sequencing by Pacific Biosciences; LRS-TELL-Seq, synthetic long-read sequencing by Universal Sequencing Technology on the Illumina platform; LRS-10×, synthetic long-read sequencing by 10× Genomics on the Illumina platform; OGM, optical genome mapping by Bionano Genomics; SRS, short-read sequencing by Illumina platform; VUS, variant of unknown significance. *The main annotation with the lowest intersection factor (the highest overlap) that passed the selected filters between the sample and the database.

Regarding deletions, 62%–78% of deletions detected by the SRS datasets overlapped with the deletions in dbVar_common and approximately 10% with those in ClinVar. When using AnnotSV, approximately 80% of the SVs in the SRS datasets were annotated as benign, approximately 1% (38–90) of deletions per sample were annotated as pathogenic/likely pathogenic, and less than 18% were annotated as a VUS. Using true LRS, approximately 45% of deletions were detected in dbVar_common and 10% in ClinVar; for more than half of the deletions, no annotation was available (Table 1). Using ClinVar annotations, most SVs in this database span very large regions containing many genes; therefore, we used the size proportion parameter of 1% for LoReC annotations. Using this threshold, the LoReC annotations were consistent with the AnnotSV results: 49%–67% of SVs in the LRS datasets were annotated as benign, approximately 10 deletions ($\sim$0.1%) were annotated as pathogenic/likely pathogenic, and approximately 4,500 deletions (30%–50%) were annotated as a VUS, depending on the sample and technology used (Table 1). Additionally, LoReC observed more pathogenic/likely pathogenic SVs in LRS datasets (Table 1). Regarding insertions, the dbVar_common/ClinVar databases contain only breakpoints and lack information on the insertion length for most insertions, making annotation impossible, which is also problematic for inversions and translocations. For information regarding annotations using the T2T-CHM13 reference genome, see Supplementary Table S9.

### Annotation of the structural variants not present in the clinical databases by LoReC

**Figure 5:**
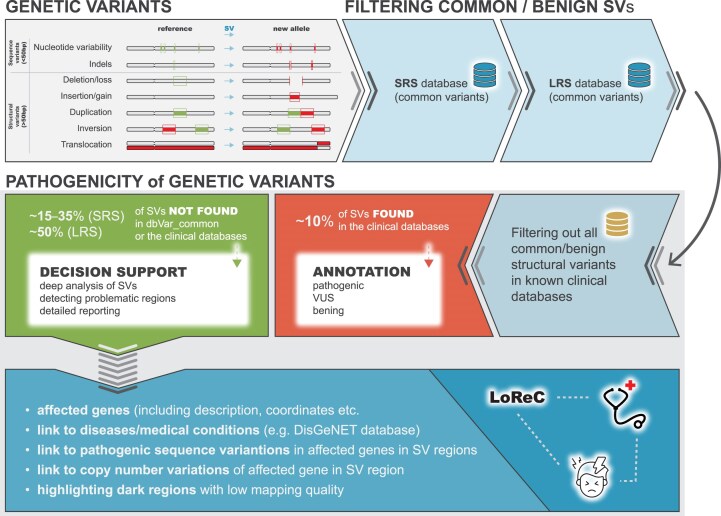
Suggested workflow for annotation of structural variants (SVs) detected by long-read sequencing in diagnostic samples. First, SVs in the samples are filtered using a dbVar_common database or similar to filter out common and benign SVs. The SVs not found in the dbVar_common database are compared with the ClinVar SV database or similar. For SVs not found in the ClinVar database, the LoReC toolkit is used for the comparison and annotation of SVs in the samples based on the NCBI annotation file(s), including the affected gene (gene description, coordinates) and a link to the disease or medical condition based on the gene/variant–disease associations present in DisGeNET or similar.

We suggest a workflow for diagnostic laboratories to annotate SVs from LRS/SRS. Briefly, after filtering out benign/common SVs present in the clinical databases (currently, mainly based on SRS, but in the future, LRS datasets will be added; e.g., dbVar), the remaining SVs are compared with the ClinVar clinical database or similar (Fig. 5). For SVs not presented in the dbVar_common/ClinVar databases, our LoReC toolkit enables the addition of custom annotations for regions/genes within the detected SVs. In particular, genes may be linked to the gene/VDAs provided in the annotation file (e.g., DisGeNET) or other custom annotations (Supplementary Table S7). In addition, the LoReC toolkit provides coverage for genes and regions for selected mapping quality and information about the SV localization (e.g., protein-coding region, medically relevant genes), enabling the visualization of regions of interest for expert evaluation.

## Discussion

The importance of SVs in clinical diagnostics continues to expand due to advances in genomic technologies and wet lab protocols, the introduction of the gapless reference sequence T2T-CHM13, and the release of high-quality datasets. Despite the growing importance of SVs in human diseases, our knowledge of SVs in health and disease is limited, largely due to their structural complexity and variable length in individuals, as well as the limitations inherent in available genomic technologies. Here, we comprehensively evaluated SVs from human whole-genome datasets obtained from SRS, alongside all available LRS and OGM platforms across different samples, technologies, and clinical databases using 2 human references (hg38 and T2T-CHM13). Moreover, our multiplatform approach enables annotation of detected SVs to support the implementation of SV diagnostics in clinical practice. Despite advances in clinical genetic diagnostics, approximately 50% of all suspected Mendelian diseases remain unresolved [39, 40]. The diagnostics of SVs is relevant not only in rare genetic diseases; SVs are also important contributors to chronic diseases, including cancer [5, 6, 41]. However, the technical limitations inherent in the available genomic technologies, as well as the structural complexity of SVs and their variable length in individuals, have led to an incomplete characterization of SVs in the human genome in relation to health and disease compared with single-nucleotide variations and small INDELs. To gain more insights into the performance of available genomic technologies, we compared our own and public whole-genome datasets from SRS, 2 LRS platforms (PacBio-LRS, ONT-LRS) requiring specific instruments, and 3 LRS approaches utilizing synthetic long reads sequenced on conventional short-read NGS. To achieve this, we developed the multiplatform LoReC toolkit, which compares the size and type of SVs, as well as their overlap, coverage, coordinates, affected genes, and disease associations from different SRS and LRS datasets in specific samples and/or across samples and databases, regardless of which of the many available algorithms was used to detect SVs [42, 43].

The LoReC toolkit was developed to provide comprehensive SV comparison and annotation between samples, technologies, and databases for use in diagnostic laboratories, thereby extending the functionalities of SV comparison toolkits such as Jasmine [44] and SURVIVOR [45] (Supplementary Table S10). First, we were interested in the performance of SRS compared with third-generation technologies. On average, approximately 13,000 SVs/genome were detected by SRS and twice as many ($\sim$25,000 SVs/genome) by LRS. The lower performance of SRS in SV detection may arise from mapping short reads to multiple regions, discordant pairs, and split reads, which limit SV detection by current variant callers [46, 47]. Notably, most of the SVs detected by SRS were also detected by LRS. Our findings are consistent with those of others, showing that LRS can identify hidden disease-related SVs that are not detected by SRS [3, 36, 46]. Since 80% of the SVs detected by LRS/SRS were smaller than 0.5 kbp, OGM did not detect most of the SVs detected by LRS/SRS. The most common type of SVs were deletions and insertions, whereas half as many deletions ($\sim$5,000 vs. $\sim$10,000) and insertions ($\sim$6,000 vs. more than 12,000) per sample were detected by SRS compared with LRS. Our data reveal that LRS-ONT and LRS-PacBio technologies outperform SRS, the synthetic read LRS-ICLR, LRS-10×, LRS-TELL-Seq, and OGM in detecting SVs. In addition to detecting more SVs through LRS, longer reads map more uniquely to the genome than SRS. Using the default MAPQ0 settings in current SRS/LRS aligners and variant callers, which allow the use of misplaced reads, effective coverage of most genes across genomes was achieved for all SRS/LRS technologies. To address this issue, current SRS pipelines often mask these problematic dark regions, which include repetitive elements and polymorphic regions, potentially leading to the loss of key information in these regions. In addition, PacBio provides a BED file for dark regions that occur in repetitive areas or areas with high GC content. When stricter MAPQ1 or MAPQ50 was applied, many regions across the genome were not covered well in the SRS datasets, including numerous protein-coding and medically relevant genes. Of the technologies analyzed, the lowest probability of mismatches was observed for the data obtained by LRS-PacBio HiFi, the highest for SRS. The LRS-ONT datasets demonstrated a high percentage of low-quality reads due to lower nucleotide accuracy when using the MAPQ0 setting. When using MAPQ1 or MAPQ50 to filter out low-quality reads, LRS-ONT achieved uniform coverage across the whole genome with high-quality and very long reads. Notably, LRS-ONT has introduced adaptive sampling, a computational enrichment technique that enriches regions of interest by selectively rejecting off-target molecules [48, 49], enabling deeper coverage in these regions [50].

Another key step for clinical genetics is the introduction of the gapless T2T-CHM13 human reference assembly, which uncovers 8% of the dark regions of the genome, adds nearly 200 million bases and predicts 99 novel protein-coding genes compared with reference hg38 [51]. Our comprehensive bioinformatic analysis of whole-genome datasets further supports the clinical utility of using the T2T-CHM13 sequence for medical diagnostics. When we compared medically relevant genes using coordinates based on NCBI RefSeq annotations on both references, approximately 85% of the genes changed in size and more than half by more than 10 bp. Notably, approximately 9% of the medically relevant genes differed by > 1,000 bp between hg38 and T2T-CHM13. Among the genes significantly differing between both references were those that were disassembled, not correctly assembled, or highly similar in hg38, such as the challenging medically relevant genes *GRK1, LPA, SMN1/2, DUX4* , and *HLA-DRB5* or the *GBA* gene and its pseudogene *GBAP1* and others [52].

For example, hg38 contains only 6 copies of the 5.5-kb KIV-2 repeat in *LPA*, even though human genomes carry $\sim$5 to > 50 copies [33]; it also omits the complete macrosatellite region containing > 20 D4Z4 repeats at the *DUX4* locus [53]; and does not represent the structurally complex *SMN1/SMN2* locus and its variable copy numbers [54]. Furthermore, the novel T2T-CHM13 reference resulted in approximately 20% more deletions and 20% fewer insertions than hg38 in LRS. It should be noted that some of the observed differences in SV counts reflect reference bias, as sequences that are minor alleles in hg38 may be common in human populations and are represented in T2T-CHM13. Given that many disease-causing SVs are located in repetitive, duplicated, inverted, or structurally complex regions that were missing, disassembled, or misinterpreted in older references and cannot be resolved using SRS [55], clinical genetics can benefit from the use of T2T-CHM13 and LRS. For example, T2T-CHM13 and LRS were used to detect previously hidden variants in undiagnosed cases of rare diseases within the European Solve-RD consortium [56], repeated expansions in cerebellar ataxia [57], and inversions disrupting the *EHMT1* gene in Kleefstra syndrome [58]. Similarly, T2T-CHM13 and LRS show promise in diseases associated with copy number variability, such as those known in the *SMN1/2* genes in spinal muscular atrophy [59] and the D4Z4 repeats in the *DUX4* locus in facioscapulohumeral muscular dystrophy [60]. Even in SRS data, the use of T2T-CHM13 improves read mapping and enhances the detection of clinically relevant rare and deleterious variants [61]. Taken together, there is growing evidence of the benefits of LRS and T2T-CHM13 for clinical medicine.

Another challenging topic is the pathogenicity annotations of thousands of SVs detected by LRS/SRS. Unlike sequence variants, for which interpretation guidelines exist [62, 63] and clinical databases such as dbVar_common/ClinVar based on large available SRS datasets, SV annotations are more difficult, not only due to the insufficient number of LRS datasets in the databases but also the substantial variability in SV breakpoints between individuals and the complexity of rearrangements. Therefore, we introduced crucial measures for SV evaluations, such as the distance in base pairs between SVs, the intersection factor, and the size proportion to facilitate comparison of SVs across datasets, references, and databases. Since most deletions in the ClinVar database cover very large regions containing many genes, a size proportion of 1% was used for LoReC annotations. Both LoReC and AnnotSV [38] have annotated many SVs using their own algorithm, but there are still many SVs not present in the current dbVar_common and ClinVar databases or other databases. It should be noted that for most SVs per genome, many of which were located in protein-coding sequences, no annotation was available in the current version of the ClinVar database. Regarding insertions and other SVs, the length of the insertion and the sequence of the insert are missing in the dbVar_common/ClinVar databases, making their annotation impossible. Unlike deletions, which remove annotated regions and can be assessed by their absence, insertions introduce sequences that are not present in the reference. Moreover, insertions frequently occur within or generate tandem repeats and segmental duplications, regions inherently difficult to assess. Consequently, insertions frequently lack reliable population frequency estimates and functional or phenotypic evidence, making confident clinical classification challenging [64]. Addressing these challenges will require systematic characterization of insertion sequences and their functional annotation, precise breakpoint resolution, and the development of tools for functional impact prediction. For SVs not annotated by ClinVar, we applied the LoReC toolkit, and with its help, the affected gene(s) can be linked with gene/variant–disease associations using DisGeNET, human phenotype ontology, or a similar database. In the future, this approach will allow SV annotations to be matched to continuously updated clinical databases based on the LRS datasets and linked to the reference T2T-CHM13.

This study and others [11, 12, 46, 50, 65–67] further support the introduction of LRS into medical diagnostics. However, LRS will not replace SRS in the near future but will complement it, especially in cases with negative SRS results, as this method will remain the gold standard for routine diagnostics due to its cost, speed, and established clinical and bioinformatic pipelines. Nevertheless, to introduce LRS into diagnostics, laboratories should collect cells for high-molecular-weight DNA isolation, as LRS cannot be performed on fragmented DNA obtained by standard isolation methods and increases computational and data storage capacity due to the large datasets it obtains. We highlight the introduction of distance variance, intersection, gene overlap, and the closest SV in the clinical database for SV comparisons and annotations, which is currently the weakest point of SV integration into clinical diagnostics. Although this first comprehensive study on the performance of all available genomic technologies is focused on SVs in the human genome, SV events are widespread in other species, and our toolkit is also suitable for these datasets.

## Conclusions

In this study, we introduced an innovative multiplatform approach for any SRS and third-generation dataset that advances SV comparisons across samples and databases, as well as annotations of SVs based on comparisons with clinical databases. Although the gold standard SRS uncovers thousands of SVs that may be clinically relevant, we demonstrated that LRS is more effective at detecting SVs than SRS. Thus, LRS is expected to complement the SRS analysis in clinical diagnostics soon, especially in cases with negative SRS results. However, the implementation of LRS will require the introduction of isolation methods, leading to high-molecular-weight DNA, and the update of clinical databases to include LRS datasets and the T2T-CHM13 reference for the correct annotations of SVs.

## Availability of Source Code and Requirements

Project name: lorec-comparator

Project homepage: https://github.com/novosadt/lorec-comparator

Operating system: Windows, Linux, macOS, Solaris

Programming language: Java 8

Other requirements: None

License: GPL-3.0 license

RRID: SCR_027211

Project name: lorec-coverage

Project homepage: https://github.com/novosadt/lorec-coverage

Operating system: Windows, Linux, macOS, Solaris

Programming language: Java 8

Other requirements: None

License: GPL-3.0 license

RRID: SCR_027210

## Additional Files

**Supplementary Table S1**. Whole-genome datasets from short-read sequencing, long-read sequencing, and optical mapping analyzed in this study.

**Supplementary Table S2**. Structural variants detected in the NA12878 and SKBR3 cell lines and the P3 and S48 diagnostic samples using SRS and different LRS technologies for the hg38 and T2T-CHM13 human references.

**Supplementary Table S3**. Comparison of deletions and insertions larger/smaller than 0.5 kbp in the whole-genome datasets detected by current long-read sequencing (LRS) and short-read sequencing (SRS) technologies and optical genome mapping (OGM). The number (in brackets, the percentage) presents the SVs that overlap with the SVs detected by base technology (A) LRS-ONT, (B) SRS, and (C) OGM detected by other technologies using hg38 and T2T-CHM13 references.

**Supplementary Table S4**. (A) Summary of structural variants (SVs) detected by LRS-ONT (=base) and confirmed by SRS, LRS-PacBio, LRS-ICLR, LRS-TELL-Seq, LRS-10×, and OGM in NA12878 and SKBR3 cell lines for both hg38 and T2T-CHM13 references. List of SVs for hg38 reference in (A) NA12878 and (B) SKBR3 and for T2T-CHM13 reference in (C) NA12878 and (D) SKBR3 cell lines. For each sample and technology, the total number of SVs in LRS-ONT (=base), the types of SVs (insertion, deletion, duplication, inversion, translocation), and the number and percentage of confirmed SVs detected by other NGS and OGM technologies based on threshold parameters (distance variance threshold of 1,000 bp, an intersection factor of 0–0.5, and a minimum size fraction of 5%) are provided.

**Supplementary Table S5**. (A) Summary of structural variants (SVs) detected by SRS (=base) and confirmed by LRS-ONT, LRS-PacBio, LRS-ICLR, LRS-TELL-Seq, LRS-10×, and OGM in NA12878 and SKBR3 cell lines and P3 and S48 diagnostic samples for both hg38 and T2T-CHM13 references. List of SVs for hg38 reference in (B) NA12878 and (C) SKBR3 cell lines and (D) P3 tissue and (E) S48 tissue diagnostic samples, and for T2T-CHM13 reference in (F) NA12878 and (G) SKBR3 cell lines and (H) P3 and (I) S48 diagnostic samples. For each sample and technology, the total number of SVs in SRS (=base), the types of SVs (insertion, deletion, duplication, inversion, translocation), and the number and percentage of confirmed SVs detected by other NGS and OGM technologies based on threshold parameters (distance variance threshold of 1,000 bp, an intersection factor of 0–0.5, and a minimum size fraction of 5%) are presented.

**Supplementary Table S6**. (A) Summary of structural variants (SVs) detected by OGM (=base) and confirmed by SRS, LRS-ONT, LRS-PacBio, LRS-ICLR, LRS-TELL-Seq, and LRS-10× in NA12878 and SKBR3 cell lines and P3, S48 diagnostic samples. List of SVs in (B) NA12878 and (C) SKBR3 cell lines, (D) P3 tissue and (E) S48 tissue diagnostic samples using hg38 reference and (F) NA12878 and (G) SKBR3 cell lines and (H) P3 tissue and (I) S48 tissue diagnostic samples using T2T-CHM13 reference. For each sample and technology, the total number of SVs in SRS (=base), the types of SV (insertion, deletion, duplication, inversion, translocation), and the number and percentage of confirmed SVs detected by other SRS/LRS technologies and OGM are presented based on threshold parameters (distance variance threshold of 50,000 bp, an intersection factor of 0–0.5, and a minimum size fraction of 5%).

**Supplementary Table S7**. Summary of genes and pseudogenes, along with their coordinates, biotypes, discrepant regions, and gene/disease associations according to the DisGeNET using hg38 and T2T-CHM13 references. The coverages and statistics for the NA12878 cell line for short-read sequencing (SRS) and long-read sequencing technologies (LRS-ONT, LRS-PacBio, LRS-ICLR, LRS-TELL-Seq, LRS-10×) and OGM are summarized. The number of labels within individual genes used in OGM is shown.

**Supplementary Table S8**. Summary of structural variants in protein-coding genes present in MANE transcripts (A, deletions; B, insertions), and 5′-UTR (C, deletions; D, insertions), 3′-UTR (E, deletions; F, insertions), including comparison between hg38 and CHM13-T2T reference genomes for the NA12878 sample.

**Supplementary Table S9**. Annotations of deletions detected in whole-genome datasets for the NA12878 and SKBR3 cell lines and P3 and S48 diagnostic samples by SRS and LRS technologies employing (A) LoReC using dbVar_common and ClinVar and (B) AnnotSV, both for the CHM13-T2T reference.

**Supplementary Table S10**. Comparison of functionalities between toolkits for analyzing structural variants.

**Supplementary Fig. S1**. Distribution of insertions (left) and deletions (right) in whole-genome datasets for NA12878 and SKBR3 cell lines and diagnostic tissue samples P3 and detected by various technologies using hg38 and T2T-CHM13 references. LRS-ICLR, synthetic long-read sequencing by Illumina—complete long-read technology on Illumina platform; LRS-ONT, true long-read sequencing by Oxford Nanopore Technologies; LRS-PacBio, true long-read sequencing by Pacific Biosciences; LRS-TELL-Seq, synthetic long-read sequencing by Universal Sequencing Technology on the Illumina platform; LRS-10×, synthetic long-read sequencing by 10× Genomics on the Illumina platform; OGM, optical genome mapping by Bionano Genomics; SRS, short-read sequencing by the Illumina platform.

**Supplementary Fig. S2**. Coverage plots generated by the LoReC toolkit coupled with the Samplot software tool. Bar above figures represents structural variant (SV) detected by optical genome mapping (OGM) technology using the hg38 reference genome compared to the corresponding SV detected by long-read sequencing (LRS platforms) and short-read sequencing (SRS). ICLR, synthetic long-read sequencing by Illumina—complete long-read technology on the Illumina platform; OGM, optical genome mapping by Bionano Genomics; PacBio, true long-read sequencing by Pacific Biosciences; ONT, true long-read sequencing by Oxford Nanopore Technologies; SRS, short-read sequencing by Illumina platform; Tell-Seq, synthetic long-read sequencing by Universal Sequencing Technology on the Illumina platform; 10×, synthetic long-read sequencing by 10× Genomics on the Illumina platform.

**Supplementary Fig. S3**. Counts of low-coverage genes detected by different technologies using the hg38 reference genome and different read mapping quality measures (MAPQ0, MAPQ1, MAPQ50) for NA12878 cell line datasets among (A) genes and pseudogenes, (B) protein-coding genes, and (C) medically relevant genes. MAPQ0 is a default setting in the current SRS/LRS aligners and variant callers, allowing read mapping to multiple regions. MAPQ1 is associated with a lower probability of misplaced reads and MAPQ50 with a very low probability of misplaced genes (99.999% accuracy). The coverage of all genes in the datasets using different MAPQ is shown in Supplementary Table S7. LRS-ICLR, synthetic long-read sequencing by Illumina—complete long-read technology on the Illumina platform; LRS-ONT, true long-read sequencing by Oxford Nanopore Technologies; LRS-PacBio, true long-read sequencing by Pacific Biosciences; LRS-TELL-Seq, synthetic long-read sequencing by Universal Sequencing Technology on the Illumina platform; LRS-10×, synthetic long-read sequencing by 10× Genomics on the Illumina platform; OGM, optical genome mapping by Bionano Genomics; SRS, short-read sequencing by Illumina platform.

**Supplementary Fig. S4**. Overview of structural variants (SVs) detected by optical genome mapping (OGM) for the SKBR3 cell line before and after filtering using the OGM healthy database with hg38 and T2T-CHM13 references. The circos plots were generated using Bionano Access software (v1.8). Circos plots showed the SVs detected by OGM using (A) hg38, (B) T2T-CHM13, and those SVs present in (C) only in unique regions for T2T-CHM13 before and after filtering using the OGM healthy database. (D) Table represents counts of SVs and their types for NA12878, SKBR3, P3, and S48 samples before and after filtering using the OGM healthy database with hg38 and T2T-CHM13 references. LRS-ICLR, synthetic long-read sequencing by Illumina—complete long-read technology on the Illumina platform; LRS-ONT, true long-read sequencing by Oxford Nanopore Technologies; LRS-PacBio, true long-read sequencing by Pacific Biosciences; LRS-TELL-Seq, synthetic long-read sequencing by Universal Sequencing Technology on the Illumina platform; LRS-10×, synthetic long-read sequencing by 10× Genomics on the Illumina platform; OGM, optical genome mapping by Bionano Genomics; SRS, short-read sequencing by the Illumina platform.

**Supplementary Fig. S5**. Coverage profiles for *DUX4, LPA*, and *GRK1* genes detected by short-read and long-read technologies using hg38 and T2T-CHM13 references and different mapping quality thresholds (MAPQ0 and MAPQ1). This figure was generated using Integrative Genomics Viewer (IGV) with corresponding gene coordinates for *DUX4, LPA*, and *GRK1* for both reference genomes. LRS-ONT, true long-read sequencing; LRS-PacBio, true long-read sequencing by Pacific Biosciences; SRS, short-read sequencing by the Illumina platform.

**Supplementary Fig. S6**. Coverage profiles for *SMN1* and *SMN2* genes detected by short-read and long-read technologies using hg38 and T2T-CHM13 references and different mapping quality thresholds (MAPQ0 and MAPQ1). This figure was generated using Integrative Genomics Viewer (IGV) with corresponding gene coordinates for *SMN1* and *SMN2* for both reference genomes. LRS-ONT, true long-read sequencing; LRS-PacBio, true long-read sequencing by Pacific Biosciences; SRS, short-read sequencing by the Illumina platform.

## Abbreviations

GDA: gene–disease association; GIAB: Genome in a Bottle Consortium; HiFi: high-fidelity; INDEL: insertion and deletion; LoReC: LongReadChecker; LRS: long-read sequencing; NGS: next-generation sequencing; OGM: optical genome mapping; SRS: short-read sequencing; SV: structural variant; VDA: variant–disease association; VUS: variant of uncertain significance.

## Supplementary Material

giag027_Supplemental_Files

giag027_Authors_Response_To_Reviewer_Comments_original_submission

giag027_GIGA-D-25-00250_original_submission

giag027_GIGA-D-25-00250_Revision_1

giag027_Reviewer_1_Report_original_submission Reviewer 1 -- 8/11/2025

giag027_Reviewer_1_Report_revision_1 Reviewer 1 -- 8/11/2025

giag027_Reviewer_2_Report_original_submission Reviewer 2 -- 9/26/2025

## Data Availability

All additional supporting data are available in the *GigaScience* repository, GigaDB [31]
