# Supplementary material for: Multiplatform comparisons and annotation of structural variants highlight the utility of the T2T reference genome in human diagnostics
Source: Gigascience. 2026 Mar 9;15:giag027. doi: 10.1093/gigascience/giag027 (PMC13137335; doi:10.1093/gigascience/giag027)
Supplement: giag027_Supplemental_Files [file giag027_supplemental_files.zip › Supplementary Fig 1.pdf]

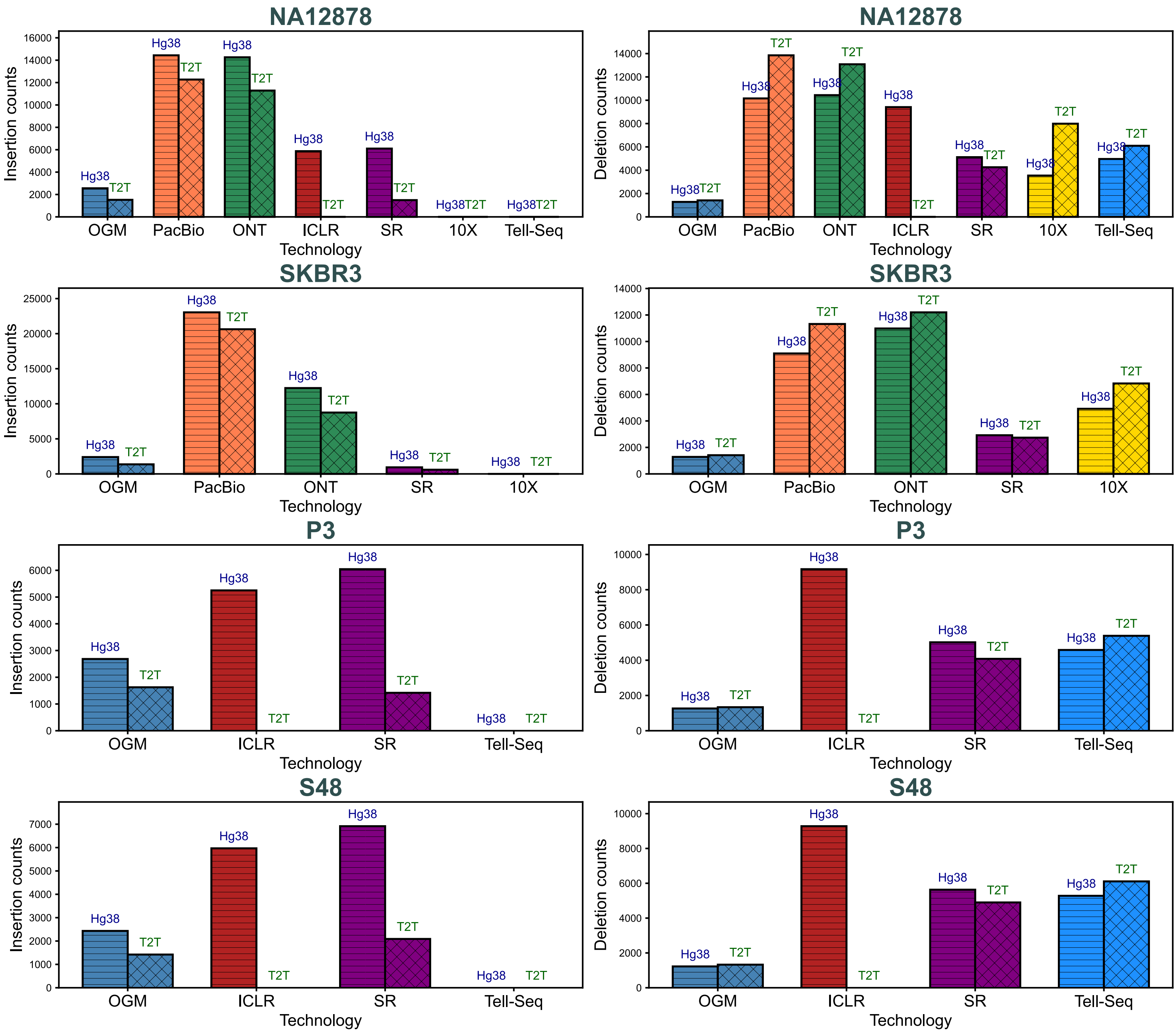

Supplementary Fig. 1. Distribution of insertions (left part) and deletions (right part) in whole-genome datasets for NA12878 and SKBR3 cell lines and diagnostic tissue samples P3 and P48 detected by various technologies using hg38 and T2T-CHM13 references.

Legend: SRS, short-read sequencing by Illumina platform; LRS-PacBio, true long-read sequencing by Pacific Biosciences; LRS-ONT, true long-read sequencing by Oxford Nanopore Technologies; LRS-ICLR, synthetic long-read sequencing by Illumina - complete long-reads technology on Illumina platform; LRS-TELL-Seq, synthetic long-read sequencing by Universal Sequencing Technology on Illumina platform; LRS-10x, synthetic long-read sequencing by 10x Genomics on Illumina platform; OGM, optical genome mapping by Bionano Genomics.
