# Supplementary material for: Multiplatform comparisons and annotation of structural variants highlight the utility of the T2T reference genome in human diagnostics
Source: Gigascience. 2026 Mar 9;15:giag027. doi: 10.1093/gigascience/giag027 (PMC13137335; doi:10.1093/gigascience/giag027)
Supplement: giag027_Supplemental_Files [file giag027_supplemental_files.zip › Supplementary Fig 2.pdf]

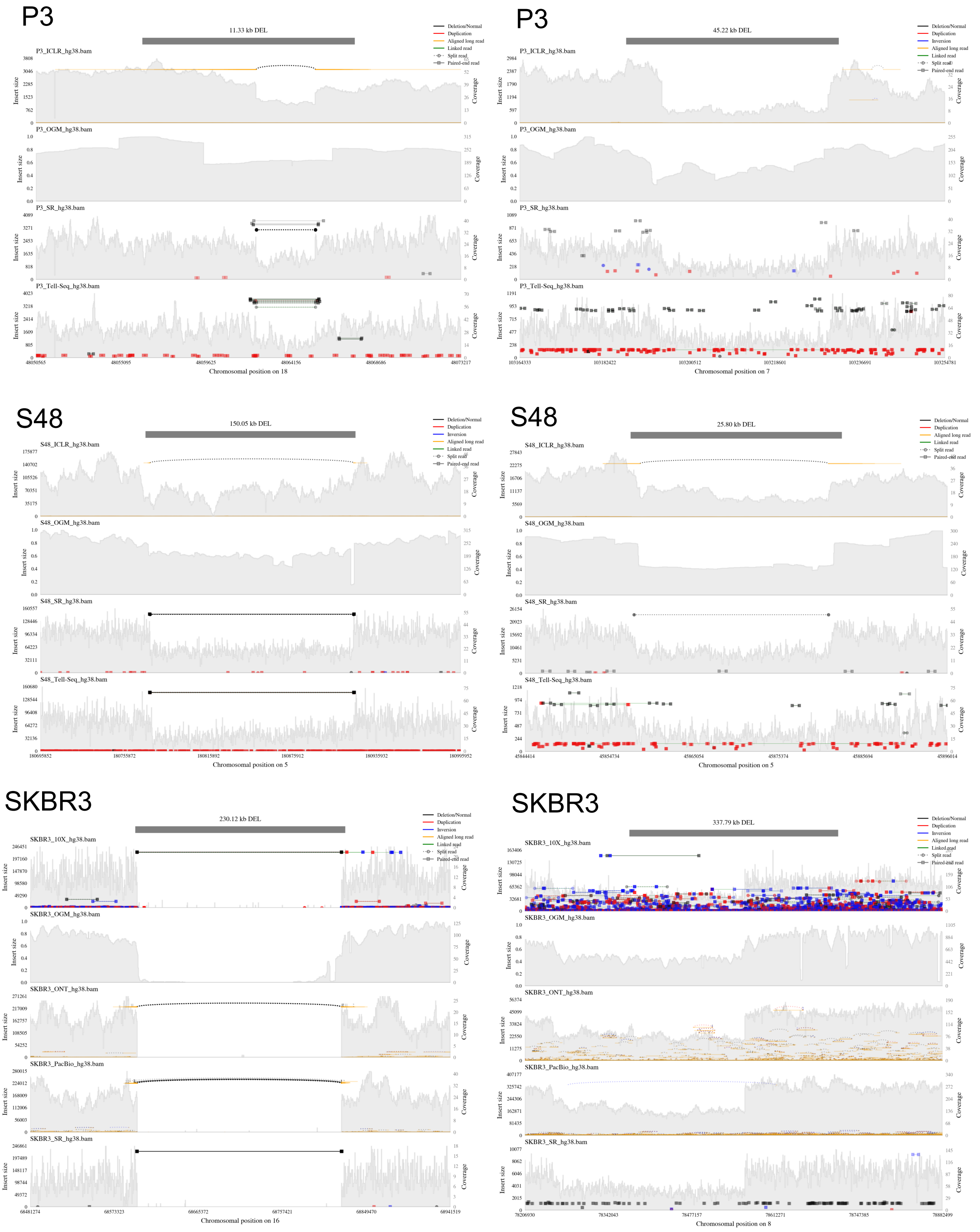

Supplementary Fig. 2. Coverage plots generated by LoReC toolkit coupled with Samplot software tool. Bar above figures represents structural variant (SV) detected by optical genome mapping (OGM) technology using hg38 reference genome compared to the corresponding SV detected by long-read sequencing (LRS platforms) and short-read sequencing (SRS).

Legend: SRS, short-read sequencing by Illumina platform; PacBio, true long-read sequencing by Pacific Biosciences; ONT, true long-read sequencing by Oxford Nanopore Technologies; ICLR, synthetic long-read sequencing by Illumina - complete long-reads technology on Illumina platform; Tell-Seq, synthetic long-read sequencing by Universal Sequencing Technology on Illumina platform; 10X, synthetic long-read sequencing by 10x Genomics on Illumina platform; OGM, optical genome mapping by Bionano Genomics.
