# Supplementary material for: Multiplatform comparisons and annotation of structural variants highlight the utility of the T2T reference genome in human diagnostics
Source: Gigascience. 2026 Mar 9;15:giag027. doi: 10.1093/gigascience/giag027 (PMC13137335; doi:10.1093/gigascience/giag027)
Supplement: giag027_Supplemental_Files [file giag027_supplemental_files.zip › Supplementary Fig 3.pdf]

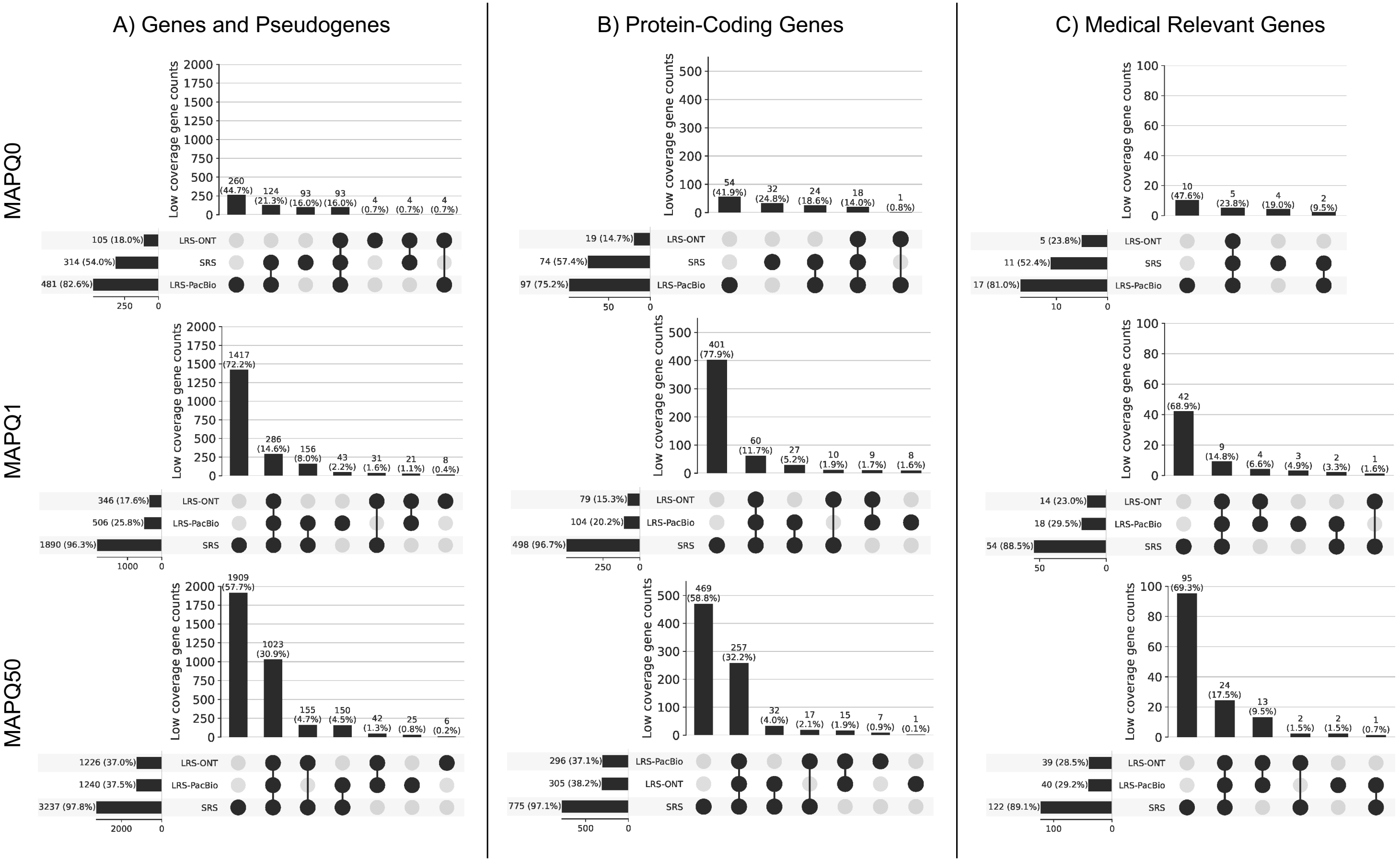

Supplementary Fig. 3. Counts of low coverage genes detected by different technologies using hg38 reference genome and different read mapping quality measures (MAPQ0, MAPQ1, MAPQ50) for NA12878 cell line datasets among: A) genes and pseudogenes, B) protein-coding genes, and C) medical relevant genes.

MAPQ0 is a default setting in the current SRS/LRS aligners and variant callers allowing read mapping to multiple regions. MAPQ1 is associated with lower probability of misplaced reads, MAPQ50 with a very low probability of misplaces genes (99.999% accuracy). The coverages of all genes in the datasets using different MAPQ are shown in Supplementary Table 7.

Legend: SRS, short-read sequencing by Illumina platform; LRS-PacBio, true long-read sequencing by Pacific Biosciences; LRS-ONT, true long-read sequencing by Oxford Nanopore Technologies; LRS-ICLR, synthetic long-read sequencing by Illumina - complete long-reads technology on Illumina platform; LRS-TELL-Seq, synthetic long-read sequencing by Universal Sequencing Technology on Illumina platform; LRS-10x, synthetic long read sequencing by 10x Genomics on Illumina platform; OGM, optical genome mapping by Bionano Genomics.
