# Supplementary material for: Multiplatform comparisons and annotation of structural variants highlight the utility of the T2T reference genome in human diagnostics
Source: Gigascience. 2026 Mar 9;15:giag027. doi: 10.1093/gigascience/giag027 (PMC13137335; doi:10.1093/gigascience/giag027)
Supplement: giag027_Supplemental_Files [file giag027_supplemental_files.zip › Supplementary Fig 4.pdf]

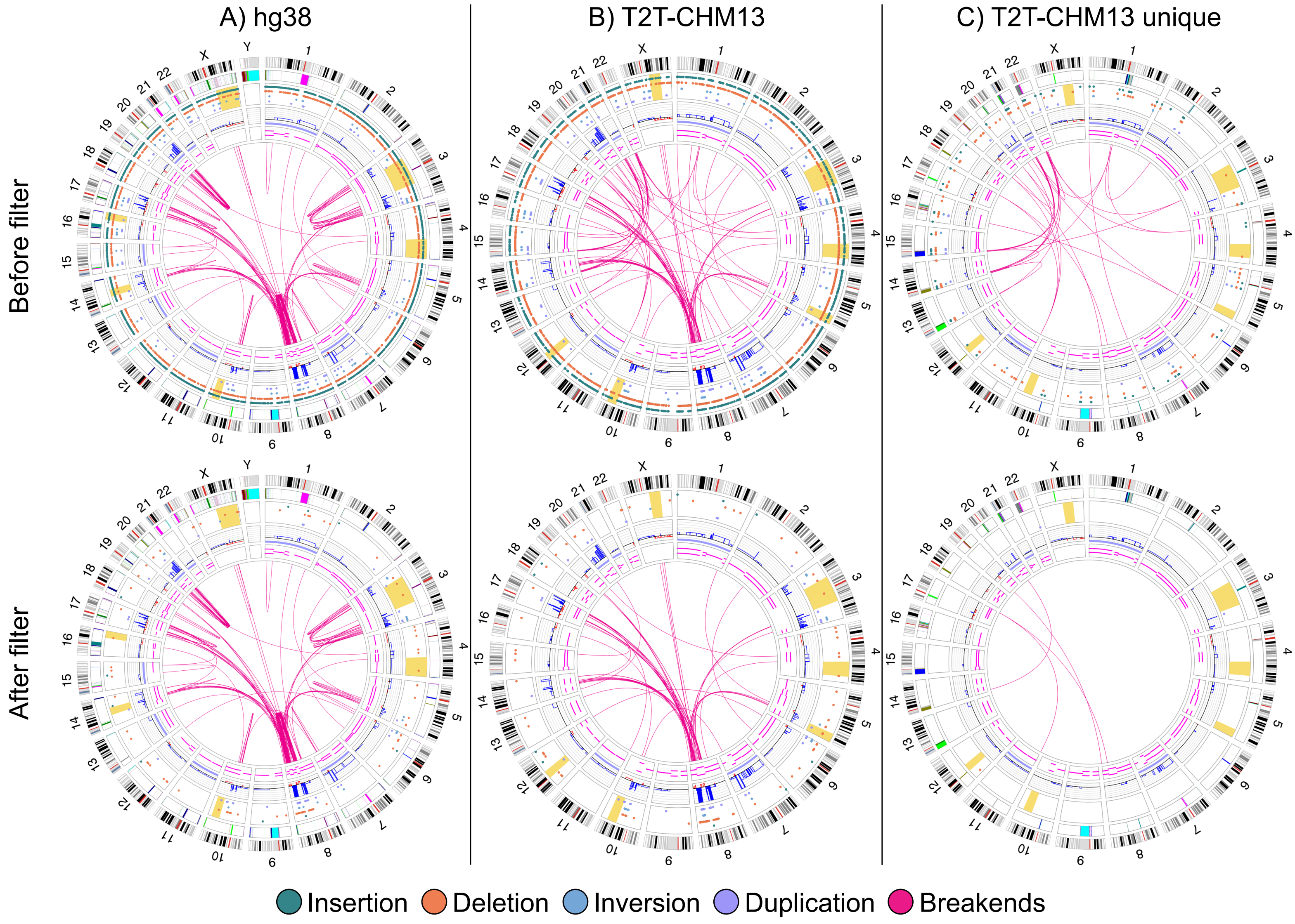

D) OGM SV counts before and after healthy database filter

Reference	hg38				T2T-CHM13			
	before/after filtering OGM healthy database				before/after filtering OGM healthy database			
**Sample**	**NA12878**	**SKBR3**	**P3**	**S48**	**NA12878**	**SKBR3**	**P3**	**S48**
**Insertions (INS)**	2556 / 3	2403 / 7	2638 / 16	2432 / 10	1523 / 6	1365 / 10	1625 / 15	1421 / 10
**Deletions (DEL)**	1283 / 3	1283 / 87	1262 / 19	1225 / 40	1414 / 3	1413 / 90	1335 / 27	1326 / 40
**Duplications (DUP)**	44 / 0	64 / 19	84 / 23	55 / 6	38 / 0	60 / 20	64 / 18	46 / 6
**Inversions (INV)**	64 / 0	98 / 28	71 / 4	72 / 0	42 / 0	66 / 24	34 / 1	44 / 0
**breakends (BND)**	0 / 0	182 / 182	21 / 21	12 / 11	65 / 5	103 / 57	72 / 16	40 / 10

Supplementary Fig. 4. Overview of structural variants (SVs) detected by optical genome mapping (OGM) for SKBR3 cell line before and after filtering using OGM healthy database using hg38 and T2T-CHM13 references. The circos plots were generated using Bionano Access software (v1.8).
Circos plots showed the SVs detected by OGM using A) hg38, B) T2T-CHM13 and those SVs present in C) only in unique regions for T2T-CHM13 before and after filtering using the OGM healthy database. D) Table represents counts of SVs and their types for NA12878, SKBR3, P3, S48 samples before and after filtering using the OGM healthy database using hg38 and T2T-CHM13 references.
Legend: SRS, short-read sequencing by Illumina platform; LRS-PacBio, true long read sequencing by Pacific Biosciences; LRS-ONT, true long read sequencing by Oxford Nanopore Technologies; LRS-ICLR, synthetic long read sequencing by Illumina - complete long reads technology on Illumina platform; LRS-TELL-Seq, synthetic long read sequencing by Universal Sequencing Technology on Illumina platform; LRS-10x, synthetic long read sequencing by 10x Genomics on Illumina platform; OGM, optical genome mapping by Bionano Genomics.
