# Supplementary material for: Multiplatform comparisons and annotation of structural variants highlight the utility of the T2T reference genome in human diagnostics
Source: Gigascience. 2026 Mar 9;15:giag027. doi: 10.1093/gigascience/giag027 (PMC13137335; doi:10.1093/gigascience/giag027)
Supplement: giag027_Supplemental_Files [file giag027_supplemental_files.zip › Supplementary Table 1.pdf]

Supplementary Table 1 Whole-genome datasets from short-read sequencing, long-read sequencing and optical mapping analysed in this study.

	SRS	LRS-PacBio	LRS-ONT	LRS-ICLR	LRS-TELL-Seq	LRS-10x	OGM
**NA12878 cell line**	This study	ftp://ftp-trace.ncbi.nlm.nih.gov/giab/ftp/data/NA12878 (HiFi reads)[14]	ftp://ftp-trace.ncbi.nlm.nih.gov/giab/ftp/data/NA12878[14]	This study	This study	ftp://ftp-trace.ncbi.nlm.nih.gov/giab/ftp/data/NA12878[14]	This study
**SKBR3 cell line**	https://schatz-lab.org/publications/SKBR3/[15]	https://schatz-lab.org/publications/SKBR3/ (CLR reads) [15]	https://schatz-lab.org/publications/SKBR3/[15]	NA	NA	https://schatz-lab.org/publications/SKBR3/[15]	https://bionanogenomics.com/library/datasets/
**P3 (pheochromocytoma tumor tissue)**	This study	NA	NA	This study	This study	NA	This study
**MM48 (multiple myeloma cells from bone marrow)**	This study	NA	NA	This study	This study	NA	This study

Legend: SRS, short-read sequencing by Illumina platform; LRS-PacBio, true long-read sequencing by Pacific Biosciences; LRS-ONT, true long-read sequencing by Oxford Nanopore Technologies; LRS-ICLR, synthetic long-read sequencing by Illumina - complete long-reads technology on Illumina platform; LRS-TELL-Seq, synthetic long-read sequencing by Universal Sequencing Technology on Illumina platform; LRS-10x, synthetic long-read sequencing by 10x Genomics on Illumina platform; OGM, optical genome mapping by Bionano Genomics. NA, not available.
