# Supplementary material for: Multiplatform comparisons and annotation of structural variants highlight the utility of the T2T reference genome in human diagnostics
Source: Gigascience. 2026 Mar 9;15:giag027. doi: 10.1093/gigascience/giag027 (PMC13137335; doi:10.1093/gigascience/giag027)
Supplement: giag027_Supplemental_Files [file giag027_supplemental_files.zip › Supplementary Table 10.pdf]

Supplementary Table 10 Comparison of functionalities between toolkits for analyzing structural variants

**Toolkits**	**LoReC (v1.0)**		**Jasmine (v1.1.5)**	**SURVIVOR**	
**Feature**	**LoReC-comparator**	**LoReC-coverage**		**SURVIVOR**	**SURVIVOR_ant**
Github link	https://github.com/novosadt/lorec-comparator	https://github.com/novosadt/lorec-comparator	https://github.com/mkirsche/Jasmine	https://github.com/fritzsedlazeck/SURVIVOR	https://github.com/fritzsedlazeck/SURVIVOR_ant
Description	Long read, high-throughput sequencing and optical mapping structural variant Comparator	Optical mapping, high-throughput and long read coverage comparison and visualization tool	SV Merging Across Samples	Toolset for SV simulation, comparison and filtering	A framework to annotate SVs with previous known SVs (vcf file) and or with genomic features (gff and or bed files)
Last update	November 2025	November 2025	April 2022	February 2019	March 2018
Input formats	VCF, AnnotSV, SMAP, BED, ClinVar, dbVar	VCF, BAM	VCF list	VCF list	VCF, GFF, BED
Output formats	Comparison tables (CSV) and statistics (csv)	Coverage reports and statistics (CSV), plots (PDF, PNG)	Merged VCF	Merged VCF	Annotated VCF
Annotation capability	✔	✔	✗	✗	✔
Region filtering	✔	✔	✗	✔	✔
Multi-sample support	✔	✔	✔	✔	✗
Configurable distance threshold	✔	✗	✔	✔	✔
Intersection factor & size proportion	✔	✗	✗	✗	✗
Filtering on overlap genes	✔	✔	✗	✗	✗
Filtering on MAPQ	✗	✔	✗	✗	✗
Coverage analysis	✗	✔	✗	✗	✗
Plot generation	✗	✔	✗	✗	✗
SV type filtering	✔	✗	✗	✗	✗
