# Supplementary material for: Multiplatform comparisons and annotation of structural variants highlight the utility of the T2T reference genome in human diagnostics
Source: Gigascience. 2026 Mar 9;15:giag027. doi: 10.1093/gigascience/giag027 (PMC13137335; doi:10.1093/gigascience/giag027)
Supplement: giag027_Supplemental_Files [file giag027_supplemental_files.zip › Supplementary Table 2.pdf]

Supplementary Table 2 Structural variants detected in the NA12878 and SKBR3 cell lines and the P3 and S48 diagnostic samples using SRS and different LRS technologies for the hg38 and T2T-CHM13 human references.

Sample	SV type	Total number of SVs		Deletions		Insertions		Duplications		Inversions		Breakends/ Translocations	
	References/ Technology	hg38	T2T-CHM13	hg38	T2T-CHM13	hg38	T2T-CHM13	hg38	T2T-CHM13	hg38	T2T-CHM13	hg38	T2T-CHM13
**NA12878**	SRS	12,912	6,769	5,120	4,255	6,103	1,498	48	435	261	220	1421	361
	LRS-PacBio	24,811	26,397	10,157	13,852	14,435	12,270	102	88	94	54	312	133
	LRS-ONT	24,793	24,516	10,439	13,085	14,259	11,285	55	22	65	46	123	78
	LRS-ICLR	15,410	NA	9,412	NA	5,872	NA	28	NA	80	NA	38	NA
	LRS-10x	11,800	15,120	3,542	7,989	ND	ND	849	780	6,623	5,867	786	484
	LRS-TELL-Seq	8,543	10,873	4,964	6,096	ND	ND	54	162	3432	4,514	93	101
	OGM	3,947	3,082	1,283	1,414	2,556	1,523	44	38	64	42	0	65
**SKBR3**	SRS*	5,324	4,011	2,920	2,738	937	598	937	238	258	212	272	225
	LRS-PacBio	32,850	32,533	9,097	11,321	22,994	20,630	154	120	235	207	370	255
	LRS-ONT	23,597	21,291	10,983	12,200	12,221	8,749	49	39	170	152	174	151
	LRS-10x	10,319	12,140	4,918	3,393	ND	ND	1,318	6,830	3,393	1,300	690	617
	OGM	4,030	3,007	1,283	1,413	2,403	1,365	64	60	98	66	182	103
**P3**	SRS	12,823	6,441	5,018	4,079	5993	1418	54	444	246	196	1512	304
	LRS-ICLR	14,513	NA	9,166	NA	5228	NA	28	NA	60	NA	31	NA
	LRS-TELL-Seq	8,434	9,171	4,582	5,385	ND	ND	193	153	3,553	3,524	106	109
	OGM	4,076	3,130	1,262	1,335	2,638	1,625	84	64	71	34	21	72
**S48**	SRS	14,846	8,386	5,632	4,900	6,862	2,089	72	554	284	256	1,996	587
	LRS-ICLR	15,342	NA	9279	NA	5951	NA	24	NA	62	NA	26	NA
	LRS-TELL-Seq	9,960	10,888	5,281	6,109	ND	ND	153	164	4,436	4,514	90	101
	OGM	3,796	2,877	1,225	1,326	2,432	1,421	55	44	72	46	12	40

Legend: SRS, short-read sequencing by Illumina platform; LRS-PacBio, true long-read sequencing by Pacific Biosciences; LRS-ONT, true long-read sequencing by Oxford Nanopore Technologies; LRS-ICLR, synthetic long-read sequencing by Illumina - complete long-reads technology on Illumina platform; LRS-TELL-Seq, synthetic long-read sequencing by Universal Sequencing Technology on Illumina platform; LRS-10x, synthetic long-read sequencing by 10x Genomics on Illumina platform; SVs, structural variants; OGM, optical genome mapping by Bionano Genomics; NA, not available; ND, not detected. *dataset with average coverage 20× (less than in other datasets, where 30× coverage was achieved).
