# Supplementary material for: Multiplatform comparisons and annotation of structural variants highlight the utility of the T2T reference genome in human diagnostics
Source: Gigascience. 2026 Mar 9;15:giag027. doi: 10.1093/gigascience/giag027 (PMC13137335; doi:10.1093/gigascience/giag027)
Supplement: giag027_Supplemental_Files [file giag027_supplemental_files.zip › Supplementary Table 3.pdf]

Supplementary Table 3 Comparison of deletions and insertions larger/smaller than 0.5 kbp in the whole-genome datasets detected by current long-read (LRS) and short-read sequencing (SRS) technologies and optical genome mapping (OGM). The number (in bracket the percentage) present the SVs that overlap of the SVs detected by base technology A) LRS-ONT, B) SRS and C) OGM detected by other technologies using hg38 and T2T-CHM13 references.

A) LRS-ONT as a base for comparison with other technologies

Sample	Reference	SV type	LRS-ONT (base)	OGM	LRS-PacBio	LRS-10x	LRS-TELL-Seq	LRS-ICLR	SRS	LRS-ICLR+SRS
NA12878	hg38	DEL < 0.5 kbp	8,434	41 (0%)	7,505 (89%)	2,153 (26%)	2,682 (32%)	5,983 (71%)	3,694 (44%)	6,239 (74%)
	hg38	DEL ≥ 0.5 kbp	2,005	1,020 (51%)	1,663 (83%)	985 (49%)	751 (37%)	1,099 (55%)	961 (48%)	1,214 (61%)
	hg38	INS <0.5 kbp	10,619	135 (1%)	9,979 (94%)	ND	ND	4,585 (43%)	4,204 (40%)	5,844 (55%)
	hg38	INS ≥ 0.5 kbp	3,640	2,231 (61%)	3,213 (88%)	ND	ND	458 (13%)	551 (15%)	880 (24%)
	T2T-CHM13	DEL < 0.5 kbp	9,979	36 (0%)	8,647 (87%)	2,620 (26%)	1,803 (18%)	NA	2,982 (30%)	NA
	T2T-CHM13	DEL ≥ 0.5 kbp	3,106	1,141 (37%)	2,418 (78%)	1,081 (35%)	525 (17%)	NA	846 (27%)	NA
	T2T-CHM13	INS <0.5 kbp	9,020	59 (1%)	8,220 (91%)	ND	ND	NA	1,197 (13%)	NA
	T2T-CHM13	INS ≥ 0.5 kbp	2,265	1,095 (48%)	1,766 (78%)	ND	ND	NA	58 (3%)	NA
SKBR3	hg38	DEL < 0.5 kbp	9,358	28 (0%)	6,820 (73%)	2,677 (29%)	NA	NA	1,851 (20%)	NA
	hg38	DEL ≥ 0.5 kbp	1,625	960 (59%)	1,399 (86%)	858 (53%)	NA	NA	814 (50%)	NA
	hg38	INS <0.5 kbp	9,304	131 (1%)	8,766 (94%)	ND	NA	NA	767 (8%)	NA
	hg38	INS ≥ 0.5 kbp	2,938	1,854 (63%)	2,654 (90%)	ND	NA	NA	57 (2%)	NA
	T2T-CHM13	DEL < 0.5 kbp	10,265	39 (0%)	7,401 (72%)	2,599 (25%)	NA	NA	1,743 (17%)	NA
	T2T-CHM13	DEL ≥ 0.5 kbp	1,935	988 (51%)	1,628 (84%)	851 (44%)	NA	NA	770 (40%)	NA
	T2T-CHM13	INS <0.5 kbp	7,216	42 (1%)	6,720 (93%)	ND	NA	NA	461 (6%)	NA
	T2T-CHM13	INS ≥ 0.5 kbp	1,553	879 (57%)	1,270 (82%)	ND	NA	NA	18 (1%)	NA

B) SRS as a base for comparison with other technologies									
**Sample**	**Reference**	**SV type**	**SRS (base)**	**OGM**	**LRS-PacBio**	**LRS-ONT**	**LRS-10x**	**LRS-TELL-Seq**	**LRS-ICLR**
**NA12878**	hg38	DEL < 0.5 kbp	4,148	15 (0%)	3,658 (88%)	3,645 (88%)	1,763 (43%)	2,225 (54%)	3,527 (85%)
	hg38	DEL ≥ 0.5 kbp	972	731 (75%)	923 (95%)	925 (95%)	844 (87%)	653 (67%)	821 (84%)
	hg38	INS <0.5 kbp	5,978	41 (1%)	4,785 (80%)	4,735 (79%)	ND	ND	3,255 (54%)
	hg38	INS ≥ 0.5 kbp	125	43 (34%)	120 (96%)	120 (96%)	ND	ND	45 (36%)
	T2T-CHM13	DEL < 0.5 kbp	3,351	10 (0%)	2,994 (89%)	2,943 (88%)	1,715 (51%)	1,219 (36%)	ND
	T2T-CHM13	DEL ≥ 0.5 kbp	904	613 (68%)	796 (88%)	791 (88%)	740 (82%)	355 (39%)	ND
	T2T-CHM13	INS <0.5 kbp	1,492	6 (0%)	1,270 (85%)	1,240 (83%)	ND	ND	NA
	T2T-CHM13	INS ≥ 0.5 kbp	6	3 (50%)	6 (100%)	6 (100%)	ND	ND	NA
**SKBR3**	hg38	DEL < 0.5 kbp	1,991	5 (0%)	1,800 (90%)	1,808 (91%)	1,465 (74%)	NA	NA
	hg38	DEL ≥ 0.5 kbp	929	666 (72%)	772 (83%)	788 (85%)	685 (74%)	NA	NA
	hg38	INS <0.5 kbp	937	5 (1%)	822 (88%)	805 (86%)	ND	NA	NA
	hg38	INS ≥ 0.5 kbp	1	1 (100%)	1 (100%)	1 (100%)	ND	NA	NA
	T2T-CHM13	DEL < 0.5 kbp	1,890	5 (0%)	1,699 (90%)	1,700 (90%)	1,333 (71%)	NA	NA
	T2T-CHM13	DEL ≥ 0.5 kbp	848	615 (73%)	741 (87%)	743 (88%)	647 (76%)	NA	NA
	T2T-CHM13	INS <0.5 kbp	598	1 (0%)	506 (85%)	479 (80%)	ND	NA	NA
	T2T-CHM13	INS ≥ 0.5 kbp	0	0 (0%)	0 (0%)	0 (0%)	ND	NA	NA

C) OGM as a base for comparison with other technologies

Sample	Reference	SV type	OGM (base)	LRS-ONT	LRS-PacBio	LRS-10X	LRS-Tell-Seq	LRS-ICLR	SRS	ICLR+SR
NA12878	hg38	DEL	1,283	1,028 (80%)	1,001 (78%)	828 (65%)	643 (50%)	787 (61%)	787 (61%)	873 (68%)
	hg38	INS	2,556	2,118 (83%)	2,041 (80%)	ND	ND	322 (13%)	880 (34%)	942 (37%)
	T2T-CHM13	DEL	1,414	1,082 (77%)	1,085 (77%)	805 (57%)	373 (26%)	NA	639 (45%)	NA
	T2T-CHM13	INS	1,523	1,100 (72%)	1,029 (68%)	ND	NA	NA	179 (12%)	NA
SKBR3	hg38	DEL	1,283	991 (77%)	945 (74%)	766 (60%)	NA	NA	707 (55%)	NA
	hg38	INS	2,403	1,889 (79%)	1,934 (80%)	ND	NA	NA	119 (5%)	NA
	T2T-CHM13	DEL	1,413	1,017 (72%)	992 (70%)	737 (52%)	NA	NA	647 (46%)	NA
	T2T-CHM13	INS	1,365	896 (66%)	920 (67%)	ND	NA	NA	84 (6%)	NA

Legend: SRS, short-read sequencing by Illumina platform; LRS-PacBio, true long-read sequencing by Pacific Biosciences; LRS-ONT, true long-read sequencing by Oxford Nanopore Technologies; LRS-ICLR, synthetic long-read sequencing by Illumina - complete long-reads technology on Illumina platform; LRS-TELL-Seq, synthetic long-read sequencing by Universal Sequencing Technology on Illumina platform; LRS-10x, synthetic long-read sequencing by 10x Genomics on Illumina platform; OGM, optical genome mapping by Bionano Genomics. NA, not available.
