# Supplementary material for: Multiplatform comparisons and annotation of structural variants highlight the utility of the T2T reference genome in human diagnostics
Source: Gigascience. 2026 Mar 9;15:giag027. doi: 10.1093/gigascience/giag027 (PMC13137335; doi:10.1093/gigascience/giag027)
Supplement: giag027_Supplemental_Files [file giag027_supplemental_files.zip › Supplementary Table 9.pdf]

Supplementary Table 9 Annotations of deletions detected in whole-genome datasets for the NA12878 and SKBR3 cell lines and P3 and S48 diagnostic samples by SRS and LRS technologies employing A) LoReC using dbVar_common and ClinVar and B) AnnotSV, both for the CHM13-T2T reference.

ALL		A) LoReC							B) AnnotSV			
Sample	Technology	TOTAL DEL	dbVar_common	ClinVar	Pathogenic / Likely Pathogenic	VUS	Benign/ Likely Benign	Not annotated	TOTAL DEL+BND	Benign	Pathogenic /Likely Pathogenic	VUS (Not annotated)
NA12878	SRS	4,255	1,521 (36%)	455 (11%)	42/10	80	293/18	2,604 (61%)	4,224	3,008 (71%)	12/2	1,202 (29%)
	LRS-PacBio	13,852	2,680 (19%)	987 (7%)	69/22	130	704/45	10,755 (78%)	13,852	5,641 (41%)	7/12	8,192 (59%)
	LRS-ONT	13,085	2,661 (20%)	987 (8%)	67/22	139	696/44	10,015 (77%)	13,085	5,370 (41%)	5/12	7,698 (59%)
SKBR3	SRS	2,738	1,153 (42%)	453 (17%)	65/14	102	242/21	1,426 (52%)	2,721	1,969 (72%)	48/11	693 (49%)
	LRS-PacBio	11,321	2,344 (21%)	826 (7%)	83/16	132	538/41	8,611 (76%)	11,321	4,802 (42%)	35/14	6,470 (57%)
	LRS-ONT	12,220	2,473 (20%)	867 (7%)	80/18	134	578/34	9,356 (77%)	12,200	5,428 (45%)	35/18	6,719 (55%)
P3	SRS	4,079	1,469 (36%)	450 (11%)	39/15	93	269/20	2,473 (61%)	4,046	2,907 (72%)	10/10	1,119 (28%)
S48	SRS	4,900	1,693 (35%)	510 (11%)	54/15	102	295/30	3,045 (62%)	4,869	3,386 (70%)	18/5	1,460 (30%)

Legend: SRS, short-read sequencing by Illumina platform; LRS-PacBio, true long-read sequencing by Pacific Biosciences; LRS-ONT, true long-read sequencing by Oxford Nanopore Technologies; LRS-ICLR, synthetic long-read sequencing by Illumina - complete long-reads technology on Illumina platform; LRS-TELL-Seq, synthetic long-read sequencing by Universal Sequencing Technology on Illumina platform; LRS-10x, synthetic long-read sequencing by 10x Genomics on Illumina platform; OGM, optical genome mapping by Bionano Genomics; VUS, variant of unknown significance; DEL, deletion; BND, Breakends.
