# Supplementary material for: Multiplatform comparisons and annotation of structural variants highlight the utility of the T2T reference genome in human diagnostics
Source: Gigascience. 2026 Mar 9;15:giag027. doi: 10.1093/gigascience/giag027 (PMC13137335; doi:10.1093/gigascience/giag027)
Supplement: giag027_GIGA-D-25-00250_original_submission [file giag027_giga-d-25-00250_original_submission.pdf]

GigaScience

Multiplatform comparisons and annotation of structural variants highlight the utility of the T2T reference genome in human diagnostics

--Manuscript Draft--

**Manuscript Number:**	GIGA-D-25-00250
**Full Title:**	Multiplatform comparisons and annotation of structural variants highlight the utility of the T2T reference genome in human diagnostics
**Article Type:**	Research
**Funding Information:**	Univerzita Palackého v Olomouci (JG_2025_035) — Prof. Eva Kriegova<br>Univerzita Palackého v Olomouci (IGA_LF_2025_014) — Prof. Eva Kriegova<br>Ministerstvo Zdravotnictví Ceské Republiky (NW24-10-00395) — Prof. Eva Kriegova<br>Ministerstvo Zdravotnictví Ceské Republiky (MH CZ – DRO (FNOL, 00098892)) — Prof. Eva Kriegova
**Abstract:**	Background: Structural variants (SVs) are increasingly recognized as important contributors of human diseases. However, our understanding of SVs in health and disease is limited, mainly due to their structural complexity and variable length between individuals, as well as limitations inherent to the available genomic technologies and used reference genome.<br>Results: To systematically evaluate SVs across human whole-genome samples using hg38/GRCh38 and gapless T2T-CHM13 reference, we introduced an innovative multi-platform approach, LongReadChecker (LoReC), which advances SVs comparison and annotation based on distance variance, intersection, gene overlap and the closest SV in the clinical database. Comparison of the performance in detecting SVs from public and own whole-genome datasets from short-read sequencing (SRS), available long-read sequencing (LRS) platforms, and optical genome mapping (OGM) revealed that most SVs detected by SRS were confirmed by LRS, but LRS can identify twice as many SVs (~25,000 SVs/genome) with higher read mapping accuracy. Our data further highlights the utility of the T2T-CHM13 reference in SV detection, as 20% more deletions and less 20% insertion are detected compared with hg38/GRCh38, particularly evident in long-read datasets. Since 80% of the SVs detected by LRS/SRS are smaller than 0.5 kbp, OGM did not detect them.<br>Conclusions: Our study revealed that introducing of distance variance, intersection, gene overlap and the closest SV in the clinical database may help to compare and annotate SVs in diagnostics. Our data showed that LRS together with T2T-CHM13 gapless sequences can improve the diagnostics of patients with human diseases when SRS fails to identify the cause.
**Corresponding Author:**	Eva Kriegová<br>Palacky University Olomouc: Univerzita Palackeho v Olomouci<br>Olomouc, CZECH REPUBLIC
**Corresponding Author Secondary Information:**	
**Corresponding Author's Institution:**	Palacky University Olomouc: Univerzita Palackeho v Olomouci
**Corresponding Author's Secondary Institution:**	
**First Author:**	Jakub Savara
**First Author Secondary Information:**	
**Order of Authors:**	Jakub Savara<br>Tomas Novosad<br>Petr Gajdos

Powered by Editorial Manager® and ProduXion Manager® from Aries Systems Corporation

	Anna Petrackova
	Marek Behalek
	Jirina Manakova
	Filip Ctvrtlik
	Jiri Minarik
	Tomas Papajik
	Eva Kriegova
**Order of Authors Secondary Information:**	
**Additional Information:**	
**Question**	**Response**
Are you submitting this manuscript to a special series or article collection?	No
**Experimental design and statistics**<br><br>Full details of the experimental design and statistical methods used should be given in the Methods section, as detailed in our Minimum Standards Reporting Checklist. Information essential to interpreting the data presented should be made available in the figure legends.<br><br>Have you included all the information requested in your manuscript?	Yes
**Resources**<br><br>A description of all resources used, including antibodies, cell lines, animals and software tools, with enough information to allow them to be uniquely identified, should be included in the Methods section. Authors are strongly encouraged to cite Research Resource Identifiers (RRIDs) for antibodies, model organisms and tools, where possible.<br><br>Have you included the information requested as detailed in our Minimum Standards Reporting Checklist?	Yes
**Availability of data and materials**	Yes

Powered by Editorial Manager® and ProduXion Manager® from Aries Systems Corporation

All datasets and code on which the conclusions of the paper rely must be either included in your submission or deposited in publicly available repositories (where available and ethically appropriate), referencing such data using a unique identifier in the references and in the "Availability of Data and Materials" section of your manuscript.<br><br>Have you have met the above requirement as detailed in our Minimum Standards Reporting Checklist?	
GigaScience has policies and guidelines in place for the use of generative AI-writing tools such as ChatGPT. If you have used such writing tools to assist with writing the manuscript this must be declared and cited in the text. Authors should not list AI-writing tools and other AI-assisted technologies as an author or co-author and should acknowledge that they are fully responsible for text generated or refined by AI-writing tools.<p><br><br>A summary of use (particularly in the introduction or among methods) needs to be included at the end of the paper, and the outputs should also be included as a supplementary file hosted in GigaDB or other open repositories. Please read our guidelines for more information. <p><br><br>By submitting to GigaScience, you are aware of the journal's AI-writing tools policy, and if you have declared use of such tools below, you have acknowledged this where appropriate in your manuscript and have made a summary of use and outputs available. <p><br><b>AI-assisted writing tools have been used in the preparation of this manuscript?	No

Powered by Editorial Manager® and ProduXion Manager® from Aries Systems Corporation

LaTeX - Main Document (TeX file)
Click here to access/download;LaTeX - Main Document (TeX file);main.tex

Placeholder for OUP logo oup.pdf

(GIGA)^n SCIENCE

GigaScience, 2023, 1–11

doi: xx.xxxx/xxxx
Manuscript in Preparation
Research

RESEARCH

Multiplatform comparisons and annotation of structural variants highlight the utility of the T2T reference genome in human diagnostics

Jakub Savara[1,2], Tomas Novosad[2], Petr Gajdos[2], Anna Petrackova[1], Marek Behalek[2], Jirina Manakova[1], Filip Ctvrtlik[3], Jiri Minarik[4], Tomas Papajik[4] and Eva Kriegova[1]

[1]Department of Immunology, Faculty of Medicine and Dentistry, Palacký University Olomouc and University Hospital Olomouc, Olomouc, Czech Republic and [2]Department of Computer Science, Faculty of Electrical Engineering and Computer Science, VSB-Technical University of Ostrava, Ostrava, Czech Republic and [3]Department of Radiology, Faculty of Medicine and Dentistry, Palacký University Olomouc and University Hospital Olomouc, Olomouc, Czech Republic and [4]Department of Hemato-oncology, Faculty of Medicine and Dentistry, Palacký University Olomouc and University Hospital Olomouc, Olomouc, Czech Republic

Abstract

Background: Structural variants (SVs) are increasingly recognized as important contributors of human diseases. However, our understanding of SVs in health and disease is limited, mainly due to their structural complexity and variable length between individuals, as well as limitations inherent to the available genomic technologies and used reference genome. **Results:** To systematically evaluate SVs across human whole-genome samples using hg38/GRCh38 and gapless T2T-CHM13 reference, we introduced an innovative multi-platform approach, LongReadChecker (LoReC), which advances SVs comparison and annotation based on distance variance, intersection, gene overlap and the closest SV in the clinical database. Comparison of the performance in detecting SVs from public and own whole-genome datasets from short-read sequencing (SRS), available long-read sequencing (LRS) platforms, and optical genome mapping (OGM) revealed that most SVs detected by SRS were confirmed by LRS, but LRS can identify twice as many SVs (25,000 SVs/genome) with higher read mapping accuracy. Our data further highlights the utility of the T2T-CHM13 reference in SV detection, as 20% more deletions and less 20% insertion are detected compared with hg38/GRCh38, particularly evident in long-read datasets. Since 80% of the SVs detected by LRS/SRS are smaller than 0.5 kbp, OGM did not detect them. **Conclusions:** Our study revealed that introducing of distance variance, intersection, gene overlap and the closest SV in the clinical database may help to compare and annotate SVs in diagnostics. Our data showed that LRS together with T2T-CHM13 gapless sequences can improve the diagnostics of patients with human diseases when SRS fails to identify the cause.

Key words: Next-generation sequencing; structural variants; annotations; LongReadChecker (LoReC) toolkit; long-read technology

Introduction

Structural variants (SVs) are a major source of human genetic diversity that arises from the breakdown and rejoining of DNA fragments, which can lead to loss, gain and rearrangement of genes and regulatory elements [1]. Since SVs are larger than 50 bp and can affect thousands to millions of nucleotides [2, 3], they are expected to have a strong effect on transcriptional regulation in health and disease [4]. SV detection is of particular importance, as they are responsible for more than 25% of all rare protein truncations in a

Compiled on: June 26, 2025.
Draft manuscript prepared by the author.

genome and are associated with many diseases [5, 6]. However, SV characterization and functional interrogation have largely lagged behind single nucleotide variations and small INDELs (insertions and deletions), mainly due to their structural complexity and variable length between individuals, as well as limitations inherent to available genomic technologies [7]. Traditionally, SVs in the human genome have been detected using array-based methods or locus-specific assays for targeted regions [8]. Currently, great progress is being made in the detection of SVs by short-read sequencing (SRS), which remains essential due to practical and cost considerations in clinical diagnostics [9]. Novel genomic technologies for SV detection are also being rapidly developed. Among them, long-read sequencing (LRS) has demonstrated a high potential to detect SVs [10] through longer reads and increased accuracy compared to SRS [1]. The increasing throughput, lower prices and portability of LRS technologies increase the potential of the introduction of LRS into diagnostic testing, particularly for patients with genetic disorders with negative results using SRS [11, 12]. However, accurate and precise identification of SVs in specific samples and/or across samples is challenging [13]. In addition, there are incomplete data on the performance and comparison of currently available genomic technologies, the clinical utility of the novel human reference assembly T2T-CHM13, and clinical databases for annotation of detected SVs. This study therefore focused on a comprehensive comparison of SVs detected from whole-genome datasets, both public and our own, obtained by SRS, LRS from currently available technologies, and optical genome mapping (OGM). For this, we introduced an innovative bioinformatics approach LongRead-Checker (LoReC; http://olgen.cz/en/resources) enabling comparisons of SVs across whole-genome datasets, technologies, and reference genomes, including their annotations using clinical genomic databases. This multi-platform approach revolutionizes the comparisons of SVs by introducing key parameters such as distance variance, intersection, and gene overlap between datasets, thus advancing their comparison and annotation across samples or technologies or clinical databases. Our study further highlights the utility of T2T reference and long-read technologies in clinical and research applications.

Materials and methods

Public and our own human whole-genome datasets

This study evaluated whole-genome datasets from a human DNA standard (NA12878 cell line, also known as HG001; B-Lymphocyte; female; healthy; Genome in a Bottle Consortium; [14]) and a breast cancer cell line (SKBR3; [15]) obtained by i) traditional SRS (Illumina), ii) true LRS on a single molecule real-time platform from Pacific Biosciences (LRS-PacBio), iii) true LRS by Oxford Nanopore Technologies (LRS-ONT), iv) synthetic LRS from LRS-TELL-Seq (transposase enzyme-linked long-reads sequencing, Universal Sequencing Technology), v) synthetic LRS from Illumina Complete Long-Reads (LRS-ICLR; Illumina) and vi) synthetic LRS from 10x assay (LRS-10x; 10x Genomics). All of these were synthetic LRS sequenced on an Illumina short-read platform. In addition, OGM from Bionano Genomics was used (Supplementary Table 1). The principles of the LRS technologies used are described elsewhere [16]. Our own datasets from SRS, LRS-TELL-Seq and LRS-ICLR as well as OGM were obtained for two diagnostic samples, P3 (pheochromocytoma adrenal medulla tissue, man, 63 years) and P48 (enriched CD138+ myeloma cells from bone marrow aspirate, woman, 39 years, IgG lambda, stage IIIA, ISS II), as well as for the NA12878 cell line. For our own analysis, the high-molecular-weight DNA from tumour tissues and cells from cell lines was isolated from agarose plugs, as reported previously [17]. The NGS libraries were prepared according to the manufacturer recommendations for LRS-ICLR (Illumina, CA, USA), transposase enzyme-linked long-reads sequencing [TELL-Seq™] (Universal Sequencing technology, MA, USA) and SRS TruSeq DNA PCR-Free (Illumina) and sequenced (150 bp paired-end reads) on NovaSeq 6000 (Illumina). In addition, OGM labelling and measurements using the Bionano Saphyr instrument (Bionano Genomics, CA, USA) were performed as reported previously [17]. The sequence depth for LRS and SRS was approximately 30× and for OGM 300×.

Bioinformatic processing of whole-genome datasets from different short-read sequencing and long-read sequencing platforms

To minimize the difference in precision, recall and F1-score metrics using different callers/aligners, we used the LRS aligner Minimap2 [18] and the SV caller Sniffles2 (v2.2) software [19] for true LRS and the LongRanger software (v2.2.2) [20] for synthetic LRS analyzes, which were found to provide a strong basis for SV pipeline calling in LRS [21]. Moreover, this combination is the basis for the Illumina DRAGEN analysis of Illumina LRS-ICLR, an approach that has also been compared in this study. For SRS datasets, the BWA aligner software (v0.7.17) [22] and the Manta structural variant caller (v1.6.0) [23] were used. The raw data from OGM were analyzed using Bionano Access (v1.8) software by Bionano Genomics, and the De Novo assembly pipeline was performed using Bionano Solve tools (v3.8) (Bionano Genomics).

The hg38 (GRCh38.p14) and T2T-CHM13 (v2.0) human reference genomes were used. The coordinates of genes and pseudogenes, their names, and biotype (e.g. protein-coding) are based on RefSeq NCBI annotations (version 110); in all analyzes, the Y chromosome and ALT contigs were excluded. The list of medically relevant genes is based on the DisGeNET [24] database (DisGeNET v20.1), which includes information on gene/variant disease associations originating from ClinVar, GWAS Catalog, UniProt, GAD and BeFree data [24]. The selection of medically relevant genes is based on gene disease associations (GDA) and variant-disease associations (VDA), GDA/VDA > 0.5 and evidence index > 0.8, indicating that most publications support GDA/VDA [24].

Bioinformatic processing of whole-genome datasets from different short-read sequencing and long-read sequencing platforms

For comparison of SVs across different samples, technologies, and databases, we designed our own LoReC toolkit, containing two tools: the LoReC comparator (source file: variant call format, vcf) and LoReC coverage (source file: mapped reads in binary format, bam).

The LoReC comparator can find the closest SV detected by another technology, database, or reference genome for each SV across the whole genome or region(s) of interest. For each comparison, the following parameters need to be established: 1) the distance variance threshold (the accepted difference in bp between the start and end coordinates between each SV in dataset 1 and the nearest SV in dataset 2, expressed as the sum of the two differences); 2) the intersection factor (the overlap between each SV in dataset 1 and the nearest SV in dataset 2; 0 = 100% overlap, 0–0.5 = partial overlap, >0.5 = no overlap) and 3) the minimal size proportion (the percentage of the size in bp between each SV in dataset 1 and the nearest SV in dataset 2; e.g. 5% means that the SVs in dataset 1 encompass at least 5% of the SVs in dataset 2 or vice versa). To compare SVs in the same regions detected by different technologies, which may differ in size and coordinates because of the principles of the method, the LoReC toolkit lists the closest SV of the same type (e.g. deletion, insertion) from dataset 2 and its coordinates, distance, intersection factor, and size proportion compared with the SV from dataset 1. A distance variance threshold of 1,000 bp was used for a comparison between different NGS platforms and 50,000 bp between NGS and

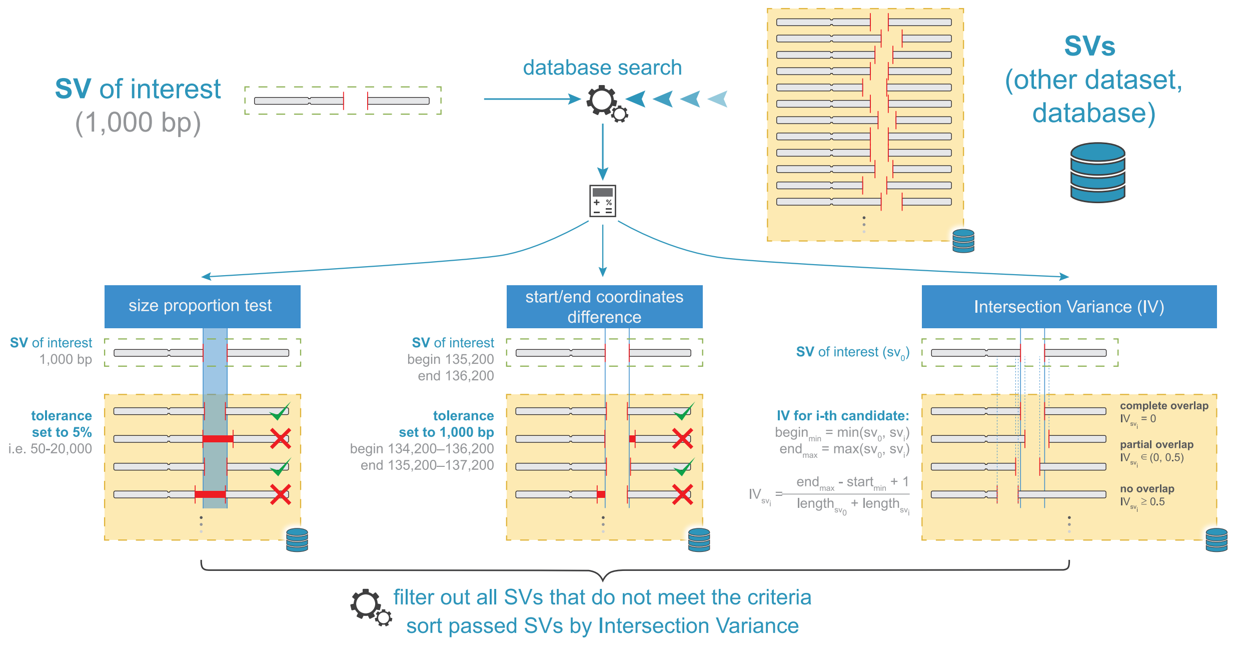

Figure 1. Annotation of structural variants (SVs) detected in the long-read sequencing datasets in clinical diagnostics using the LoReC toolkit. Due to the high variability in SV breakpoints, three measurements are needed for the SV comparison with SVs from clinical databases, such as the distance in bp between the sample SV and the database SV (start and end coordinate difference), the intersection (intersection factor) and the minimum overlap between the sample SV(s) and the database SV(s) (size proportion test).

OGM, and an intersection factor of 0 to 0.5 and a minimum size fraction of 5% were used to indicate SVs that overlap and are very similar; different parameters can be used for filtering (Fig. 1). To visually inspect the SVs of interest, the Samplot tool was used [25].

The LoReC coverage tool is able to provide the coverage of the gene/region of interest, including statistics (mean, median, min, max, Q1 and Q3 coverages), filter reads based on the mapping quality and visualization of the regions of interest. The LoReC coverage outputs are as follows: 1) coverage across regions of interest or across the whole-genome based on the coordinates and gene names given in a region file; 2) coverage calculations based on the mapping quality threshold value, which is able to filter out the reads that map to multiple regions or those of poor quality (MAPQ, MAPQ0 = high probability that a read is mapped to multiple locations with an equal score, MAPQ1 = high probability that a read is mapped to at least two locations, MAPQ50 = 99.999% probability that a read is mapped to a unique region); and 3) visualization of the regions of interest specified in the region files, which allows a comparison of multiple technologies, different samples, or reference genomes. As low coverage genes, those genes that Q1 coverages were below 25% of the mean genome coverage of the sample were marked.

Annotation of structural variants by the LongReadChecker toolkit

Another functionality of the LoReC comparator is to annotate detected SVs based on the annotation file(s) from dbVar (NCBI, https://www.ncbi.nlm.nih.gov/dbvar/; [26]). As the current dbVar_common and ClinVar databases are based on hg38, all SV annotations were performed only on this reference. The following parameters were established for SV comparisons: a threshold of ±1,000 bp distance between the start and end coordinates of the SVs compared, an intersection factor threshold of 0.5 and a minimal size proportion of 5%. First, the detected SVs are compared with the SVs included in the dbVar_common database of common SVs found with a frequency >1% in the population (e.g. nstd186, NCBI Curated Common Structural Variants) or any other vcf file of interest, and must fulfill the set-up criteria to be marked as PASS. Second, the detected SVs not found in the dbVar_common database are compared with the ClinVar SV database (e.g. summary of nstd102, Clinical Structural Variants) or variant file format (VCF) of interest, and for SVs present in the database, the clinical significance of SVs is reported (e.g. pathogenetic, likely pathogenic, VUS, likely benign, benign). For SVs not found in the ClinVar SV database and with an overlap of at least one gene, additional information is reported through the LoReC toolkit based on the NCBI annotation file, which includes the biotype, gene description, and gene/variant disease associations present in DisGeNET.

Results

True long-read sequencing technologies have superior performance in detecting structural variants

To evaluate the performance of the currently available LRS technologies and SRS, whole-genome sequencing datasets for NA12878 healthy [14] and SKBR3 breast cancer [15] cell lines and two diagnostic tissue samples, P3 (pheochromocytoma tissue) and P48 (multiple myeloma bone marrow aspirate), both public and our own (Supplementary Table 1), were compared using different LRS and SRS platforms. Among the tested platforms were the following: 1) two true LRS technologies obtained from Pacific Biosciences (LRS-PacBio) and Oxford Nanopore Technologies (LRS-ONT) instruments; 2) three synthetic linked-read LRS approaches (Illumina Complete Long-Reads (LRS-ICLR), 10x Genomics (LRS-10x), and Universal Sequencing Technology (LRS-TELL-Seq)), sequenced on the short-read Illumina platform; 3) SRS on Illumina; and 4) OGM from Bionano Genomics. For comparisons of detected SVs, their types, coordinates, sizes, intersection factors, size proportions, and coverage between different technologies, reference genomes (hg38/GRCh38.p14; T2T-CHM13, v2.0) and annotations of detected SVs according to clinical databases, a LoReC toolkit was developed (Fig. 1). For more details on functionalities, see Materials and methods section. Using hg38, approximately 25,000 SVs per genome were detected using LRS-PacBio and LRS-ONT, 14,000–15,000 us-

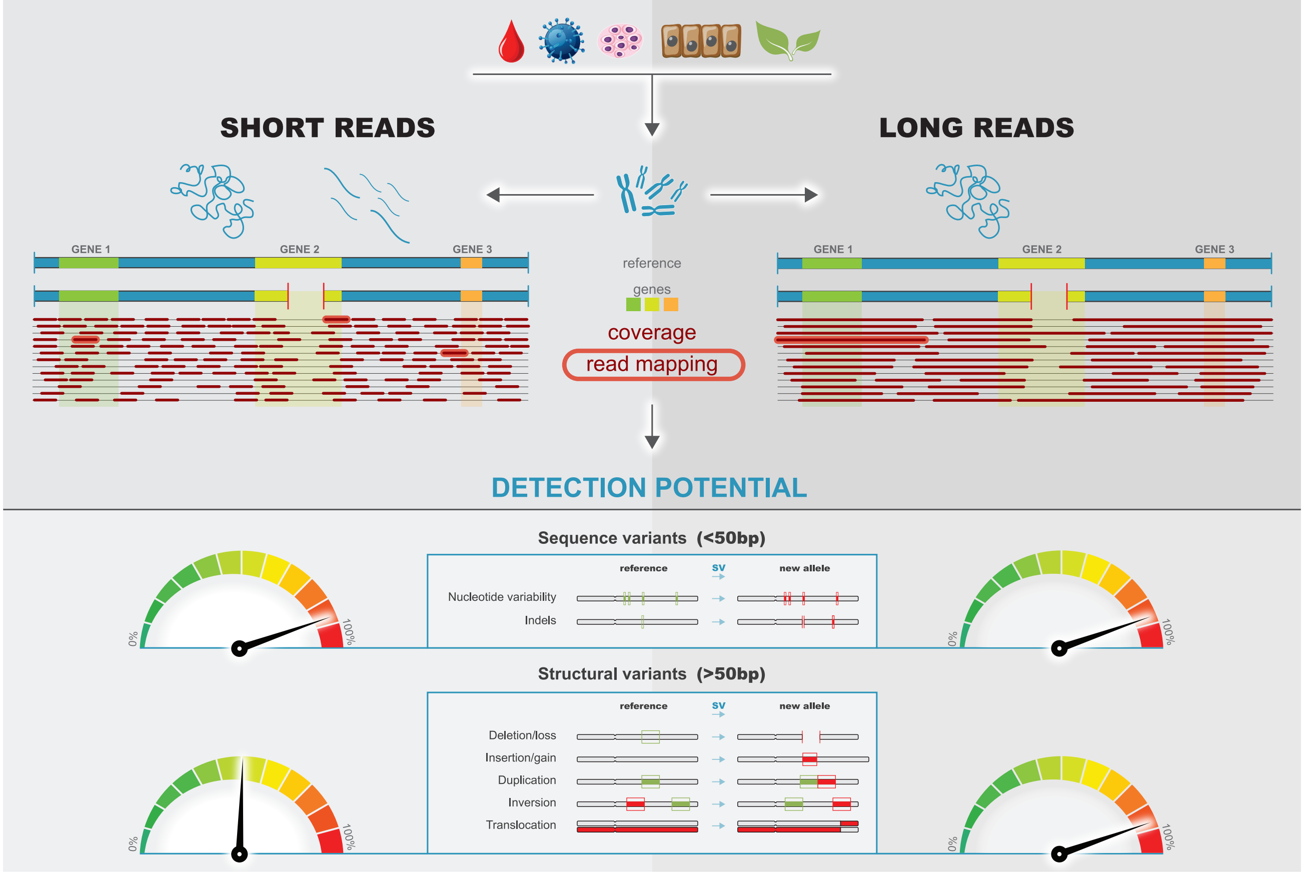

Figure 2. Principle of short-read sequencing (SRS) and long-read sequencing (LRS) and their detection potential for sequence and structural variants. Example of the coverage and read mapping for a heterozygous gene deletion is shown; LRS requires high-molecular-weight DNA than SRS. The lower part shows the detection potential for sequence and structural variants by SRS and LRS.

ing LRS-ICLR, 10,000–12,500 using LRS-10x, 12,500–15,000 using SRS and 4,000 using OGM. Insertions were the most common SVs detected by all technologies: 14,000 per genome using LRS-PacBio and LRS-ONT, 5,000 to 6,000 using LRS-ICLR, 6,000 to 7,000 using SRS and 2,500 using OGM. Insertions were not detected in the LRS-TELL-Seq and LRS-10x datasets, as the LongRanger pipeline available to analyze synthetic reads cannot call insertions. Deletions were the second most common SVs: 10,500 per genome using LRS-PacBio and LRS-ONT, 9,000 to 10,000 using LRS-ICLR, 3,500 to 5,000 using LRS-10x, 5,000 to 6,000 using SRS and 1,250 using OGM. Regarding other SVs such as inversions and duplications, breakends/translocations, their counts varied across samples and genomes, ranging from 240 to 1,430 per genome, depending on the technology (Table 1, Fig. 3, Supplementary Fig. 1). Selected SVs from the OGM and LRS datasets were verified using fluorescence immunophenotyping and interphase cytogenetics as a tool for the investigation of neoplasms (FICTION), arrayCGH and/or targeted SRS.

When comparing available technologies, the majority of deletions and insertions (~80%–95%) detected by LRS-ONT were confirmed by LRS-PacBio; however, less than 50% of them were detected by SRS and 55%–71% by LRS-ICLR. Furthermore, LRS-TELL-Seq and LRS-10x confirmed approximately 25%–50% of the deletions detected by LRS-ONT/LRS-PacBio, but it was not possible to call insertions from synthetic reads. Regarding inversions, duplications, and breakends/translocations, the best overlap was observed between LRS-PacBio and LRS-ONT, and a moderate overlap was identified using synthetic LRS and SRS (Supplementary Tables 2, 3).

Most structural variants detected by short-read sequencing were confirmed by long-read sequencing

Next, we compared the SVs detected by SRS, the most widely used platform today in clinical diagnostics, with SVs detected by LRS-ONT and LRS-PacBio and synthetic LRS. Most of the deletions and insertions detected by SRS (10,000 to 12,500 per genome) were smaller than 0.5 kbp (~80%) and were confirmed by LRS-PacBio/LRS-ONT (~90%). LRS-ICLR was similar to true LRS in detecting deletions, but has a lower precision in the case of insertions (~50%) as this technology is based on SRS. Notably, SRS did not detect approximately 50% of the SVs detected by LRS (Supplementary Tables 2, 4).

True long-read sequencing and short-read sequencing technologies have superior performance over optical genome mapping

We also compared LRS and SRS with OGM, a non-sequencing technology based on the labeling of high-molecular weight DNA using fluorophore tags on specific sequence motifs. As OGM does not detect SVs smaller than 0.5 kbp or SVs in genomic regions that lack specific sequence motifs, OGM detected a lower number of SVs than LRS or SRS (hg38: 3,946 vs 24,941 vs 12,953; T2T-CHM13: 3,082 vs 24,516 vs 6,769). The majority (~80%) of deletions and insertions detected by OGM were confirmed by LRS-ONT and LRS-PacBio; SRS confirmed ~35% of the insertions and ~50%–60% of the deletions detected by OGM (Supplementary Tables 2, 5). Comparison of OGM with LRS and SRS showed that OGM does not provide the exact coordinates of individual SVs and their position may be significantly different compared to coordinates detected by LRS and

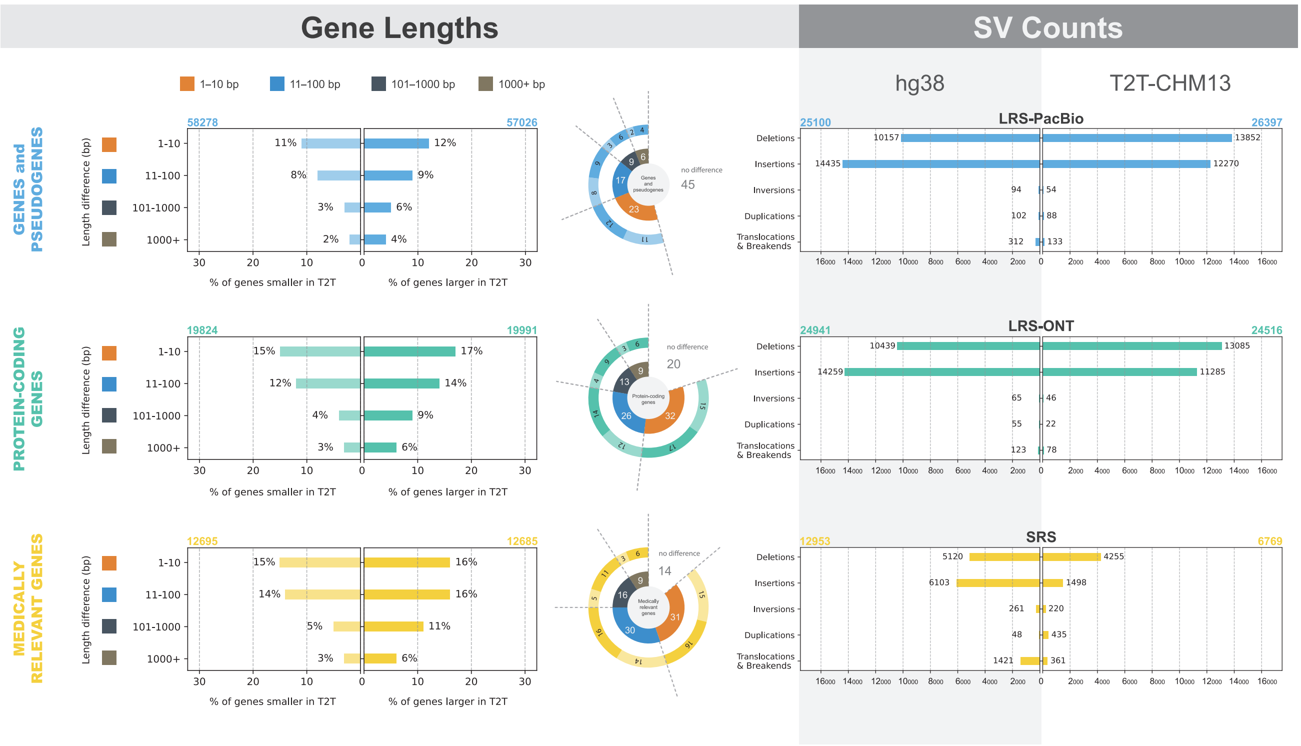

Figure 3. Comparison of gene lengths and the number of structural variants (SVs) in the NA12878 cell line using hg38 and T2T-CHM13 references. The differences in the number and lengths of genes and pseudogenes, protein-coding genes and medically relevant genes for both references are presented; genes were annotated based on RefSeq NCBI (version 110, based on gene IDs), excluding genes on the Y chromosome and ALT contigs. The number of SVs detected by long-read sequencing (LRS-PacBio, LRS-ONT) and short-read sequencing (SRS) on both references are shown on the right part.

SRS (Supplementary Fig. 2).

True long-reads map with high probability to unique regions compared with short-reads

Next, we evaluated the read mapping quality (MAPQ) [22], a measure of the probability that a read is misplaced, for different LRS and SRS technologies. For MAPQ0, a default setting in current SRS/LRS aligners and variant callers that allows read mapping to multiple regions, most genes were covered for all SRS/LRS technologies (Fig. 2, Supplementary Fig. 3, Supplementary Table 6). To eliminate problematic regions with misplaced reads, current SRS pipelines often mask these repetitive 'dark regions'. When stricter MAPQ1 and MAPQ50 associated with a lower probability of misplaced reads compared to MAPQ0 were applied, many regions of the genome were not covered in the SRS datasets, including many protein-coding genes and medically relevant genes (Fig. 2, Supplementary Fig. 3, Supplementary Table 6). In LRS-PacBio high-fidelity (HiFi) datasets, most reads were mapped with high probability to a unique region, as demonstrated by applying MAPQ1/MAPQ50. Regarding LRS-ONT, mapping to multiple regions is less probable because of the very long-reads (up to Mbp); however, many reads are of low quality, resulting in less accuracy in mapping (Fig. 2, Supplementary Fig. 3, Supplementary Table 6).

T2T-CHM13 reference improves the analysis of structural variants for long-read sequencing and short-read sequencing datasets

To understand the added value of the gapless T2T-CHM13 reference with the currently used hg38, SVs in known genes and pseudogenes, protein-coding genes, and medically relevant genes and their lengths were compared for the LRS and SRS datasets, excluding the Y chromosome and alternate (ALT) contigs (Fig. 3, Supplementary Table 6). Using the T2T-CHM13 reference, the number of deletions increased by more than 20%, and the number of insertions decreased by more than 20% using the LRS-ONT and LRS-PacBio datasets compared to hg38 (Fig. 3, Supplementary Fig. 1, Table 1). Using SRS, approximately 10% more deletions and 80% fewer insertions were detected using T2T-CHM13 than using hg38 (Fig. 3, Supplementary Fig. 1, Table 1). In the unique regions of T2T-CHM13, numerous translocations, deletions, and other SVs were detected in the telomere, centromere, and subcentromere regions in all analyzed datasets (Supplementary Tables 3-5, Supplementary Fig. 4). The T2T-CHM13 reference also refined the length of the genes. Comparing T2T-CHM13 and hg38 references for 54,553 genes and pseudogenes that overlap (gene IDs in RefSeq NCBI annotations, version 110) (Fig. 3, Supplementary Table 6), 45.6% had the same length, 23.7% differed by 1–10 bp, 16.8% by 11–100 bp, 8.2% by 101–1,000 bp and 5.6% by more than 1,000 bp. Regarding protein-coding genes (medically relevant genes), 19.9% (14.3%) had the same length, 31.7% (31.0%) differed by 1–10 bp, 26.7% (30.2%) by 11–100 bp, 13.3% (15.8%) by 101–1,000 bp and 8.4% (8.8%) by more than 1,000 bp (Fig. 3). Among those with the largest differences in length were *GRK1* [27] and *LPA* [28], many other genes like *SMN1&2* [29], *DUX4* and *HLA-DRB5* or gene *GBA* and its pseudogene *GBAP1*, were found disassembled, not correctly assembled or highly similar in hg38 (Supplementary Fig. 5, Supplementary Fig. 6, Supplementary Table 6). Furthermore, 167 more protein-coding genes were annotated in T2T-CHM13 than in hg38 (Supplementary Table 6). To complement the added value of T2T-CHM13, we added an annotation about genes found in the discrepant regions in hg19 and hg38 [30, 31] (Supplementary Table 6).

Table 1. Structural variants detected in the NA12878 and SKBR3 cell lines and the P3 and S48 diagnostic samples using SRS and different LRS technologies for the hg38 and T2T-CHM13 human references.

Sample	SV type	Total number of SVs		Deletions		Insertions		Duplications		Inversions		Breakends/ Translocations	
	References/ Technology	hg38	T2T-CHM13	hg38	T2T-CHM13	hg38	T2T-CHM13	hg38	T2T-CHM13	hg38	T2T-CHM13	hg38	T2T-CHM13
NA12878	SRS	12,912	6,769	5,120	4,255	6,103	1,498	48	435	261	220	1,421	361
	LRS-PacBio	24,811	26,397	10,157	13,852	14,435	12,270	102	88	94	54	312	133
	LRS-ONT	24,793	24,516	10,439	13,085	14,259	11,285	55	22	65	46	123	78
	LRS-ICLR	15,410	NA	9,412	NA	5,872	NA	28	NA	80	NA	38	NA
	LRS-10x	11,800	15,120	3,542	7,989	ND	ND	849	780	6,623	5,867	786	484
	LRS-TELL-Seq	8,543	10,873	4,964	6,096	ND	ND	54	162	3,432	4,514	93	101
	OGM	3,947	3,082	1,283	1,414	2,556	1,523	44	38	64	42	0	65
SKBR3	SRS*	5,324	4,011	2,920	2,738	937	598	937	238	258	212	272	225
	LRS-PacBio	32,850	32,533	9,097	11,321	22,994	20,630	154	120	235	207	370	255
	LRS-ONT	23,597	21,291	10,983	12,200	12,221	8,749	49	39	170	152	174	151
	LRS-10x	10,319	12,140	4,918	3,393	ND	ND	1,318	6,830	3,393	1,300	690	617
	OGM	4,030	3,007	1,283	1,413	2,403	1,365	64	60	98	66	182	103
P3	SRS	12,823	6,441	5,018	4,079	5,993	1,418	54	444	246	196	1,512	304
	LRS-ICLR	14,513	NA	9,166	NA	5,228	NA	28	NA	60	NA	31	NA
	LRS-TELL-Seq	8,434	9,171	4,582	5,385	ND	ND	193	153	3,553	3,524	106	109
	OGM	4,076	3,130	1,262	1,335	2,638	1,625	84	64	71	34	21	72
S48	SRS	14,846	8,386	5,632	4,900	6,862	2,089	72	554	284	256	1,996	587
	LRS-ICLR	15,342	NA	9,279	NA	5,951	NA	24	NA	62	NA	26	NA
	LRS-TELL-Seq	9,960	10,888	5,281	6,109	ND	ND	153	164	4,436	4,514	90	101
	OGM	3,796	2,877	1,225	1,326	2,432	1,421	55	44	72	46	12	40

Legend: SRS, short-read sequencing by Illumina platform; LRS-PacBio, true long-read sequencing by Pacific Biosciences; LRS-ONT, true long-read sequencing by Oxford Nanopore Technologies; LRS-ICLR, synthetic long-read sequencing by Illumina - complete long-reads technology on Illumina platform; LRS-TELL-Seq, synthetic long-read sequencing by Universal Sequencing Technology on Illumina platform; LRS-10x, synthetic long-read sequencing by 10x Genomics on Illumina platform; SVs, structural variants; OGM, optical genome mapping by Bionano Genomics; NA, not available; ND, not detected. *dataset with average coverage 20× (less than in other datasets, where 30× coverage was achieved).

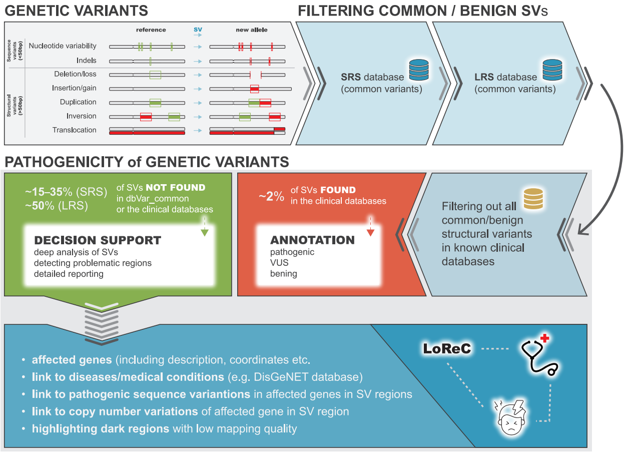

Figure 4. Suggested workflow for annotation of structural variants (SVs) detected by long-read sequencing (LRS) in diagnostic samples. First, SVs in the samples are filtered using a dbVar_common database or other to filter out the common and benign SVs. The SVs not found in the dbVar_common database are compared with the ClinVar SV database or similar. For SVs not found in the ClinVar database, the LoReC toolkit is used for the comparison and annotation of SVs in the samples based on the NCBI annotation file(s), including affected gene (gene description, coordinates) and link to the disease or medical condition based on the gene/variant disease associations present in DisGeNET or similar.

Annotation of structural variants in clinical databases is challenging

To annotate the SVs detected by tested technologies, we used a LoReC comparator toolkit to compare the detected SVs with the dbVar_common (NCBI dbVar Curated Common Structural Variants, GnomAD and other sources, ID: nstd186) [26] and ClinVar (ID: nstd102) clinical datasets [32]. DbVar_common database includes those SVs that occur with a frequency >1% in the population. As the current ClinVar database is based on hg38, all annotations of SVs were performed using this reference. Given that SV coordinates may vary from individual to individual, the following parameters need to be defined prior to each analysis: 1) the distance variance threshold (acceptable difference in bp between the SV of interest and the SV in the clinical database), 2) the intersection factor (overlap between the SV of interest and the nearest SV in the clinical database) and 3) the minimum size fraction (minimum proportion of the SV of interest and the nearest SV in the clinical database) (see the Methods for details). All SVs were also evaluated using the AnnotSV tool [33] (Table 2, Fig. 3). Regarding deletions, approximately 65% of the deletions detected by the SRS datasets overlapped with the deletions in dbVar_common and approximately 2% with those in ClinVar. When using AnnotSV, 83% of the SVs in the SRS datasets were annotated as benign, approximately 1% (30–90) deletions per sample were annotated as pathogenic/likely pathogenic and less than 18% as a variant of unknown significance (VUS). Using true LRS, approximately 50% of deletions were detected in dbVar_common and 2% in ClinVar; for more than half of deletions, no annotation was available (Table 2). Using ClinVar annotations, the majority of SVs in this database span very large regions containing many genes; therefore, they do not meet the size proportion criteria of the PASS filters for SVs detected by LRS. When using AnnotSV, 50%–60% of SVs in the LRS datasets were annotated as benign, approximately 10 deletions (~0.1%) were annotated as pathogenic/likely pathogenic and approximately 4,500 deletions (30%–50%) as VUS, depending on the sample and technology used (Table 2). Regarding insertions, the dbVar_common/ClinVar databases contain only breakpoints and lack information on insertion length for most insertions, making annotation impossible, which is also problematic for inversions and translocations.

Table 2. Annotations of deletions detected in whole-genome datasets for the NA12878 and SKBR3 cell lines and P3 and S48 diagnostic samples by SRS and LRS technologies employing A) LoReC using dbVar_common and ClinVar and B) AnnotSV, both for the hg38 reference.

Sample	ALL		A) LoReC						B) AnnotSV			
	Technology	TOTAL DEL	dbVar_common	ClinVar	Pathogenic/ Likely Pathogenic	VUS	Benign/ Likely Benign	Not annotated	TOTAL DEL+BND	Benign	Pathogenic/ Likely Pathogenic	VUS (Not annotated)
NA12878	SRS	5,120	3,332 (65%)	135 (3%)	0/1	12	121/0	1,754 (34%)	5,525	4,598 (83%)	32/6	889 (16%)
	LRS-PacBio	10,157	4,588 (45%)	221 (2%)	0/4	17	198/1	5,466 (54%)	10,157	6,030 (59%)	7/6	4,114 (41%)
	LRS-ONT	10,439	4,580 (44%)	221 (2%)	0/5	17	196/2	5,767 (55%)	10,439	5,893 (56%)	2/7	4,537 (43%)
	LRS-ICLR	9,412	3,978 (42%)	195 (2%)	1/3	14	175/1	5,372 (57%)	9,412	4,767 (51%)	2/4	4,639 (49%)
SKBR3	SRS	2,920	2,239 (77%)	101 (4%)	1/0	5	92/0	597 (20%)	2,890	2,373 (82%)	46/32	457 (16%)
	LRS-PacBio	9,097	4,098 (45%)	196 (2%)	1/4	10	175/3	4,880 (54%)	9,097	6,095 (67%)	30/10	2,962 (33%)
	LRS-ONT	10,983	4,236 (39%)	190 (2%)	3/4	10	169/1	6,634 (60%)	10,983	7,183 (65%)	29/15	3,756 (34%)
P3	SRS	5,018	3,221 (64%)	128 (3%)	0/0	13	113/1	1,771 (35%)	5,478	4,463 (81%)	36/4	975 (18%)
	LRS-ICLR	9,166	3,856 (42%)	172 (2%)	0/3	17	151/0	5,258 (57%)	9,166	4,486 (49%)	0/3	4,677 (51%)
S48	SRS	5,632	3,469 (62%)	129 (2%)	0/0	12	116/0	2,115 (38%)	6,199	5,035 (81%)	84/6	1,074 (17%)
	LRS-ICLR	9,279	3,918 (42%)	144 (2%)	0/3	14	156/3	5,280 (57%)	9,279	4,715 (51%)	5/6	4,553 (49%)

Legend: SRS, short-read sequencing by Illumina platform; LRS-PacBio, true long-read sequencing by Pacific Biosciences; LRS-ONT, true long-read sequencing by Oxford Nanopore Technologies; LRS-ICLR, synthetic long-read sequencing by Illumina - complete long-reads technology on Illumina platform; LRS-TELL-Seq, synthetic long-read sequencing by Universal Sequencing Technology on Illumina platform; LRS-10x, synthetic long-read sequencing by 10x Genomics on Illumina platform; OGM, optical genome mapping by Bionano Genomics; VUS, variant of unknown significance; DEL, deletion; BND, Breakends.

Annotation of the structural variants not presented in the clinical databases by LoReC

We suggested a workflow for diagnostic laboratories to annotate SVs from LRS/SRS. Briefly, after filtering out benign/common SVs present in the databases (nowadays mainly based on SRS, but in the future LRS datasets will be added), the remaining SVs will be compared with clinical databases ClinVar or similar databases (Fig. 4). For SVs not presented in the dbVar_common/ClinVar databases, our LoReC toolkit enables the addition of custom annotations for regions/genes within the detected SVs. In particular, genes may be linked to the gene/variant disease associations provided in the annotation file (e.g. DisGeNET) or other custom annotations (Supplementary Table 6). In addition, the LoReC toolkit provides coverage for genes and regions, mapping quality, and enables the visualization of the regions of interest for expert evaluation.

Discussion

The field of SV importance in clinical diagnostics continues to expand due to advances in genomic technologies and wet lab protocols, the introduction of gap-less reference sequence T2T-CHM13, and the release of high-quality datasets. Despite the growing importance of SVs in human diseases, our knowledge of SVs in health and disease is limited, largely due to their structural complexity and variable length in different individuals, as well as the limitations inherent in available genomic technologies. Here, we comprehensively evaluated SVs from human whole-genome datasets obtained from SRS, all available LRS and OGM platforms across different samples, technologies and clinical databases using two human references (hg38 and T2T-CHM13). Moreover, our multi-platform approach enables annotation of detected SVs to support the implementation of SV diagnostics in clinical practice.

Despite advances in clinical genetic diagnostics, approximately 50% of all suspected Mendelian diseases and many sporadic development syndromes and chronic diseases remain unresolved [34, 35]. Diagnostics of SVs is relevant not only in rare genetic diseases, but SVs are also important contributors to chronic diseases, including cancer [5, 6, 36]. However, the technical limitations inherent in the available genomic technologies, as well as the structural complexity of SVs and their variable length in different individuals, have led to an incomplete characterization of SVs in the human genome in health and disease compared to single nucleotide variations and small INDELs. To gain more insights into the performance of available genomic technologies, we compared our own and public whole-genome datasets from SRS, two LRS platforms (PacBio-LRS, ONT-LRS) requiring specific instruments, and three LRS approaches utilizing synthetic long-reads sequenced on conventional short-read next-generation sequencers (NGS). For this, we developed the multi-platform LoReC toolkit, which compares the size and type of SVs, their overlap, coverage, coordinates, affected genes and disease associations from different SRS and LRS datasets in specific samples and/or across samples and databases, regardless of which of the many available algorithms was used to detect SVs [37, 38].

First, we were interested in the performance of SRS, as this gold standard method remains essential in diagnostics due to practical and cost considerations, compared to third-generation technologies. On average, approximately 13,000 SVs/genome were detected by SRS and twice as many (∼25,000 SVs/genome) by LRS. Notably, most of the SVs detected by SRS were also detected by LRS. Our findings are consistent with others showing that LRS can identify hidden disease-related SVs that are not detected by SRS [3, 31, 39]. Since 80% of the SVs detected by LRS/SRS were smaller than 0.5 kbp, OGM did not detect most of the SVs detected by LRS/SRS. The most common type of SVs were deletions and insertions, whereas half as many deletions (∼5,000 vs ∼10,000) and insertions (∼6,000 vs more than 12,000) per sample were detected by SRS compared with LRS. Our data reveal that LRS-ONT and LRS-PacBio technologies have superior performance in detecting SVs to the SRS, the synthetic read LRS-ICLR, LRS-10x, LRS-TELL-Seq, and OGM.

In addition to detecting more SVs in LRS, longer reads map more uniquely to the genome than SRS. Despite using the default MAPQ0 settings employed in the current SRS/LRS aligners and variant callers that allow the use of misplaced reads, effective coverage of most genes across genomes was achieved for all SRS/LRS technologies. To address this issue, current SRS pipelines often mask these problematic 'dark regions', which include repetitive elements and polymorphic regions, potentially leading to the loss of key information in these regions. In addition, PacBio provides a BED file for 'dark regions' that occur in repetitive areas or areas with high GC content. When stricter MAPQ1 or MAPQ50 were applied, many regions across the genome were not covered well in the SRS datasets, including numerous protein-coding genes and medically relevant genes. Of the technologies analyzed, the lowest probability of mismatches was observed for the data obtained by LRS-PacBio HiFi, the highest for SRS. The LRS-ONT datasets demonstrated a high percentage of low-quality reads due to lower nucleotide accuracy when using the MAPQ0 setting. When using MAPQ1 or MAPQ50 to filter out low-quality reads, uniform coverage across the whole genome with high-quality reads and very long-reads generated was achieved by LRS-ONT. Notably, LRS-ONT has introduced adaptive sampling, a computational enrichment technique that adjusts the sequencing parameters in the regions of interest [40, 41], enabling deeper coverage in these regions [42].

Another key step for clinical genetics is the introduction of gap-less T2T-CHM13 human reference assembly, uncovering 8% of the dark regions of the genome, adds nearly 200 million bases, and predicts 99 novel protein coding genes compared to reference hg38 [43]. Our comprehensive bioinformatic analysis of whole-genome datasets further supports the clinical utility of using the T2T-CHM13 sequence for medical diagnostics. When we compared medically relevant genes using coordinates based on NCBI RefSeq annotations on both references, approximately 85% of the genes changed size and more than half by more than 10 bp. Notably, approximately 9% of the medically relevant genes differed by >1,000 bp between hg38 and T2T-CHM13. Among the genes significantly differing between both references were those that were disassembled, not correctly assembled, or highly similar in hg38, such as the challenging medically relevant genes *GRK1*, *LPA*, *SMN1&2*, *DUX4* and *HLA-DRB5* or the *GBA* gene and its pseudogene *GBAP1*, etc. [44]. In addition, the novel T2T-CHM13 reference resulted in approximately 20% more deletions and 20% fewer insertions than hg38. Taken together, the gene length and SV changes may reflect the repetitive nature and complexity of some medically relevant genes, further highlighting the advantage of LRS and the T2T-CHM13 reference for clinical medicine.

Another challenging topic is the pathogenicity annotations of thousands of SVs detected by LRS/SRS. Unlike sequence variants, for whose interpretation there are standards and guidelines [45, 46] and clinical databases such as dbVar_common/ClinVar based on large available SRS datasets, SV annotations are more difficult not only due to the insufficient number of LRS datasets in the databases, but also due to the significant variability in SV break points between individuals and the complexity of rearrangements. Therefore, we introduced crucial measures for SV evaluations, such as the distance in bp between the sample SVs and the datasets/references/databases SVs, the intersection and minimum overlap of the sample SVs with the datasets/references/databases SVs. When we annotated deletions in LRS datasets using dbVar_common/ClinVar or AnnotSV [33], approximately 45% of deletions were detected in dbVar_common and less than 2% were annotated in ClinVar. Notably, for approximately 5,500 deletions (56%) per genome, many of them in protein-coding sequences, no annotation was available in the current version of the ClinVar database. Moreover, the majority of

deletions in the ClinVar database span very large regions containing many genes; therefore, they do not meet the size proportion criteria of the PASS filters for SVs detected by LRS. Regarding insertions and other SVs, the length of the insertion and sequence of the insert are missing in the dbVar_common/ClinVar databases, making their annotation impossible. For SVs not annotated by ClinVar, we applied the LoReC toolkit, and, with its help, the affected gene(s) can be linked with gene/variant disease associations using DisGeNET, human phenotype ontology, or a similar database. In the future, this approach will allow SV annotations to be matched to continuously updated clinical databases based on the LRS datasets and linked to the reference T2T-CHM13.

This study and other studies [11, 12, 39, 42, 47, 48, 49] further support the introduction of LRS into medical diagnostics in the near future, especially in cases with negative SRS results. To introduce LRS into diagnostics, laboratories should collect cells for high molecular weight DNA isolation, as LRS cannot be performed on fragmented DNA obtained by standard isolation methods and increases computational and data storage capacity due to the large datasets obtained by LRS. We highlight the introduction of distance variance, intersection, gene overlap and the closest SV in the clinical database for SVs comparisons and annotations, which is currently the weakest point of SV implementation into clinical diagnostics. Although this first comprehensive study on the performance of all available genomic technologies is focused on SVs in the human genome, SV events are widespread in other species, and our toolkit is suitable for these datasets as well.

Conclusions

In this study, we introduced innovative multi-platform approach for any SRS and third-generation dataset that advance SV comparisons across the samples and databases as well as annotations of SVs based on comparisons with the clinical databases. Despite that gold standard SRS may uncover thousands of SVs that may be clinically relevant, we showed that LRS is more effective at detecting SVs than SRS. Thus, LRS is expected to complement SRS analysis of negative samples in clinical diagnostics soon as the identification of novel genetic biomarkers can be used to individually tailor the prevention and treatment not only in chronic diseases. However, the implementation of LRS will also require the introduction of isolation methods leading to high molecular weight DNA and the update of clinical databases to include LRS datasets and the T2T-CHM13 reference for correct annotations of SVs.

Declarations

Ethical Approval

The patients provided written informed consent for the use of their biological materials for the purpose of this study, which was conducted in accordance with the Helsinki Declaration and approved by the local ethics committee (NW25-03-00391).

Consent for publication

Not applicable

Competing Interests

The authors have no competing interests to declare.

Funding

This study was supported by the Internal Grant Agency of Palacký University (JG_2025_035, IGA_LF_2025_014), NW24-10-00395 and, in part, by the Ministry of Health of the Czech Republic (MH CZ – DRO (FNOL, 00098892).

Author's Contributions

JS and EK designed the research; TN and JS developed the LoReC toolkit; JS, TN, PG and MB performed the bioinformatic analysis; JM and AP performed the laboratory experiments; MM, TP and FC collected the patient samples and clinical characteristics; JS and EK wrote the manuscript; and PG and AP critically revised the manuscript. All authors read and approved the final manuscript.

Code availability

Code is available at: https://github.com/novosadt/lorec-comparator.git and https://github.com/novosadt/lorec-coverage.git.

References

1. Collins RL, Talkowski ME. Diversity and consequences of structural variation in the human genome. Nature Reviews Genetics 2025 Jan;https://www.nature.com/articles/s41576-024-00808-9.
2. Logsdon GA, Vollger MR, Eichler EE. Long-read human genome sequencing and its applications. Nature Reviews Genetics 2020 Oct;21(10):597–614. https://www.nature.com/articles/s41576-020-0236-x, publisher: Nature Publishing Group.
3. Mahmoud M, Huang Y, Garimella K, Audano PA, Wan W, Prasad N, et al. Utility of long-read sequencing for All of Us. Nature Communications 2024 Jan;15(1):837. https://www.nature.com/articles/s41467-024-44804-3, publisher: Nature Publishing Group.
4. Sudmant PH, Rausch T, Gardner EJ, Handsaker RE, Abyzov A, Huddleston J, et al. An integrated map of structural variation in 2,504 human genomes. Nature 2015 Oct;526(7571):75–81. https://www.nature.com/articles/nature15394, publisher: Nature Publishing Group.
5. DeBoever C, Tanigawa Y, Lindholm ME, McInnes G, Lavertu A, Ingelsson E, et al. Medical relevance of protein-truncating variants across 337,205 individuals in the UK Biobank study. Nature Communications 2018 Apr;9(1):1612. https://www.nature.com/articles/s41467-018-03910-9, publisher: Nature Publishing Group.
6. Collins RL, Brand H, Karczewski KJ, Zhao X, Alföldi J, Francioli LC, et al. A structural variation reference for medical and population genetics. Nature 2020 May;581(7809):444–451. https://www.nature.com/articles/s41586-020-2287-8, publisher: Nature Publishing Group.
7. Kosugi S, Momozawa Y, Liu X, Terao C, Kubo M, Kamatani Y. Comprehensive evaluation of structural variation detection algorithms for whole genome sequencing. Genome Biology 2019 Jun;20(1):117. https://doi.org/10.1186/s13059-019-1720-5.
8. Escaramís G, Docampo E, Rabionet R. A decade of structural variants: description, history and methods to detect structural variation. Briefings in Functional Genomics 2015 Sep;14(5):305–314. https://doi.org/10.1093/bfgp/elv014.
9. Ahsan MU, Liu Q, Perdomo JE, Fang L, Wang K. A survey of algorithms for the detection of genomic structural variants from long-read sequencing data. Nature Methods 2023

Aug;20(8):1143–1158. https://www.nature.com/articles/s41592-023-01932-w, publisher: Nature Publishing Group.

10. Amarasinghe SL, Su S, Dong X, Zappia L, Ritchie ME, Gouil Q. Opportunities and challenges in long-read sequencing data analysis. Genome Biology 2020 Feb;21(1):30. https://doi.org/10.1186/s13059-020-1935-5.
11. Mantere T, Kersten S, Hoischen A. Long-Read Sequencing Emerging in Medical Genetics. Frontiers in Genetics 2019 May;10. https://www.frontiersin.org/journals/genetics/articles/10.3389/fgene.2019.00426/full, publisher: Frontiers.
12. Sanford Kobayashi E, Batalov S, Wenger AM, Lambert C, Dhillon H, Hall RJ, et al. Approaches to long-read sequencing in a clinical setting to improve diagnostic rate. Scientific Reports 2022 Oct;12(1):16945. https://www.nature.com/articles/s41598-022-20113-x, publisher: Nature Publishing Group.
13. Mahmoud M, Gobet N, Cruz-Dávalos DI, Mounier N, Dessimoz C, Sedlazeck FJ. Structural variant calling: the long and the short of it. Genome Biology 2019 Nov;20(1):246. https://doi.org/10.1186/s13059-019-1828-7.
14. Zook JM, Catoe D, McDaniel J, Vang L, Spies N, Sidow A, et al. Extensive sequencing of seven human genomes to characterize benchmark reference materials. Scientific Data 2016 Jun;3(1):160025. https://www.nature.com/articles/sdata201625, publisher: Nature Publishing Group.
15. Aganezov S, Goodwin S, Sherman RM, Sedlazeck FJ, Arun G, Bhatia S, et al. Comprehensive analysis of structural variants in breast cancer genomes using single-molecule sequencing. Genome Research 2020 Sep;30(9):1258–1273. http://genome.cshlp.org/content/30/9/1258, company: Cold Spring Harbor Laboratory Press Distributor: Cold Spring Harbor Laboratory Press Institution: Cold Spring Harbor Laboratory Press Label: Cold Spring Harbor Laboratory Press Publisher: Cold Spring Harbor Lab.
16. van Dijk EL, Naquin D, Gorrichon K, Jaszczyszyn Y, Ouazahrou R, Thermes C, et al. Genomics in the long-read sequencing era. Trends in Genetics 2023 Sep;39(9):649–671. https://www.sciencedirect.com/science/article/pii/S0168952523001191.
17. Kriegova E, Fillerova R, Minarik J, Savara J, Manakova J, Petrackova A, et al. Whole-genome optical mapping of bone-marrow myeloma cells reveals association of extramedullary multiple myeloma with chromosome 1 abnormalities. Scientific Reports 2021 Jul;11(1):14671. https://www.nature.com/articles/s41598-021-93835-z, number: 1 Publisher: Nature Publishing Group.
18. Li H. Minimap2: pairwise alignment for nucleotide sequences. Bioinformatics 2018 Sep;34(18):3094–3100. https://doi.org/10.1093/bioinformatics/bty191.
19. Smolka M, Paulin LF, Grochowski CM, Horner DW, Mahmoud M, Behera S, et al. Detection of mosaic and population-level structural variants with Sniffles2. Nature Biotechnology 2024 Jan;p. 1–10. https://www.nature.com/articles/s41587-023-02024-y, publisher: Nature Publishing Group.
20. Marks P, Garcia S, Barrio AM, Belhocine K, Bernate J, Bharadwaj R, et al. Resolving the full spectrum of human genome variation using Linked-Reads. Genome Research 2019 Apr;29(4):635–645.
21. Helal AA, Saad BT, Saad MT, Mosaad GS, Aboshanab KM. Benchmarking long-read aligners and SV callers for structural variation detection in Oxford nanopore sequencing data. Scientific Reports 2024 Mar;14(1):6160. https://www.nature.com/articles/s41598-024-56604-2, publisher: Nature Publishing Group.
22. Li H, Durbin R. Fast and accurate short read alignment with Burrows-Wheeler transform. Bioinformatics (Oxford, England) 2009 Jul;25(14):1754–1760.
23. Chen X, Schulz-Trieglaff O, Shaw R, Barnes B, Schlesinger F, Källberg M, et al. Manta: rapid detection of structural variants and indels for germline and cancer sequencing applications. Bioinformatics 2016 Apr;32(8):1220–1222. https://doi.org/10.1093/bioinformatics/btv710.
24. Piñero J, Ramírez-Anguita JM, Saüch-Pitarch J, Ronzano F, Centeno E, Sanz F, et al. The DisGeNET knowledge platform for disease genomics: 2019 update. Nucleic Acids Research 2020 Jan;48(D1):D845–D855. https://doi.org/10.1093/nar/gkz1021.
25. Belyeu JR, Chowdhury M, Brown J, Pedersen BS, Cormier MJ, Quinlan AR, et al. Samplot: a platform for structural variant visual validation and automated filtering. Genome Biology 2021 May;22(1):161. https://doi.org/10.1186/s13059-021-02380-5.
26. Lappalainen I, Lopez J, Skipper L, Hefferon T, Spalding JD, Garner J, et al. dbVar and DGVa: public archives for genomic structural variation. Nucleic Acids Research 2013 Jan;41(D1):D936–D941. https://doi.org/10.1093/nar/gks1213.
27. Xie H, Li W, Hu Y, Yang C, Lu J, Guo Y, et al. De novo assembly of human genome at single-cell levels. Nucleic Acids Research 2022 Jul;50(13):7479–7492. https://doi.org/10.1093/nar/gkac586.
28. Chin CS, Behera S, Metcalf GA, Gibbs RA, Boerwinkle E, Sedlazeck FJ, A pan-genome approach to decipher variants in the highly complex tandem repeat of LPA. bioRxiv; 2022. https://www.biorxiv.org/content/10.1101/2022.06.08.495395v2, pages: 2022.06.08.495395 Section: New Results.
29. Chen X, Harting J, Farrow E, Thiffault I, Kasperaviciute D, Hoischen A, et al. Comprehensive SMN1 and SMN2 profiling for spinal muscular atrophy analysis using long-read PacBio HiFi sequencing. American Journal of Human Genetics 2023 Feb;110(2):240–250. https://www.ncbi.nlm.nih.gov/pmc/articles/PMC9943720/.
30. Yang X, Wang X, Zou Y, Zhang S, Xia M, Fu L, et al. Characterization of large-scale genomic differences in the first complete human genome. Genome Biology 2023 Jul;24(1):157. https://doi.org/10.1186/s13059-023-02995-w.
31. Mandelker D, Schmidt RJ, Ankala A, McDonald Gibson K, Bowser M, Sharma H, et al. Navigating highly homologous genes in a molecular diagnostic setting: a resource for clinical next-generation sequencing. Genetics in Medicine 2016 Dec;18(12):1282–1289. https://www.sciencedirect.com/science/article/pii/S1098360021014258.
32. Landrum MJ, Lee JM, Benson M, Brown GR, Chao C, Chitipiralla S, et al. ClinVar: improving access to variant interpretations and supporting evidence. Nucleic Acids Research 2018 Jan;46(D1):D1062–D1067. https://doi.org/10.1093/nar/gkx1153.
33. Geoffroy V, Herenger Y, Kress A, Stoetzel C, Piton A, Dollfus H, et al. AnnotSV: an integrated tool for structural variations annotation. Bioinformatics 2018 Oct;34(20):3572–3574. https://doi.org/10.1093/bioinformatics/bty304.
34. Wojcik MH, Reuter CM, Marwaha S, Mahmoud M, Duyzend MH, Barseghyan H, et al. Beyond the exome: What's next in diagnostic testing for Mendelian conditions. The American Journal of Human Genetics 2023 Aug;110(8):1229–1248. https://www.sciencedirect.com/science/article/pii/S0002929723002100.
35. Merker JD, Wenger AM, Sneddon T, Grove M, Zappala Z, Fresard L, et al. Long-read genome sequencing identifies causal structural variation in a Mendelian disease. Genetics in Medicine 2018 Jan;20(1):159–163. https://www.sciencedirect.com/science/article/pii/S1098360021019328.
36. van Belzen IAEM, Schönhuth A, Kemmeren P, Hehir-Kwa JY. Structural variant detection in cancer genomes: computational challenges and perspectives for precision oncology. npj Precision Oncology 2021 Mar;5(1):1–11. https://www.nature.com/articles/s41698-021-00155-6, publisher: Nature Publishing Group.

37. Duan DM, Cheng C, Huang YS, Chung Ak, Chen PX, Chen YA, et al. Comparisons of performances of structural variants detection algorithms in solitary or combination strategy. PLOS ONE 2025;20(2):e0314982. https://journals.plos.org/plosone/article?id=10.1371/journal.pone.0314982, publisher: Public Library of Science.
38. Liu Z, Roberts R, Mercer TR, Xu J, Sedlazeck FJ, Tong W. Towards accurate and reliable resolution of structural variants for clinical diagnosis. Genome Biology 2022 Mar;23(1):68. https://doi.org/10.1186/s13059-022-02636-8.
39. Wagner J, Olson ND, Harris L, Khan Z, Farek J, Mahmoud M, et al. Benchmarking challenging small variants with linked and long reads. Cell Genomics 2022 May;2(5).
40. Lu H, Giordano F, Ning Z. Oxford Nanopore MinION Sequencing and Genome Assembly. Genomics, Proteomics & Bioinformatics 2016 Oct;14(5):265–279. https://www.sciencedirect.com/science/article/pii/S1672022916301309.
41. Martin S, Heavens D, Lan Y, Horsfield S, Clark MD, Leggett RM. Nanopore adaptive sampling: a tool for enrichment of low abundance species in metagenomic samples. Genome Biology 2022 Jan;23(1):11. https://doi.org/10.1186/s13059-021-02582-x.
42. Oehler JB, Wright H, Stark Z, Mallett AJ, Schmitz U. The application of long-read sequencing in clinical settings. Human Genomics 2023 Aug;17(1):73. https://doi.org/10.1186/s40246-023-00522-3.
43. Rhie A, Nurk S, Cechova M, Hoyt SJ, Taylor DJ, Altemose N, et al. The complete sequence of a human Y chromosome. Nature 2023 Sep;621(7978):344–354. https://www.nature.com/articles/s41586-023-06457-y, publisher: Nature Publishing Group.
44. Nurk S, Koren S, Rhie A, Rautiainen M, Bzikadze AV, Mikheenko A, et al. The complete sequence of a human genome. Science 2022 Apr;376(6588):44–53. https://www.science.org/doi/10.1126/science.abj6987, publisher: American Association for the Advancement of Science.
45. Richards S, Aziz N, Bale S, Bick D, Das S, Gastier-Foster J, et al. Standards and guidelines for the interpretation of sequence variants: a joint consensus recommendation of the American College of Medical Genetics and Genomics and the Association for Molecular Pathology. Genetics in Medicine 2015 May;17(5):405–424. https://www.gimjournal.org/article/S1098-3600%2821%2903031-8/fulltext, publisher: Elsevier.
46. Miller DT, Lee K, Abul-Husn NS, Amendola LM, Brothers K, Chung WK, et al. ACMG SF v3.2 list for reporting of secondary findings in clinical exome and genome sequencing: A policy statement of the American College of Medical Genetics and Genomics (ACMG). Genetics in Medicine 2023 Aug;25(8). https://www.gimjournal.org/article/S1098-3600(23)00879-1/fulltext, publisher: Elsevier.
47. Olivucci G, Iovino E, Innella G, Turchetti D, Pippucci T, Magini P. Long read sequencing on its way to the routine diagnostics of genetic diseases. Frontiers in Genetics 2024 Mar;15. https://www.frontiersin.org/journals/genetics/articles/10.3389/fgene.2024.1374860/full, publisher: Frontiers.
48. Negi S, Stenton SL, Berger SI, Canigiula P, McNulty B, Violich I, et al. Advancing long-read nanopore genome assembly and accurate variant calling for rare disease detection. The American Journal of Human Genetics 2025 Feb;112(2):428–449. https://www.sciencedirect.com/science/article/pii/S0002929725000023.
49. Savara J, Novosád T, Gajdoš P, Kriegová E. Comparison of structural variants detected by optical mapping with long-read next-generation sequencing. Bioinformatics 2021 Oct;37(20):3398–3404. https://doi.org/10.1093/bioinformatics/btab359.

Manuscript

Click here to access/download;Manuscript;25_06_26_Savara_LRS_T2T.pdf

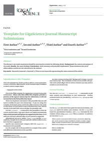

GigaScience, 2023, 1–11

doi: xx.xxxx/xxxx
Manuscript in Preparation
Research

RESEARCH

Multiplatform comparisons and annotation of structural variants highlight the utility of the T2T reference genome in human diagnostics

Jakub Savara[1,2], Tomas Novosad[2], Petr Gajdos[2], Anna Petrackova[1], Marek Behalek[2], Jirina Manakova[1], Filip Ctvrtlik[3], Jiri Minarik[4], Tomas Papajik[4] and Eva Kriegova[1]

[1]Department of Immunology, Faculty of Medicine and Dentistry, Palacký University Olomouc and University Hospital Olomouc, Olomouc, Czech Republic and [2]Department of Computer Science, Faculty of Electrical Engineering and Computer Science, VSB-Technical University of Ostrava, Ostrava, Czech Republic and [3]Department of Radiology, Faculty of Medicine and Dentistry, Palacký University Olomouc and University Hospital Olomouc, Olomouc, Czech Republic and [4]Department of Hemato-oncology, Faculty of Medicine and Dentistry, Palacký University Olomouc and University Hospital Olomouc, Olomouc, Czech Republic

Abstract

Background: Structural variants (SVs) are increasingly recognized as important contributors of human diseases. However, our understanding of SVs in health and disease is limited, mainly due to their structural complexity and variable length between individuals, as well as limitations inherent to the available genomic technologies and used reference genome. **Results:** To systematically evaluate SVs across human whole-genome samples using hg38/GRCh38 and gapless T2T-CHM13 reference, we introduced an innovative multi-platform approach, LongReadChecker (LoReC), which advances SVs comparison and annotation based on distance variance, intersection, gene overlap and the closest SV in the clinical database. Comparison of the performance in detecting SVs from public and own whole-genome datasets from short-read sequencing (SRS), available long-read sequencing (LRS) platforms, and optical genome mapping (OGM) revealed that most SVs detected by SRS were confirmed by LRS, but LRS can identify twice as many SVs (25,000 SVs/genome) with higher read mapping accuracy. Our data further highlights the utility of the T2T-CHM13 reference in SV detection, as 20% more deletions and less 20% insertion are detected compared with hg38/GRCh38, particularly evident in long-read datasets. Since 80% of the SVs detected by LRS/SRS are smaller than 0.5 kbp, OGM did not detect them. **Conclusions:** Our study revealed that introducing of distance variance, intersection, gene overlap and the closest SV in the clinical database may help to compare and annotate SVs in diagnostics. Our data showed that LRS together with T2T-CHM13 gapless sequences can improve the diagnostics of patients with human diseases when SRS fails to identify the cause.

Key words: Next-generation sequencing; structural variants; annotations; LongReadChecker (LoReC) toolkit; long-read technology

Introduction

Structural variants (SVs) are a major source of human genetic diversity that arises from the breakdown and rejoining of DNA fragments, which can lead to loss, gain and rearrangement of genes and regulatory elements [1]. Since SVs are larger than 50 bp and can affect thousands to millions of nucleotides [2, 3], they are expected to have a strong effect on transcriptional regulation in health and disease [4]. SV detection is of particular importance, as they are responsible for more than 25% of all rare protein truncations in a

Compiled on: June 26, 2025.
Draft manuscript prepared by the author.

genome and are associated with many diseases [5, 6]. However, SV characterization and functional interrogation have largely lagged behind single nucleotide variations and small INDELs (insertions and deletions), mainly due to their structural complexity and variable length between individuals, as well as limitations inherent to available genomic technologies [7]. Traditionally, SVs in the human genome have been detected using array-based methods or locus-specific assays for targeted regions [8]. Currently, great progress is being made in the detection of SVs by short-read sequencing (SRS), which remains essential due to practical and cost considerations in clinical diagnostics [9]. Novel genomic technologies for SV detection are also being rapidly developed. Among them, long-read sequencing (LRS) has demonstrated a high potential to detect SVs [10] through longer reads and increased accuracy compared to SRS [1]. The increasing throughput, lower prices and portability of LRS technologies increase the potential of the introduction of LRS into diagnostic testing, particularly for patients with genetic disorders with negative results using SRS [11, 12]. However, accurate and precise identification of SVs in specific samples and/or across samples is challenging [13]. In addition, there are incomplete data on the performance and comparison of currently available genomic technologies, the clinical utility of the novel human reference assembly T2T-CHM13, and clinical databases for annotation of detected SVs. This study therefore focused on a comprehensive comparison of SVs detected from whole-genome datasets, both public and our own, obtained by SRS, LRS from currently available technologies, and optical genome mapping (OGM). For this, we introduced an innovative bioinformatics approach LongReadChecker (LoReC; http://olgen.cz/en/resources) enabling comparisons of SVs across whole-genome datasets, technologies, and reference genomes, including their annotations using clinical genomic databases. This multi-platform approach revolutionizes the comparisons of SVs by introducing key parameters such as distance variance, intersection, and gene overlap between datasets, thus advancing their comparison and annotation across samples or technologies or clinical databases. Our study further highlights the utility of T2T reference and long-read technologies in clinical and research applications.

Materials and methods

Public and our own human whole-genome datasets

This study evaluated whole-genome datasets from a human DNA standard (NA12878 cell line, also known as HG001; B-Lymphocyte; female; healthy; Genome in a Bottle Consortium; [14]) and a breast cancer cell line (SKBR3; [15]) obtained by i) traditional SRS (Illumina), ii) true LRS on a single molecule real-time platform from Pacific Biosciences (LRS-PacBio), iii) true LRS by Oxford Nanopore Technologies (LRS-ONT), iv) synthetic LRS from LRS-TELL-Seq (transposase enzyme-linked long-reads sequencing, Universal Sequencing Technology), v) synthetic LRS from Illumina Complete Long-Reads (LRS-ICLR; Illumina) and vi) synthetic LRS from 10x assay (LRS-10x; 10x Genomics). All of these were synthetic LRS sequenced on an Illumina short-read platform. In addition, OGM from Bionano Genomics was used (Supplementary Table 1). The principles of the LRS technologies used are described elsewhere [16]. Our own datasets from SRS, LRS-TELL-Seq and LRS-ICLR as well as OGM were obtained for two diagnostic samples, P3 (pheochromocytoma adrenal medulla tissue, man, 63 years) and P48 (enriched CD138+ myeloma cells from bone marrow aspirate, woman, 39 years, IgG lambda, stage IIIA, ISS II), as well as for the NA12878 cell line. For our own analysis, the high-molecular-weight DNA from tumour tissues and cells from cell lines was isolated from agarose plugs, as reported previously [17]. The NGS libraries were prepared according to the manufacturer recommendations for LRS-ICLR (Illumina, CA, USA), transposase enzyme-linked long-reads sequencing [TELL-Seq™] (Universal Sequencing technology, MA, USA) and SRS TruSeq DNA PCR-Free (Illumina) and sequenced (150 bp paired-end reads) on NovaSeq 6000 (Illumina). In addition, OGM labelling and measurements using the Bionano Saphyr instrument (Bionano Genomics, CA, USA) were performed as reported previously [17]. The sequence depth for LRS and SRS was approximately 30× and for OGM 300×.

Bioinformatic processing of whole-genome datasets from different short-read sequencing and long-read sequencing platforms

To minimize the difference in precision, recall and F1-score metrics using different callers/aligners, we used the LRS aligner Minimap2 [18] and the SV caller Sniffles2 (v2.2) software [19] for true LRS and the LongRanger software (v2.2.2) [20] for synthetic LRS analyzes, which were found to provide a strong basis for SV pipeline calling in LRS [21]. Moreover, this combination is the basis for the Illumina DRAGEN analysis of Illumina LRS-ICLR, an approach that has also been compared in this study. For SRS datasets, the BWA aligner software (v0.7.17) [22] and the Manta structural variant caller (v1.6.0) [23] were used. The raw data from OGM were analyzed using Bionano Access (v1.8) software by Bionano Genomics, and the De Novo assembly pipeline was performed using Bionano Solve tools (v3.8) (Bionano Genomics).

The hg38 (GRCh38.p14) and T2T-CHM13 (v2.0) human reference genomes were used. The coordinates of genes and pseudogenes, their names, and biotype (e.g. protein-coding) are based on RefSeq NCBI annotations (version 110); in all analyzes, the Y chromosome and ALT contigs were excluded. The list of medically relevant genes is based on the DisGeNET [24] database (DisGeNET v20.1), which includes information on gene/variant disease associations originating from ClinVar, GWAS Catalog, UniProt, GAD and BeFree data [24]. The selection of medically relevant genes is based on gene disease associations (GDA) and variant-disease associations (VDA), GDA/VDA > 0.5 and evidence index > 0.8, indicating that most publications support GDA/VDA [24].

Bioinformatic processing of whole-genome datasets from different short-read sequencing and long-read sequencing platforms

For comparison of SVs across different samples, technologies, and databases, we designed our own LoReC toolkit, containing two tools: the LoReC comparator (source file: variant call format, vcf) and LoReC coverage (source file: mapped reads in binary format, bam).

The LoReC comparator can find the closest SV detected by another technology, database, or reference genome for each SV across the whole genome or region(s) of interest. For each comparison, the following parameters need to be established: 1) the distance variance threshold (the accepted difference in bp between the start and end coordinates between each SV in dataset 1 and the nearest SV in dataset 2, expressed as the sum of the two differences); 2) the intersection factor (the overlap between each SV in dataset 1 and the nearest SV in dataset 2; 0 = 100% overlap, 0–0.5 = partial overlap, >0.5 = no overlap) and 3) the minimal size proportion (the percentage of the size in bp between each SV in dataset 1 and the nearest SV in dataset 2; e.g. 5% means that the SVs in dataset 1 encompass at least 5% of the SVs in dataset 2 or vice versa). To compare SVs in the same regions detected by different technologies, which may differ in size and coordinates because of the principles of the method, the LoReC toolkit lists the closest SV of the same type (e.g. deletion, insertion) from dataset 2 and its coordinates, distance, intersection factor, and size proportion compared with the SV from dataset 1. A distance variance threshold of 1,000 bp was used for a comparison between different NGS platforms and 50,000 bp between NGS and

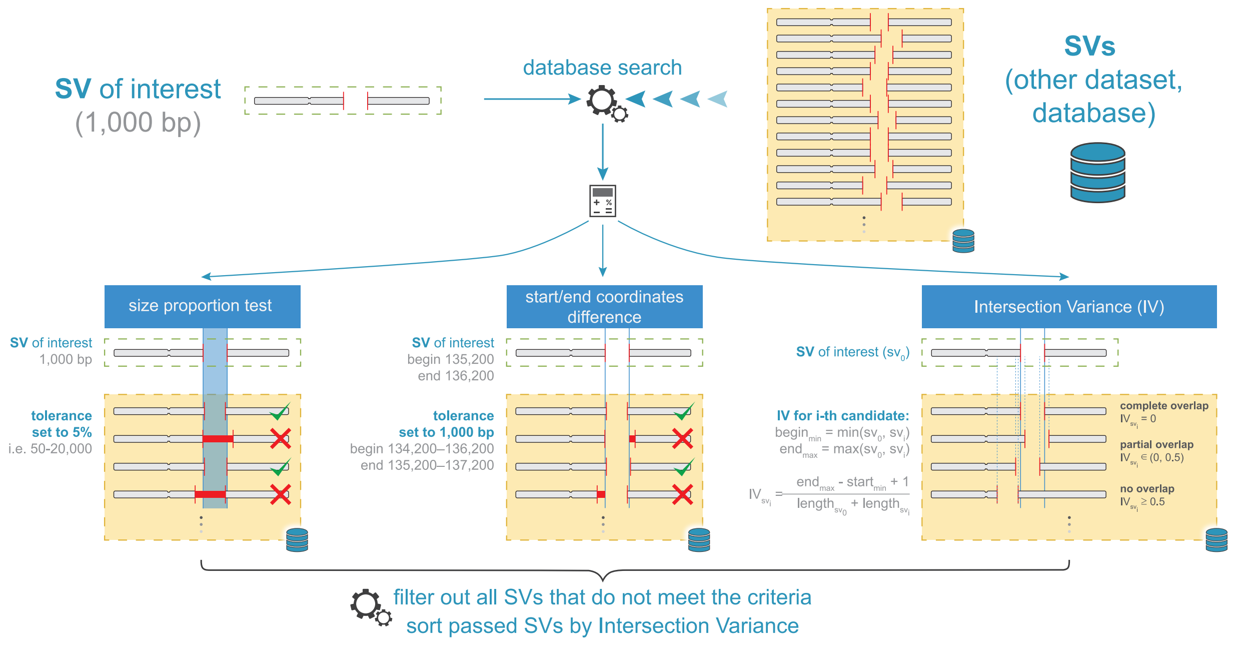

Figure 1. Annotation of structural variants (SVs) detected in the long-read sequencing datasets in clinical diagnostics using the LoReC toolkit. Due to the high variability in SV breakpoints, three measurements are needed for the SV comparison with SVs from clinical databases, such as the distance in bp between the sample SV and the database SV (start and end coordinate difference), the intersection (intersection factor) and the minimum overlap between the sample SV(s) and the database SV(s) (size proportion test).

OGM, and an intersection factor of 0 to 0.5 and a minimum size fraction of 5% were used to indicate SVs that overlap and are very similar; different parameters can be used for filtering (Fig. 1). To visually inspect the SVs of interest, the Samplot tool was used [25].

The LoReC coverage tool is able to provide the coverage of the gene/region of interest, including statistics (mean, median, min, max, Q1 and Q3 coverages), filter reads based on the mapping quality and visualization of the regions of interest. The LoReC coverage outputs are as follows: 1) coverage across regions of interest or across the whole-genome based on the coordinates and gene names given in a region file; 2) coverage calculations based on the mapping quality threshold value, which is able to filter out the reads that map to multiple regions or those of poor quality (MAPQ, MAPQ0 = high probability that a read is mapped to multiple locations with an equal score, MAPQ1 = high probability that a read is mapped to at least two locations, MAPQ50 = 99.999% probability that a read is mapped to a unique region); and 3) visualization of the regions of interest specified in the region files, which allows a comparison of multiple technologies, different samples, or reference genomes. As low coverage genes, those genes that Q1 coverages were below 25% of the mean genome coverage of the sample were marked.

Annotation of structural variants by the LongReadChecker toolkit

Another functionality of the LoReC comparator is to annotate detected SVs based on the annotation file(s) from dbVar (NCBI, https://www.ncbi.nlm.nih.gov/dbvar/; [26]). As the current dbVar_common and ClinVar databases are based on hg38, all SV annotations were performed only on this reference. The following parameters were established for SV comparisons: a threshold of ±1,000 bp distance between the start and end coordinates of the SVs compared, an intersection factor threshold of 0.5 and a minimal size proportion of 5%. First, the detected SVs are compared with the SVs included in the dbVar_common database of common SVs found with a frequency >1% in the population (e.g. nstd186, NCBI Curated Common Structural Variants) or any other vcf file of interest, and must fulfill the set-up criteria to be marked as PASS. Second, the detected SVs not found in the dbVar_common database are compared with the ClinVar SV database (e.g. summary of nstd102, Clinical Structural Variants) or variant file format (VCF) of interest, and for SVs present in the database, the clinical significance of SVs is reported (e.g. pathogenetic, likely pathogenic, VUS, likely benign, benign). For SVs not found in the ClinVar SV database and with an overlap of at least one gene, additional information is reported through the LoReC toolkit based on the NCBI annotation file, which includes the biotype, gene description, and gene/variant disease associations present in DisGeNET.

Results

True long-read sequencing technologies have superior performance in detecting structural variants

To evaluate the performance of the currently available LRS technologies and SRS, whole-genome sequencing datasets for NA12878 healthy [14] and SKBR3 breast cancer [15] cell lines and two diagnostic tissue samples, P3 (pheochromocytoma tissue) and P48 (multiple myeloma bone marrow aspirate), both public and our own (Supplementary Table 1), were compared using different LRS and SRS platforms. Among the tested platforms were the following: 1) two true LRS technologies obtained from Pacific Biosciences (LRS-PacBio) and Oxford Nanopore Technologies (LRS-ONT) instruments; 2) three synthetic linked-read LRS approaches (Illumina Complete Long-Reads (LRS-ICLR), 10x Genomics (LRS-10x), and Universal Sequencing Technology (LRS-TELL-Seq)), sequenced on the short-read Illumina platform; 3) SRS on Illumina; and 4) OGM from Bionano Genomics. For comparisons of detected SVs, their types, coordinates, sizes, intersection factors, size proportions, and coverage between different technologies, reference genomes (hg38/GRCh38.p14; T2T-CHM13, v2.0) and annotations of detected SVs according to clinical databases, a LoReC toolkit was developed (Fig. 1). For more details on functionalities, see Materials and methods section. Using hg38, approximately 25,000 SVs per genome were detected using LRS-PacBio and LRS-ONT, 14,000–15,000 us-

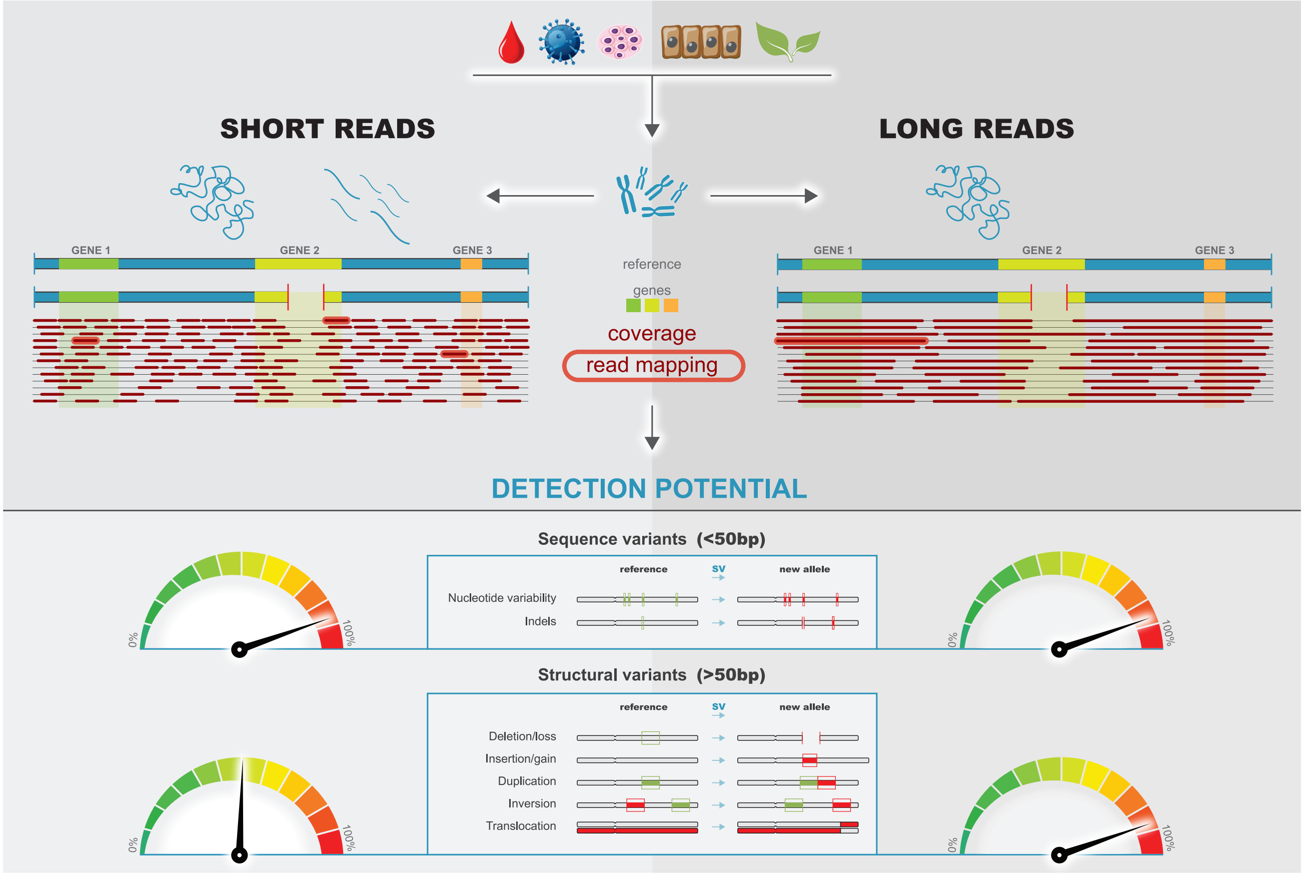

Figure 2. Principle of short-read sequencing (SRS) and long-read sequencing (LRS) and their detection potential for sequence and structural variants. Example of the coverage and read mapping for a heterozygous gene deletion is shown; LRS requires high-molecular-weight DNA than SRS. The lower part shows the detection potential for sequence and structural variants by SRS and LRS.

ing LRS-ICLR, 10,000–12,500 using LRS-10x, 12,500–15,000 using SRS and 4,000 using OGM. Insertions were the most common SVs detected by all technologies: 14,000 per genome using LRS-PacBio and LRS-ONT, 5,000 to 6,000 using LRS-ICLR, 6,000 to 7,000 using SRS and 2,500 using OGM. Insertions were not detected in the LRS-TELL-Seq and LRS-10x datasets, as the LongRanger pipeline available to analyze synthetic reads cannot call insertions. Deletions were the second most common SVs: 10,500 per genome using LRS-PacBio and LRS-ONT, 9,000 to 10,000 using LRS-ICLR, 3,500 to 5,000 using LRS-10x, 5,000 to 6,000 using SRS and 1,250 using OGM. Regarding other SVs such as inversions and duplications, breakends/translocations, their counts varied across samples and genomes, ranging from 240 to 1,430 per genome, depending on the technology (Table 1, Fig. 3, Supplementary Fig. 1). Selected SVs from the OGM and LRS datasets were verified using fluorescence immunophenotyping and interphase cytogenetics as a tool for the investigation of neoplasms (FICTION), arrayCGH and/or targeted SRS.

When comparing available technologies, the majority of deletions and insertions (~80%–95%) detected by LRS-ONT were confirmed by LRS-PacBio; however, less than 50% of them were detected by SRS and 55%–71% by LRS-ICLR. Furthermore, LRS-TELL-Seq and LRS-10x confirmed approximately 25%–50% of the deletions detected by LRS-ONT/LRS-PacBio, but it was not possible to call insertions from synthetic reads. Regarding inversions, duplications, and breakends/translocations, the best overlap was observed between LRS-PacBio and LRS-ONT, and a moderate overlap was identified using synthetic LRS and SRS (Supplementary Tables 2, 3).

Most structural variants detected by short-read sequencing were confirmed by long-read sequencing

Next, we compared the SVs detected by SRS, the most widely used platform today in clinical diagnostics, with SVs detected by LRS-ONT and LRS-PacBio and synthetic LRS. Most of the deletions and insertions detected by SRS (10,000 to 12,500 per genome) were smaller than 0.5 kbp (~80%) and were confirmed by LRS-PacBio/LRS-ONT (~90%). LRS-ICLR was similar to true LRS in detecting deletions, but has a lower precision in the case of insertions (~50%) as this technology is based on SRS. Notably, SRS did not detect approximately 50% of the SVs detected by LRS (Supplementary Tables 2, 4).

True long-read sequencing and short-read sequencing technologies have superior performance over optical genome mapping

We also compared LRS and SRS with OGM, a non-sequencing technology based on the labeling of high-molecular weight DNA using fluorophore tags on specific sequence motifs. As OGM does not detect SVs smaller than 0.5 kbp or SVs in genomic regions that lack specific sequence motifs, OGM detected a lower number of SVs than LRS or SRS (hg38: 3,946 vs 24,941 vs 12,953; T2T-CHM13: 3,082 vs 24,516 vs 6,769). The majority (~80%) of deletions and insertions detected by OGM were confirmed by LRS-ONT and LRS-PacBio; SRS confirmed ~35% of the insertions and ~50%–60% of the deletions detected by OGM (Supplementary Tables 2, 5). Comparison of OGM with LRS and SRS showed that OGM does not provide the exact coordinates of individual SVs and their position may be significantly different compared to coordinates detected by LRS and

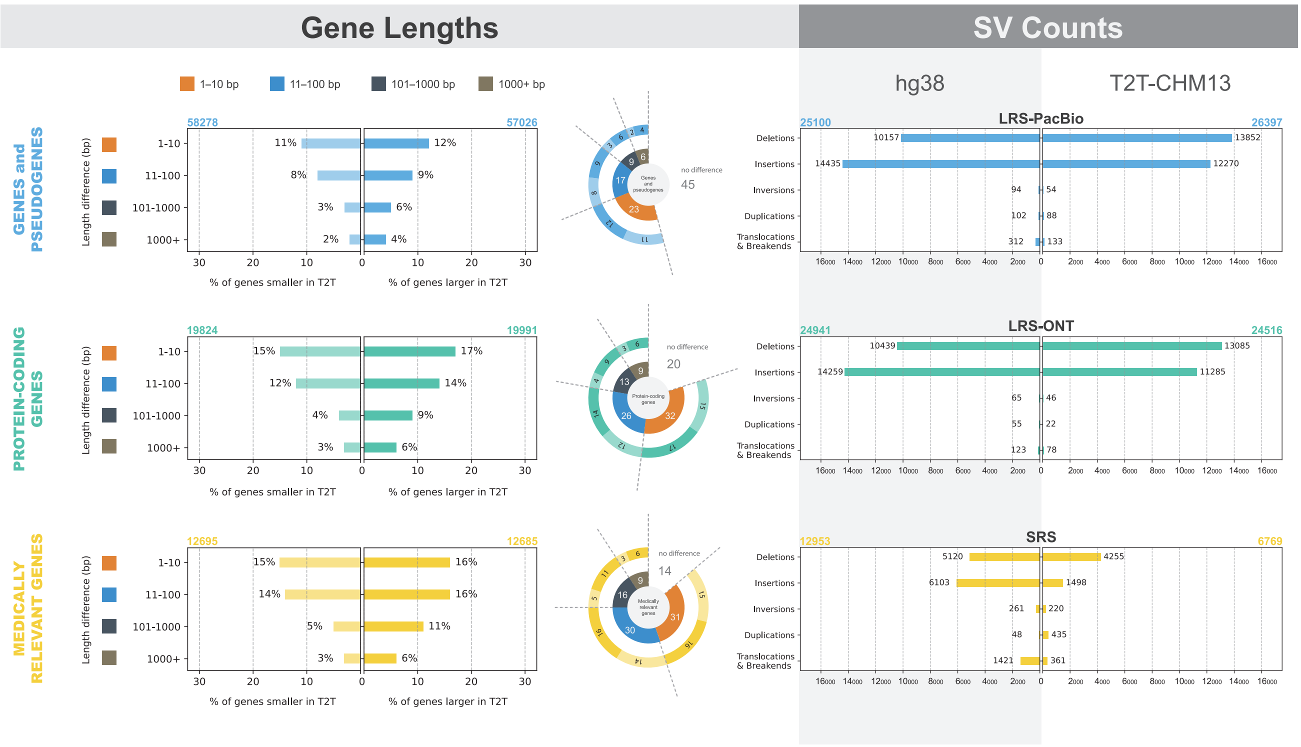

Figure 3. Comparison of gene lengths and the number of structural variants (SVs) in the NA12878 cell line using hg38 and T2T-CHM13 references. The differences in the number and lengths of genes and pseudogenes, protein-coding genes and medically relevant genes for both references are presented; genes were annotated based on RefSeq NCBI (version 110, based on gene IDs), excluding genes on the Y chromosome and ALT contigs. The number of SVs detected by long-read sequencing (LRS-PacBio, LRS-ONT) and short-read sequencing (SRS) on both references are shown on the right part.

SRS (Supplementary Fig. 2).

True long-reads map with high probability to unique regions compared with short-reads

Next, we evaluated the read mapping quality (MAPQ) [22], a measure of the probability that a read is misplaced, for different LRS and SRS technologies. For MAPQ0, a default setting in current SRS/LRS aligners and variant callers that allows read mapping to multiple regions, most genes were covered for all SRS/LRS technologies (Fig. 2, Supplementary Fig. 3, Supplementary Table 6). To eliminate problematic regions with misplaced reads, current SRS pipelines often mask these repetitive 'dark regions'. When stricter MAPQ1 and MAPQ50 associated with a lower probability of misplaced reads compared to MAPQ0 were applied, many regions of the genome were not covered in the SRS datasets, including many protein-coding genes and medically relevant genes (Fig. 2, Supplementary Fig. 3, Supplementary Table 6). In LRS-PacBio high-fidelity (HiFi) datasets, most reads were mapped with high probability to a unique region, as demonstrated by applying MAPQ1/MAPQ50. Regarding LRS-ONT, mapping to multiple regions is less probable because of the very long-reads (up to Mbp); however, many reads are of low quality, resulting in less accuracy in mapping (Fig. 2, Supplementary Fig. 3, Supplementary Table 6).

T2T-CHM13 reference improves the analysis of structural variants for long-read sequencing and short-read sequencing datasets

To understand the added value of the gapless T2T-CHM13 reference with the currently used hg38, SVs in known genes and pseudogenes, protein-coding genes, and medically relevant genes and their lengths were compared for the LRS and SRS datasets, excluding the Y chromosome and alternate (ALT) contigs (Fig. 3, Supplementary Table 6). Using the T2T-CHM13 reference, the number of deletions increased by more than 20%, and the number of insertions decreased by more than 20% using the LRS-ONT and LRS-PacBio datasets compared to hg38 (Fig. 3, Supplementary Fig. 1, Table 1). Using SRS, approximately 10% more deletions and 80% fewer insertions were detected using T2T-CHM13 than using hg38 (Fig. 3, Supplementary Fig. 1, Table 1). In the unique regions of T2T-CHM13, numerous translocations, deletions, and other SVs were detected in the telomere, centromere, and subcentromere regions in all analyzed datasets (Supplementary Tables 3-5, Supplementary Fig. 4). The T2T-CHM13 reference also refined the length of the genes. Comparing T2T-CHM13 and hg38 references for 54,553 genes and pseudogenes that overlap (gene IDs in RefSeq NCBI annotations, version 110) (Fig. 3, Supplementary Table 6), 45.6% had the same length, 23.7% differed by 1–10 bp, 16.8% by 11–100 bp, 8.2% by 101–1,000 bp and 5.6% by more than 1,000 bp. Regarding protein-coding genes (medically relevant genes), 19.9% (14.3%) had the same length, 31.7% (31.0%) differed by 1–10 bp, 26.7% (30.2%) by 11–100 bp, 13.3% (15.8%) by 101–1,000 bp and 8.4% (8.8%) by more than 1,000 bp (Fig. 3). Among those with the largest differences in length were *GRK1* [27] and *LPA* [28], many other genes like *SMN1&2* [29], *DUX4* and *HLA-DRB5* or gene *GBA* and its pseudogene *GBAP1*, were found disassembled, not correctly assembled or highly similar in hg38 (Supplementary Fig. 5, Supplementary Fig. 6, Supplementary Table 6). Furthermore, 167 more protein-coding genes were annotated in T2T-CHM13 than in hg38 (Supplementary Table 6). To complement the added value of T2T-CHM13, we added an annotation about genes found in the discrepant regions in hg19 and hg38 [30, 31] (Supplementary Table 6).

Table 1. Structural variants detected in the NA12878 and SKBR3 cell lines and the P3 and S48 diagnostic samples using SRS and different LRS technologies for the hg38 and T2T-CHM13 human references.

Sample	SV type	Total number of SVs		Deletions		Insertions		Duplications		Inversions		Breakends/ Translocations	
	References/ Technology	hg38	T2T-CHM13	hg38	T2T-CHM13	hg38	T2T-CHM13	hg38	T2T-CHM13	hg38	T2T-CHM13	hg38	T2T-CHM13
NA12878	SRS	12,912	6,769	5,120	4,255	6,103	1,498	48	435	261	220	1,421	361
	LRS-PacBio	24,811	26,397	10,157	13,852	14,435	12,270	102	88	94	54	312	133
	LRS-ONT	24,793	24,516	10,439	13,085	14,259	11,285	55	22	65	46	123	78
	LRS-ICLR	15,410	NA	9,412	NA	5,872	NA	28	NA	80	NA	38	NA
	LRS-10x	11,800	15,120	3,542	7,989	ND	ND	849	780	6,623	5,867	786	484
	LRS-TELL-Seq	8,543	10,873	4,964	6,096	ND	ND	54	162	3,432	4,514	93	101
	OGM	3,947	3,082	1,283	1,414	2,556	1,523	44	38	64	42	0	65
SKBR3	SRS*	5,324	4,011	2,920	2,738	937	598	937	238	258	212	272	225
	LRS-PacBio	32,850	32,533	9,097	11,321	22,994	20,630	154	120	235	207	370	255
	LRS-ONT	23,597	21,291	10,983	12,200	12,221	8,749	49	39	170	152	174	151
	LRS-10x	10,319	12,140	4,918	3,393	ND	ND	1,318	6,830	3,393	1,300	690	617
	OGM	4,030	3,007	1,283	1,413	2,403	1,365	64	60	98	66	182	103
P3	SRS	12,823	6,441	5,018	4,079	5,993	1,418	54	444	246	196	1,512	304
	LRS-ICLR	14,513	NA	9,166	NA	5,228	NA	28	NA	60	NA	31	NA
	LRS-TELL-Seq	8,434	9,171	4,582	5,385	ND	ND	193	153	3,553	3,524	106	109
	OGM	4,076	3,130	1,262	1,335	2,638	1,625	84	64	71	34	21	72
S48	SRS	14,846	8,386	5,632	4,900	6,862	2,089	72	554	284	256	1,996	587
	LRS-ICLR	15,342	NA	9,279	NA	5,951	NA	24	NA	62	NA	26	NA
	LRS-TELL-Seq	9,960	10,888	5,281	6,109	ND	ND	153	164	4,436	4,514	90	101
	OGM	3,796	2,877	1,225	1,326	2,432	1,421	55	44	72	46	12	40

Legend: SRS, short-read sequencing by Illumina platform; LRS-PacBio, true long-read sequencing by Pacific Biosciences; LRS-ONT, true long-read sequencing by Oxford Nanopore Technologies; LRS-ICLR, synthetic long-read sequencing by Illumina - complete long-reads technology on Illumina platform; LRS-TELL-Seq, synthetic long-read sequencing by Universal Sequencing Technology on Illumina platform; LRS-10x, synthetic long-read sequencing by 10x Genomics on Illumina platform; SVs, structural variants; OGM, optical genome mapping by Bionano Genomics; NA, not available; ND, not detected. *dataset with average coverage 20× (less than in other datasets, where 30× coverage was achieved).

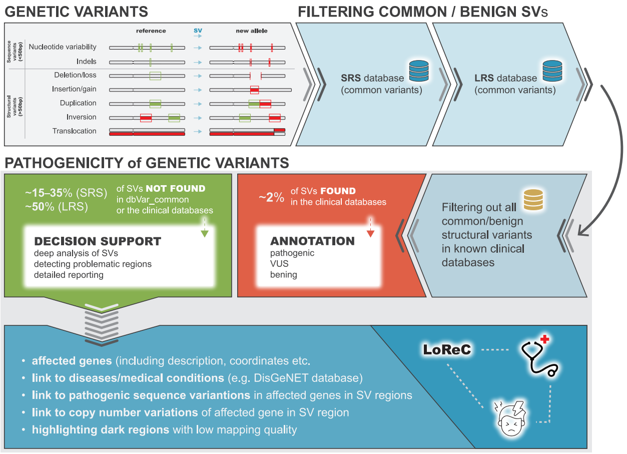

Figure 4. Suggested workflow for annotation of structural variants (SVs) detected by long-read sequencing (LRS) in diagnostic samples. First, SVs in the samples are filtered using a dbVar_common database or other to filter out the common and benign SVs. The SVs not found in the dbVar_common database are compared with the ClinVar SV database or similar. For SVs not found in the ClinVar database, the LoReC toolkit is used for the comparison and annotation of SVs in the samples based on the NCBI annotation file(s), including affected gene (gene description, coordinates) and link to the disease or medical condition based on the gene/variant disease associations present in DisGeNET or similar.

Annotation of structural variants in clinical databases is challenging

To annotate the SVs detected by tested technologies, we used a LoReC comparator toolkit to compare the detected SVs with the dbVar_common (NCBI dbVar Curated Common Structural Variants, GnomAD and other sources, ID: nstd186) [26] and ClinVar (ID: nstd102) clinical datasets [32]. DbVar_common database includes those SVs that occur with a frequency >1% in the population. As the current ClinVar database is based on hg38, all annotations of SVs were performed using this reference. Given that SV coordinates may vary from individual to individual, the following parameters need to be defined prior to each analysis: 1) the distance variance threshold (acceptable difference in bp between the SV of interest and the SV in the clinical database), 2) the intersection factor (overlap between the SV of interest and the nearest SV in the clinical database) and 3) the minimum size fraction (minimum proportion of the SV of interest and the nearest SV in the clinical database) (see the Methods for details). All SVs were also evaluated using the AnnotSV tool [33] (Table 2, Fig. 3). Regarding deletions, approximately 65% of the deletions detected by the SRS datasets overlapped with the deletions in dbVar_common and approximately 2% with those in ClinVar. When using AnnotSV, 83% of the SVs in the SRS datasets were annotated as benign, approximately 1% (30–90) deletions per sample were annotated as pathogenic/likely pathogenic and less than 18% as a variant of unknown significance (VUS). Using true LRS, approximately 50% of deletions were detected in dbVar_common and 2% in ClinVar; for more than half of deletions, no annotation was available (Table 2). Using ClinVar annotations, the majority of SVs in this database span very large regions containing many genes; therefore, they do not meet the size proportion criteria of the PASS filters for SVs detected by LRS. When using AnnotSV, 50%–60% of SVs in the LRS datasets were annotated as benign, approximately 10 deletions (~0.1%) were annotated as pathogenic/likely pathogenic and approximately 4,500 deletions (30%–50%) as VUS, depending on the sample and technology used (Table 2). Regarding insertions, the dbVar_common/ClinVar databases contain only breakpoints and lack information on insertion length for most insertions, making annotation impossible, which is also problematic for inversions and translocations.

Table 2. Annotations of deletions detected in whole-genome datasets for the NA12878 and SKBR3 cell lines and P3 and S48 diagnostic samples by SRS and LRS technologies employing A) LoReC using dbVar_common and ClinVar and B) AnnotSV, both for the hg38 reference.

Sample	Technology	TOTAL DEL	dbVar_common	ClinVar	Pathogenic/ Likely Pathogenic	VUS	Benign/ Likely Benign	Not annotated	TOTAL DEL+BND	Benign	Pathogenic/ Likely Pathogenic	VUS (Not annotated)
	ALL		A) LoReC						B) AnnotSV			
NA12878	SRS	5,120	3,332 (65%)	135 (3%)	0/1	12	121/0	1,754 (34%)	5,525	4,598 (83%)	32/6	889 (16%)
	LRS-PacBio	10,157	4,588 (45%)	221 (2%)	0/4	17	198/1	5,466 (54%)	10,157	6,030 (59%)	7/6	4,114 (41%)
	LRS-ONT	10,439	4,580 (44%)	221 (2%)	0/5	17	196/2	5,767 (55%)	10,439	5,893 (56%)	2/7	4,537 (43%)
	LRS-ICLR	9,412	3,978 (42%)	195 (2%)	1/3	14	175/1	5,372 (57%)	9,412	4,767 (51%)	2/4	4,639 (49%)
SKBR3	SRS	2,920	2,239 (77%)	101 (4%)	1/0	5	92/0	597 (20%)	2,890	2,373 (82%)	46/32	457 (16%)
	LRS-PacBio	9,097	4,098 (45%)	196 (2%)	1/4	10	175/3	4,880 (54%)	9,097	6,095 (67%)	30/10	2,962 (33%)
	LRS-ONT	10,983	4,236 (39%)	190 (2%)	3/4	10	169/1	6,634 (60%)	10,983	7,183 (65%)	29/15	3,756 (34%)
P3	SRS	5,018	3,221 (64%)	128 (3%)	0/0	13	113/1	1,771 (35%)	5,478	4,463 (81%)	36/4	975 (18%)
	LRS-ICLR	9,166	3,856 (42%)	172 (2%)	0/3	17	151/0	5,258 (57%)	9,166	4,486 (49%)	0/3	4,677 (51%)
S48	SRS	5,632	3,469 (62%)	129 (2%)	0/0	12	116/0	2,115 (38%)	6,199	5,035 (81%)	84/6	1,074 (17%)
	LRS-ICLR	9,279	3,918 (42%)	144 (2%)	0/3	14	156/3	5,280 (57%)	9,279	4,715 (51%)	5/6	4,553 (49%)

Legend: SRS, short-read sequencing by Illumina platform; LRS-PacBio, true long-read sequencing by Pacific Biosciences; LRS-ONT, true long-read sequencing by Oxford Nanopore Technologies; LRS-ICLR, synthetic long-read sequencing by Illumina - complete long-reads technology on Illumina platform; LRS-TELL-Seq, synthetic long-read sequencing by Universal Sequencing Technology on Illumina platform; LRS-10x, synthetic long-read sequencing by 10x Genomics on Illumina platform; OGM, optical genome mapping by Bionano Genomics; VUS, variant of unknown significance; DEL, deletion; BND, Breakends.

Annotation of the structural variants not presented in the clinical databases by LoReC

We suggested a workflow for diagnostic laboratories to annotate SVs from LRS/SRS. Briefly, after filtering out benign/common SVs present in the databases (nowadays mainly based on SRS, but in the future LRS datasets will be added), the remaining SVs will be compared with clinical databases ClinVar or similar databases (Fig. 4). For SVs not presented in the dbVar_common/ClinVar databases, our LoReC toolkit enables the addition of custom annotations for regions/genes within the detected SVs. In particular, genes may be linked to the gene/variant disease associations provided in the annotation file (e.g. DisGeNET) or other custom annotations (Supplementary Table 6). In addition, the LoReC toolkit provides coverage for genes and regions, mapping quality, and enables the visualization of the regions of interest for expert evaluation.

Discussion

The field of SV importance in clinical diagnostics continues to expand due to advances in genomic technologies and wet lab protocols, the introduction of gap-less reference sequence T2T-CHM13, and the release of high-quality datasets. Despite the growing importance of SVs in human diseases, our knowledge of SVs in health and disease is limited, largely due to their structural complexity and variable length in different individuals, as well as the limitations inherent in available genomic technologies. Here, we comprehensively evaluated SVs from human whole-genome datasets obtained from SRS, all available LRS and OGM platforms across different samples, technologies and clinical databases using two human references (hg38 and T2T-CHM13). Moreover, our multi-platform approach enables annotation of detected SVs to support the implementation of SV diagnostics in clinical practice.

Despite advances in clinical genetic diagnostics, approximately 50% of all suspected Mendelian diseases and many sporadic development syndromes and chronic diseases remain unresolved [34, 35]. Diagnostics of SVs is relevant not only in rare genetic diseases, but SVs are also important contributors to chronic diseases, including cancer [5, 6, 36]. However, the technical limitations inherent in the available genomic technologies, as well as the structural complexity of SVs and their variable length in different individuals, have led to an incomplete characterization of SVs in the human genome in health and disease compared to single nucleotide variations and small INDELs. To gain more insights into the performance of available genomic technologies, we compared our own and public whole-genome datasets from SRS, two LRS platforms (PacBio-LRS, ONT-LRS) requiring specific instruments, and three LRS approaches utilizing synthetic long-reads sequenced on conventional short-read next-generation sequencers (NGS). For this, we developed the multi-platform LoReC toolkit, which compares the size and type of SVs, their overlap, coverage, coordinates, affected genes and disease associations from different SRS and LRS datasets in specific samples and/or across samples and databases, regardless of which of the many available algorithms was used to detect SVs [37, 38].

First, we were interested in the performance of SRS, as this gold standard method remains essential in diagnostics due to practical and cost considerations, compared to third-generation technologies. On average, approximately 13,000 SVs/genome were detected by SRS and twice as many (~25,000 SVs/genome) by LRS. Notably, most of the SVs detected by SRS were also detected by LRS. Our findings are consistent with others showing that LRS can identify hidden disease-related SVs that are not detected by SRS [3, 31, 39]. Since 80% of the SVs detected by LRS/SRS were smaller than 0.5 kbp, OGM did not detect most of the SVs detected by LRS/SRS. The most common type of SVs were deletions and insertions, whereas half as many deletions (~5,000 vs ~10,000) and insertions (~6,000 vs more than 12,000) per sample were detected by SRS compared with LRS. Our data reveal that LRS-ONT and LRS-PacBio technologies have superior performance in detecting SVs to the SRS, the synthetic read LRS-ICLR, LRS-10x, LRS-TELL-Seq, and OGM.

In addition to detecting more SVs in LRS, longer reads map more uniquely to the genome than SRS. Despite using the default MAPQ0 settings employed in the current SRS/LRS aligners and variant callers that allow the use of misplaced reads, effective coverage of most genes across genomes was achieved for all SRS/LRS technologies. To address this issue, current SRS pipelines often mask these problematic ‘dark regions’, which include repetitive elements and polymorphic regions, potentially leading to the loss of key information in these regions. In addition, PacBio provides a BED file for ’dark regions’ that occur in repetitive areas or areas with high GC content. When stricter MAPQ1 or MAPQ50 were applied, many regions across the genome were not covered well in the SRS datasets, including numerous protein-coding genes and medically relevant genes. Of the technologies analyzed, the lowest probability of mismatches was observed for the data obtained by LRS-PacBio HiFi, the highest for SRS. The LRS-ONT datasets demonstrated a high percentage of low-quality reads due to lower nucleotide accuracy when using the MAPQ0 setting. When using MAPQ1 or MAPQ50 to filter out low-quality reads, uniform coverage across the whole genome with high-quality reads and very long-reads generated was achieved by LRS-ONT. Notably, LRS-ONT has introduced adaptive sampling, a computational enrichment technique that adjusts the sequencing parameters in the regions of interest [40, 41], enabling deeper coverage in these regions [42].

Another key step for clinical genetics is the introduction of gap-less T2T-CHM13 human reference assembly, uncovering 8% of the dark regions of the genome, adds nearly 200 million bases, and predicts 99 novel protein coding genes compared to reference hg38 [43]. Our comprehensive bioinformatic analysis of whole-genome datasets further supports the clinical utility of using the T2T-CHM13 sequence for medical diagnostics. When we compared medically relevant genes using coordinates based on NCBI RefSeq annotations on both references, approximately 85% of the genes changed size and more than half by more than 10 bp. Notably, approximately 9% of the medically relevant genes differed by >1,000 bp between hg38 and T2T-CHM13. Among the genes significantly differing between both references were those that were disassembled, not correctly assembled, or highly similar in hg38, such as the challenging medically relevant genes *GRK1*, *LPA*, *SMN1&2*, *DUX4* and *HLA-DRB5* or the *GBA* gene and its pseudogene *GBAP1*, etc. [44]. In addition, the novel T2T-CHM13 reference resulted in approximately 20% more deletions and 20% fewer insertions than hg38. Taken together, the gene length and SV changes may reflect the repetitive nature and complexity of some medically relevant genes, further highlighting the advantage of LRS and the T2T-CHM13 reference for clinical medicine.

Another challenging topic is the pathogenicity annotations of thousands of SVs detected by LRS/SRS. Unlike sequence variants, for whose interpretation there are standards and guidelines [45, 46] and clinical databases such as dbVar_common/ClinVar based on large available SRS datasets, SV annotations are more difficult not only due to the insufficient number of LRS datasets in the databases, but also due to the significant variability in SV break points between individuals and the complexity of rearrangements. Therefore, we introduced crucial measures for SV evaluations, such as the distance in bp between the sample SVs and the datasets/references/databases SVs, the intersection and minimum overlap of the sample SVs with the datasets/references/databases SVs. When we annotated deletions in LRS datasets using dbVar_common/ClinVar or AnnotSV [33], approximately 45% of deletions were detected in dbVar_common and less than 2% were annotated in ClinVar. Notably, for approximately 5,500 deletions (56%) per genome, many of them in protein-coding sequences, no annotation was available in the current version of the ClinVar database. Moreover, the majority of

deletions in the ClinVar database span very large regions containing many genes; therefore, they do not meet the size proportion criteria of the PASS filters for SVs detected by LRS. Regarding insertions and other SVs, the length of the insertion and sequence of the insert are missing in the dbVar_common/ClinVar databases, making their annotation impossible. For SVs not annotated by ClinVar, we applied the LoReC toolkit, and, with its help, the affected gene(s) can be linked with gene/variant disease associations using DisGeNET, human phenotype ontology, or a similar database. In the future, this approach will allow SV annotations to be matched to continuously updated clinical databases based on the LRS datasets and linked to the reference T2T-CHM13.

This study and other studies [11, 12, 39, 42, 47, 48, 49] further support the introduction of LRS into medical diagnostics in the near future, especially in cases with negative SRS results. To introduce LRS into diagnostics, laboratories should collect cells for high molecular weight DNA isolation, as LRS cannot be performed on fragmented DNA obtained by standard isolation methods and increases computational and data storage capacity due to the large datasets obtained by LRS. We highlight the introduction of distance variance, intersection, gene overlap and the closest SV in the clinical database for SVs comparisons and annotations, which is currently the weakest point of SV implementation into clinical diagnostics. Although this first comprehensive study on the performance of all available genomic technologies is focused on SVs in the human genome, SV events are widespread in other species, and our toolkit is suitable for these datasets as well.

Conclusions

In this study, we introduced innovative multi-platform approach for any SRS and third-generation dataset that advance SV comparisons across the samples and databases as well as annotations of SVs based on comparisons with the clinical databases. Despite that gold standard SRS may uncover thousands of SVs that may be clinically relevant, we showed that LRS is more effective at detecting SVs than SRS. Thus, LRS is expected to complement SRS analysis of negative samples in clinical diagnostics soon as the identification of novel genetic biomarkers can be used to individually tailor the prevention and treatment not only in chronic diseases. However, the implementation of LRS will also require the introduction of isolation methods leading to high molecular weight DNA and the update of clinical databases to include LRS datasets and the T2T-CHM13 reference for correct annotations of SVs.

Declarations

Ethical Approval

The patients provided written informed consent for the use of their biological materials for the purpose of this study, which was conducted in accordance with the Helsinki Declaration and approved by the local ethics committee (NW25-03-00391).

Consent for publication

Not applicable

Competing Interests

The authors have no competing interests to declare.

Funding

This study was supported by the Internal Grant Agency of Palacký University (JG_2025_035, IGA_LF_2025_014), NW24-10-00395 and, in part, by the Ministry of Health of the Czech Republic (MH CZ – DRO (FNOL, 00098892).

Author's Contributions

JS and EK designed the research; TN and JS developed the LoReC toolkit; JS, TN, PG and MB performed the bioinformatic analysis; JM and AP performed the laboratory experiments; MM, TP and FC collected the patient samples and clinical characteristics; JS and EK wrote the manuscript; and PG and AP critically revised the manuscript. All authors read and approved the final manuscript.

Code availability

Code is available at: https://github.com/novosadt/lorec-comparator.git and https://github.com/novosadt/lorec-coverage.git.

References

1. Collins RL, Talkowski ME. Diversity and consequences of structural variation in the human genome. Nature Reviews Genetics 2025 Jan;https://www.nature.com/articles/s41576-024-00808-9.
2. Logsdon GA, Vollger MR, Eichler EE. Long-read human genome sequencing and its applications. Nature Reviews Genetics 2020 Oct;21(10):597–614. https://www.nature.com/articles/s41576-020-0236-x, publisher: Nature Publishing Group.
3. Mahmoud M, Huang Y, Garimella K, Audano PA, Wan W, Prasad N, et al. Utility of long-read sequencing for All of Us. Nature Communications 2024 Jan;15(1):837. https://www.nature.com/articles/s41467-024-44804-3, publisher: Nature Publishing Group.
4. Sudmant PH, Rausch T, Gardner EJ, Handsaker RE, Abyzov A, Huddleston J, et al. An integrated map of structural variation in 2,504 human genomes. Nature 2015 Oct;526(7571):75–81. https://www.nature.com/articles/nature15394, publisher: Nature Publishing Group.
5. DeBoever C, Tanigawa Y, Lindholm ME, McInnes G, Lavertu A, Ingelsson E, et al. Medical relevance of protein-truncating variants across 337,205 individuals in the UK Biobank study. Nature Communications 2018 Apr;9(1):1612. https://www.nature.com/articles/s41467-018-03910-9, publisher: Nature Publishing Group.
6. Collins RL, Brand H, Karczewski KJ, Zhao X, Alföldi J, Francioli LC, et al. A structural variation reference for medical and population genetics. Nature 2020 May;581(7809):444–451. https://www.nature.com/articles/s41586-020-2287-8, publisher: Nature Publishing Group.
7. Kosugi S, Momozawa Y, Liu X, Terao C, Kubo M, Kamatani Y. Comprehensive evaluation of structural variation detection algorithms for whole genome sequencing. Genome Biology 2019 Jun;20(1):117. https://doi.org/10.1186/s13059-019-1720-5.
8. Escaramís G, Docampo E, Rabionet R. A decade of structural variants: description, history and methods to detect structural variation. Briefings in Functional Genomics 2015 Sep;14(5):305–314. https://doi.org/10.1093/bfgp/elv014.
9. Ahsan MU, Liu Q, Perdomo JE, Fang L, Wang K. A survey of algorithms for the detection of genomic structural variants from long-read sequencing data. Nature Methods 2023

Aug;20(8):1143–1158. https://www.nature.com/articles/s41592-023-01932-w, publisher: Nature Publishing Group.

10. Amarasinghe SL, Su S, Dong X, Zappia L, Ritchie ME, Gouil Q. Opportunities and challenges in long-read sequencing data analysis. Genome Biology 2020 Feb;21(1):30. https://doi.org/10.1186/s13059-020-1935-5.
11. Mantere T, Kersten S, Hoischen A. Long-Read Sequencing Emerging in Medical Genetics. Frontiers in Genetics 2019 May;10. https://www.frontiersin.org/journals/genetics/articles/10.3389/fgene.2019.00426/full, publisher: Frontiers.
12. Sanford Kobayashi E, Batalov S, Wenger AM, Lambert C, Dhillon H, Hall RJ, et al. Approaches to long-read sequencing in a clinical setting to improve diagnostic rate. Scientific Reports 2022 Oct;12(1):16945. https://www.nature.com/articles/s41598-022-20113-x, publisher: Nature Publishing Group.
13. Mahmoud M, Gobet N, Cruz-Dávalos DI, Mounier N, Dessimoz C, Sedlazeck FJ. Structural variant calling: the long and the short of it. Genome Biology 2019 Nov;20(1):246. https://doi.org/10.1186/s13059-019-1828-7.
14. Zook JM, Catoe D, McDaniel J, Vang L, Spies N, Sidow A, et al. Extensive sequencing of seven human genomes to characterize benchmark reference materials. Scientific Data 2016 Jun;3(1):160025. https://www.nature.com/articles/sdata201625, publisher: Nature Publishing Group.
15. Aganezov S, Goodwin S, Sherman RM, Sedlazeck FJ, Arun G, Bhatia S, et al. Comprehensive analysis of structural variants in breast cancer genomes using single-molecule sequencing. Genome Research 2020 Sep;30(9):1258–1273. http://genome.cshlp.org/content/30/9/1258, company: Cold Spring Harbor Laboratory Press Distributor: Cold Spring Harbor Laboratory Press Institution: Cold Spring Harbor Laboratory Press Label: Cold Spring Harbor Laboratory Press Publisher: Cold Spring Harbor Lab.
16. van Dijk EL, Naquin D, Gorrichon K, Jaszczyszyn Y, Ouazahrou R, Thermes C, et al. Genomics in the long-read sequencing era. Trends in Genetics 2023 Sep;39(9):649–671. https://www.sciencedirect.com/science/article/pii/S0168952523001191.
17. Kriegova E, Fillerova R, Minarik J, Savara J, Manakova J, Petrackova A, et al. Whole-genome optical mapping of bone-marrow myeloma cells reveals association of extramedullary multiple myeloma with chromosome 1 abnormalities. Scientific Reports 2021 Jul;11(1):14671. https://www.nature.com/articles/s41598-021-93835-z, number: 1 Publisher: Nature Publishing Group.
18. Li H. Minimap2: pairwise alignment for nucleotide sequences. Bioinformatics 2018 Sep;34(18):3094–3100. https://doi.org/10.1093/bioinformatics/bty191.
19. Smolka M, Paulin LF, Grochowski CM, Horner DW, Mahmoud M, Behera S, et al. Detection of mosaic and population-level structural variants with Sniffles2. Nature Biotechnology 2024 Jan;p. 1–10. https://www.nature.com/articles/s41587-023-02024-y, publisher: Nature Publishing Group.
20. Marks P, Garcia S, Barrio AM, Belhocine K, Bernate J, Bharadwaj R, et al. Resolving the full spectrum of human genome variation using Linked-Reads. Genome Research 2019 Apr;29(4):635–645.
21. Helal AA, Saad BT, Saad MT, Mosaad GS, Aboshanab KM. Benchmarking long-read aligners and SV callers for structural variation detection in Oxford nanopore sequencing data. Scientific Reports 2024 Mar;14(1):6160. https://www.nature.com/articles/s41598-024-56604-2, publisher: Nature Publishing Group.
22. Li H, Durbin R. Fast and accurate short read alignment with Burrows-Wheeler transform. Bioinformatics (Oxford, England) 2009 Jul;25(14):1754–1760.
23. Chen X, Schulz-Trieglaff O, Shaw R, Barnes B, Schlesinger F, Källberg M, et al. Manta: rapid detection of structural variants and indels for germline and cancer sequencing applications. Bioinformatics 2016 Apr;32(8):1220–1222. https://doi.org/10.1093/bioinformatics/btv710.
24. Piñero J, Ramírez-Anguita JM, Saüch-Pitarch J, Ronzano F, Centeno E, Sanz F, et al. The DisGeNET knowledge platform for disease genomics: 2019 update. Nucleic Acids Research 2020 Jan;48(D1):D845–D855. https://doi.org/10.1093/nar/gkz1021.
25. Belyeu JR, Chowdhury M, Brown J, Pedersen BS, Cormier MJ, Quinlan AR, et al. Samplot: a platform for structural variant visual validation and automated filtering. Genome Biology 2021 May;22(1):161. https://doi.org/10.1186/s13059-021-02380-5.
26. Lappalainen I, Lopez J, Skipper L, Hefferon T, Spalding JD, Garner J, et al. dbVar and DGVa: public archives for genomic structural variation. Nucleic Acids Research 2013 Jan;41(D1):D936–D941. https://doi.org/10.1093/nar/gks1213.
27. Xie H, Li W, Hu Y, Yang C, Lu J, Guo Y, et al. De novo assembly of human genome at single-cell levels. Nucleic Acids Research 2022 Jul;50(13):7479–7492. https://doi.org/10.1093/nar/gkac586.
28. Chin CS, Behera S, Metcalf GA, Gibbs RA, Boerwinkle E, Sedlazeck FJ, A pan-genome approach to decipher variants in the highly complex tandem repeat of LPA. bioRxiv; 2022. https://www.biorxiv.org/content/10.1101/2022.06.08.495395v2, pages: 2022.06.08.495395 Section: New Results.
29. Chen X, Harting J, Farrow E, Thiffault I, Kasperaviciute D, Hoischen A, et al. Comprehensive SMN1 and SMN2 profiling for spinal muscular atrophy analysis using long-read PacBio HiFi sequencing. American Journal of Human Genetics 2023 Feb;110(2):240–250. https://www.ncbi.nlm.nih.gov/pmc/articles/PMC9943720/.
30. Yang X, Wang X, Zou Y, Zhang S, Xia M, Fu L, et al. Characterization of large-scale genomic differences in the first complete human genome. Genome Biology 2023 Jul;24(1):157. https://doi.org/10.1186/s13059-023-02995-w.
31. Mandelker D, Schmidt RJ, Ankala A, McDonald Gibson K, Bowser M, Sharma H, et al. Navigating highly homologous genes in a molecular diagnostic setting: a resource for clinical next-generation sequencing. Genetics in Medicine 2016 Dec;18(12):1282–1289. https://www.sciencedirect.com/science/article/pii/S1098360021014258.
32. Landrum MJ, Lee JM, Benson M, Brown GR, Chao C, Chitipiralla S, et al. ClinVar: improving access to variant interpretations and supporting evidence. Nucleic Acids Research 2018 Jan;46(D1):D1062–D1067. https://doi.org/10.1093/nar/gkx1153.
33. Geoffroy V, Herenger Y, Kress A, Stoetzel C, Piton A, Dollfus H, et al. AnnotSV: an integrated tool for structural variations annotation. Bioinformatics 2018 Oct;34(20):3572–3574. https://doi.org/10.1093/bioinformatics/bty304.
34. Wojcik MH, Reuter CM, Marwaha S, Mahmoud M, Duyzend MH, Barseghyan H, et al. Beyond the exome: What's next in diagnostic testing for Mendelian conditions. The American Journal of Human Genetics 2023 Aug;110(8):1229–1248. https://www.sciencedirect.com/science/article/pii/S0002929723002100.
35. Merker JD, Wenger AM, Sneddon T, Grove M, Zappala Z, Fresard L, et al. Long-read genome sequencing identifies causal structural variation in a Mendelian disease. Genetics in Medicine 2018 Jan;20(1):159–163. https://www.sciencedirect.com/science/article/pii/S1098360021019328.
36. van Belzen IAEM, Schönhuth A, Kemmeren P, Hehir-Kwa JY. Structural variant detection in cancer genomes: computational challenges and perspectives for precision oncology. npj Precision Oncology 2021 Mar;5(1):1–11. https://www.nature.com/articles/s41698-021-00155-6, publisher: Nature Publishing Group.

37. Duan DM, Cheng C, Huang YS, Chung Ak, Chen PX, Chen YA, et al. Comparisons of performances of structural variants detection algorithms in solitary or combination strategy. PLOS ONE 2025;20(2):e0314982. https://journals.plos.org/plosone/article?id=10.1371/journal.pone.0314982, publisher: Public Library of Science.
38. Liu Z, Roberts R, Mercer TR, Xu J, Sedlazeck FJ, Tong W. Towards accurate and reliable resolution of structural variants for clinical diagnosis. Genome Biology 2022 Mar;23(1):68. https://doi.org/10.1186/s13059-022-02636-8.
39. Wagner J, Olson ND, Harris L, Khan Z, Farek J, Mahmoud M, et al. Benchmarking challenging small variants with linked and long reads. Cell Genomics 2022 May;2(5).
40. Lu H, Giordano F, Ning Z. Oxford Nanopore MinION Sequencing and Genome Assembly. Genomics, Proteomics & Bioinformatics 2016 Oct;14(5):265–279. https://www.sciencedirect.com/science/article/pii/S1672022916301309.
41. Martin S, Heavens D, Lan Y, Horsfield S, Clark MD, Leggett RM. Nanopore adaptive sampling: a tool for enrichment of low abundance species in metagenomic samples. Genome Biology 2022 Jan;23(1):11. https://doi.org/10.1186/s13059-021-02582-x.
42. Oehler JB, Wright H, Stark Z, Mallett AJ, Schmitz U. The application of long-read sequencing in clinical settings. Human Genomics 2023 Aug;17(1):73. https://doi.org/10.1186/s40246-023-00522-3.
43. Rhie A, Nurk S, Cechova M, Hoyt SJ, Taylor DJ, Altemose N, et al. The complete sequence of a human Y chromosome. Nature 2023 Sep;621(7978):344–354. https://www.nature.com/articles/s41586-023-06457-y, publisher: Nature Publishing Group.
44. Nurk S, Koren S, Rhie A, Rautiainen M, Bzikadze AV, Mikheenko A, et al. The complete sequence of a human genome. Science 2022 Apr;376(6588):44–53. https://www.science.org/doi/10.1126/science.abj6987, publisher: American Association for the Advancement of Science.
45. Richards S, Aziz N, Bale S, Bick D, Das S, Gastier-Foster J, et al. Standards and guidelines for the interpretation of sequence variants: a joint consensus recommendation of the American College of Medical Genetics and Genomics and the Association for Molecular Pathology. Genetics in Medicine 2015 May;17(5):405–424. https://www.gimjournal.org/article/S1098-3600%2821%2903031-8/fulltext, publisher: Elsevier.
46. Miller DT, Lee K, Abul-Husn NS, Amendola LM, Brothers K, Chung WK, et al. ACMG SF v3.2 list for reporting of secondary findings in clinical exome and genome sequencing: A policy statement of the American College of Medical Genetics and Genomics (ACMG). Genetics in Medicine 2023 Aug;25(8). https://www.gimjournal.org/article/S1098-3600(23)00879-1/fulltext, publisher: Elsevier.
47. Olivucci G, Iovino E, Innella G, Turchetti D, Pippucci T, Magini P. Long read sequencing on its way to the routine diagnostics of genetic diseases. Frontiers in Genetics 2024 Mar;15. https://www.frontiersin.org/journals/genetics/articles/10.3389/fgene.2024.1374860/full, publisher: Frontiers.
48. Negi S, Stenton SL, Berger SI, Canigiula P, McNulty B, Violich I, et al. Advancing long-read nanopore genome assembly and accurate variant calling for rare disease detection. The American Journal of Human Genetics 2025 Feb;112(2):428–449. https://www.sciencedirect.com/science/article/pii/S0002929725000023.
49. Savara J, Novosád T, Gajdoš P, Kriegová E. Comparison of structural variants detected by optical mapping with long-read next-generation sequencing. Bioinformatics 2021 Oct;37(20):3398–3404. https://doi.org/10.1093/bioinformatics/btab359.

Supplementary Fig 1

Click here to access/download
Supplementary Material
Supplementary Fig 1.pdf

Supplementary Fig 2

Click here to access/download
Supplementary Material
Supplementary Fig 2.pdf

Supplementary Fig 3

Click here to access/download
Supplementary Material
Supplementary Fig 3.pdf

Supplementary Fig 4

Click here to access/download
Supplementary Material
Supplementary Fig 4.pdf

Supplementary Fig 5

Click here to access/download
Supplementary Material
Supplementary Fig 5.pdf

Supplementary Fig 6

Click here to access/download
Supplementary Material
Supplementary Fig 6.pdf

Supplementary Table 1

Click here to access/download
Supplementary Material
Supplementary Table 1.pdf

Supplementary Table 2

Click here to access/download
Supplementary Material
Supplementary Table 2.pdf

Supplementary Table 3

Click here to access/download
Supplementary Material
Supplementary Table 3.xlsx

Supplementary Table 4

Click here to access/download
Supplementary Material
Supplementary Table 4.xlsx

Supplementary Table 5

Click here to access/download
Supplementary Material
Supplementary Table 5.xlsx

Supplementary Table 6

Click here to access/download
Supplementary Material
Supplementary Table 6.xlsx

Personal Cover
Click here to access/download;Personal Cover;Cover_letter_T2T Giga 2025-06-26.docx

Guest Editors: Jue Ruan and Fritz J Sedlazeck

Dear Editors-in-Chief, dear Guest Editors,
please find submitted online the manuscript entitled **Multiplatform comparisons and annotation of structural variants highlight the utility of the T2T reference genome in human diagnostics** for evaluation for the journal *GigaScience, for new "T2T Series: Closing the Gaps from Telomere-to-Telomere".*

Characterization of structural variants (SVs) in the human genome is incomplete, mainly due to their structural complexity, variable length between individuals and the limitations inherent in available genomic technologies as well as use of incomplete reference genome hg38. Here, we introduced an innovative multi-platform approach LongReadChecker (LoReC) suitable for comparisons and annotations of SVs across the samples, genomic technologies and databases from any short-read sequencing (SRS) and long-read sequencing (LRS) datasets using hg38/GRCh38 and gapless T2T-CHM13 reference. Our approach revolutionises the comparisons and annotations of SVs by introducing key parameters such as distance variance, intersection and gene overlap between datasets and shows that LRS together with T2T-CHM13 gapless sequences can improve the diagnostics of many patients when SRS fails to identify the cause.

We believe that this study is of great interest to the general public as there is a growing demand for clinical sequencing in all fields of medicine, not only for chronic diseases.

Neither the manuscript nor any part of its essential substance has been or will be published or submitted for publication elsewhere. All authors read and approved the final version of the manuscript. Hereby, we state that there are no financial or other relationships that could lead to a conflict of interest.

Thank you for your critical appraisal of our work.

Eva Kriegova
Corresponding author
