# Supplementary material for: Multiplatform comparisons and annotation of structural variants highlight the utility of the T2T reference genome in human diagnostics
Source: Gigascience. 2026 Mar 9;15:giag027. doi: 10.1093/gigascience/giag027 (PMC13137335; doi:10.1093/gigascience/giag027)

GigaScience

Multiplatform comparisons and annotation of structural variants highlight the utility of the T2T reference genome in human diagnostics

--Manuscript Draft--

**Manuscript Number:**	GIGA-D-25-00250R1
**Full Title:**	Multiplatform comparisons and annotation of structural variants highlight the utility of the T2T reference genome in human diagnostics
**Article Type:**	Research
**Funding Information:**	Univerzita Palackého v Olomouci (JG_2025_035) — Prof. Eva Kriegova<br>Ministerstvo Zdravotnictví Ceské Republiky (NW24-10-00395) — Prof. Eva Kriegova<br>Ministerstvo Zdravotnictví Ceské Republiky (MH CZ – DRO (FNOL, 00098892)) — Prof. Eva Kriegova<br>European Regional Development Fund (CZ.02.01.01/00/23_021/0009224) — Prof. Eva Kriegova
**Abstract:**	Background: Structural variants (SVs) are increasingly recognised as key contributors to human diseases. However, our understanding of SVs in health and disease is limited, mainly due to their structural complexity and variable length in individuals as well as limitations inherent to the available genomic technologies and reference genome used.<br>Results: To systematically evaluate SVs across human whole-genome samples using hg38/GRCh38 and gapless T2T-CHM13 references, we introduced an innovative multiplatform approach, LongReadChecker (LoReC), which advances SV comparison and annotation based on distance variance, intersection, gene overlap and the closest SV in the clinical database. Comparison of the performance in detecting SVs from public and our own whole-genome datasets from short-read sequencing (SRS), available long-read sequencing (LRS) platforms and optical genome mapping (OGM) revealed that most SVs detected by SRS were confirmed by LRS, but LRS can identify twice as many SVs (25,000 SVs/genome) with greater read mapping accuracy. Our LoReC analysis further highlights the utility of the T2T-CHM13 reference in SV detection, as 20% more deletions and 20% less insertions were detected compared with hg38/GRCh38, which was particularly evident in long-read datasets. Since 80% of the SVs detected by LRS/SRS are smaller than 0.5 kbp, OGM did not detect them.<br>Conclusions: Our study revealed that introducing distance variance, intersection, gene overlap and the closest SV in the clinical database may help compare and annotate SVs in diagnostics. Our data showed that LRS together with T2T-CHM13 gapless sequences can improve the diagnostics of patients with human diseases when SRS fails to identify the cause.
**Corresponding Author:**	Eva Kriegová<br>Palacky University Olomouc: Univerzita Palackeho v Olomouci<br>Olomouc, CZECH REPUBLIC
**Corresponding Author Secondary Information:**	
**Corresponding Author's Institution:**	Palacky University Olomouc: Univerzita Palackeho v Olomouci
**Corresponding Author's Secondary Institution:**	
**First Author:**	Jakub Savara
**First Author Secondary Information:**	
**Order of Authors:**	Jakub Savara<br>Tomas Novosad<br>Petr Gajdos

Powered by Editorial Manager® and ProduXion Manager® from Aries Systems Corporation

	Anna Petrackova
	Marek Behalek
	Jirina Manakova
	Filip Ctvrtlik
	Jiri Minarik
	Tomas Papajik
	Eva Kriegova
**Order of Authors Secondary Information:**	
**Response to Reviewers:**	AUTHORS' RESPONSE TO THE REVIEWERS' COMMENTS<br>First, we would like to thank the reviewers for thoroughly reading and critiquing our manuscript. We greatly appreciate all comments and suggestions.<br><br>Reviewers' comments:<br>Reviewer#1:<br>The author presented LoReC that compares and annotates SVs detected from different sequencing technologies, highlighting the utility of T2T reference genome in diagnostics. The usage of T2T reference genome in clinical settings is an interesting and important study, especially the newly added sequence compared to hg38. This work aims to improve SV annotation and clinical interpretation, but it lacks enough evidence or analysis to support the statement where T2T improve diagnostics of patents. Here are some major comments and suggestions:<br><br>Q1. In “True long-read sequencing technologies have …”, why the concordance variation of LRS-ONT and LRS-PacBio is high (ranges from 80%~95%)?<br>A1. We thank the reviewer for this comment. The variability in concordance of LRS-ONT and LRS-PacBio was linked to the different PacBio sequencing modes (HiFi vs continuous long reads (CLR)) used for different samples. Using HiFi PacBio mode, higher concordance was observed than in CLR (~95% vs 80%), we added this information to the Results part (page 3, Annotation of structural variants by the LongReadChecker toolkit).<br><br>Q2. In “Most structural variants detected by short-read …”, what drives the large discordance between LRS and SRS? Are there any examples suggest the advantage of T2T reference and LRS, especially for clinical diagnose?<br>A2. We thank the reviewer for this valuable comment. There is growing evidence that the discordance between SRS and LRS is linked to the mapping of short reads to multiple regions, as we showed by the MAPQ evaluation, discordant pairs and split reads by current variant callers in SRS data. We now added this information the Discussion (page 8, Discussion).<br>Given that many disease-causing SVs are located in repetitive, duplicated, inverted, or structurally complex regions that were missing or misinterpreted in older references and cannot be resolved using SRS, clinical genetics can benefit from the use of T2T-CHM13 and LRS. We also mention, that the usage of T2T-CHM13 even improves read mapping and enhances detection of clinically relevant rare and deleterious variants in SRS. We added now to the MS also some examples of advantage of T2T reference and LRS for clinical diagnose (page 9, Discussion).<br><br>Q3. It is important to know which part of the gene is shorter or longer when compared T2T to hg38. For example, in 5’UTR, even 1-10 bp difference might result in different consequences. How much protein-coding gene SVs are called from each reference? How does the gene length variation affect SV calling?<br>A3. Based on the reviewer recommendation, we performed deeper analyses on SVs and their occurrence in protein coding sequences, 5´ UTR and 3´ UTR regions in both reference sequences (new Figure 3 and new Supplementary Table 8). We also compared genes with similar lengths (± 25 bp) and found minimal differences in SV counts between the two references, whereas larger differences in length were associated with substantial differences in SV counts (page 6; new Figure 4 and Supplementary Table 9).<br>In order to preserve the maximum number of images/tables, we have moved the

original Table 1 to the Supplementary files.

Q4. SV count difference on T2T and hg38 is just the reference bias. For example, the excess insertion count on hg38 is because most them are minor alleles on hg38 but major in the population.
A4. We agree with the reviewer opinion. We added this comment to the Discussion part (page 9, Discussion).

Q5. The author mentioned genes such as LPA, SMN1/2 and DUX4. Those are all copy number variable genes and known to be diverse in the population. How is SV detected in those regions and what's the difference between references? It is also worthwhile to examine the SV discovery and mappability of genome assembly in these regions such as SMN1/2.
A5. We thank the reviewer for the comment. T2T-CHM13 significantly improves the mapping of genes with variable copy numbers and we have added examples of genes (LPA, DUX4, and SMN1/2) related to discrepancies between the two reference sequences to the Discussion (Page 9, Discussion).

Q6. In table2, why the number of Pathogenic/Likely Pathogenic is different so much between LoReC and AnnotSV. For example, LeReC annotates 1/4 (LRS-Pacbio) compared to 30/10 from AnnotSV.
A6. Thank you very much for this valuable comment. As the original manuscript compared mainly samples across the technologies, we used for annotation using ClinVar 5% size proportion. However, the majority of SVs in clinical database ClinVar span very large regions containing many genes and therefore lower the size proportion threshold of 1% may be better for comparisons. Using 1% threshold, LoReC annotations were in concordance with AnnotSV results, as stated now in the updated Table 1 and Results section (page 7-8, Results).

Reviewer #2:
Savara et al presents a toolkit LoReC for annotation and comparison of SVs. The insights into T2T-CHM13 analysis makes this manuscript timely and valuable. I was able to find the software and run it locally on an small example and the github provides sufficient documentation for usage.

Some key achievements:

- The experimental design and the results makes this manuscript very strong. The authors have gone to the extent of making sure not only synthetic data is used for analysis, which is the core strength of this manuscript.
- The authors' insights in use of T2T reference genome and discussion around critical bottleneck around clinical genomics also makes it very timely.
- The developed methods and results all show good results which is positive.

Areas to improve:
Q1: - Please compare against tools like Jasmine and SURVIOR to make this study comprehensive. I believe without comparing against existing methods, it is unclear how LoReC is novel and the advantages of using it.
A1. Based on your recommendation, we have added a new Supplementary Table 10 comparing LoReC with existing merging tools such as Jasmine and SURVIVOR. Generally speaking, LoReC provides a holistic framework for SV analysis that extends and complements the functionalities of Jasmine and SURVIVOR with outputs needed for diagnostic laboratories, as stated in the Discussion section (page 10). We added to the Supplementary Table S10 the comparison of available toolkits.

Q2:- I think the authors spend too much time comparing SRS vs LRS. In the genomics community, it is now established that LRS is better for SV detection. I would request the authors to focus on the novelty in the results rather than focusing comparisons between platforms.
A2. We agree with the reviewer that LRS should be the future of genetic diagnostics. However, one may expect that LRS will not replace SRS in the near future, but will complement it, especially in cases with negative SRS results. SRS will remain the gold standard for routine diagnostics due to its cost, speed, and established clinical pipelines, the requirement for high molecular weight DNA, the complexity of

	bioinformatic analysis, and the problems with annotating thousands of SVs, together with its limited availability in clinical laboratories and ethical issues, limit its routine use in the near future. We mention it in the Discussion (page 9, Discussion).<br><br>Q3:- The discussion section lacks comprehensive details on the current bottlenecks on annotation. It would be great if the authors could focus on discussing the annotation limitations, specially around the limitations of annotating insertions.<br>A3. We thank the reviewer for this valuable comment. Annotation of insertions is challenging. Unlike deletions, which remove annotated regions and can be assessed by absence, insertions introduce sequence not present in the reference. Moreover, the insertions frequently occur within or generate tandem repeats and segmental duplications, regions inherently difficult to assess. Consequently, insertions frequently lack reliable population frequency estimates and functional or phenotypic evidence, making confident clinical classification challenging, as we state now in the Discussion (Page 9, Discussion). We also mention how to address these challenges in the future (Page 9, Discussion).
**Additional Information:**	
**Question**	**Response**
Are you submitting this manuscript to a special series or article collection?	No
**Experimental design and statistics**<br><br>Full details of the experimental design and statistical methods used should be given in the Methods section, as detailed in our Minimum Standards Reporting Checklist. Information essential to interpreting the data presented should be made available in the figure legends.<br><br>Have you included all the information requested in your manuscript?	Yes
**Resources**<br><br>A description of all resources used, including antibodies, cell lines, animals and software tools, with enough information to allow them to be uniquely identified, should be included in the Methods section. Authors are strongly encouraged to cite Research Resource Identifiers (RRIDs) for antibodies, model organisms and tools, where possible.<br><br>Have you included the information requested as detailed in our Minimum Standards Reporting Checklist?	Yes

**Availability of data and materials**<br><br>All datasets and code on which the conclusions of the paper rely must be either included in your submission or deposited in publicly available repositories (where available and ethically appropriate), referencing such data using a unique identifier in the references and in the "Availability of Data and Materials" section of your manuscript.<br><br>Have you have met the above requirement as detailed in our Minimum Standards Reporting Checklist?	Yes
GigaScience has policies and guidelines in place for the use of generative AI-writing tools such as ChatGPT. If you have used such writing tools to assist with writing the manuscript this must be declared and cited in the text. Authors should not list AI-writing tools and other AI-assisted technologies as an author or co-author and should acknowledge that they are fully responsible for text generated or refined by AI-writing tools.<p><br><br>A summary of use (particularly in the introduction or among methods) needs to be included at the end of the paper, and the outputs should also be included as a supplementary file hosted in GigaDB or other open repositories. Please read our guidelines for more information. <p><br><br>By submitting to GigaScience, you are aware of the journal's AI-writing tools policy, and if you have declared use of such tools below, you have acknowledged this where appropriate in your manuscript and have made a summary of use and outputs available. <p><br><b>AI-assisted writing tools have been used in the preparation of this manuscript?	No

Main text
Click here to access/download;LaTeX - Main Document (TeX file);main.tex

Placeholder for OUP logo oup.pdf

(GIGA)^n SCIENCE

GigaScience, 2023, 1–12

doi: xx.xxxx/xxxx
Manuscript in Preparation
Research

RESEARCH

Multiplatform comparisons and annotation of structural variants highlight the utility of the T2T reference genome in human diagnostics

Jakub Savara [0000-0003-3067-7867][1,2], Tomas Novosad [0000-0003-2291-261X][2], Petr Gajdos [0000-0003-1831-3489][2], Anna Petrackova [0000-0001-5210-4504][1], Marek Behalek [0000-0002-9204-2691][2], Jirina Manakova [0000-0001-6931-5666][1], Filip Ctvrtlik [0000-0003-1490-7175][3], Jiri Minarik [0000-0003-0513-326X][4], Tomas Papajik [0000-0001-5779-1139][4] and Eva Kriegova [0000-0002-8969-4197][1]

[1]Department of Immunology, Faculty of Medicine and Dentistry, Palacký University Olomouc and University Hospital Olomouc, Olomouc, Czech Republic and [2]Department of Computer Science, Faculty of Electrical Engineering and Computer Science, VSB-Technical University of Ostrava, Ostrava, Czech Republic and [3]Department of Radiology, Faculty of Medicine and Dentistry, Palacký University Olomouc and University Hospital Olomouc, Olomouc, Czech Republic and [4]Department of Hemato-oncology, Faculty of Medicine and Dentistry, Palacký University Olomouc and University Hospital Olomouc, Olomouc, Czech Republic

Abstract

Background: Structural variants (SVs) are increasingly recognised as key contributors to human diseases. However, our understanding of SVs in health and disease is limited, mainly due to their structural complexity and variable length in individuals as well as limitations inherent to the available genomic technologies and reference genome used. **Results:** To systematically evaluate SVs across human whole-genome samples using hg38/GRCh38 and gapless T2T-CHM13 references, we introduced an innovative multiplatform approach, LongReadChecker (LoReC), which advances SV comparison and annotation based on distance variance, intersection, gene overlap and the closest SV in the clinical database. Comparison of the performance in detecting SVs from public and our own whole-genome datasets from short-read sequencing (SRS), available long-read sequencing (LRS) platforms and optical genome mapping (OGM) revealed that most SVs detected by SRS were confirmed by LRS, but LRS can identify twice as many SVs (25,000 SVs/genome) with greater read mapping accuracy. Our LoReC analysis further highlights the utility of the T2T-CHM13 reference in SV detection, as 20% more deletions and 20% less insertions were detected compared with hg38/GRCh38, which was particularly evident in long-read datasets. Since 80% of the SVs detected by LRS/SRS are smaller than 0.5 kbp, OGM did not detect them. **Conclusions:** Our study revealed that introducing distance variance, intersection, gene overlap and the closest SV in the clinical database may help compare and annotate SVs in diagnostics. Our data showed that LRS together with T2T-CHM13 gapless sequences can improve the diagnostics of patients with human diseases when SRS fails to identify the cause.

Key words: Next-generation sequencing; structural variants; annotations; LongReadChecker (LoReC) toolkit; long-read technology

Compiled on: March 5, 2026.
Draft manuscript prepared by the author.

Introduction

Structural variants (SVs) are a major source of human genetic diversity and arise from the breakdown and rejoining of DNA fragments, which can lead to the loss, gain and rearrangement of genes and regulatory elements [1]. Since SVs are larger than 50 bp and can affect thousands to millions of nucleotides [2, 3], they are expected to have a strong effect on transcriptional regulation in health and disease [4]. Structural variant detection is of particular importance, as they are responsible for more than 25% of all rare protein truncations in a genome and are associated with many diseases [5, 6]. However, SV characterisation and functional interrogation have largely lagged behind single nucleotide variations and small insertions and deletions (INDELs), mainly due to their structural complexity and variable length in individuals as well as limitations inherent to the available genomic technologies [7]. Traditionally, SVs in the human genome have been detected using array-based methods or locus-specific assays for targeted regions [8].

Currently, great progress is being made in the detection of SVs using short-read sequencing (SRS), which remains essential due to practical and cost considerations in clinical diagnostics [9]. Novel genomic technologies for SV detection are also being rapidly developed. Among them, long-read sequencing (LRS) has demonstrated a high potential for detecting SVs [10] through longer reads and increased accuracy compared with SRS [1]. The increasing throughput, lower prices and portability of LRS technologies increase the potential of LRS introduction into diagnostic testing, particularly for patients with genetic disorders with negative results using SRS [11, 12]. However, accurate and precise identification of SVs in specific samples and/or across samples is challenging [13]. In addition, data on the performance and comparison of currently available genomic technologies, the clinical utility of the novel human reference assembly T2T-CHM13 (T2T) and clinical databases for the annotation of detected SVs are incomplete.

Therefore, this study focused on conducting a comprehensive comparison of SVs detected from whole-genome datasets, both public and our own, obtained using SRS, LRS from currently available technologies and optical genome mapping (OGM). To achieve this, we introduced an innovative bioinformatics approach LongReadChecker (LoReC)[14] enabling comparisons of SVs across whole-genome datasets, technologies and reference genomes, including their annotations using clinical genomic databases. This multi-platform approach revolutionizes the comparisons of SVs by introducing key parameters such as distance variance, intersection, and gene overlap between datasets, thus advancing their comparison and annotation across samples or technologies or clinical databases. Our study further highlights the utility of T2T reference and long-read technologies in clinical and research applications.

Materials and methods

Public and our own human whole-genome datasets

This study evaluated whole-genome datasets from a human DNA standard (NA12878 cell line, also known as HG001; B-lymphocyte; female; healthy; Genome in a Bottle Consortium (GIAB))[15] and a breast cancer cell line (SKBR3)[16] obtained by i) traditional SRS (Illumina, CA, USA), ii) true LRS on a single-molecule real-time platform from Pacific Biosciences (LRS-PacBio), iii) true LRS from Oxford Nanopore Technologies (LRS-ONT), iv) synthetic LRS from transposase enzyme-linked LRS (LRS-TELL-Seq; Universal Sequencing Technology, MA, USA), v) synthetic LRS from Illumina Complete Long-Reads (LRS-ICLR; Illumina) and vi) synthetic LRS from 10× assay (LRS-10×; 10× Genomics). All synthetic LRS were sequenced on an Illumina short-read platform. In addition, OGM from Bionano Genomics (CA, USA) was used (Supplementary Table 1). The principles of the LRS technologies used are described elsewhere [17]. Our own datasets from SRS, LRS-TELL-Seq, LRS-ICLR and OGM were obtained for two diagnostic samples, P3 (pheochromocytoma adrenal medulla tissue, man, 63 years) and P48 (enriched CD138+ myeloma cells from bone marrow aspirate, woman, 39 years, IgG lambda, stage IIIA, ISS II), as well as for the NA12878 cell line. Raw sequencing data for SKBR3 cell lines for different technologies are available within the SRA under BioProject PRJNA476239 [16]. Raw sequencing data for NA12878 are available within GIAB FTP release and under BioProject PRJNA200694 [15, 18].

For our own analysis, the high-molecular-weight DNA from tumour tissues and cells from cell lines was isolated from agarose plugs as reported previously [19]. Next-generation sequencing (NGS) libraries were prepared according to the manufacturer recommendations for LRS-ICLR, LRS-TELL-Seq™ and SRS TruSeq DNA PCR-Free (Illumina) and sequenced (150 bp paired-end reads) on a NovaSeq 6000 system (Illumina). In addition, OGM labelling and measurements using the Bionano Saphyr instrument (Bionano Genomics) were performed as reported previously [19]. The sequence depth for LRS and SRS was approximately 30× and for OGM 300×.

Bioinformatic processing of whole-genome datasets from different short-read sequencing and long-read sequencing platforms

To minimise the difference in precision, recall and F1-score metrics using different callers/aligners, we used the LRS aligner Minimap2 [20] and the SV caller Sniffles2 (v2.2) software [21] for true LRS and the LongRanger software (v2.2.2) [22] for synthetic LRS analyses, which provided a strong basis for SV pipeline calling in LRS [23]. Moreover, this combination is the basis for the Illumina DRAGEN analysis of Illumina LRS-ICLR, an approach that was also compared in this study. For SRS datasets, BWA aligner software (v0.7.17) [24] and the Manta structural variant caller (v1.6.0) [25] were used. Raw data from OGM were analysed using Bionano Access (v1.8) software by Bionano Genomics, and the *De Novo* assembly pipeline was performed using Bionano Solve tools (v3.8) (Bionano Genomics). The hg38 (GRCh38.p14) and T2T-CHM13 (v2.0) human reference genomes were used. Gene and pseudogene coordinates, names and biotypes (e.g. protein coding) are based on MANE transcripts from RefSeq NCBI annotations (version 110); in all analyses, the Y chromosome and alternate (ALT) contigs were excluded. The list of medically relevant genes is derived from the DisGeNET [26] database (DisGeNET v20.1), which includes information on gene/variant-disease associations (VDAs) originating from ClinVar, the GWAS Catalog, UniProt, GAD and BeFree data [26]. The selection of medically relevant genes is based on gene disease associations (GDAs) and VDAs, GDA/VDA > 0.5 and evidence index > 0.8, indicating that most publications support GDA/VDA [26].

Comparison of structural variants using the LongReadChecker toolkit

For comparison of SVs across different samples, technologies, and databases, we designed our own LoReC toolkit, containing two tools: the LoReC comparator (source file: variant call format, vcf) and LoReC coverage (source file: mapped reads in binary format, bam). The LoReC comparator can find the closest SV detected by another technology, database or reference genome for each SV across the whole genome or region(s) of interest. For each comparison, the following parameters need to be established: 1) the distance variance threshold (the accepted difference in bp between

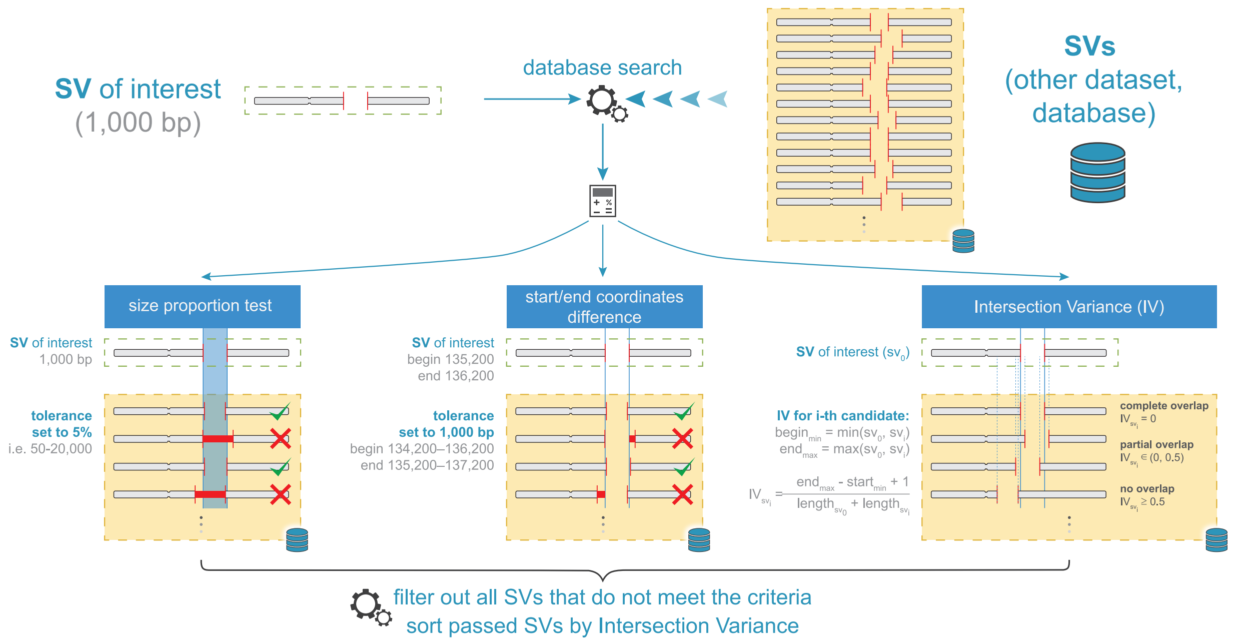

Fig. 1. Annotation of structural variants (SVs) detected in the short-read and long-read sequencing datasets in clinical diagnostics using the LoReC toolkit. Due to the high variability in SV breakpoints, three parameters are needed for the SV comparison with SVs from clinical databases, such as the distance in bp between the sample SV and the database SV (start and end coordinate difference), the intersection (intersection factor) and the minimum overlap between the sample SV(s) and the database SV(s) (size proportion).

the start and end coordinates between each SV in dataset 1 and the nearest SV in dataset 2, expressed as the sum of the two differences), 2) the intersection factor (the overlap between each SV in dataset 1 and the nearest SV in dataset 2; 0 = 100% overlap, 0–0.5 = partial overlap, >0.5 = no overlap) and 3) the minimal size proportion (the percentage of the size in bp between each SV in dataset 1 and the nearest SV in dataset 2; e.g. 5% means that the SVs in dataset 1 encompass at least 5% of the SVs in dataset 2 or vice versa). To compare SVs in the same regions detected by different technologies, which may differ in size and coordinates because of the different principles of the method, the LoReC toolkit lists the closest SV of the same type (e.g. deletion, insertion) from dataset 2 and its coordinates, distance, intersection factor and size proportion compared with the SV from dataset 1. A distance variance threshold of 1,000 bp was used for a comparison between different NGS platforms and 50,000 bp between NGS and OGM, and an intersection factor of 0 to 0.5 and a minimum size fraction of 5% were used to indicate SVs that overlap and are very similar; different parameters can be used for filtering (Fig. 1). To visually inspect the SVs of interest, the Samplot tool was used [27].

The LoReC coverage tool is able to provide coverage of the gene/region of interest, including statistics (mean, median, min, max, Q1 and Q3 coverages), filter reads based on the mapping quality and visualisation of the regions of interest. The LoReC coverage outputs are as follows: 1) coverage across regions of interest or across the whole genome based on the coordinates and gene names given in a region file; 2) coverage calculations based on the read mapping quality (MAPQ) threshold value, which is able to filter out the reads that map to multiple regions or those of poor quality (MAPQ0 = high probability that a read is mapped to multiple locations with an equal score, MAPQ1 = high probability that a read is mapped to at least two locations, MAPQ50 = 99.999% probability that a read is mapped to a unique region; Fig. 2); and 3) visualisation of the regions of interest specified in the region files, which allows a comparison of multiple technologies, different samples or reference genomes. As low coverage genes are marked those in which Q1 coverage was below 25% of the mean genome coverage of the sample.

Annotation of structural variants using the LongReadChecker toolkit

Another functionality of the LoReC comparator is to annotate detected SVs based on the annotation file(s) from dbVar (NCBI)[28, 29]. As the current dbVar_common and ClinVar databases are based on hg38, SV annotations were performed primarily on this reference. For experimental purposes, dbVar_common and ClinVar annotation files were converted from hg38 to the CHM13-T2T reference genome using BCFtools/liftover [30]. The following parameters were established for SV comparisons: a threshold of ±1,000 bp in the distance between the start and end coordinates of the SVs compared, an intersection factor threshold of 0.5 and a minimal size proportion of 1%. First, the detected SVs are compared with SVs included in the dbVar_common database of common SVs found with a frequency >1% in the population (e.g. nstd186, NCBI Curated Common Structural Variants) or any other vcf file of interest and must fulfil the set-up criteria to be marked as PASS. Second, detected SVs not found in the dbVar_common database are compared with the ClinVar SV database (e.g. summary of nstd102, Clinical Structural Variants) or vcf of interest, and for SVs present in the database, the clinical significance of SVs is reported (e.g. pathogenetic, likely pathogenic, variant of uncertain significance [VUS], likely benign, benign). For each SV, LoReC provides the main annotation (ClinVar accession ID) with the lowest intersection factor (=highest overlap) that passed the selected filters. In addition, LoReC provides all passed ClinVar accession IDs which meet filtering criteria for further investigation. For each gene(s), it is possible to filter their SVs in output tables generated by LoReC toolkit. For SVs not found in the ClinVar SV database and with an overlap of at least one gene, additional information is reported through the LoReC toolkit based on the NCBI annotation file, which includes the biotype, gene description and gene/VDAs present in DisGeNET, allowing filtering based on these features [31].

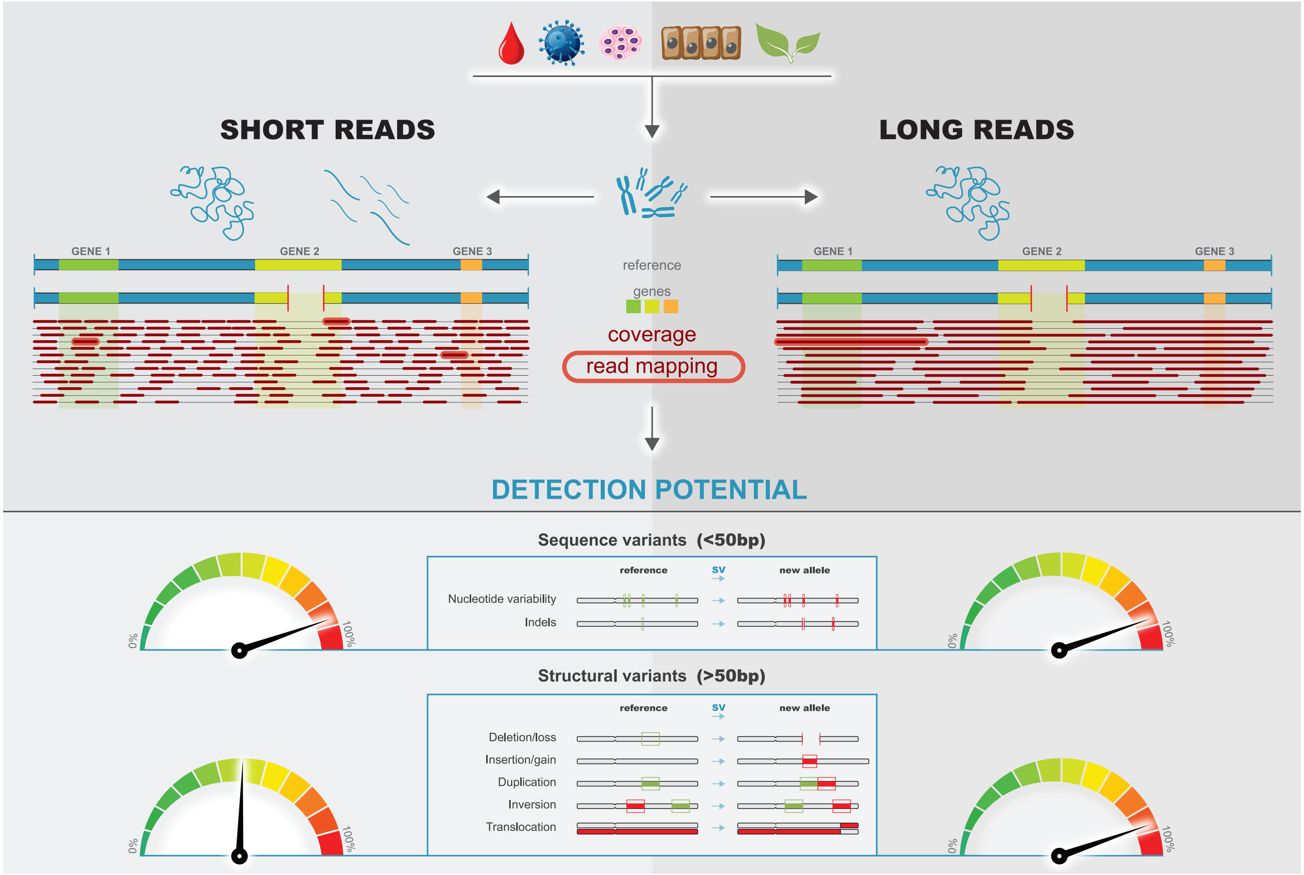

Fig. 2. Principle of short-read sequencing (SRS) and long-read sequencing (LRS) and their detection potential for sequence and structural variants. Example of the coverage and read mapping for a heterozygous gene deletion is shown; LRS requires DNA with higher molecular weight than SRS. The lower diagram shows the detection potential for sequence and structural variants using SRS and LRS.

Results

True long-read sequencing technologies have superior performance in detecting structural variants

To evaluate the performance of the currently available LRS technologies and SRS, whole-genome sequencing datasets for the NA12878 healthy [15] and SKBR3 breast cancer [16] cell lines and two diagnostic tissue samples, P3 (pheochromocytoma tissue) and P48 (multiple myeloma bone marrow aspirate), both public and our own (Supplementary Table 1), were compared using different LRS and SRS platforms. Among the tested platforms were the following: 1) two true LRS technology (LRS-PacBio and LRS-ONT) instruments; 2) three synthetic linked-read LRS approaches (LRS-ICLR, LRS-10× and LRS-TELL-Seq), sequenced on the short-read Illumina platform; 3) SRS on Illumina; and 4) OGM from Bionano Genomics. For comparisons of detected SVs, their types, coordinates, sizes, intersection factors, size proportions and coverage between different technologies, reference genomes (hg38/GRCh38.p14; T2T-CHM13, v2.0) and annotations of detected SVs according to clinical databases, a LoReC toolkit was used (Fig. 1). For more details on functionalities, see the Materials and Methods Section.

Using hg38, approximately 25,000 SVs per genome were detected using LRS-PacBio and LRS-ONT, 14,000–15,000 using LRS-ICLR, 10,000–12,500 using LRS-10×, 12,500–15,000 using SRS and 4,000 using OGM. Insertions were the most common SVs detected by all technologies: 14,000 per genome using LRS-PacBio and LRS-ONT, 5,000–6,000 using LRS-ICLR, 6,000–7,000 using SRS and 2,500 using OGM. Insertions were not detected in the LRS-TELL-Seq and LRS-10× datasets, as the LongRanger pipeline available to analyse synthetic reads cannot call insertions. Deletions were the second most common SVs: 10,500 per genome using LRS-PacBio and LRS-ONT, 9,000–10,000 using LRS-ICLR, 3,500–5,000 using LRS-10×, 5,000–6,000 using SRS and 1,250 using OGM. Regarding other SVs, such as inversions, duplications and breakends/translocations, their counts varied across samples and genomes, ranging from 240 to 1,430 per genome, depending on the technology (Supplementary Table 2, Fig. 3, Supplementary Fig. 1). Selected SVs from the OGM and LRS datasets were verified using fluorescence immunophenotyping and interphase cytogenetics as a tool for the investigation of neoplasms (FICTION), arrayCGH and/or targeted SRS.

When comparing available technologies, the majority of deletions and insertions (~80%–95%) detected by LRS-ONT were confirmed by LRS-PacBio, whereas higher concordance (~95%) was observed using the PacBio high fidelity (HiFi) sequencing mode and lower concordance (~80%) using continuous long reads. However, less than 50% were detected by SRS and 55%–71% by LRS-ICLR. Furthermore, LRS-TELL-Seq and LRS-10x confirmed approximately 25%–50% of the deletions detected by LRS-ONT/LRS-PacBio, but it was not possible to call insertions from synthetic reads. Regarding inversions, duplications and breakends/translocations, the best overlap was observed between LRS-PacBio and LRS-ONT, and a moderate overlap was identified using synthetic LRS and SRS (Supplementary Tables 3 and 4).

Most structural variants detected by short-read sequencing were confirmed by long-read sequencing

Next, we compared the SVs detected by SRS, the most widely used platform today in clinical diagnostics, with SVs detected

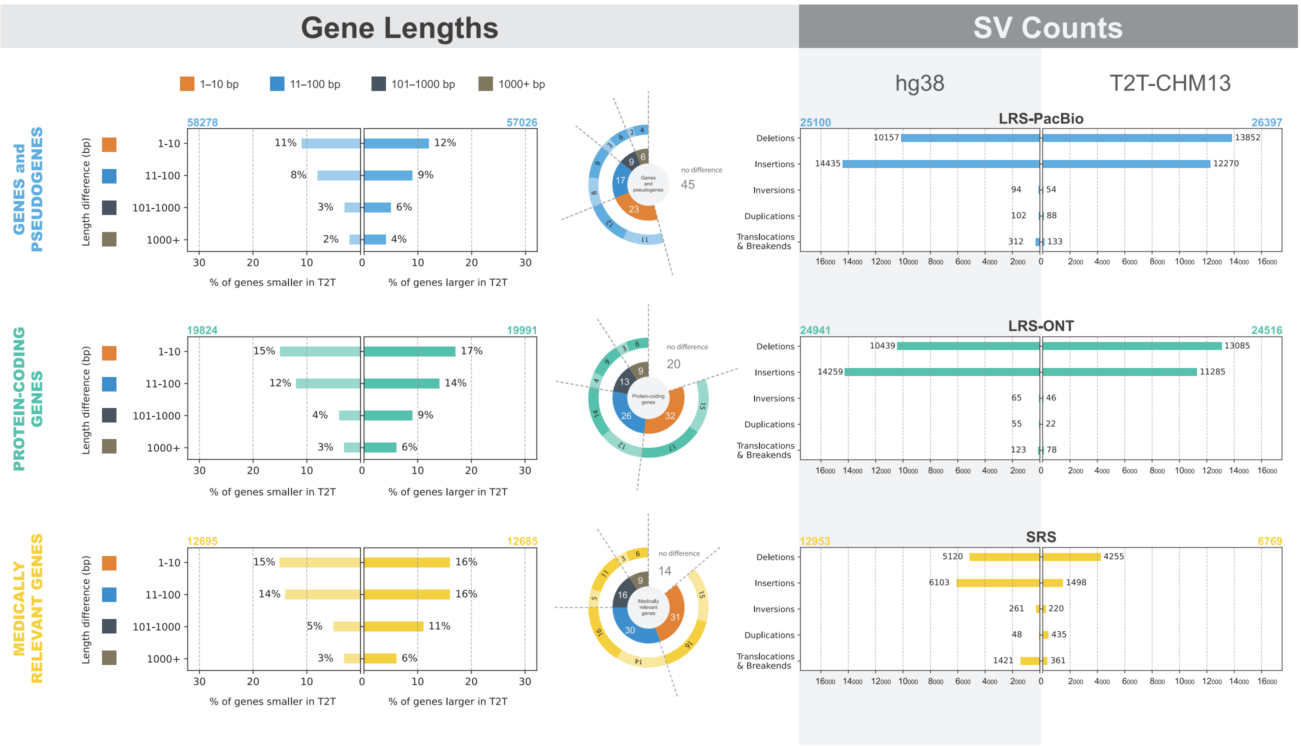

Fig. 3. Comparison of gene lengths and the number of structural variants (SVs) in the NA12878 cell line using hg38 and T2T-CHM13 references. The differences in the number and lengths of genes and pseudogenes, protein-coding genes and medically relevant genes for both references are presented; genes were annotated based on RefSeq NCBI (version 110, based on gene IDs and MANE transcript), excluding genes on the Y chromosome and ALT contigs. The number of SVs detected by long-read sequencing (LRS-PacBio, LRS-ONT) and short-read sequencing (SRS) on both references are shown on the right.

by LRS-ONT and LRS-PacBio and synthetic LRS. Most of the deletions and insertions detected by SRS (10,000–12,500 per genome) were smaller than 0.5 kbp (~80%) and were confirmed by LRS-PacBio/LRS-ONT (~90%). Although LRS-ICLR was similar to true LRS in detecting deletions, it had less precision in the case of insertions (~50%), as this technology is based on SRS. Notably, SRS failed to detect approximately 50% of the SVs detected by LRS (Supplementary Tables 3 and 5).

True long-read sequencing and short-read sequencing technologies have superior performance over optical genome mapping

We also compared LRS and SRS with OGM, a non-sequencing technology based on the labelling of high-molecular-weight DNA using fluorophore tags on specific sequence motifs. As OGM does not detect SVs smaller than 0.5 kbp or SVs in genomic regions that lack specific sequence motifs, OGM detected a lower number of SVs than LRS or SRS (hg38: 3,946 vs 24,941 vs 12,953; T2T-CHM13: 3,082 vs 24,516 vs 6,769). The majority (~80%) of deletions and insertions detected by OGM were confirmed by LRS-ONT and LRS-PacBio; SRS confirmed approximately 35% of the insertions and approximately 50%–60% of the deletions detected by OGM (Supplementary Tables 3 and 6). Comparison of OGM with LRS and SRS showed that OGM does not provide the exact coordinates of individual SVs, and their position may be substantially different from coordinates detected by LRS and SRS (Supplementary Fig. 2).

True long reads map with high probability to unique regions compared with short reads

Next, we evaluated the MAPQ [24], a measure of the probability that a read is misplaced, for different LRS and SRS technologies. For MAPQ0, a default setting in the current SRS/LRS aligners and variant callers that allows read mapping to multiple regions, most genes were covered by all SRS/LRS technologies (Fig. 2, Supplementary Fig. 3, Supplementary Table 7). To eliminate problematic regions with misplaced reads, current SRS pipelines often mask these repetitive dark regions. When stricter MAPQ1 and MAPQ50 (associated with a lower probability of misplaced reads than MAPQ0) were applied, many regions of the genome were not covered in the SRS datasets, including many protein-coding genes and medically relevant genes (Fig. 2, Supplementary Fig. 3, Supplementary Table 7). In LRS-PacBio HiFi datasets, most reads were mapped with high probability to a unique region, as demonstrated by applying MAPQ1/MAPQ50. Regarding LRS-ONT, mapping to multiple regions is less probable because of the very long reads (up to Mbp); however, many reads are of low quality, resulting in less accuracy in mapping (Fig. 2, Supplementary Fig. 3, Supplementary Table 7).

T2T-CHM13 reference improves the analysis of structural variants for long-read sequencing and short-read sequencing datasets

To understand the added value of the gapless T2T-CHM13 reference with the currently used hg38, SVs in known genes and pseudogenes, protein-coding genes and medically relevant genes and their lengths were compared for the LRS and SRS datasets, excluding the Y chromosome and ALT contigs (Fig. 3, Supplementary Table 7). For comparison between references, unique gene IDs from RefSeq NCBI annotations (version 110) using the main MANE transcript were used, which enables also comparison of gene paralogs. Using the T2T-CHM13 reference, the number of deletions increased by more than 20% and the number of insertions decreased by more than 20% using the LRS-ONT and LRS-PacBio datasets compared with hg38 (Fig. 3, Supplementary Fig. 1, Supplementary Table 2). Using SRS, approximately 10% more deletions and 80%

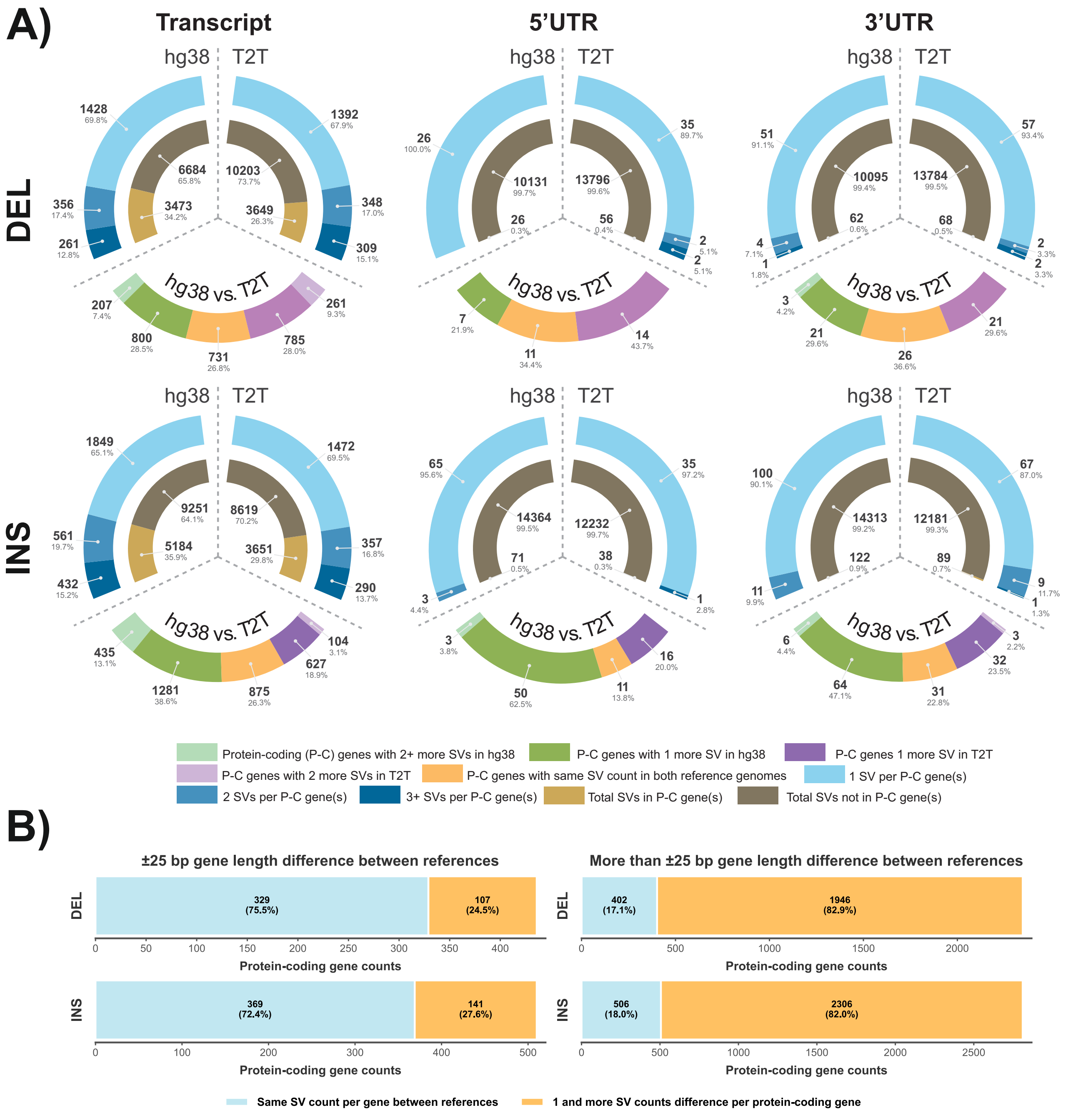

Fig. 4. Comparison of detected structural variants (SVs) located in A) protein-coding sequences (MANE transcripts) and 5'UTR and 3'UTR regions for both hg38 and T2T-CHM13 reference genomes and (B) genes with length differences of more and less than ± 25 bp between reference genomes hg38 and T2T-CHM13.

fewer insertions were detected using T2T-CHM13 than using hg38 (Fig. 3, Supplementary Fig. 1, Supplementary Table 2). In the unique regions of T2T-CHM13, numerous translocations, deletions and other SVs were detected in the telomere, centromere and subcentromere regions in all analysed datasets (Supplementary Tables 4–6, Supplementary Fig. 4).

Of the detected SVs, approximately 27%–33% were located in the protein-coding sequences, approximately 0.5% in each of the 5' UTR and 3' UTR regions and the remainder in the non-coding regions (Fig. 4, Supplementary Table 8). The T2T-CHM13 reference also refined the length of the genes. Comparing T2T-CHM13 and hg38 references for 54,553 genes and pseudogenes that overlap based on the gene ID (Fig. 3, Supplementary Table 7), 45.6% were the same length, 23.7% differed by 1–10 bp, 16.8% by 11–100 bp, 8.2% by 101–1,000 bp and 5.6% by more than 1,000 bp. Regarding protein-coding genes (medically relevant genes), 19.9% (14.3%) were the same length, 31.7% (31.0%) differed by 1–10 bp, 26.7% (30.2%) by 11–100 bp, 13.3% (15.8%) by 101–1,000 bp and 8.4% (8.8%) by more than 1,000 bp (Fig. 3). For genes of similar length (± 25 bp), minimal differences in SV counts were observed between the two references, whereas larger differences in length were associated with substantial differences in SV counts (Fig. 4, Supplementary Table 8). Among those with the largest differences in length were *GRK1* [32] and *LPA* [33] and many other genes, such as *SMN1&2* [34], *DUX4* and *HLA-DRB5* or *GBA* and its pseudogene *GBAP1*, were found to be disassembled, not correctly assembled or highly similar in hg38 (Supplementary Fig. 5, Supplementary Fig. 6, Supplementary Table 7). Furthermore, additional 167 protein-coding genes were annotated in T2T-CHM13 than in hg38 (Supplementary Table 7). To complement the added value of T2T-CHM13, we marked the genes found in the discrepant regions between hg19 and hg38 [35, 36] (Supplementary Table 7).

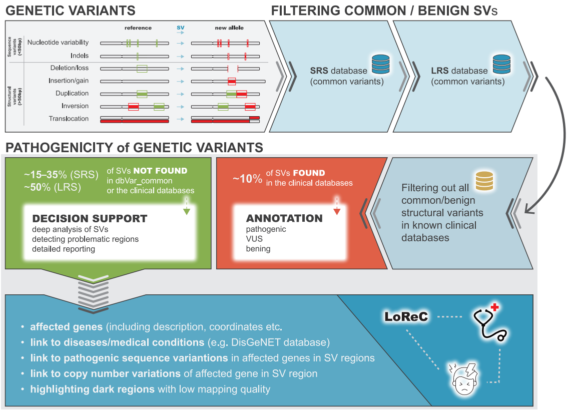

Fig. 5. Suggested workflow for annotation of structural variants (SVs) detected by long-read sequencing in diagnostic samples. First, SVs in the samples are filtered using a dbVar_common database or similar to filter out common and benign SVs. The SVs not found in the dbVar_common database are compared with the ClinVar SV database or similar. For SVs not found in the ClinVar database, the LoReC toolkit is used for the comparison and annotation of SVs in the samples based on the NCBI annotation file(s), including affected gene (gene description, coordinates) and link to the disease or medical condition based on the gene/variant-disease associations present in DisGeNET or similar.

Annotation of structural variants in clinical databases is challenging

To annotate the SVs detected by the tested technologies, we used a LoReC comparator toolkit to compare the detected SVs with the dbVar_common (NCBI dbVar Curated Common Structural Variants,

Table 1. Annotations of deletions detected in whole-genome datasets for the NA12878 and SKBR3 cell lines and P3 and S48 diagnostic samples by SRS and LRS technologies employing A) LoReC using dbVar_common and ClinVar and B) AnnotSV, both for the hg38 reference.

ALL		A) LoReC*							B) AnnotSV			
**Sample**	**Technology**	Total DEL	dbVar_common	ClinVar	Pathogenic/ Likely Pathogenic	VUS	Benign/ Likely Benign	Not annotated	Total DEL+BND	Benign	Pathogenic/ Likely Pathogenic	VUS
**NA12878**	SRS	5,120	3,332 (65%)	594 (12%)	29/11	62	461/19	1,734 (34%)	5,525	4,598 (83%)	32/6	889 (16%)
	LRS-PacBio	10,157	4,696 (46%)	1,085 (11%)	41/19	91	879/39	5,220 (51%)	10,157	6,030 (59%)	7/6	4,114 (41%)
	LRS-ONT	10,439	4,692 (45%)	1,090 (11%)	42/21	90	883/36	5,519 (53%)	10,439	5,893 (56%)	2/7	4,537 (43%)
	LRS-ICLR	9,412	4,018 (43%)	832 (9%)	42/18	75	655/25	5,199 (55%)	9,412	4,767 (51%)	2/4	4,639 (49%)
**SKBR3**	SRS	2,920	2,261 (78%)	549 (19%)	65/9	91	358/17	570 (20%)	2,890	2,373 (82%)	46/32	457 (16%)
	LRS-PacBio	9,097	4,168 (46%)	932 (10%)	62/15	98	710/33	4,693 (52%)	9,097	6,095 (67%)	30/10	2,962 (33%)
	LRS-ONT	10,983	4,343 (40%)	979 (9%)	64/18	98	748/32	6,402 (58%)	10,983	7,183 (65%)	29/15	3,756 (34%)
**P3**	SRS	5,018	3,222 (64%)	580 (12%)	28/8	72	437/22	1,741 (35%)	5,478	4,463 (81%)	36/4	975 (18%)
	LRS-ICLR	9,166	3,899 (43%)	756 (8%)	35/16	82	579/30	5,095 (56%)	9,166	4,486 (49%)	0/3	4,677 (51%)
**S48**	SRS	5,632	3,480 (62%)	652 (12%)	39/12	82	480/24	2,069 (37%)	6,199	5,035 (81%)	84/6	1,074 (17%)
	LRS-ICLR	9,279	3,970 (43%)	811 (9%)	49/13	89	613/29	5,102 (55%)	9,279	4,715 (51%)	5/6	4,553 (49%)

Legend: SRS, short-read sequencing by Illumina platform; LRS-PacBio, true long-read sequencing by Pacific Biosciences; LRS-ONT, true long-read sequencing by Oxford Nanopore Technologies; LRS-ICLR, synthetic long-read sequencing by Illumina - complete long-reads technology on Illumina platform; LRS-TELL-Seq, synthetic long-read sequencing by Universal Sequencing Technology on Illumina platform; LRS-10x, synthetic long-read sequencing by 10x Genomics on Illumina platform; OGM, optical genome mapping by Bionano Genomics; VUS, variant of unknown significance; DEL, deletion; BND, Breakends. *The main annotation with the lowest intersection factor (the highest overlap) that passed the selected filters between sample and database.

GnomAD and other sources, ID: nstd186) [28] and ClinVar (ID: nstd102) clinical datasets [37]. The dbVar_common database includes SVs that occur with a frequency of >1% in the population. As the current ClinVar database is based on hg38, annotations of SVs were primarily performed using this reference. Given that SV coordinates may vary from individual to individual, the following parameters need to be defined prior to each analysis: 1) the distance variance threshold (acceptable difference in bp between the SV of interest and the SV in the clinical database), 2) the intersection factor (overlap between the SV of interest and the nearest SV in the clinical database) and 3) the minimum size fraction (minimum proportion of the SV of interest and the nearest SV in the clinical database) (see the Materials and Methods for details). All SVs were also evaluated using the AnnotSV tool [38] (Table 1, Fig. 1).

Regarding deletions, 62%–78% of deletions detected by the SRS datasets overlapped with the deletions in dbVar_common and approximately 10% with those in ClinVar. When using AnnotSV, approximately 80% of the SVs in the SRS datasets were annotated as benign, approximately 1% (38–90) of deletions per sample were annotated as pathogenic/likely pathogenic and less than 18% were annotated as a VUS. Using true LRS, approximately 45% of deletions were detected in dbVar_common and 10% in ClinVar; for more than half the deletions, no annotation was available (Table 1). Using ClinVar annotations, the majority of SVs in this database span very large regions containing many genes; therefore, we used the size proportion parameter of 1% for LoReC annotations. Using this threshold, the LoReC annotations were consistent with the AnnotSV results: 49%–67% of SVs in the LRS datasets were annotated as benign, approximately 10 deletions (~0.1%) were annotated as pathogenic/likely pathogenic and approximately 4,500 deletions (30%–50%) as a VUS, depending on the sample and technology used (Table 1). Additionally, LoReC observed more pathogenic/likely pathogenic SVs in LRS datasets (Table 1). Regarding insertions, the dbVar_common/ClinVar databases contain only breakpoints and lack information on the insertion length for most insertions, making annotation impossible, which is also problematic for inversions and translocations. For information regarding annotations using the T2T-CHM13 reference genome, see Supplementary Table 9.

Annotation of the structural variants not presented in the clinical databases by LoReC

We suggest a workflow for diagnostic laboratories to annotate SVs from LRS/SRS. Briefly, after filtering out benign/common SVs present in the clinical databases (currently, mainly based on SRS, but in the future, LRS datasets will be added; e.g. dbVar), the remaining SVs are compared with the ClinVar clinical database or similar (Fig. 5). For SVs not presented in the dbVar_common/ClinVar databases, our LoReC toolkit enables the addition of custom annotations for regions/genes within the detected SVs. In particular, genes may be linked to the gene/VDAs provided in the annotation file (e.g. DisGeNET) or other custom annotations (Supplementary Table 7). In addition, the LoReC toolkit provides coverage for genes and regions for selected mapping quality, information about the SV localisation (e.g. protein-coding region, medical relevant genes), and enables the visualisation of regions of interest for expert evaluation.

Discussion

The importance of SVs in clinical diagnostics continues to expand due to advances in genomic technologies and wet lab protocols, the introduction of the gapless reference sequence T2T-CHM13 and the release of high-quality datasets. Despite the growing importance of SVs in human diseases, our knowledge of SVs in health and disease is limited, largely due to their structural complexity and variable length in individuals as well as the limitations inherent in available genomic technologies. Here, we comprehensively evaluated SVs from human whole-genome datasets obtained from SRS, all available LRS and OGM platforms across different samples, technologies and clinical databases using two human references (hg38 and T2T-CHM13). Moreover, our multiplatform approach enables annotation of detected SVs to support the implementation of SV diagnostics in clinical practice. Despite advances in clinical genetic diagnostics, approximately 50% of all suspected Mendelian diseases and many sporadic development syndromes and chronic diseases remain unresolved [39, 40]. The diagnostics of SVs is relevant not only in rare genetic diseases; SVs are also important contributors to chronic diseases, including cancer [5, 6, 41]. However, the technical limitations inherent in the available genomic technologies, as well as the structural complexity of SVs and their variable length in individuals, have led to an incomplete characterisation of SVs in the human genome in relation to health and disease compared with single nucleotide variations and small INDELs. To gain more insights into the performance of available genomic technologies, we compared our own and public whole-genome datasets from SRS, two LRS platforms (PacBio-LRS, ONT-LRS) requiring specific instruments, and three LRS approaches utilising synthetic long reads sequenced on conventional short-read NGSs. To achieve this, we developed the multiplatform LoReC toolkit, which compares the size and type of SVs, their overlap, coverage, coordinates, affected genes and disease associations from different SRS and LRS datasets in specific samples and/or across samples and databases, regardless of which of the many available algorithms was used to detect SVs [42, 43].

The LoReC toolkit was developed to provide comprehensive SV comparison and annotation between samples, technologies and databases for use in diagnostic laboratories, thereby extending the functionalities of SV comparison toolkits such as Jasmine [44] and SURVIVOR [45] (Supplementary Table 10). First, we were interested in the performance of SRS compared with third-generation technologies. On average, approximately 13,000 SVs/genome were detected by SRS and twice as many (~25,000 SVs/genome) by LRS. The lower performance of SRS in SV detection may arise from mapping short reads to multiple regions, discordant pairs and split reads, which limit SV detection by current variant callers [46, 47]. Notably, most of the SVs detected by SRS were also detected by LRS. Our findings are consistent with those of others showing that LRS can identify hidden disease-related SVs that are not detected by SRS [3, 36, 46]. Since 80% of the SVs detected by LRS/SRS were smaller than 0.5 kbp, OGM did not detect most of the SVs detected by LRS/SRS. The most common type of SVs were deletions and insertions, whereas half as many deletions (~5,000 vs ~10,000) and insertions (~6,000 vs more than 12,000) per sample were detected by SRS compared with LRS. Our data reveal that LRS-ONT and LRS-PacBio technologies have superior performance in detecting SVs to the SRS, the synthetic read LRS-ICLR, LRS-10x, LRS-TELL-Seq, and OGM. In addition to detecting more SVs through LRS, longer reads map more uniquely to the genome than SRS. Despite using the default MAPQ0 settings employed in the current SRS/LRS aligners and variant callers that allow the use of misplaced reads, effective coverage of most genes across genomes was achieved for all SRS/LRS technologies. To address this issue, current SRS pipelines often mask these problematic dark regions, which include repetitive elements and polymorphic regions, potentially leading to the loss of key information in these regions. In addition, PacBio provides a BED file for dark regions that occur in repetitive areas or areas with high GC content. When stricter MAPQ1 or MAPQ50 was applied, many regions across the genome were not covered well in the SRS datasets, including numerous protein-coding and medically relevant genes. Of the technologies analysed, the lowest probability of mismatches was observed for the data obtained by LRS-PacBio HiFi, the highest

for SRS. The LRS-ONT datasets demonstrated a high percentage of low-quality reads due to lower nucleotide accuracy when using the MAPQ0 setting. When using MAPQ1 or MAPQ50 to filter out low-quality reads, uniform coverage across the whole genome with the high-quality reads and very long reads generated was achieved by LRS-ONT. Notably, LRS-ONT has introduced adaptive sampling, a computational enrichment technique that adjusts the sequencing parameters in the regions of interest [48, 49], enabling deeper coverage in these regions [50].

Another key step for clinical genetics is the introduction of the gapless T2T-CHM13 human reference assembly, which uncovers 8% of the dark regions of the genome, adds nearly 200 million bases and predicts 99 novel protein-coding genes compared with reference hg38 [51]. Our comprehensive bioinformatic analysis of whole-genome datasets further supports the clinical utility of using the T2T-CHM13 sequence for medical diagnostics. When we compared medically relevant genes using coordinates based on NCBI RefSeq annotations on both references, approximately 85% of the genes changed size and more than half by more than 10 bp. Notably, approximately 9% of the medically relevant genes differed by >1,000 bp between hg38 and T2T-CHM13. Among the genes significantly differing between both references were those that were disassembled, not correctly assembled or highly similar in hg38, such as the challenging medically relevant genes *GRK1*, *LPA*, *SMN1/2*, *DUX4* and *HLA-DRB5* or the *GBA* gene and its pseudogene *GBAP1* [52].

For example, GRCh38 contains only six copies of the 5.5 kb KIV-2 repeat in *LPA*, even though human genomes carry ~5 to >50 copies [33], omits the complete macrosatellite region containing >20 D4Z4 repeats in *DUX4* [53], introduces gaps and misassemblies within the approximately 500-kbp segmental duplication, omits Alu-mediated deletions and does not represent the variable *SMN1/SMN2* copy numbers [54]. Furthermore, the novel T2T-CHM13 reference resulted in approximately 20% more deletions and 20% fewer insertions than hg38. It should be noted that some of the observed differences in SV counts reflect reference bias, as sequences that are minor alleles in hg38 but common in human populations and thus represented in T2T-CHM13. Given that many disease-causing SVs are located in repetitive, duplicated, inverted, or structurally complex regions that were missing. disassembled or misinterpreted in older references and cannot be resolved using SRS [55], clinical genetics can benefit from the use of T2T-CHM13 and LRS. For example, T2T-CHM13 and LRS were used to detect previously hidden variants in undiagnosed cases of rare diseases within the European Solve-RD consortium [56], repeated expansions in cerebellar ataxia [57] and inversions disrupting the *EHMT1* gene in Kleefstra syndrome [58]. Similarly, T2T-CHM13 and LRS show promise in diseases associated with copy number variability, such as those known in the *SMN1/2* genes in spinal muscular atrophy [59] and the D4Z4 repeats in the *DUX4* gene in facioscapulohumeral muscular dystrophy [60]. Even in SRS data, the use of T2T-CHM13 improves read mapping and enhances the detection of clinically relevant rare and deleterious variants [61]. Taken together, there is growing evidence of the benefits of LRS and T2T-CHM13 for clinical medicine.

Another challenging topic is the pathogenicity annotations of thousands of SVs detected by LRS/SRS. Unlike sequence variants, for whose interpretation exist guidelines [62, 63] and clinical databases such as dbVar_common/ClinVar based on large available SRS datasets, SV annotations are more difficult not only due to the insufficient number of LRS datasets in the databases but also the substantial variability in SV breakpoints between individuals and the complexity of rearrangements. Therefore, we introduced crucial measures for SV evaluations, such as the distance in bp between SVs, the intersection factor and the size proportion for SVs in datasets, references and databases. Since most deletions in the ClinVar database cover very large regions containing many genes, a size proportion of 1% was used for LoReC annotations. Both LoReC and AnnotSV [38] have annotated many SVs using their own algorithm, but there are still many SVs that are not present in the current dbVar_common and ClinVar databases or other databases. It should be noted that for the majority of SVs per genome, many of which were located in protein-coding sequences, no annotation was available in the current version of the ClinVar database. Regarding insertions and other SVs, the length of the insertion and sequence of the insert are missing in the dbVar_common/ClinVar databases, making their annotation impossible. Unlike deletions, which remove annotated regions and can be assessed by their absence, insertions introduce sequences that are not present in the reference. Moreover, insertions frequently occur within, or generate, tandem repeats and segmental duplications, regions inherently difficult to assess. Consequently, insertions frequently lack reliable population frequency estimates and functional or phenotypic evidence, making confident clinical classification challenging [64]. Addressing these challenges will require systematic characterisation of insertion sequences and their functional annotation, precise breakpoint resolution and the development of tools for functional impact prediction. For SVs not annotated by ClinVar, we applied the LoReC toolkit, and, with its help, the affected gene(s) can be linked with gene/variant disease associations using DisGeNET, human phenotype ontology, or a similar database. In the future, this approach will allow SV annotations to be matched to continuously updated clinical databases based on the LRS datasets and linked to the reference T2T-CHM13.

This study and other [11, 12, 46, 50, 65, 66, 67] further support the introduction of LRS into medical diagnostics. However, LRS will not replace SRS in the near future but will complement it, especially in cases with negative SRS results, as this method will remain the gold standard for routine diagnostics due to its cost, speed and established clinical and bioinformatic pipelines. Nevertheless, to introduce LRS into diagnostics, laboratories should collect cells for high-molecular-weight DNA isolation, as LRS cannot be performed on fragmented DNA obtained by standard isolation methods and increases computational and data storage capacity due to the large datasets it obtains. We highlight the introduction of distance variance, intersection, gene overlap and the closest SV in the clinical database for SV comparisons and annotations, which is currently the weakest point of SV integration into clinical diagnostics. Although this first comprehensive study on the performance of all available genomic technologies is focused on SVs in the human genome, SV events are widespread in other species, and our toolkit is also suitable for these datasets.

Conclusions

In this study, we introduced an innovative multiplatform approach for any SRS and third-generation dataset that advances SV comparisons across samples and databases as well as annotations of SVs based on comparisons with clinical databases. Although the gold standard SRS uncovers thousands of SVs that may be clinically relevant, we demonstrated that LRS is more effective at detecting SVs than SRS. Thus, LRS is expected to complement the SRS analysis in clinical diagnostics soon, especially in cases with negative SRS results. However, the implementation of LRS will require the introduction of isolation methods leading to high-molecular-weight DNA and the update of clinical databases to include LRS datasets and the T2T-CHM13 reference for the correct annotations of SVs.

Declarations

Ethical Approval

The patients provided written informed consent for the use of their biological materials for the purpose of this study, which was conducted in accordance with the Helsinki Declaration and approved by the local ethics committee (NW25-03-00391).

Consent for publication

Not applicable

Competing Interests

The authors have no competing interests to declare.

Funding

This study was supported from European Regional Development Fund-Project "Interdisciplinary Approaches for the Development and Application of New Materials in Medical Practice - New Omic Technologies" (CZ.02.01.01/00/23_021/0009224), the Internal Grant Agency of Palacký University (JG_2025_035), and the Ministry of Health of the Czech Republic (NW24-10-00395, MH CZ – DRO (FNOL, 00098892).

Author's Contributions

JS and EK designed the research; TN and JS developed the LoReC toolkit; JS, TN, PG and MB performed the bioinformatic analysis; JM and AP performed the laboratory experiments; MM, TP and FC collected the patient samples and clinical characteristics; JS and EK wrote the manuscript; and PG and AP critically revised the manuscript. All authors read and approved the final manuscript.

Availability of Source Code and Requirements

Project name: lorec-comparator
Project homepage: https://github.com/novosadt/lorec-comparator
Operating system: Windows, Linux, macOS, Solaris
Programming language: Java 8
Other requirements: None
License: GPL-3.0 license
RRID: SCR_027211

Project name: lorec-coverage
Project homepage: https://github.com/novosadt/lorec-coverage
Operating system: Windows, Linux, macOS, Solaris
Programming language: Java 8
Other requirements: None
License: GPL-3.0 license
RRID: RRID:SCR_027210

Data availability

All additional supporting data are available in the GigaScience repository, GigaDB [31]

References

1. Collins RL, Talkowski ME. Diversity and consequences of structural variation in the human genome. Nature Reviews Genetics 2025 Jan;https://www.nature.com/articles/s41576-024-00808-9.
2. Logsdon GA, Vollger MR, Eichler EE. Long-read human genome sequencing and its applications. Nature Reviews Genetics 2020 Oct;21(10):597–614. https://www.nature.com/articles/s41576-020-0236-x.
3. Mahmoud M, Huang Y, Garimella K, Audano PA, Wan W, Prasad N, et al. Utility of long-read sequencing for All of Us. Nature Communications 2024 Jan;15(1):837. https://www.nature.com/articles/s41467-024-44804-3.
4. Sudmant PH, Rausch T, Gardner EJ, Handsaker RE, Abyzov A, Huddleston J, et al. An integrated map of structural variation in 2,504 human genomes. Nature 2015 Oct;526(7571):75–81. https://www.nature.com/articles/nature15394.
5. DeBoever C, Tanigawa Y, Lindholm ME, McInnes G, Lavertu A, Ingelsson E, et al. Medical relevance of protein-truncating variants across 337,205 individuals in the UK Biobank study. Nature Communications 2018 Apr;9(1):1612. https://www.nature.com/articles/s41467-018-03910-9.
6. Collins RL, Brand H, Karczewski KJ, Zhao X, Alföldi J, Francioli LC, et al. A structural variation reference for medical and population genetics. Nature 2020 May;581(7809):444–451. https://www.nature.com/articles/s41586-020-2287-8.
7. Kosugi S, Momozawa Y, Liu X, Terao C, Kubo M, Kamatani Y. Comprehensive evaluation of structural variation detection algorithms for whole genome sequencing. Genome Biology 2019 Jun;20(1):117. https://doi.org/10.1186/s13059-019-1720-5.
8. Escaramís G, Docampo E, Rabionet R. A decade of structural variants: description, history and methods to detect structural variation. Briefings in Functional Genomics 2015 Sep;14(5):305–314. https://doi.org/10.1093/bfgp/elv014.
9. Ahsan MU, Liu Q, Perdomo JE, Fang L, Wang K. A survey of algorithms for the detection of genomic structural variants from long-read sequencing data. Nature Methods 2023 Aug;20(8):1143–1158. https://www.nature.com/articles/s41592-023-01932-w.
10. Amarasinghe SL, Su S, Dong X, Zappia L, Ritchie ME, Gouil Q. Opportunities and challenges in long-read sequencing data analysis. Genome Biology 2020 Feb;21(1):30. https://doi.org/10.1186/s13059-020-1935-5.
11. Mantere T, Kersten S, Hoischen A. Long-Read Sequencing Emerging in Medical Genetics. Frontiers in Genetics 2019 May;10. https://www.frontiersin.org/journals/genetics/articles/10.3389/fgene.2019.00426/full.
12. Sanford Kobayashi E, Batalov S, Wenger AM, Lambert C, Dhillon H, Hall RJ, et al. Approaches to long-read sequencing in a clinical setting to improve diagnostic rate. Scientific Reports 2022 Oct;12(1):16945. https://www.nature.com/articles/s41598-022-20113-x.
13. Mahmoud M, Gobet N, Cruz-Dávalos DI, Mounier N, Dessimoz C, Sedlazeck FJ. Structural variant calling: the long and the short of it. Genome Biology 2019 Nov;20(1):246. https://doi.org/10.1186/s13059-019-1828-7.
14. OLGEN, OLGEN Resources;. http://olgen.cz/en/resources, accessed 16 February 2026.
15. Zook JM, Catoe D, McDaniel J, Vang L, Spies N, Sidow A, et al. Extensive sequencing of seven human genomes to characterize benchmark reference materials. Scientific Data 2016 Jun;3(1):160025. https://www.nature.com/articles/sdata201625.
16. Aganezov S, Goodwin S, Sherman RM, Sedlazeck FJ, Arun G, Bhatia S, et al. Comprehensive analysis of structural variants in breast cancer genomes using single-molecule sequencing. Genome Research 2020 Sep;30(9):1258–1273. http://genome.cshlp.org/content/30/9/1258, company: Cold Spring Harbor Laboratory Press Distributor: Cold Spring Harbor Laboratory Press Institution: Cold Spring Harbor Laboratory Press Label: Cold Spring Harbor Laboratory Press.
17. van Dijk EL, Naquin D, Gorrichon K, Jaszczyszyn Y, Ouazahrou

R, Thermes C, et al. Genomics in the long-read sequencing era. Trends in Genetics 2023 Sep;39(9):649–671. https://www.sciencedirect.com/science/article/pii/S0168952523001191.

18. Genome in a Bottle Consortium, Genome in a Bottle (GIAB) data for NA12878 (HG001);. https://ftp.ncbi.nlm.nih.gov/ReferenceSamples/giab/data/NA12878/, accessed 16 February 2026.
19. Kriegova E, Fillerova R, Minarik J, Savara J, Manakova J, Petrackova A, et al. Whole-genome optical mapping of bone-marrow myeloma cells reveals association of extramedullary multiple myeloma with chromosome 1 abnormalities. Scientific Reports 2021 Jul;11(1):14671. https://www.nature.com/articles/s41598-021-93835-z, number: 1.
20. Li H. Minimap2: pairwise alignment for nucleotide sequences. Bioinformatics 2018 Sep;34(18):3094–3100. https://doi.org/10.1093/bioinformatics/bty191.
21. Smolka M, Paulin LF, Grochowski CM, Horner DW, Mahmoud M, Behera S, et al. Detection of mosaic and population-level structural variants with Sniffles2. Nature Biotechnology 2024 Jan;p. 1–10. https://www.nature.com/articles/s41587-023-02024-y.
22. Marks P, Garcia S, Barrio AM, Belhocine K, Bernate J, Bharadwaj R, et al. Resolving the full spectrum of human genome variation using Linked-Reads. Genome Research 2019 Apr;29(4):635–645.
23. Helal AA, Saad BT, Saad MT, Mosaad GS, Aboshanab KM. Benchmarking long-read aligners and SV callers for structural variation detection in Oxford nanopore sequencing data. Scientific Reports 2024 Mar;14(1):6160. https://www.nature.com/articles/s41598-024-56604-2.
24. Li H, Durbin R. Fast and accurate short read alignment with Burrows-Wheeler transform. Bioinformatics (Oxford, England) 2009 Jul;25(14):1754–1760.
25. Chen X, Schulz-Trieglaff O, Shaw R, Barnes B, Schlesinger F, Källberg M, et al. Manta: rapid detection of structural variants and indels for germline and cancer sequencing applications. Bioinformatics 2016 Apr;32(8):1220–1222. https://doi.org/10.1093/bioinformatics/btv710.
26. Piñero J, Ramírez-Anguita JM, Saüch-Pitarch J, Ronzano F, Centeno E, Sanz F, et al. The DisGeNET knowledge platform for disease genomics: 2019 update. Nucleic Acids Research 2020 Jan;48(D1):D845–D855. https://doi.org/10.1093/nar/gkz1021.
27. Belyeu JR, Chowdhury M, Brown J, Pedersen BS, Cormier MJ, Quinlan AR, et al. Samplot: a platform for structural variant visual validation and automated filtering. Genome Biology 2021 May;22(1):161. https://doi.org/10.1186/s13059-021-02380-5.
28. Lappalainen I, Lopez J, Skipper L, Hefferon T, Spalding JD, Garner J, et al. dbVar and DGVa: public archives for genomic structural variation. Nucleic Acids Research 2013 Jan;41(D1):D936–D941. https://doi.org/10.1093/nar/gks1213.
29. National Center for Biotechnology Information (NCBI), dbVar: Database of Genomic Structural Variation;. https://www.ncbi.nlm.nih.gov/dbvar/, accessed 16 February 2026.
30. Genovese G, Rockweiler NB, Gorman BR, Bigdeli TB, Pato MT, Pato CN, et al. BCFtools/liftover: an accurate and comprehensive tool to convert genetic variants across genome assemblies. Bioinformatics 2024 Feb;40(2):btae038. https://doi.org/10.1093/bioinformatics/btae038.
31. Savara J, Novosad T, Gajdos P, Petrackova A, Behalek M, Manakova J, et al., Supporting data for "Multiplatform comparisons and annotation of structural variants highlight the utility of the T2T reference genome in human diagnostics". GigaScience Database; 2026. https://doi.org/10.5524/102806.
32. Xie H, Li W, Hu Y, Yang C, Lu J, Guo Y, et al. De novo assembly of human genome at single-cell levels. Nucleic Acids Research 2022 Jul;50(13):7479–7492. https://doi.org/10.1093/nar/gkac586.
33. Chin CS, Behera S, Metcalf GA, Gibbs RA, Boerwinkle E, Sedlazeck FJ, A pan-genome approach to decipher variants in the highly complex tandem repeat of LPA. bioRxiv; 2022. https://www.biorxiv.org/content/10.1101/2022.06.08.495395v2, pages: 2022.06.08.495395 Section: New Results.
34. Chen X, Harting J, Farrow E, Thiffault I, Kasperaviciute D, Hoischen A, et al. Comprehensive SMN1 and SMN2 profiling for spinal muscular atrophy analysis using long-read PacBio HiFi sequencing. American Journal of Human Genetics 2023 Feb;110(2):240–250. https://www.ncbi.nlm.nih.gov/pmc/articles/PMC9943720/.
35. Yang X, Wang X, Zou Y, Zhang S, Xia M, Fu L, et al. Characterization of large-scale genomic differences in the first complete human genome. Genome Biology 2023 Jul;24(1):157. https://doi.org/10.1186/s13059-023-02995-w.
36. Mandelker D, Schmidt RJ, Ankala A, McDonald Gibson K, Bowser M, Sharma H, et al. Navigating highly homologous genes in a molecular diagnostic setting: a resource for clinical next-generation sequencing. Genetics in Medicine 2016 Dec;18(12):1282–1289. https://www.sciencedirect.com/science/article/pii/S1098360021014258.
37. Landrum MJ, Lee JM, Benson M, Brown GR, Chao C, Chitipiralla S, et al. ClinVar: improving access to variant interpretations and supporting evidence. Nucleic Acids Research 2018 Jan;46(D1):D1062–D1067. https://doi.org/10.1093/nar/gkx1153.
38. Geoffroy V, Herenger Y, Kress A, Stoetzel C, Piton A, Dollfus H, et al. AnnotSV: an integrated tool for structural variations annotation. Bioinformatics 2018 Oct;34(20):3572–3574. https://doi.org/10.1093/bioinformatics/bty304.
39. Wojcik MH, Reuter CM, Marwaha S, Mahmoud M, Duyzend MH, Barseghyan H, et al. Beyond the exome: What's next in diagnostic testing for Mendelian conditions. The American Journal of Human Genetics 2023 Aug;110(8):1229–1248. https://www.sciencedirect.com/science/article/pii/S0002929723002100.
40. Merker JD, Wenger AM, Sneddon T, Grove M, Zappala Z, Fresard L, et al. Long-read genome sequencing identifies causal structural variation in a Mendelian disease. Genetics in Medicine 2018 Jan;20(1):159–163. https://www.sciencedirect.com/science/article/pii/S1098360021019328.
41. van Belzen IAEM, Schönhuth A, Kemmeren P, Hehir-Kwa JY. Structural variant detection in cancer genomes: computational challenges and perspectives for precision oncology. npj Precision Oncology 2021 Mar;5(1):1–11. https://www.nature.com/articles/s41698-021-00155-6.
42. Duan DM, Cheng C, Huang YS, Chung Ak, Chen PX, Chen YA, et al. Comparisons of performances of structural variants detection algorithms in solitary or combination strategy. PLOS ONE 2025;20(2):e0314982. https://journals.plos.org/plosone/article?id=10.1371/journal.pone.0314982.
43. Liu Z, Roberts R, Mercer TR, Xu J, Sedlazeck FJ, Tong W. Towards accurate and reliable resolution of structural variants for clinical diagnosis. Genome Biology 2022 Mar;23(1):68. https://doi.org/10.1186/s13059-022-02636-8.
44. Kirsche M, Prabhu G, Sherman R, Ni B, Battle A, Aganezov S, et al. Jasmine and Iris: population-scale structural variant comparison and analysis. Nature Methods 2023 Mar;20(3):408–417. https://www.nature.com/articles/s41592-022-01753-3.
45. Jeffares DC, Jolly C, Hoti M, Speed D, Shaw L, Rallis C, et al. Transient structural variations have strong effects on quantitative traits and reproductive isolation in fission yeast. Nature Communications 2017 Jan;8(1):14061. https://www.na

ture.com/articles/ncomms14061.
46. Wagner J, Olson ND, Harris L, Khan Z, Farek J, Mahmoud M, et al. Benchmarking challenging small variants with linked and long reads. Cell Genomics 2022 May;2(5).
47. Zook JM, Hansen NF, Olson ND, Chapman L, Mullikin JC, Xiao C, et al. A robust benchmark for detection of germline large deletions and insertions. Nature Biotechnology 2020 Nov;38(11):1347–1355. https://www.nature.com/articles/s41587-020-0538-8.
48. Lu H, Giordano F, Ning Z. Oxford Nanopore MinION Sequencing and Genome Assembly. Genomics, Proteomics & Bioinformatics 2016 Oct;14(5):265–279. https://www.sciencedirect.com/science/article/pii/S1672022916301309.
49. Martin S, Heavens D, Lan Y, Horsfield S, Clark MD, Leggett RM. Nanopore adaptive sampling: a tool for enrichment of low abundance species in metagenomic samples. Genome Biology 2022 Jan;23(1):11. https://doi.org/10.1186/s13059-021-02582-x.
50. Oehler JB, Wright H, Stark Z, Mallett AJ, Schmitz U. The application of long-read sequencing in clinical settings. Human Genomics 2023 Aug;17(1):73. https://doi.org/10.1186/s40246-023-00522-3.
51. Rhie A, Nurk S, Cechova M, Hoyt SJ, Taylor DJ, Altemose N, et al. The complete sequence of a human Y chromosome. Nature 2023 Sep;621(7978):344–354. https://www.nature.com/articles/s41586-023-06457-y.
52. Nurk S, Koren S, Rhie A, Rautiainen M, Bzikadze AV, Mikheenko A, et al. The complete sequence of a human genome. Science 2022 Apr;376(6588):44–53. https://www.science.org/doi/10.1126/science.abj6987.
53. Grobecker P, Berri S, Peden JF, Chow KJ, Fielding C, Armogida I, et al. A dedicated caller for DUX4 rearrangements from whole-genome sequencing data. BMC Medical Genomics 2025 Jan;18(1):24. https://doi.org/10.1186/s12920-024-02069-1.
54. Logsdon GA, Ebert P, Audano PA, Loftus M, Porubsky D, Ebler J, et al. Complex genetic variation in nearly complete human genomes. Nature 2025 Aug;644(8076):430–441. https://www.nature.com/articles/s41586-025-09140-6.
55. Gobbo GFD, Boycott KM. The additional diagnostic yield of long-read sequencing in undiagnosed rare diseases. Genome Research 2025 Apr;35(4):559–571. http://genome.cshlp.org/content/35/4/559, company: Cold Spring Harbor Laboratory Press Distributor: Cold Spring Harbor Laboratory Press Institution: Cold Spring Harbor Laboratory Press Label: Cold Spring Harbor Laboratory Press.
56. Steyaert W, Sagath L, Demidov G, Yépez VA, Esteve-Codina A, Gagneur J, et al. Unraveling undiagnosed rare disease cases by HiFi long-read genome sequencing. Genome Research 2025 Apr;35(4):755–768. http://genome.cshlp.org/content/35/4/755, company: Cold Spring Harbor Laboratory Press Distributor: Cold Spring Harbor Laboratory Press Institution: Cold Spring Harbor Laboratory Press Label: Cold Spring Harbor Laboratory Press.
57. Rafehi H, Fearnley LG, Read J, Snell P, Davies KC, Scott L, et al. A prospective trial comparing programmable targeted long-read sequencing and short-read genome sequencing for genetic diagnosis of cerebellar ataxia. Genome Research 2025 Apr;35(4):769–785. http://genome.cshlp.org/content/35/4/769, company: Cold Spring Harbor Laboratory Press Distributor: Cold Spring Harbor Laboratory Press Institution: Cold Spring Harbor Laboratory Press Label: Cold Spring Harbor Laboratory Press.
58. Saether KB, Eisfeldt J, Bengtsson JD, Lun MY, Grochowski CM, Mahmoud M, et al. Leveraging the T2T assembly to resolve rare and pathogenic inversions in reference genome gaps. Genome Research 2024 Nov;34(11):1785–1797. http://genome.cshlp.org/content/34/11/1785, company: Cold Spring Harbor Laboratory Press Distributor: Cold Spring Harbor Laboratory Press Institution: Cold Spring Harbor Laboratory Press Label: Cold Spring Harbor Laboratory Press.
59. Zwartkruis MM, Elferink MG, Gommers D, Signoria I, Blasco-Pérez L, Costa-Roger M, et al. Long-read sequencing identifies copy-specific markers of SMN gene conversion in spinal muscular atrophy. Genome Medicine 2025 Mar;17(1):26. https://doi.org/10.1186/s13073-025-01448-2.
60. Huang M, Zhang Q, Jiao J, Shi J, Xu Y, Zhang C, et al. Comprehensive genetic analysis of facioscapulohumeral muscular dystrophy by Nanopore long-read whole-genome sequencing. Journal of Translational Medicine 2024 May;22(1):451. https://doi.org/10.1186/s12967-024-05259-8.
61. Schmitz D, Ameur A, Johansson A. T2T-CHM13 improves read mapping and detection of clinically relevant genetic variation in the Swedish population. Genome Research 2025 Nov;35(11):2377–2388. http://genome.cshlp.org/content/35/11/2377, company: Cold Spring Harbor Laboratory Press Distributor: Cold Spring Harbor Laboratory Press Institution: Cold Spring Harbor Laboratory Press Label: Cold Spring Harbor Laboratory Press.
62. Richards S, Aziz N, Bale S, Bick D, Das S, Gastier-Foster J, et al. Standards and guidelines for the interpretation of sequence variants: a joint consensus recommendation of the American College of Medical Genetics and Genomics and the Association for Molecular Pathology. Genetics in Medicine 2015 May;17(5):405–424. https://www.gimjournal.org/article/S1098-3600%2821%2903031-8/fulltext.
63. Miller DT, Lee K, Abul-Husn NS, Amendola LM, Brothers K, Chung WK, et al. ACMG SF v3.2 list for reporting of secondary findings in clinical exome and genome sequencing: A policy statement of the American College of Medical Genetics and Genomics (ACMG). Genetics in Medicine 2023 Aug;25(8). https://www.gimjournal.org/article/S1098-3600(23)00879-1/fulltext.
64. Nicholas TJ, Cormier MJ, Quinlan AR. Annotation of structural variants with reported allele frequencies and related metrics from multiple datasets using SVAFotate. BMC Bioinformatics 2022 Nov;23(1):490. https://doi.org/10.1186/s12859-022-05008-y.
65. Olivucci G, Iovino E, Innella G, Turchetti D, Pippucci T, Magini P. Long read sequencing on its way to the routine diagnostics of genetic diseases. Frontiers in Genetics 2024 Mar;15. https://www.frontiersin.org/journals/genetics/articles/10.3389/fgene.2024.1374860/full.
66. Negi S, Stenton SL, Berger SI, Canigiula P, McNulty B, Violich I, et al. Advancing long-read nanopore genome assembly and accurate variant calling for rare disease detection. The American Journal of Human Genetics 2025 Feb;112(2):428–449. https://www.sciencedirect.com/science/article/pii/S0002929725000023.
67. Savara J, Novosád T, Gajdoš P, Kriegová E. Comparison of structural variants detected by optical mapping with long-read next-generation sequencing. Bioinformatics 2021 Oct;37(20):3398–3404. https://doi.org/10.1093/bioinformatics/btab359.

Manuscript

Click here to access/download;Manuscript;26_03_03_GIGA_D_25_00250_R

Placeholder for OUP logo oup.pdf

GigaScience, 2023, 1–12

doi: xx.xxxx/xxxx
Manuscript in Preparation
Research

RESEARCH

Multiplatform comparisons and annotation of structural variants highlight the utility of the T2T reference genome in human diagnostics

Jakub Savara [0000-0003-3067-7867][1,2], Tomas Novosad [0000-0003-2291-261X][2], Petr Gajdos [0000-0003-1831-3489][2], Anna Petrackova [0000-0001-5210-4504][1], Marek Behalek [0000-0002-9204-2691][2], Jirina Manakova [0000-0001-6931-5666][1], Filip Ctvrtlik [0000-0003-1490-7175][3], Jiri Minarik [0000-0003-0513-326X][4], Tomas Papajik [0000-0001-5779-1139][4] and Eva Kriegova [0000-0002-8969-4197][1]

[1]Department of Immunology, Faculty of Medicine and Dentistry, Palacký University Olomouc and University Hospital Olomouc, Olomouc, Czech Republic and [2]Department of Computer Science, Faculty of Electrical Engineering and Computer Science, VSB-Technical University of Ostrava, Ostrava, Czech Republic and [3]Department of Radiology, Faculty of Medicine and Dentistry, Palacký University Olomouc and University Hospital Olomouc, Olomouc, Czech Republic and [4]Department of Hemato-oncology, Faculty of Medicine and Dentistry, Palacký University Olomouc and University Hospital Olomouc, Olomouc, Czech Republic

Abstract

Background: Structural variants (SVs) are increasingly recognised as key contributors to human diseases. However, our understanding of SVs in health and disease is limited, mainly due to their structural complexity and variable length in individuals as well as limitations inherent to the available genomic technologies and reference genome used. **Results:** To systematically evaluate SVs across human whole-genome samples using hg38/GRCh38 and gapless T2T-CHM13 references, we introduced an innovative multiplatform approach, LongReadChecker (LoReC), which advances SV comparison and annotation based on distance variance, intersection, gene overlap and the closest SV in the clinical database. Comparison of the performance in detecting SVs from public and our own whole-genome datasets from short-read sequencing (SRS), available long-read sequencing (LRS) platforms and optical genome mapping (OGM) revealed that most SVs detected by SRS were confirmed by LRS, but LRS can identify twice as many SVs (25,000 SVs/genome) with greater read mapping accuracy. Our LoReC analysis further highlights the utility of the T2T-CHM13 reference in SV detection, as 20% more deletions and 20% less insertions were detected compared with hg38/GRCh38, which was particularly evident in long-read datasets. Since 80% of the SVs detected by LRS/SRS are smaller than 0.5 kbp, OGM did not detect them. **Conclusions:** Our study revealed that introducing distance variance, intersection, gene overlap and the closest SV in the clinical database may help compare and annotate SVs in diagnostics. Our data showed that LRS together with T2T-CHM13 gapless sequences can improve the diagnostics of patients with human diseases when SRS fails to identify the cause.

Key words: Next-generation sequencing; structural variants; annotations; LongReadChecker (LoReC) toolkit; long-read technology

Compiled on: March 4, 2026.
Draft manuscript prepared by the author.

Introduction

Structural variants (SVs) are a major source of human genetic diversity and arise from the breakdown and rejoining of DNA fragments, which can lead to the loss, gain and rearrangement of genes and regulatory elements [1]. Since SVs are larger than 50 bp and can affect thousands to millions of nucleotides [2, 3], they are expected to have a strong effect on transcriptional regulation in health and disease [4]. Structural variant detection is of particular importance, as they are responsible for more than 25% of all rare protein truncations in a genome and are associated with many diseases [5, 6]. However, SV characterisation and functional interrogation have largely lagged behind single nucleotide variations and small insertions and deletions (INDELs), mainly due to their structural complexity and variable length in individuals as well as limitations inherent to the available genomic technologies [7]. Traditionally, SVs in the human genome have been detected using array-based methods or locus-specific assays for targeted regions [8].

Currently, great progress is being made in the detection of SVs using short-read sequencing (SRS), which remains essential due to practical and cost considerations in clinical diagnostics [9]. Novel genomic technologies for SV detection are also being rapidly developed. Among them, long-read sequencing (LRS) has demonstrated a high potential for detecting SVs [10] through longer reads and increased accuracy compared with SRS [1]. The increasing throughput, lower prices and portability of LRS technologies increase the potential of LRS introduction into diagnostic testing, particularly for patients with genetic disorders with negative results using SRS [11, 12]. However, accurate and precise identification of SVs in specific samples and/or across samples is challenging [13]. In addition, data on the performance and comparison of currently available genomic technologies, the clinical utility of the novel human reference assembly T2T-CHM13 (T2T) and clinical databases for the annotation of detected SVs are incomplete.

Therefore, this study focused on conducting a comprehensive comparison of SVs detected from whole-genome datasets, both public and our own, obtained using SRS, LRS from currently available technologies and optical genome mapping (OGM). To achieve this, we introduced an innovative bioinformatics approach LongReadChecker (LoReC)[14] enabling comparisons of SVs across whole-genome datasets, technologies and reference genomes, including their annotations using clinical genomic databases. This multi-platform approach revolutionizes the comparisons of SVs by introducing key parameters such as distance variance, intersection, and gene overlap between datasets, thus advancing their comparison and annotation across samples or technologies or clinical databases. Our study further highlights the utility of T2T reference and long-read technologies in clinical and research applications.

Materials and methods

Public and our own human whole-genome datasets

This study evaluated whole-genome datasets from a human DNA standard (NA12878 cell line, also known as HG001; B-lymphocyte; female; healthy; Genome in a Bottle Consortium (GIAB))[15] and a breast cancer cell line (SKBR3)[16] obtained by i) traditional SRS (Illumina, CA, USA), ii) true LRS on a single-molecule real-time platform from Pacific Biosciences (LRS-PacBio), iii) true LRS from Oxford Nanopore Technologies (LRS-ONT), iv) synthetic LRS from transposase enzyme-linked LRS (LRS-TELL-Seq; Universal Sequencing Technology, MA, USA), v) synthetic LRS from Illumina Complete Long-Reads (LRS-ICLR; Illumina) and vi) synthetic LRS from 10× assay (LRS-10×; 10× Genomics). All synthetic LRS were sequenced on an Illumina short-read platform. In addition, OGM from Bionano Genomics (CA, USA) was used (Supplementary Table 1). The principles of the LRS technologies used are described elsewhere [17]. Our own datasets from SRS, LRS-TELL-Seq, LRS-ICLR and OGM were obtained for two diagnostic samples, P3 (pheochromocytoma adrenal medulla tissue, man, 63 years) and P48 (enriched CD138+ myeloma cells from bone marrow aspirate, woman, 39 years, IgG lambda, stage IIIA, ISS II), as well as for the NA12878 cell line. Raw sequencing data for SKBR3 cell lines for different technologies are available within the SRA under BioProject PRJNA476239 [16]. Raw sequencing data for NA12878 are available within GIAB FTP release and under BioProject PRJNA200694 [15, 18].

For our own analysis, the high-molecular-weight DNA from tumour tissues and cells from cell lines was isolated from agarose plugs as reported previously [19]. Next-generation sequencing (NGS) libraries were prepared according to the manufacturer recommendations for LRS-ICLR, LRS-TELL-Seq™ and SRS TruSeq DNA PCR-Free (Illumina) and sequenced (150 bp paired-end reads) on a NovaSeq 6000 system (Illumina). In addition, OGM labelling and measurements using the Bionano Saphyr instrument (Bionano Genomics) were performed as reported previously [19]. The sequence depth for LRS and SRS was approximately 30× and for OGM 300×.

Bioinformatic processing of whole-genome datasets from different short-read sequencing and long-read sequencing platforms

To minimise the difference in precision, recall and F1-score metrics using different callers/aligners, we used the LRS aligner Minimap2 [20] and the SV caller Sniffles2 (v2.2) software [21] for true LRS and the LongRanger software (v2.2.2) [22] for synthetic LRS analyses, which provided a strong basis for SV pipeline calling in LRS [23]. Moreover, this combination is the basis for the Illumina DRAGEN analysis of Illumina LRS-ICLR, an approach that was also compared in this study. For SRS datasets, BWA aligner software (v0.7.17) [24] and the Manta structural variant caller (v1.6.0) [25] were used. Raw data from OGM were analysed using Bionano Access (v1.8) software by Bionano Genomics, and the *De Novo* assembly pipeline was performed using Bionano Solve tools (v3.8) (Bionano Genomics). The hg38 (GRCh38.p14) and T2T-CHM13 (v2.0) human reference genomes were used. Gene and pseudogene coordinates, names and biotypes (e.g. protein coding) are based on MANE transcripts from RefSeq NCBI annotations (version 110); in all analyses, the Y chromosome and alternate (ALT) contigs were excluded. The list of medically relevant genes is derived from the DisGeNET [26] database (DisGeNET v20.1), which includes information on gene/variant-disease associations (VDAs) originating from ClinVar, the GWAS Catalog, UniProt, GAD and BeFree data [26]. The selection of medically relevant genes is based on gene disease associations (GDAs) and VDAs, GDA/VDA > 0.5 and evidence index > 0.8, indicating that most publications support GDA/VDA [26].

Comparison of structural variants using the LongReadChecker toolkit

For comparison of SVs across different samples, technologies, and databases, we designed our own LoReC toolkit, containing two tools: the LoReC comparator (source file: variant call format, vcf) and LoReC coverage (source file: mapped reads in binary format, bam). The LoReC comparator can find the closest SV detected by another technology, database or reference genome for each SV across the whole genome or region(s) of interest. For each comparison, the following parameters need to be established: 1) the distance variance threshold (the accepted difference in bp between

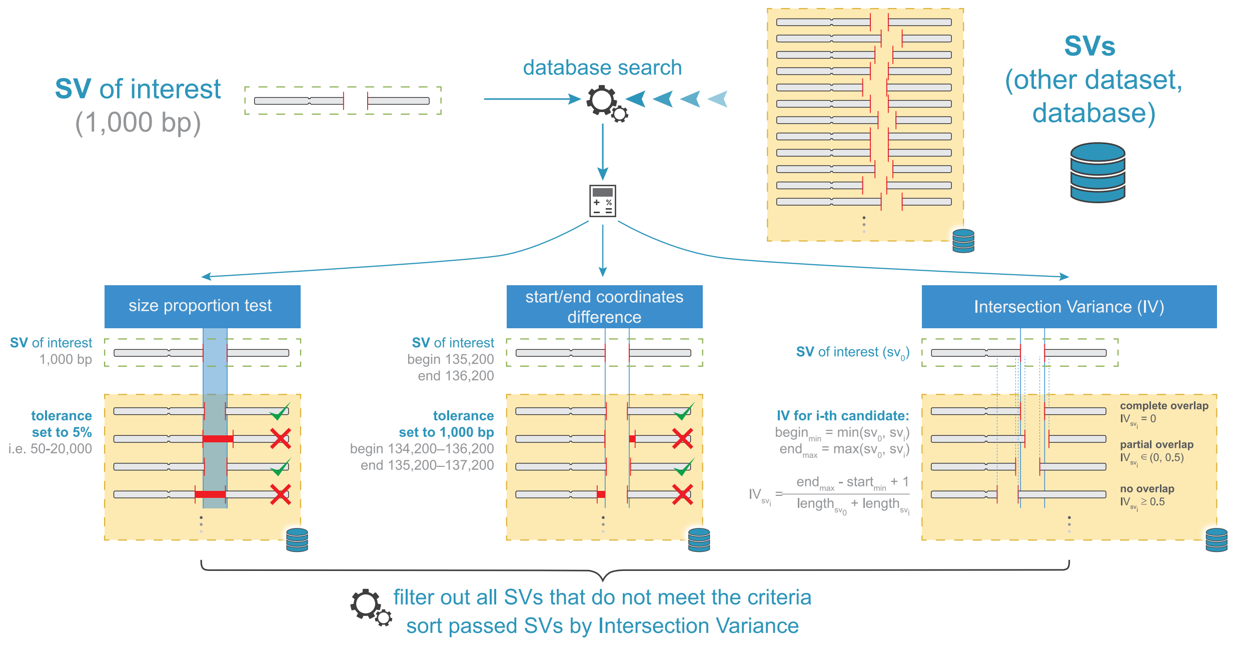

Fig. 1. Annotation of structural variants (SVs) detected in the short-read and long-read sequencing datasets in clinical diagnostics using the LoReC toolkit. Due to the high variability in SV breakpoints, three parameters are needed for the SV comparison with SVs from clinical databases, such as the distance in bp between the sample SV and the database SV (start and end coordinate difference), the intersection (intersection factor) and the minimum overlap between the sample SV(s) and the database SV(s) (size proportion).

the start and end coordinates between each SV in dataset 1 and the nearest SV in dataset 2, expressed as the sum of the two differences), 2) the intersection factor (the overlap between each SV in dataset 1 and the nearest SV in dataset 2; 0 = 100% overlap, 0–0.5 = partial overlap, >0.5 = no overlap) and 3) the minimal size proportion (the percentage of the size in bp between each SV in dataset 1 and the nearest SV in dataset 2; e.g. 5% means that the SVs in dataset 1 encompass at least 5% of the SVs in dataset 2 or vice versa). To compare SVs in the same regions detected by different technologies, which may differ in size and coordinates because of the different principles of the method, the LoReC toolkit lists the closest SV of the same type (e.g. deletion, insertion) from dataset 2 and its coordinates, distance, intersection factor and size proportion compared with the SV from dataset 1. A distance variance threshold of 1,000 bp was used for a comparison between different NGS platforms and 50,000 bp between NGS and OGM, and an intersection factor of 0 to 0.5 and a minimum size fraction of 5% were used to indicate SVs that overlap and are very similar; different parameters can be used for filtering (Fig. 1). To visually inspect the SVs of interest, the Samplot tool was used [27].

The LoReC coverage tool is able to provide coverage of the gene/region of interest, including statistics (mean, median, min, max, Q1 and Q3 coverages), filter reads based on the mapping quality and visualisation of the regions of interest. The LoReC coverage outputs are as follows: 1) coverage across regions of interest or across the whole genome based on the coordinates and gene names given in a region file; 2) coverage calculations based on the read mapping quality (MAPQ) threshold value, which is able to filter out the reads that map to multiple regions or those of poor quality (MAPQ0 = high probability that a read is mapped to multiple locations with an equal score, MAPQ1 = high probability that a read is mapped to at least two locations, MAPQ50 = 99.999% probability that a read is mapped to a unique region; Fig. 2); and 3) visualisation of the regions of interest specified in the region files, which allows a comparison of multiple technologies, different samples or reference genomes. As low coverage genes are marked those in which Q1 coverage was below 25% of the mean genome coverage of the sample.

Annotation of structural variants using the LongReadChecker toolkit

Another functionality of the LoReC comparator is to annotate detected SVs based on the annotation file(s) from dbVar (NCBI)[28, 29]. As the current dbVar_common and ClinVar databases are based on hg38, SV annotations were performed primarily on this reference. For experimental purposes, dbVar_common and ClinVar annotation files were converted from hg38 to the CHM13-T2T reference genome using BCFtools/liftover [30]. The following parameters were established for SV comparisons: a threshold of ±1,000 bp in the distance between the start and end coordinates of the SVs compared, an intersection factor threshold of 0.5 and a minimal size proportion of 1%. First, the detected SVs are compared with SVs included in the dbVar_common database of common SVs found with a frequency >1% in the population (e.g. nstd186, NCBI Curated Common Structural Variants) or any other vcf file of interest and must fulfil the set-up criteria to be marked as PASS. Second, detected SVs not found in the dbVar_common database are compared with the ClinVar SV database (e.g. summary of nstd102, Clinical Structural Variants) or vcf of interest, and for SVs present in the database, the clinical significance of SVs is reported (e.g. pathogenetic, likely pathogenic, variant of uncertain significance [VUS], likely benign, benign). For each SV, LoReC provides the main annotation (ClinVar accession ID) with the lowest intersection factor (=highest overlap) that passed the selected filters. In addition, LoReC provides all passed ClinVar accession IDs which meet filtering criteria for further investigation. For each gene(s), it is possible to filter their SVs in output tables generated by LoReC toolkit. For SVs not found in the ClinVar SV database and with an overlap of at least one gene, additional information is reported through the LoReC toolkit based on the NCBI annotation file, which includes the biotype, gene description and gene/VDAs present in DisGeNET, allowing filtering based on these features [31].

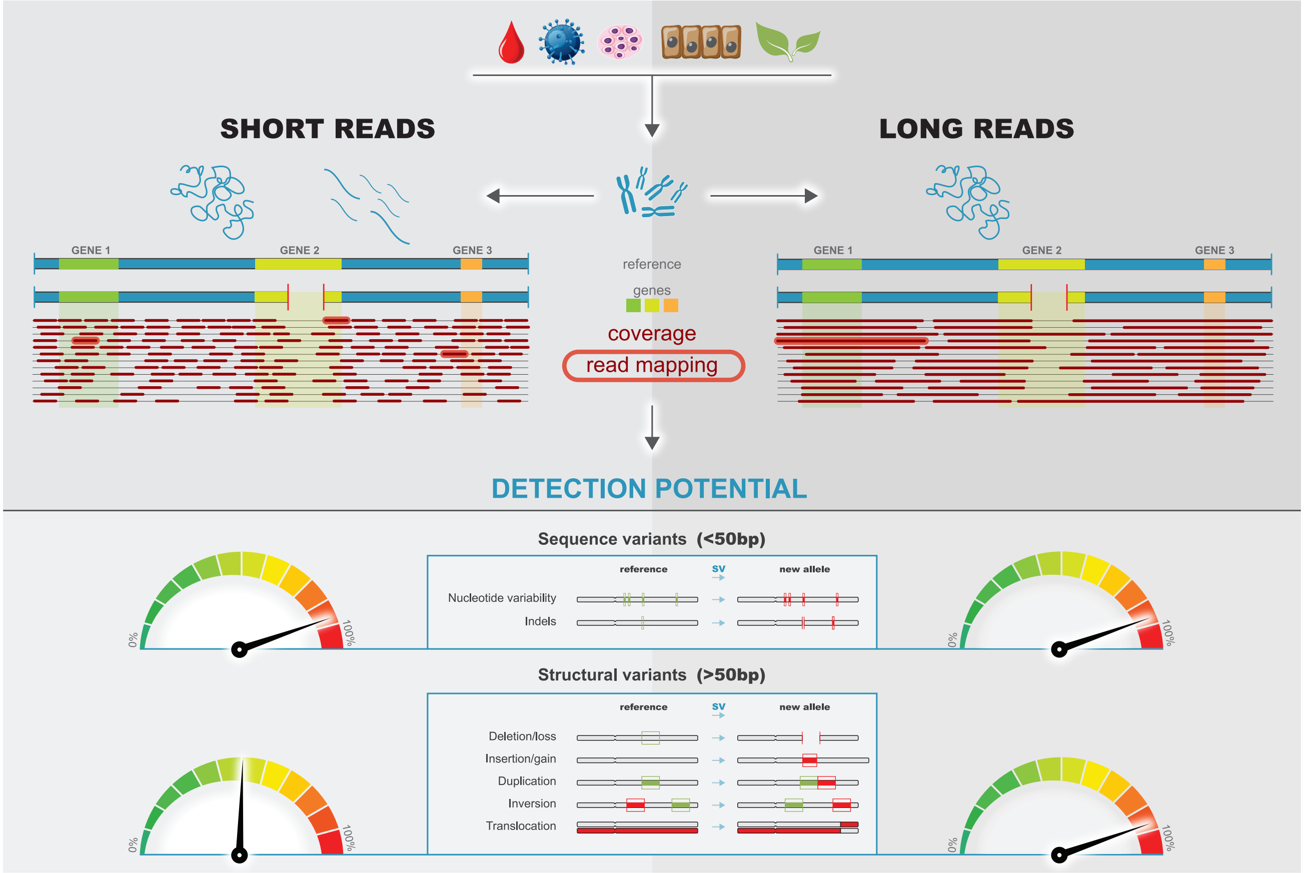

Fig. 2. Principle of short-read sequencing (SRS) and long-read sequencing (LRS) and their detection potential for sequence and structural variants. Example of the coverage and read mapping for a heterozygous gene deletion is shown; LRS requires DNA with higher molecular weight than SRS. The lower diagram shows the detection potential for sequence and structural variants using SRS and LRS.

Results

True long-read sequencing technologies have superior performance in detecting structural variants

To evaluate the performance of the currently available LRS technologies and SRS, whole-genome sequencing datasets for the NA12878 healthy [15] and SKBR3 breast cancer [16] cell lines and two diagnostic tissue samples, P3 (pheochromocytoma tissue) and P48 (multiple myeloma bone marrow aspirate), both public and our own (Supplementary Table 1), were compared using different LRS and SRS platforms. Among the tested platforms were the following: 1) two true LRS technology (LRS-PacBio and LRS-ONT) instruments; 2) three synthetic linked-read LRS approaches (LRS-ICLR, LRS-10× and LRS-TELL-Seq), sequenced on the short-read Illumina platform; 3) SRS on Illumina; and 4) OGM from Bionano Genomics. For comparisons of detected SVs, their types, coordinates, sizes, intersection factors, size proportions and coverage between different technologies, reference genomes (hg38/GRCh38.p14; T2T-CHM13, v2.0) and annotations of detected SVs according to clinical databases, a LoReC toolkit was used (Fig. 1). For more details on functionalities, see the Materials and Methods Section.

Using hg38, approximately 25,000 SVs per genome were detected using LRS-PacBio and LRS-ONT, 14,000–15,000 using LRS-ICLR, 10,000–12,500 using LRS-10×, 12,500–15,000 using SRS and 4,000 using OGM. Insertions were the most common SVs detected by all technologies: 14,000 per genome using LRS-PacBio and LRS-ONT, 5,000–6,000 using LRS-ICLR, 6,000–7,000 using SRS and 2,500 using OGM. Insertions were not detected in the LRS-TELL-Seq and LRS-10× datasets, as the LongRanger pipeline available to analyse synthetic reads cannot call insertions. Deletions were the second most common SVs: 10,500 per genome using LRS-PacBio and LRS-ONT, 9,000–10,000 using LRS-ICLR, 3,500–5,000 using LRS-10×, 5,000–6,000 using SRS and 1,250 using OGM. Regarding other SVs, such as inversions, duplications and breakends/translocations, their counts varied across samples and genomes, ranging from 240 to 1,430 per genome, depending on the technology (Supplementary Table 2, Fig. 3, Supplementary Fig. 1). Selected SVs from the OGM and LRS datasets were verified using fluorescence immunophenotyping and interphase cytogenetics as a tool for the investigation of neoplasms (FICTION), arrayCGH and/or targeted SRS.

When comparing available technologies, the majority of deletions and insertions (~80%–95%) detected by LRS-ONT were confirmed by LRS-PacBio, whereas higher concordance (~95%) was observed using the PacBio high fidelity (HiFi) sequencing mode and lower concordance (~80%) using continuous long reads. However, less than 50% were detected by SRS and 55%–71% by LRS-ICLR. Furthermore, LRS-TELL-Seq and LRS-10x confirmed approximately 25%–50% of the deletions detected by LRS-ONT/LRS-PacBio, but it was not possible to call insertions from synthetic reads. Regarding inversions, duplications and breakends/translocations, the best overlap was observed between LRS-PacBio and LRS-ONT, and a moderate overlap was identified using synthetic LRS and SRS (Supplementary Tables 3 and 4).

Most structural variants detected by short-read sequencing were confirmed by long-read sequencing

Next, we compared the SVs detected by SRS, the most widely used platform today in clinical diagnostics, with SVs detected

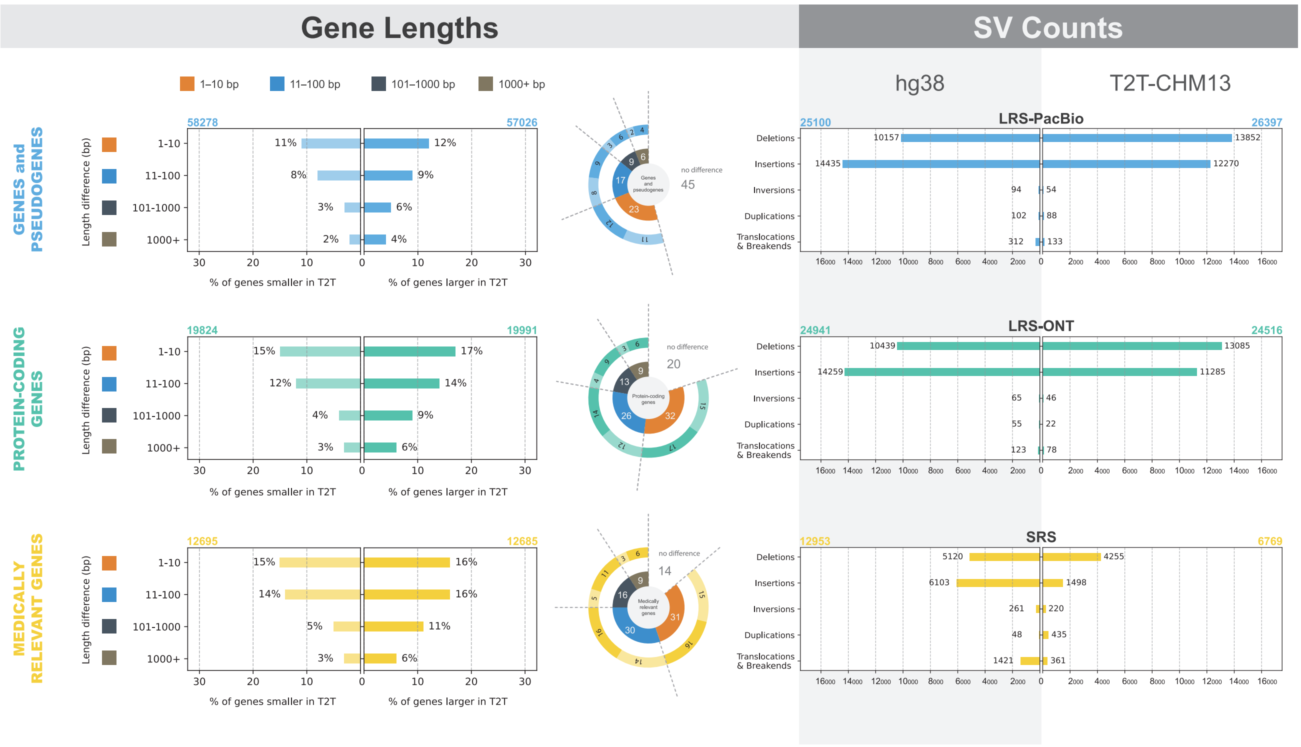

Fig. 3. Comparison of gene lengths and the number of structural variants (SVs) in the NA12878 cell line using hg38 and T2T-CHM13 references. The differences in the number and lengths of genes and pseudogenes, protein-coding genes and medically relevant genes for both references are presented; genes were annotated based on RefSeq NCBI (version 110, based on gene IDs and MANE transcript), excluding genes on the Y chromosome and ALT contigs. The number of SVs detected by long-read sequencing (LRS-PacBio, LRS-ONT) and short-read sequencing (SRS) on both references are shown on the right.

by LRS-ONT and LRS-PacBio and synthetic LRS. Most of the deletions and insertions detected by SRS (10,000–12,500 per genome) were smaller than 0.5 kbp (~80%) and were confirmed by LRS-PacBio/LRS-ONT (~90%). Although LRS-ICLR was similar to true LRS in detecting deletions, it had less precision in the case of insertions (~50%), as this technology is based on SRS. Notably, SRS failed to detect approximately 50% of the SVs detected by LRS (Supplementary Tables 3 and 5).

True long-read sequencing and short-read sequencing technologies have superior performance over optical genome mapping

We also compared LRS and SRS with OGM, a non-sequencing technology based on the labelling of high-molecular-weight DNA using fluorophore tags on specific sequence motifs. As OGM does not detect SVs smaller than 0.5 kbp or SVs in genomic regions that lack specific sequence motifs, OGM detected a lower number of SVs than LRS or SRS (hg38: 3,946 vs 24,941 vs 12,953; T2T-CHM13: 3,082 vs 24,516 vs 6,769). The majority (~80%) of deletions and insertions detected by OGM were confirmed by LRS-ONT and LRS-PacBio; SRS confirmed approximately 35% of the insertions and approximately 50%–60% of the deletions detected by OGM (Supplementary Tables 3 and 6). Comparison of OGM with LRS and SRS showed that OGM does not provide the exact coordinates of individual SVs, and their position may be substantially different from coordinates detected by LRS and SRS (Supplementary Fig. 2).

True long reads map with high probability to unique regions compared with short reads

Next, we evaluated the MAPQ [24], a measure of the probability that a read is misplaced, for different LRS and SRS technologies. For MAPQ0, a default setting in the current SRS/LRS aligners and variant callers that allows read mapping to multiple regions, most genes were covered by all SRS/LRS technologies (Fig. 2, Supplementary Fig. 3, Supplementary Table 7). To eliminate problematic regions with misplaced reads, current SRS pipelines often mask these repetitive dark regions. When stricter MAPQ1 and MAPQ50 (associated with a lower probability of misplaced reads than MAPQ0) were applied, many regions of the genome were not covered in the SRS datasets, including many protein-coding genes and medically relevant genes (Fig. 2, Supplementary Fig. 3, Supplementary Table 7). In LRS-PacBio HiFi datasets, most reads were mapped with high probability to a unique region, as demonstrated by applying MAPQ1/MAPQ50. Regarding LRS-ONT, mapping to multiple regions is less probable because of the very long reads (up to Mbp); however, many reads are of low quality, resulting in less accuracy in mapping (Fig. 2, Supplementary Fig. 3, Supplementary Table 7).

T2T-CHM13 reference improves the analysis of structural variants for long-read sequencing and short-read sequencing datasets

To understand the added value of the gapless T2T-CHM13 reference with the currently used hg38, SVs in known genes and pseudogenes, protein-coding genes and medically relevant genes and their lengths were compared for the LRS and SRS datasets, excluding the Y chromosome and ALT contigs (Fig. 3, Supplementary Table 7). For comparison between references, unique gene IDs from RefSeq NCBI annotations (version 110) using the main MANE transcript were used, which enables also comparison of gene paralogs. Using the T2T-CHM13 reference, the number of deletions increased by more than 20% and the number of insertions decreased by more than 20% using the LRS-ONT and LRS-PacBio datasets compared with hg38 (Fig. 3, Supplementary Fig. 1, Supplementary Table 2). Using SRS, approximately 10% more deletions and 80%

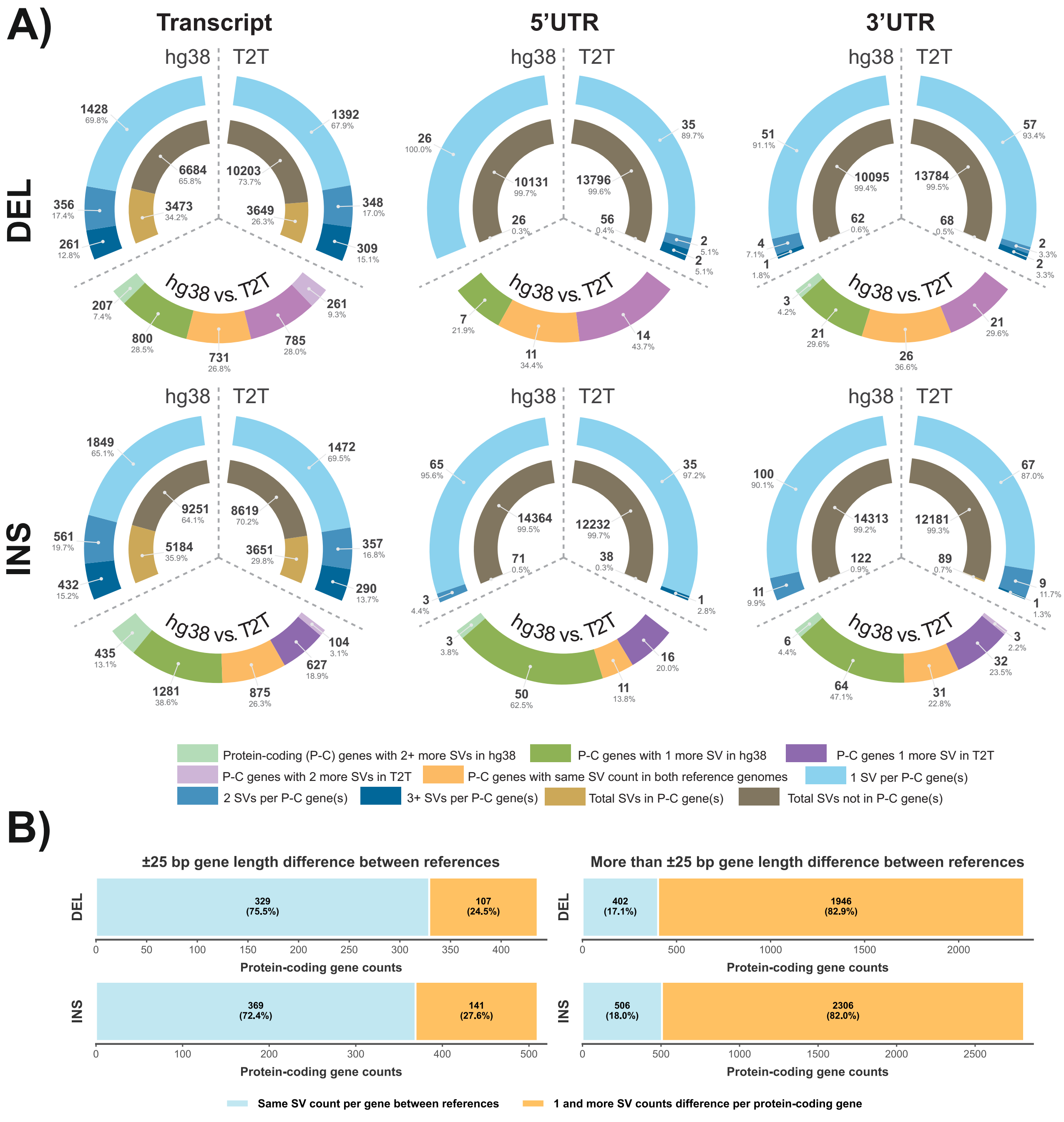

Fig. 4. Comparison of detected structural variants (SVs) located in A) protein-coding sequences (MANE transcripts) and 5'UTR and 3'UTR regions for both hg38 and T2T-CHM13 reference genomes and (B) genes with length differences of more and less than ± 25 bp between reference genomes hg38 and T2T-CHM13.

fewer insertions were detected using T2T-CHM13 than using hg38 (Fig. 3, Supplementary Fig. 1, Supplementary Table 2). In the unique regions of T2T-CHM13, numerous translocations, deletions and other SVs were detected in the telomere, centromere and subcentromere regions in all analysed datasets (Supplementary Tables 4–6, Supplementary Fig. 4).

Of the detected SVs, approximately 27%–33% were located in the protein-coding sequences, approximately 0.5% in each of the 5' UTR and 3' UTR regions and the remainder in the non-coding regions (Fig. 4, Supplementary Table 8). The T2T-CHM13 reference also refined the length of the genes. Comparing T2T-CHM13 and hg38 references for 54,553 genes and pseudogenes that overlap based on the gene ID (Fig. 3, Supplementary Table 7), 45.6% were the same length, 23.7% differed by 1–10 bp, 16.8% by 11–100 bp, 8.2% by 101–1,000 bp and 5.6% by more than 1,000 bp. Regarding protein-coding genes (medically relevant genes), 19.9% (14.3%) were the same length, 31.7% (31.0%) differed by 1–10 bp, 26.7% (30.2%) by 11–100 bp, 13.3% (15.8%) by 101–1,000 bp and 8.4% (8.8%) by more than 1,000 bp (Fig. 3). For genes of similar length (± 25 bp), minimal differences in SV counts were observed between the two references, whereas larger differences in length were associated with substantial differences in SV counts (Fig. 4, Supplementary Table 8). Among those with the largest differences in length were *GRK1* [32] and *LPA* [33] and many other genes, such as *SMN1&2* [34], *DUX4* and *HLA-DRB5* or *GBA* and its pseudogene *GBAP1*, were found to be disassembled, not correctly assembled or highly similar in hg38 (Supplementary Fig. 5, Supplementary Fig. 6, Supplementary Table 7). Furthermore, additional 167 protein-coding genes were annotated in T2T-CHM13 than in hg38 (Supplementary Table 7). To complement the added value of T2T-CHM13, we marked the genes found in the discrepant regions between hg19 and hg38 [35, 36] (Supplementary Table 7).

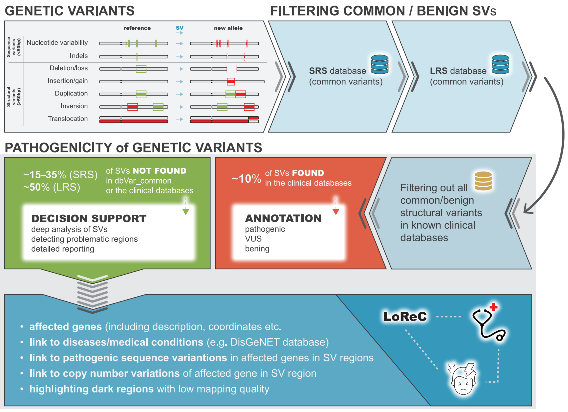

Fig. 5. Suggested workflow for annotation of structural variants (SVs) detected by long-read sequencing in diagnostic samples. First, SVs in the samples are filtered using a dbVar_common database or similar to filter out common and benign SVs. The SVs not found in the dbVar_common database are compared with the ClinVar SV database or similar. For SVs not found in the ClinVar database, the LoReC toolkit is used for the comparison and annotation of SVs in the samples based on the NCBI annotation file(s), including affected gene (gene description, coordinates) and link to the disease or medical condition based on the gene/variant-disease associations present in DisGeNET or similar.

Annotation of structural variants in clinical databases is challenging

To annotate the SVs detected by the tested technologies, we used a LoReC comparator toolkit to compare the detected SVs with the dbVar_common (NCBI dbVar Curated Common Structural Variants,

Table 1. Annotations of deletions detected in whole-genome datasets for the NA12878 and SKBR3 cell lines and P3 and S48 diagnostic samples by SRS and LRS technologies employing A) LoReC using dbVar_common and ClinVar and B) AnnotSV, both for the hg38 reference.

ALL		A) LoReC*							B) AnnotSV			
**Sample**	**Technology**	Total DEL	dbVar_common	ClinVar	Pathogenic/ Likely Pathogenic	VUS	Benign/ Likely Benign	Not annotated	Total DEL+BND	Benign	Pathogenic/ Likely Pathogenic	VUS
**NA12878**	SRS	5,120	3,332 (65%)	594 (12%)	29/11	62	461/19	1,734 (34%)	5,525	4,598 (83%)	32/6	889 (16%)
	LRS-PacBio	10,157	4,696 (46%)	1,085 (11%)	41/19	91	879/39	5,220 (51%)	10,157	6,030 (59%)	7/6	4,114 (41%)
	LRS-ONT	10,439	4,692 (45%)	1,090 (11%)	42/21	90	883/36	5,519 (53%)	10,439	5,893 (56%)	2/7	4,537 (43%)
	LRS-ICLR	9,412	4,018 (43%)	832 (9%)	42/18	75	655/25	5,199 (55%)	9,412	4,767 (51%)	2/4	4,639 (49%)
**SKBR3**	SRS	2,920	2,261 (78%)	549 (19%)	65/9	91	358/17	570 (20%)	2,890	2,373 (82%)	46/32	457 (16%)
	LRS-PacBio	9,097	4,168 (46%)	932 (10%)	62/15	98	710/33	4,693 (52%)	9,097	6,095 (67%)	30/10	2,962 (33%)
	LRS-ONT	10,983	4,343 (40%)	979 (9%)	64/18	98	748/32	6,402 (58%)	10,983	7,183 (65%)	29/15	3,756 (34%)
**P3**	SRS	5,018	3,222 (64%)	580 (12%)	28/8	72	437/22	1,741 (35%)	5,478	4,463 (81%)	36/4	975 (18%)
	LRS-ICLR	9,166	3,899 (43%)	756 (8%)	35/16	82	579/30	5,095 (56%)	9,166	4,486 (49%)	0/3	4,677 (51%)
**S48**	SRS	5,632	3,480 (62%)	652 (12%)	39/12	82	480/24	2,069 (37%)	6,199	5,035 (81%)	84/6	1,074 (17%)
	LRS-ICLR	9,279	3,970 (43%)	811 (9%)	49/13	89	613/29	5,102 (55%)	9,279	4,715 (51%)	5/6	4,553 (49%)

Legend: SRS, short-read sequencing by Illumina platform; LRS-PacBio, true long-read sequencing by Pacific Biosciences; LRS-ONT, true long-read sequencing by Oxford Nanopore Technologies; LRS-ICLR, synthetic long-read sequencing by Illumina - complete long-reads technology on Illumina platform; LRS-TELL-Seq, synthetic long-read sequencing by Universal Sequencing Technology on Illumina platform; LRS-10x, synthetic long-read sequencing by 10x Genomics on Illumina platform; OGM, optical genome mapping by Bionano Genomics; VUS, variant of unknown significance; DEL, deletion; BND, Breakends. *The main annotation with the lowest intersection factor (the highest overlap) that passed the selected filters between sample and database.

GnomAD and other sources, ID: nstd186) [28] and ClinVar (ID: nstd102) clinical datasets [37]. The dbVar_common database includes SVs that occur with a frequency of >1% in the population. As the current ClinVar database is based on hg38, annotations of SVs were primarily performed using this reference. Given that SV coordinates may vary from individual to individual, the following parameters need to be defined prior to each analysis: 1) the distance variance threshold (acceptable difference in bp between the SV of interest and the SV in the clinical database), 2) the intersection factor (overlap between the SV of interest and the nearest SV in the clinical database) and 3) the minimum size fraction (minimum proportion of the SV of interest and the nearest SV in the clinical database) (see the Materials and Methods for details). All SVs were also evaluated using the AnnotSV tool [38] (Table 1, Fig. 1).

Regarding deletions, 62%–78% of deletions detected by the SRS datasets overlapped with the deletions in dbVar_common and approximately 10% with those in ClinVar. When using AnnotSV, approximately 80% of the SVs in the SRS datasets were annotated as benign, approximately 1% (38–90) of deletions per sample were annotated as pathogenic/likely pathogenic and less than 18% were annotated as a VUS. Using true LRS, approximately 45% of deletions were detected in dbVar_common and 10% in ClinVar; for more than half the deletions, no annotation was available (Table 1). Using ClinVar annotations, the majority of SVs in this database span very large regions containing many genes; therefore, we used the size proportion parameter of 1% for LoReC annotations. Using this threshold, the LoReC annotations were consistent with the AnnotSV results: 49%–67% of SVs in the LRS datasets were annotated as benign, approximately 10 deletions (~0.1%) were annotated as pathogenic/likely pathogenic and approximately 4,500 deletions (30%–50%) as a VUS, depending on the sample and technology used (Table 1). Additionally, LoReC observed more pathogenic/likely pathogenic SVs in LRS datasets (Table 1). Regarding insertions, the dbVar_common/ClinVar databases contain only breakpoints and lack information on the insertion length for most insertions, making annotation impossible, which is also problematic for inversions and translocations. For information regarding annotations using the T2T-CHM13 reference genome, see Supplementary Table 9.

Annotation of the structural variants not presented in the clinical databases by LoReC

We suggest a workflow for diagnostic laboratories to annotate SVs from LRS/SRS. Briefly, after filtering out benign/common SVs present in the clinical databases (currently, mainly based on SRS, but in the future, LRS datasets will be added; e.g. dbVar), the remaining SVs are compared with the ClinVar clinical database or similar (Fig. 5). For SVs not presented in the dbVar_common/ClinVar databases, our LoReC toolkit enables the addition of custom annotations for regions/genes within the detected SVs. In particular, genes may be linked to the gene/VDAs provided in the annotation file (e.g. DisGeNET) or other custom annotations (Supplementary Table 7). In addition, the LoReC toolkit provides coverage for genes and regions for selected mapping quality, information about the SV localisation (e.g. protein-coding region, medical relevant genes), and enables the visualisation of regions of interest for expert evaluation.

Discussion

The importance of SVs in clinical diagnostics continues to expand due to advances in genomic technologies and wet lab protocols, the introduction of the gapless reference sequence T2T-CHM13 and the release of high-quality datasets. Despite the growing importance of SVs in human diseases, our knowledge of SVs in health and disease is limited, largely due to their structural complexity and variable length in individuals as well as the limitations inherent in available genomic technologies. Here, we comprehensively evaluated SVs from human whole-genome datasets obtained from SRS, all available LRS and OGM platforms across different samples, technologies and clinical databases using two human references (hg38 and T2T-CHM13). Moreover, our multiplatform approach enables annotation of detected SVs to support the implementation of SV diagnostics in clinical practice. Despite advances in clinical genetic diagnostics, approximately 50% of all suspected Mendelian diseases and many sporadic development syndromes and chronic diseases remain unresolved [39, 40]. The diagnostics of SVs is relevant not only in rare genetic diseases; SVs are also important contributors to chronic diseases, including cancer [5, 6, 41]. However, the technical limitations inherent in the available genomic technologies, as well as the structural complexity of SVs and their variable length in individuals, have led to an incomplete characterisation of SVs in the human genome in relation to health and disease compared with single nucleotide variations and small INDELs. To gain more insights into the performance of available genomic technologies, we compared our own and public whole-genome datasets from SRS, two LRS platforms (PacBio-LRS, ONT-LRS) requiring specific instruments, and three LRS approaches utilising synthetic long reads sequenced on conventional short-read NGSs. To achieve this, we developed the multiplatform LoReC toolkit, which compares the size and type of SVs, their overlap, coverage, coordinates, affected genes and disease associations from different SRS and LRS datasets in specific samples and/or across samples and databases, regardless of which of the many available algorithms was used to detect SVs [42, 43].

The LoReC toolkit was developed to provide comprehensive SV comparison and annotation between samples, technologies and databases for use in diagnostic laboratories, thereby extending the functionalities of SV comparison toolkits such as Jasmine [44] and SURVIVOR [45] (Supplementary Table 10). First, we were interested in the performance of SRS compared with third-generation technologies. On average, approximately 13,000 SVs/genome were detected by SRS and twice as many (~25,000 SVs/genome) by LRS. The lower performance of SRS in SV detection may arise from mapping short reads to multiple regions, discordant pairs and split reads, which limit SV detection by current variant callers [46, 47]. Notably, most of the SVs detected by SRS were also detected by LRS. Our findings are consistent with those of others showing that LRS can identify hidden disease-related SVs that are not detected by SRS [3, 36, 46]. Since 80% of the SVs detected by LRS/SRS were smaller than 0.5 kbp, OGM did not detect most of the SVs detected by LRS/SRS. The most common type of SVs were deletions and insertions, whereas half as many deletions (~5,000 vs ~10,000) and insertions (~6,000 vs more than 12,000) per sample were detected by SRS compared with LRS. Our data reveal that LRS-ONT and LRS-PacBio technologies have superior performance in detecting SVs to the SRS, the synthetic read LRS-ICLR, LRS-10x, LRS-TELL-Seq, and OGM. In addition to detecting more SVs through LRS, longer reads map more uniquely to the genome than SRS. Despite using the default MAPQ0 settings employed in the current SRS/LRS aligners and variant callers that allow the use of misplaced reads, effective coverage of most genes across genomes was achieved for all SRS/LRS technologies. To address this issue, current SRS pipelines often mask these problematic dark regions, which include repetitive elements and polymorphic regions, potentially leading to the loss of key information in these regions. In addition, PacBio provides a BED file for dark regions that occur in repetitive areas or areas with high GC content. When stricter MAPQ1 or MAPQ50 was applied, many regions across the genome were not covered well in the SRS datasets, including numerous protein-coding and medically relevant genes. Of the technologies analysed, the lowest probability of mismatches was observed for the data obtained by LRS-PacBio HiFi, the highest

for SRS. The LRS-ONT datasets demonstrated a high percentage of low-quality reads due to lower nucleotide accuracy when using the MAPQ0 setting. When using MAPQ1 or MAPQ50 to filter out low-quality reads, uniform coverage across the whole genome with the high-quality reads and very long reads generated was achieved by LRS-ONT. Notably, LRS-ONT has introduced adaptive sampling, a computational enrichment technique that adjusts the sequencing parameters in the regions of interest [48, 49], enabling deeper coverage in these regions [50].

Another key step for clinical genetics is the introduction of the gapless T2T-CHM13 human reference assembly, which uncovers 8% of the dark regions of the genome, adds nearly 200 million bases and predicts 99 novel protein-coding genes compared with reference hg38 [51]. Our comprehensive bioinformatic analysis of whole-genome datasets further supports the clinical utility of using the T2T-CHM13 sequence for medical diagnostics. When we compared medically relevant genes using coordinates based on NCBI RefSeq annotations on both references, approximately 85% of the genes changed size and more than half by more than 10 bp. Notably, approximately 9% of the medically relevant genes differed by >1,000 bp between hg38 and T2T-CHM13. Among the genes significantly differing between both references were those that were disassembled, not correctly assembled or highly similar in hg38, such as the challenging medically relevant genes *GRK1*, *LPA*, *SMN1/2*, *DUX4* and *HLA-DRB5* or the *GBA* gene and its pseudogene *GBAP1* [52].

For example, GRCh38 contains only six copies of the 5.5 kb KIV-2 repeat in *LPA*, even though human genomes carry ~5 to >50 copies [33], omits the complete macrosatellite region containing >20 D4Z4 repeats in *DUX4* [53], introduces gaps and misassemblies within the approximately 500-kbp segmental duplication, omits Alu-mediated deletions and does not represent the variable *SMN1/SMN2* copy numbers [54]. Furthermore, the novel T2T-CHM13 reference resulted in approximately 20% more deletions and 20% fewer insertions than hg38. It should be noted that some of the observed differences in SV counts reflect reference bias, as sequences that are minor alleles in hg38 but common in human populations and thus represented in T2T-CHM13. Given that many disease-causing SVs are located in repetitive, duplicated, inverted, or structurally complex regions that were missing. disassembled or misinterpreted in older references and cannot be resolved using SRS [55], clinical genetics can benefit from the use of T2T-CHM13 and LRS. For example, T2T-CHM13 and LRS were used to detect previously hidden variants in undiagnosed cases of rare diseases within the European Solve-RD consortium [56], repeated expansions in cerebellar ataxia [57] and inversions disrupting the *EHMT1* gene in Kleefstra syndrome [58]. Similarly, T2T-CHM13 and LRS show promise in diseases associated with copy number variability, such as those known in the *SMN1/2* genes in spinal muscular atrophy [59] and the D4Z4 repeats in the *DUX4* gene in facioscapulohumeral muscular dystrophy [60]. Even in SRS data, the use of T2T-CHM13 improves read mapping and enhances the detection of clinically relevant rare and deleterious variants [61]. Taken together, there is growing evidence of the benefits of LRS and T2T-CHM13 for clinical medicine.

Another challenging topic is the pathogenicity annotations of thousands of SVs detected by LRS/SRS. Unlike sequence variants, for whose interpretation exist guidelines [62, 63] and clinical databases such as dbVar_common/ClinVar based on large available SRS datasets, SV annotations are more difficult not only due to the insufficient number of LRS datasets in the databases but also the substantial variability in SV breakpoints between individuals and the complexity of rearrangements. Therefore, we introduced crucial measures for SV evaluations, such as the distance in bp between SVs, the intersection factor and the size proportion for SVs in datasets, references and databases. Since most deletions in the ClinVar database cover very large regions containing many genes, a size proportion of 1% was used for LoReC annotations. Both LoReC and AnnotSV [38] have annotated many SVs using their own algorithm, but there are still many SVs that are not present in the current dbVar_common and ClinVar databases or other databases. It should be noted that for the majority of SVs per genome, many of which were located in protein-coding sequences, no annotation was available in the current version of the ClinVar database. Regarding insertions and other SVs, the length of the insertion and sequence of the insert are missing in the dbVar_common/ClinVar databases, making their annotation impossible. Unlike deletions, which remove annotated regions and can be assessed by their absence, insertions introduce sequences that are not present in the reference. Moreover, insertions frequently occur within, or generate, tandem repeats and segmental duplications, regions inherently difficult to assess. Consequently, insertions frequently lack reliable population frequency estimates and functional or phenotypic evidence, making confident clinical classification challenging [64]. Addressing these challenges will require systematic characterisation of insertion sequences and their functional annotation, precise breakpoint resolution and the development of tools for functional impact prediction. For SVs not annotated by ClinVar, we applied the LoReC toolkit, and, with its help, the affected gene(s) can be linked with gene/variant disease associations using DisGeNET, human phenotype ontology, or a similar database. In the future, this approach will allow SV annotations to be matched to continuously updated clinical databases based on the LRS datasets and linked to the reference T2T-CHM13.

This study and other [11, 12, 46, 50, 65, 66, 67] further support the introduction of LRS into medical diagnostics. However, LRS will not replace SRS in the near future but will complement it, especially in cases with negative SRS results, as this method will remain the gold standard for routine diagnostics due to its cost, speed and established clinical and bioinformatic pipelines. Nevertheless, to introduce LRS into diagnostics, laboratories should collect cells for high-molecular-weight DNA isolation, as LRS cannot be performed on fragmented DNA obtained by standard isolation methods and increases computational and data storage capacity due to the large datasets it obtains. We highlight the introduction of distance variance, intersection, gene overlap and the closest SV in the clinical database for SV comparisons and annotations, which is currently the weakest point of SV integration into clinical diagnostics. Although this first comprehensive study on the performance of all available genomic technologies is focused on SVs in the human genome, SV events are widespread in other species, and our toolkit is also suitable for these datasets.

Conclusions

In this study, we introduced an innovative multiplatform approach for any SRS and third-generation dataset that advances SV comparisons across samples and databases as well as annotations of SVs based on comparisons with clinical databases. Although the gold standard SRS uncovers thousands of SVs that may be clinically relevant, we demonstrated that LRS is more effective at detecting SVs than SRS. Thus, LRS is expected to complement the SRS analysis in clinical diagnostics soon, especially in cases with negative SRS results. However, the implementation of LRS will require the introduction of isolation methods leading to high-molecular-weight DNA and the update of clinical databases to include LRS datasets and the T2T-CHM13 reference for the correct annotations of SVs.

Declarations

Ethical Approval

The patients provided written informed consent for the use of their biological materials for the purpose of this study, which was conducted in accordance with the Helsinki Declaration and approved by the local ethics committee (NW25-03-00391).

Consent for publication

Not applicable

Competing Interests

The authors have no competing interests to declare.

Funding

This study was supported from European Regional Development Fund-Project "Interdisciplinary Approaches for the Development and Application of New Materials in Medical Practice - New Omic Technologies" (CZ.02.01.01/00/23_021/0009224), the Internal Grant Agency of Palacký University (JG_2025_035), and the Ministry of Health of the Czech Republic (NW24-10-00395, MH CZ – DRO (FNOL, 00098892).

Author's Contributions

JS and EK designed the research; TN and JS developed the LoReC toolkit; JS, TN, PG and MB performed the bioinformatic analysis; JM and AP performed the laboratory experiments; MM, TP and FC collected the patient samples and clinical characteristics; JS and EK wrote the manuscript; and PG and AP critically revised the manuscript. All authors read and approved the final manuscript.

Availability of Source Code and Requirements

Project name: lorec-comparator
Project homepage: https://github.com/novosadt/lorec-comparator
Operating system: Windows, Linux, macOS, Solaris
Programming language: Java 8
Other requirements: None
License: GPL-3.0 license
RRID: SCR_027211

Project name: lorec-coverage
Project homepage: https://github.com/novosadt/lorec-coverage
Operating system: Windows, Linux, macOS, Solaris
Programming language: Java 8
Other requirements: None
License: GPL-3.0 license
RRID: RRID:SCR_027210

Data availability

All additional supporting data are available in the GigaScience repository, GigaDB [31]

References

1. Collins RL, Talkowski ME. Diversity and consequences of structural variation in the human genome. Nature Reviews Genetics 2025 Jan;https://www.nature.com/articles/s41576-024-00808-9.
2. Logsdon GA, Vollger MR, Eichler EE. Long-read human genome sequencing and its applications. Nature Reviews Genetics 2020 Oct;21(10):597–614. https://www.nature.com/articles/s41576-020-0236-x.
3. Mahmoud M, Huang Y, Garimella K, Audano PA, Wan W, Prasad N, et al. Utility of long-read sequencing for All of Us. Nature Communications 2024 Jan;15(1):837. https://www.nature.com/articles/s41467-024-44804-3.
4. Sudmant PH, Rausch T, Gardner EJ, Handsaker RE, Abyzov A, Huddleston J, et al. An integrated map of structural variation in 2,504 human genomes. Nature 2015 Oct;526(7571):75–81. https://www.nature.com/articles/nature15394.
5. DeBoever C, Tanigawa Y, Lindholm ME, McInnes G, Lavertu A, Ingelsson E, et al. Medical relevance of protein-truncating variants across 337,205 individuals in the UK Biobank study. Nature Communications 2018 Apr;9(1):1612. https://www.nature.com/articles/s41467-018-03910-9.
6. Collins RL, Brand H, Karczewski KJ, Zhao X, Alföldi J, Francioli LC, et al. A structural variation reference for medical and population genetics. Nature 2020 May;581(7809):444–451. https://www.nature.com/articles/s41586-020-2287-8.
7. Kosugi S, Momozawa Y, Liu X, Terao C, Kubo M, Kamatani Y. Comprehensive evaluation of structural variation detection algorithms for whole genome sequencing. Genome Biology 2019 Jun;20(1):117. https://doi.org/10.1186/s13059-019-1720-5.
8. Escaramís G, Docampo E, Rabionet R. A decade of structural variants: description, history and methods to detect structural variation. Briefings in Functional Genomics 2015 Sep;14(5):305–314. https://doi.org/10.1093/bfgp/elv014.
9. Ahsan MU, Liu Q, Perdomo JE, Fang L, Wang K. A survey of algorithms for the detection of genomic structural variants from long-read sequencing data. Nature Methods 2023 Aug;20(8):1143–1158. https://www.nature.com/articles/s41592-023-01932-w.
10. Amarasinghe SL, Su S, Dong X, Zappia L, Ritchie ME, Gouil Q. Opportunities and challenges in long-read sequencing data analysis. Genome Biology 2020 Feb;21(1):30. https://doi.org/10.1186/s13059-020-1935-5.
11. Mantere T, Kersten S, Hoischen A. Long-Read Sequencing Emerging in Medical Genetics. Frontiers in Genetics 2019 May;10. https://www.frontiersin.org/journals/genetics/articles/10.3389/fgene.2019.00426/full.
12. Sanford Kobayashi E, Batalov S, Wenger AM, Lambert C, Dhillon H, Hall RJ, et al. Approaches to long-read sequencing in a clinical setting to improve diagnostic rate. Scientific Reports 2022 Oct;12(1):16945. https://www.nature.com/articles/s41598-022-20113-x.
13. Mahmoud M, Gobet N, Cruz-Dávalos DI, Mounier N, Dessimoz C, Sedlazeck FJ. Structural variant calling: the long and the short of it. Genome Biology 2019 Nov;20(1):246. https://doi.org/10.1186/s13059-019-1828-7.
14. OLGEN, OLGEN Resources;. http://olgen.cz/en/resources, accessed 16 February 2026.
15. Zook JM, Catoe D, McDaniel J, Vang L, Spies N, Sidow A, et al. Extensive sequencing of seven human genomes to characterize benchmark reference materials. Scientific Data 2016 Jun;3(1):160025. https://www.nature.com/articles/sdata201625.
16. Aganezov S, Goodwin S, Sherman RM, Sedlazeck FJ, Arun G, Bhatia S, et al. Comprehensive analysis of structural variants in breast cancer genomes using single-molecule sequencing. Genome Research 2020 Sep;30(9):1258–1273. http://genome.cshlp.org/content/30/9/1258, company: Cold Spring Harbor Laboratory Press Distributor: Cold Spring Harbor Laboratory Press Institution: Cold Spring Harbor Laboratory Press Label: Cold Spring Harbor Laboratory Press.
17. van Dijk EL, Naquin D, Gorrichon K, Jaszczyszyn Y, Ouazahrou

R, Thermes C, et al. Genomics in the long-read sequencing era. Trends in Genetics 2023 Sep;39(9):649–671. https://www.sciencedirect.com/science/article/pii/S0168952523001191.
18. Genome in a Bottle Consortium, Genome in a Bottle (GIAB) data for NA12878 (HG001);. https://ftp.ncbi.nlm.nih.gov/ReferenceSamples/giab/data/NA12878/, accessed 16 February 2026.
19. Kriegova E, Fillerova R, Minarik J, Savara J, Manakova J, Petrackova A, et al. Whole-genome optical mapping of bone-marrow myeloma cells reveals association of extramedullary multiple myeloma with chromosome 1 abnormalities. Scientific Reports 2021 Jul;11(1):14671. https://www.nature.com/articles/s41598-021-93835-z, number: 1.
20. Li H. Minimap2: pairwise alignment for nucleotide sequences. Bioinformatics 2018 Sep;34(18):3094–3100. https://doi.org/10.1093/bioinformatics/bty191.
21. Smolka M, Paulin LF, Grochowski CM, Horner DW, Mahmoud M, Behera S, et al. Detection of mosaic and population-level structural variants with Sniffles2. Nature Biotechnology 2024 Jan;p. 1–10. https://www.nature.com/articles/s41587-023-02024-y.
22. Marks P, Garcia S, Barrio AM, Belhocine K, Bernate J, Bharadwaj R, et al. Resolving the full spectrum of human genome variation using Linked-Reads. Genome Research 2019 Apr;29(4):635–645.
23. Helal AA, Saad BT, Saad MT, Mosaad GS, Aboshanab KM. Benchmarking long-read aligners and SV callers for structural variation detection in Oxford nanopore sequencing data. Scientific Reports 2024 Mar;14(1):6160. https://www.nature.com/articles/s41598-024-56604-2.
24. Li H, Durbin R. Fast and accurate short read alignment with Burrows-Wheeler transform. Bioinformatics (Oxford, England) 2009 Jul;25(14):1754–1760.
25. Chen X, Schulz-Trieglaff O, Shaw R, Barnes B, Schlesinger F, Källberg M, et al. Manta: rapid detection of structural variants and indels for germline and cancer sequencing applications. Bioinformatics 2016 Apr;32(8):1220–1222. https://doi.org/10.1093/bioinformatics/btv710.
26. Piñero J, Ramírez-Anguita JM, Saüch-Pitarch J, Ronzano F, Centeno E, Sanz F, et al. The DisGeNET knowledge platform for disease genomics: 2019 update. Nucleic Acids Research 2020 Jan;48(D1):D845–D855. https://doi.org/10.1093/nar/gkz1021.
27. Belyeu JR, Chowdhury M, Brown J, Pedersen BS, Cormier MJ, Quinlan AR, et al. Samplot: a platform for structural variant visual validation and automated filtering. Genome Biology 2021 May;22(1):161. https://doi.org/10.1186/s13059-021-02380-5.
28. Lappalainen I, Lopez J, Skipper L, Hefferon T, Spalding JD, Garner J, et al. dbVar and DGVa: public archives for genomic structural variation. Nucleic Acids Research 2013 Jan;41(D1):D936–D941. https://doi.org/10.1093/nar/gks1213.
29. National Center for Biotechnology Information (NCBI), dbVar: Database of Genomic Structural Variation;. https://www.ncbi.nlm.nih.gov/dbvar/, accessed 16 February 2026.
30. Genovese G, Rockweiler NB, Gorman BR, Bigdeli TB, Pato MT, Pato CN, et al. BCFtools/liftover: an accurate and comprehensive tool to convert genetic variants across genome assemblies. Bioinformatics 2024 Feb;40(2):btae038. https://doi.org/10.1093/bioinformatics/btae038.
31. Savara J, Novosad T, Gajdos P, Petrackova A, Behalek M, Manakova J, et al., Supporting data for "Multiplatform comparisons and annotation of structural variants highlight the utility of the T2T reference genome in human diagnostics". GigaScience Database; 2026. https://doi.org/10.5524/102806.
32. Xie H, Li W, Hu Y, Yang C, Lu J, Guo Y, et al. De novo assembly of human genome at single-cell levels. Nucleic Acids Research 2022 Jul;50(13):7479–7492. https://doi.org/10.1093/nar/gkac586.
33. Chin CS, Behera S, Metcalf GA, Gibbs RA, Boerwinkle E, Sedlazeck FJ, A pan-genome approach to decipher variants in the highly complex tandem repeat of LPA. bioRxiv; 2022. https://www.biorxiv.org/content/10.1101/2022.06.08.495395v2, pages: 2022.06.08.495395 Section: New Results.
34. Chen X, Harting J, Farrow E, Thiffault I, Kasperaviciute D, Hoischen A, et al. Comprehensive SMN1 and SMN2 profiling for spinal muscular atrophy analysis using long-read PacBio HiFi sequencing. American Journal of Human Genetics 2023 Feb;110(2):240–250. https://www.ncbi.nlm.nih.gov/pmc/articles/PMC9943720/.
35. Yang X, Wang X, Zou Y, Zhang S, Xia M, Fu L, et al. Characterization of large-scale genomic differences in the first complete human genome. Genome Biology 2023 Jul;24(1):157. https://doi.org/10.1186/s13059-023-02995-w.
36. Mandelker D, Schmidt RJ, Ankala A, McDonald Gibson K, Bowser M, Sharma H, et al. Navigating highly homologous genes in a molecular diagnostic setting: a resource for clinical next-generation sequencing. Genetics in Medicine 2016 Dec;18(12):1282–1289. https://www.sciencedirect.com/science/article/pii/S1098360021014258.
37. Landrum MJ, Lee JM, Benson M, Brown GR, Chao C, Chitipiralla S, et al. ClinVar: improving access to variant interpretations and supporting evidence. Nucleic Acids Research 2018 Jan;46(D1):D1062–D1067. https://doi.org/10.1093/nar/gkx1153.
38. Geoffroy V, Herenger Y, Kress A, Stoetzel C, Piton A, Dollfus H, et al. AnnotSV: an integrated tool for structural variations annotation. Bioinformatics 2018 Oct;34(20):3572–3574. https://doi.org/10.1093/bioinformatics/bty304.
39. Wojcik MH, Reuter CM, Marwaha S, Mahmoud M, Duyzend MH, Barseghyan H, et al. Beyond the exome: What's next in diagnostic testing for Mendelian conditions. The American Journal of Human Genetics 2023 Aug;110(8):1229–1248. https://www.sciencedirect.com/science/article/pii/S0002929723002100.
40. Merker JD, Wenger AM, Sneddon T, Grove M, Zappala Z, Fresard L, et al. Long-read genome sequencing identifies causal structural variation in a Mendelian disease. Genetics in Medicine 2018 Jan;20(1):159–163. https://www.sciencedirect.com/science/article/pii/S1098360021019328.
41. van Belzen IAEM, Schönhuth A, Kemmeren P, Hehir-Kwa JY. Structural variant detection in cancer genomes: computational challenges and perspectives for precision oncology. npj Precision Oncology 2021 Mar;5(1):1–11. https://www.nature.com/articles/s41698-021-00155-6.
42. Duan DM, Cheng C, Huang YS, Chung Ak, Chen PX, Chen YA, et al. Comparisons of performances of structural variants detection algorithms in solitary or combination strategy. PLOS ONE 2025;20(2):e0314982. https://journals.plos.org/plosone/article?id=10.1371/journal.pone.0314982.
43. Liu Z, Roberts R, Mercer TR, Xu J, Sedlazeck FJ, Tong W. Towards accurate and reliable resolution of structural variants for clinical diagnosis. Genome Biology 2022 Mar;23(1):68. https://doi.org/10.1186/s13059-022-02636-8.
44. Kirsche M, Prabhu G, Sherman R, Ni B, Battle A, Aganezov S, et al. Jasmine and Iris: population-scale structural variant comparison and analysis. Nature Methods 2023 Mar;20(3):408–417. https://www.nature.com/articles/s41592-022-01753-3.
45. Jeffares DC, Jolly C, Hoti M, Speed D, Shaw L, Rallis C, et al. Transient structural variations have strong effects on quantitative traits and reproductive isolation in fission yeast. Nature Communications 2017 Jan;8(1):14061. https://www.na

ture.com/articles/ncomms14061.
46. Wagner J, Olson ND, Harris L, Khan Z, Farek J, Mahmoud M, et al. Benchmarking challenging small variants with linked and long reads. Cell Genomics 2022 May;2(5).
47. Zook JM, Hansen NF, Olson ND, Chapman L, Mullikin JC, Xiao C, et al. A robust benchmark for detection of germline large deletions and insertions. Nature Biotechnology 2020 Nov;38(11):1347–1355. https://www.nature.com/articles/s41587-020-0538-8.
48. Lu H, Giordano F, Ning Z. Oxford Nanopore MinION Sequencing and Genome Assembly. Genomics, Proteomics & Bioinformatics 2016 Oct;14(5):265–279. https://www.sciencedirect.com/science/article/pii/S1672022916301309.
49. Martin S, Heavens D, Lan Y, Horsfield S, Clark MD, Leggett RM. Nanopore adaptive sampling: a tool for enrichment of low abundance species in metagenomic samples. Genome Biology 2022 Jan;23(1):11. https://doi.org/10.1186/s13059-021-02582-x.
50. Oehler JB, Wright H, Stark Z, Mallett AJ, Schmitz U. The application of long-read sequencing in clinical settings. Human Genomics 2023 Aug;17(1):73. https://doi.org/10.1186/s40246-023-00522-3.
51. Rhie A, Nurk S, Cechova M, Hoyt SJ, Taylor DJ, Altemose N, et al. The complete sequence of a human Y chromosome. Nature 2023 Sep;621(7978):344–354. https://www.nature.com/articles/s41586-023-06457-y.
52. Nurk S, Koren S, Rhie A, Rautiainen M, Bzikadze AV, Mikheenko A, et al. The complete sequence of a human genome. Science 2022 Apr;376(6588):44–53. https://www.science.org/doi/10.1126/science.abj6987.
53. Grobecker P, Berri S, Peden JF, Chow KJ, Fielding C, Armogida I, et al. A dedicated caller for DUX4 rearrangements from whole-genome sequencing data. BMC Medical Genomics 2025 Jan;18(1):24. https://doi.org/10.1186/s12920-024-02069-1.
54. Logsdon GA, Ebert P, Audano PA, Loftus M, Porubsky D, Ebler J, et al. Complex genetic variation in nearly complete human genomes. Nature 2025 Aug;644(8076):430–441. https://www.nature.com/articles/s41586-025-09140-6.
55. Gobbo GFD, Boycott KM. The additional diagnostic yield of long-read sequencing in undiagnosed rare diseases. Genome Research 2025 Apr;35(4):559–571. http://genome.cshlp.org/content/35/4/559, company: Cold Spring Harbor Laboratory Press Distributor: Cold Spring Harbor Laboratory Press Institution: Cold Spring Harbor Laboratory Press Label: Cold Spring Harbor Laboratory Press.
56. Steyaert W, Sagath L, Demidov G, Yépez VA, Esteve-Codina A, Gagneur J, et al. Unraveling undiagnosed rare disease cases by HiFi long-read genome sequencing. Genome Research 2025 Apr;35(4):755–768. http://genome.cshlp.org/content/35/4/755, company: Cold Spring Harbor Laboratory Press Distributor: Cold Spring Harbor Laboratory Press Institution: Cold Spring Harbor Laboratory Press Label: Cold Spring Harbor Laboratory Press.
57. Rafehi H, Fearnley LG, Read J, Snell P, Davies KC, Scott L, et al. A prospective trial comparing programmable targeted long-read sequencing and short-read genome sequencing for genetic diagnosis of cerebellar ataxia. Genome Research 2025 Apr;35(4):769–785. http://genome.cshlp.org/content/35/4/769, company: Cold Spring Harbor Laboratory Press Distributor: Cold Spring Harbor Laboratory Press Institution: Cold Spring Harbor Laboratory Press Label: Cold Spring Harbor Laboratory Press.
58. Saether KB, Eisfeldt J, Bengtsson JD, Lun MY, Grochowski CM, Mahmoud M, et al. Leveraging the T2T assembly to resolve rare and pathogenic inversions in reference genome gaps. Genome Research 2024 Nov;34(11):1785–1797. http://genome.cshlp.org/content/34/11/1785, company: Cold Spring Harbor Laboratory Press Distributor: Cold Spring Harbor Laboratory Press Institution: Cold Spring Harbor Laboratory Press Label: Cold Spring Harbor Laboratory Press.
59. Zwartkruis MM, Elferink MG, Gommers D, Signoria I, Blasco-Pérez L, Costa-Roger M, et al. Long-read sequencing identifies copy-specific markers of SMN gene conversion in spinal muscular atrophy. Genome Medicine 2025 Mar;17(1):26. https://doi.org/10.1186/s13073-025-01448-2.
60. Huang M, Zhang Q, Jiao J, Shi J, Xu Y, Zhang C, et al. Comprehensive genetic analysis of facioscapulohumeral muscular dystrophy by Nanopore long-read whole-genome sequencing. Journal of Translational Medicine 2024 May;22(1):451. https://doi.org/10.1186/s12967-024-05259-8.
61. Schmitz D, Ameur A, Johansson A. T2T-CHM13 improves read mapping and detection of clinically relevant genetic variation in the Swedish population. Genome Research 2025 Nov;35(11):2377–2388. http://genome.cshlp.org/content/35/11/2377, company: Cold Spring Harbor Laboratory Press Distributor: Cold Spring Harbor Laboratory Press Institution: Cold Spring Harbor Laboratory Press Label: Cold Spring Harbor Laboratory Press.
62. Richards S, Aziz N, Bale S, Bick D, Das S, Gastier-Foster J, et al. Standards and guidelines for the interpretation of sequence variants: a joint consensus recommendation of the American College of Medical Genetics and Genomics and the Association for Molecular Pathology. Genetics in Medicine 2015 May;17(5):405–424. https://www.gimjournal.org/article/S1098-3600%2821%2903031-8/fulltext.
63. Miller DT, Lee K, Abul-Husn NS, Amendola LM, Brothers K, Chung WK, et al. ACMG SF v3.2 list for reporting of secondary findings in clinical exome and genome sequencing: A policy statement of the American College of Medical Genetics and Genomics (ACMG). Genetics in Medicine 2023 Aug;25(8). https://www.gimjournal.org/article/S1098-3600(23)00879-1/fulltext.
64. Nicholas TJ, Cormier MJ, Quinlan AR. Annotation of structural variants with reported allele frequencies and related metrics from multiple datasets using SVAFotate. BMC Bioinformatics 2022 Nov;23(1):490. https://doi.org/10.1186/s12859-022-05008-y.
65. Olivucci G, Iovino E, Innella G, Turchetti D, Pippucci T, Magini P. Long read sequencing on its way to the routine diagnostics of genetic diseases. Frontiers in Genetics 2024 Mar;15. https://www.frontiersin.org/journals/genetics/articles/10.3389/fgene.2024.1374860/full.
66. Negi S, Stenton SL, Berger SI, Canigiula P, McNulty B, Violich I, et al. Advancing long-read nanopore genome assembly and accurate variant calling for rare disease detection. The American Journal of Human Genetics 2025 Feb;112(2):428–449. https://www.sciencedirect.com/science/article/pii/S0002929725000023.
67. Savara J, Novosád T, Gajdoš P, Kriegová E. Comparison of structural variants detected by optical mapping with long-read next-generation sequencing. Bioinformatics 2021 Oct;37(20):3398–3404. https://doi.org/10.1093/bioinformatics/btab359.

Supplementary Fig 1

Click here to access/download
Supplementary Material
Supplementary Fig 1.pdf

Supplementary Fig 2

Click here to access/download
Supplementary Material
Supplementary Fig 2.pdf

Supplementary Fig 3

Click here to access/download
Supplementary Material
Supplementary Fig 3.pdf

Click here to access/download
Supplementary Material
Supplementary Fig 4.pdf

Supplementary Fig 5

Click here to access/download
Supplementary Material
Supplementary Fig 5.pdf

Supplementary Fig 6

Click here to access/download
Supplementary Material
Supplementary Fig 6.pdf

Supplementary Table 1

Click here to access/download
Supplementary Material
Supplementary Table 1.pdf

Supplementary Table 2

Click here to access/download
Supplementary Material
Supplementary Table 2.pdf

Supplementary Table 3

Click here to access/download
Supplementary Material
Supplementary Table 3.pdf

Supplementary Table 4

Click here to access/download
Supplementary Material
Supplementary Table 4.xlsx

Supplementary Table 5

Click here to access/download
Supplementary Material
Supplementary Table 5.xlsx

Supplementary Table 6

Click here to access/download
Supplementary Material
Supplementary Table 6.xlsx

Supplementary Table 7

Click here to access/download
Supplementary Material
Supplementary Table 7.xlsx

Supplementary Table 8

Click here to access/download
Supplementary Material
Supplementary Table 8.xlsx

Supplementary Table 9

Click here to access/download
Supplementary Material
Supplementary Table 9.pdf

Supplementary Table 10

Click here to access/download
Supplementary Material
Supplementary Table 10.pdf

Graphical Abstract

Click here to access/download;Graphical Abstract;Graphical Abstract.pdf

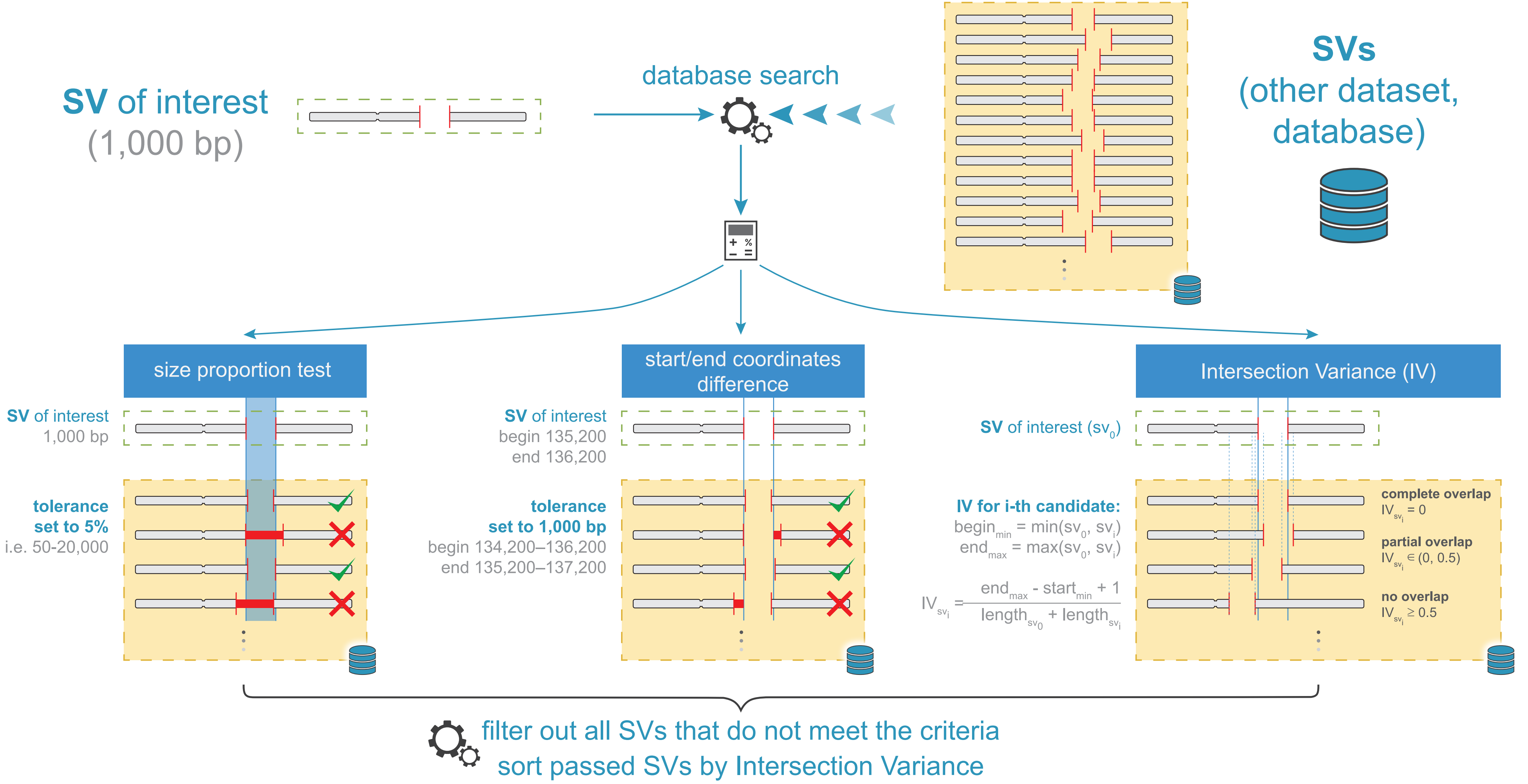

Supplement: giag027_GIGA-D-25-00250_Revision_1 [file giag027_giga-d-25-00250_revision_1.pdf]
