# Supplementary material for: Multiplatform comparisons and annotation of structural variants highlight the utility of the T2T reference genome in human diagnostics
Source: Gigascience. 2026 Mar 9;15:giag027. doi: 10.1093/gigascience/giag027 (PMC13137335; doi:10.1093/gigascience/giag027)
Supplement: giag027_Supplemental_Files [file Supplementary Fig 5.pdf › Supplementary Fig 5.pdf]

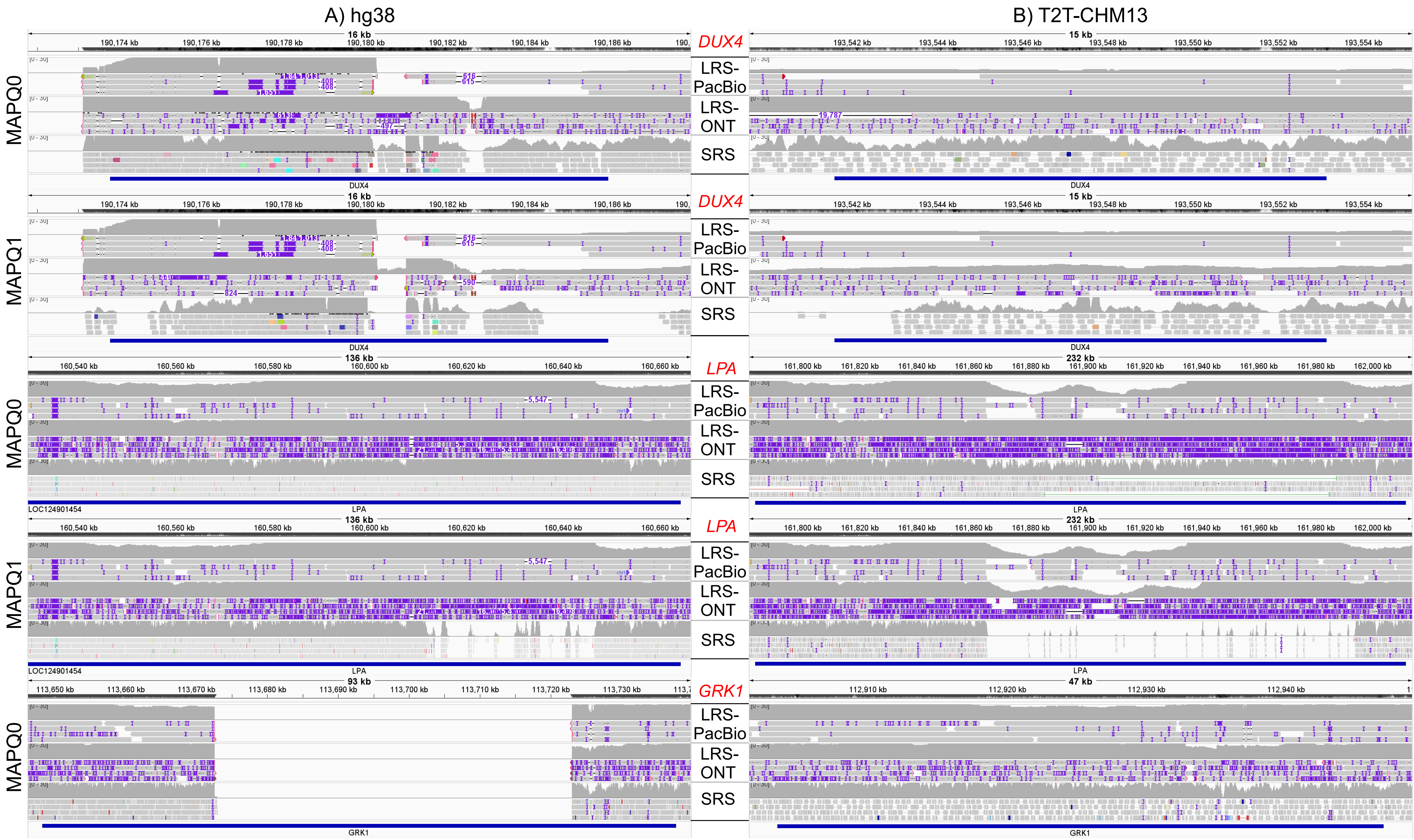

Supplementary Fig. 5. Coverage profiles for *DUX4*, *LPA* and *GRK1* genes detected by short-read and long-read technologies using hg38 and T2T-CHM13 references and different mapping quality thresholds (MAPQ0 and MAPQ1). This figure was generated using Integrative Genomics Viewer (IGV) with corresponding gene coordinates for *DUX4*, *LPA* and *GRK1* for both reference genomes.

Legend: SRS, short-read sequencing by Illumina platform; LRS-PacBio, true long-read sequencing by Pacific Biosciences; LRS-ONT, true long read sequencing
