# Supplementary material for: Multiplatform comparisons and annotation of structural variants highlight the utility of the T2T reference genome in human diagnostics
Source: Gigascience. 2026 Mar 9;15:giag027. doi: 10.1093/gigascience/giag027 (PMC13137335; doi:10.1093/gigascience/giag027)
Supplement: giag027_Authors_Response_To_Reviewer_Comments_original_submission [file giag027_authors_response_to_reviewer_comments_original_submission.pdf]

Author's Response To Reviewer Comments

AUTHORS' RESPONSE TO THE REVIEWERS' COMMENTS
First, we would like to thank the reviewers for thoroughly reading and critiquing our manuscript. We greatly appreciate all comments and suggestions.

Reviewers' comments:
Reviewer#1:
The author presented LoReC that compares and annotates SVs detected from different sequencing technologies, highlighting the utility of T2T reference genome in diagnostics. The usage of T2T reference genome in clinical settings is an interesting and important study, especially the newly added sequence compared to hg38. This work aims to improve SV annotation and clinical interpretation, but it lacks enough evidence or analysis to support the statement where T2T improve diagnostics of patents. Here are some major comments and suggestions:

Q1. In "True long-read sequencing technologies have …", why the concordance variation of LRS-ONT and LRS-PacBio is high (ranges from 80%~95%)?
A1. We thank the reviewer for this comment. The variability in concordance of LRS-ONT and LRS-PacBio was linked to the different PacBio sequencing modes (HiFi vs continuous long reads (CLR)) used for different samples. Using HiFi PacBio mode, higher concordance was observed than in CLR (~95% vs 80%), we added this information to the Results part (page 3, Annotation of structural variants by the LongReadChecker toolkit).

Q2. In "Most structural variants detected by short-read …", what drives the large discordance between LRS and SRS? Are there any examples suggest the advantage of T2T reference and LRS, especially for clinical diagnose?
A2. We thank the reviewer for this valuable comment. There is growing evidence that the discordance between SRS and LRS is linked to the mapping of short reads to multiple regions, as we showed by the MAPQ evaluation, discordant pairs and split reads by current variant callers in SRS data. We now added this information the Discussion (page 8, Discussion).
Given that many disease-causing SVs are located in repetitive, duplicated, inverted, or structurally complex regions that were missing or misinterpreted in older references and cannot be resolved using SRS, clinical genetics can benefit from the use of T2T-CHM13 and LRS. We also mention, that the usage of T2T-CHM13 even improves read mapping and enhances detection of clinically relevant rare and deleterious variants in SRS. We added now to the MS also some examples of advantage of T2T reference and LRS for clinical diagnose (page 9, Discussion).

Q3. It is important to know which part of the gene is shorter or longer when compared T2T to hg38. For example, in 5'UTR, even 1-10 bp difference might result in different consequences. How much protein-coding gene SVs are called from each reference? How does the gene length variation affect SV calling?
A3. Based on the reviewer recommendation, we performed deeper analyses on SVs and their occurrence in protein coding sequences, 5´ UTR and 3´ UTR regions in both reference sequences (new Figure 3 and new Supplementary Table 8). We also compared genes with similar lengths (± 25 bp) and found minimal differences in SV counts between the two references, whereas larger differences in length were associated with substantial differences in SV counts (page 6; new Figure 4 and Supplementary Table 9).
In order to preserve the maximum number of images/tables, we have moved the original Table 1 to the Supplementary files.

Q4. SV count difference on T2T and hg38 is just the reference bias. For example, the excess insertion count on hg38 is because most them are minor alleles on hg38 but major in the population.
A4. We agree with the reviewer opinion. We added this comment to the Discussion part (page 9, Discussion).

Q5. The author mentioned genes such as LPA, SMN1/2 and DUX4. Those are all copy number variable genes and known to be diverse in the population. How is SV detected in those regions and what's the

difference between references? It is also worthwhile to examine the SV discovery and mappability of genome assembly in these regions such as SMN1/2.
A5. We thank the reviewer for the comment. T2T-CHM13 significantly improves the mapping of genes with variable copy numbers and we have added examples of genes (LPA, DUX4, and SMN1/2) related to discrepancies between the two reference sequences to the Discussion (Page 9, Discussion).

Q6. In table2, why the number of Pathogenic/Likely Pathogenic is different so much between LoReC and AnnotSV. For example, LeReC annotates 1/4 (LRS-Pacbio) compared to 30/10 from AnnotSV.
A6. Thank you very much for this valuable comment. As the original manuscript compared mainly samples across the technologies, we used for annotation using ClinVar 5% size proportion. However, the majority of SVs in clinical database ClinVar span very large regions containing many genes and therefore lower the size proportion threshold of 1% may be better for comparisons. Using 1% threshold, LoReC annotations were in concordance with AnnotSV results, as stated now in the updated Table 1 and Results section (page 7-8, Results).

Reviewer #2:
Savara et al presents a toolkit LoReC for annotation and comparison of SVs. The insights into T2T-CHM13 analysis makes this manuscript timely and valuable. I was able to find the software and run it locally on an small example and the github provides sufficient documentation for usage.

Some key achievements:

- The experimental design and the results makes this manuscript very strong. The authors have gone to the extent of making sure not only synthetic data is used for analysis, which is the core strength of this manuscript.
- The authors' insights in use of T2T reference genome and discussion around critical bottleneck around clinical genomics also makes it very timely.
- The developed methods and results all show good results which is positive.

Areas to improve:
Q1: - Please compare against tools like Jasmine and SURVIOR to make this study comprehensive. I believe without comparing against existing methods, it is unclear how LoReC is novel and the advantages of using it.
A1. Based on your recommendation, we have added a new Supplementary Table 10 comparing LoReC with existing merging tools such as Jasmine and SURVIVOR. Generally speaking, LoReC provides a holistic framework for SV analysis that extends and complements the functionalities of Jasmine and SURVIVOR with outputs needed for diagnostic laboratories, as stated in the Discussion section (page 10). We added to the Supplementary Table S10 the comparison of available toolkits.

Q2:- I think the authors spend too much time comparing SRS vs LRS. In the genomics community, it is now established that LRS is better for SV detection. I would request the authors to focus on the novelty in the results rather than focusing comparisons between platforms.
A2. We agree with the reviewer that LRS should be the future of genetic diagnostics. However, one may expect that LRS will not replace SRS in the near future, but will complement it, especially in cases with negative SRS results. SRS will remain the gold standard for routine diagnostics due to its cost, speed, and established clinical pipelines, the requirement for high molecular weight DNA, the complexity of bioinformatic analysis, and the problems with annotating thousands of SVs, together with its limited availability in clinical laboratories and ethical issues, limit its routine use in the near future. We mention it in the Discussion (page 9, Discussion).

Q3:- The discussion section lacks comprehensive details on the current bottlenecks on annotation. It would be great if the authors could focus on discussing the annotation limitations, specially around the limitations of annotating insertions.
A3. We thank the reviewer for this valuable comment. Annotation of insertions is challenging. Unlike deletions, which remove annotated regions and can be assessed by absence, insertions introduce sequence not present in the reference. Moreover, the insertions frequently occur within or generate tandem repeats and segmental duplications, regions inherently difficult to assess. Consequently, insertions frequently lack

reliable population frequency estimates and functional or phenotypic evidence, making confident clinical classification challenging, as we state now in the Discussion (Page 9, Discussion). We also mention how to address these challenges in the future (Page 9, Discussion).

Cookie Preference Center

We use cookies which are necessary to make our site work. We may also use additional cookies to analyze, improve and personalize our content and your digital experience. For more information, see our Cookie Policy.

You may choose not to allow some types of cookies. However, blocking some types may impact your experience of our site and the services we are able to offer. See the different category headings below to find out more or change your settings.

Allow all

Manage Consent Preferences

Strictly Necessary Cookies

Always active

These cookies are necessary for the website to function and cannot be switched off in our systems. They are usually only set in response to actions made by you which amount to a request for services, such as setting your privacy preferences, logging in or filling in forms. You can set your browser to block or alert you about these cookies, but some parts of the site will not then work.

Functional Cookies

These cookies enable the website to provide enhanced functionality and personalisation. They may be set by us or by third party providers whose services we have added to our pages. If you do not allow these cookies then some or all of these services may not function properly.

Performance Cookies

These cookies allow us to count visits and traffic sources so we can measure and improve the performance of our site. They help us to know which pages are the most and least popular and see how visitors move around the site.

Targeting Cookies

These cookies may be set through our site by our advertising partners. They may be used by those companies to build a profile of your interests and show you relevant adverts on other sites. If you do not allow these cookies, you will experience less targeted advertising.

Cookie List

Clear

Apply Cancel

Consent Leg.Interest

Confirm my choices

Power
