# Supplementary material for: Multiplatform comparisons and annotation of structural variants highlight the utility of the T2T reference genome in human diagnostics
Source: Gigascience. 2026 Mar 9;15:giag027. doi: 10.1093/gigascience/giag027 (PMC13137335; doi:10.1093/gigascience/giag027)
Supplement: giag027_Reviewer_2_Report_original_submission — Reviewer 2 -- 9/26/2025 [file giag027_reviewer_2_report_original_submission.pdf]

Reviewer Report

Title: Multiplatform comparisons and annotation of structural variants highlight the utility of the T2T reference genome in human diagnostics

Version: Original Submission **Date:** 9/26/2025

Reviewer name: Reviewer 2

Reviewer Comments to Author:

Savara et al presents a toolkit LoReC for annotation and comparison of SVs. The insights into T2T-CHM13 analysis makes this manuscript timely and valuable. I was able to find the software and run it locally on an small example and the github provides sufficient documentation for usage.
Some key achievements:
- The experimental design and the results makes this manuscript very strong. The authors have gone to the extent of making sure not only synthetic data is used for analysis, which is the core strength of this manuscript.
- The authors' insights in use of T2T reference genome and discussion around critical bottleneck around clinical genomics also makes it very timely.
- The developed methods and results all show good results which is positive.

Areas to improve:
- Please compare against tools like Jasmine and SURVIOR to make this study comprehensive. I believe without comparing against existing methods, it is unclear how LoReC is novel and the advantages of using it.
- I think the authors spend too much time comparing SRS vs LRS. In the genomics community, it is now established that LRS is better for SV detection. I would request the authors to focus on the novelty in the results rather than focusing comparisons between platforms.
- The discussion section lacks comprehensive details on the current bottlenecks on annotation. It would be great if the authors could focus on discussing the annotation limitations, specially around the limitations of annotating insertions.

Overall, the authors did a good job in developing the toolkit and writing the method.

Methods

Are the methods appropriate to the aims of the study, are they well described, and are necessary controls included? Choose an item.

Conclusions

Are the conclusions adequately supported by the data shown? Choose an item.

Reporting Standards

Does the manuscript adhere to the journal's guidelines on minimum standards of reporting? Choose an item.

Choose an item.

Statistics

Are you able to assess all statistics in the manuscript, including the appropriateness of statistical tests used? Choose an item.

Quality of Written English

Please indicate the quality of language in the manuscript: Choose an item.

Declaration of Competing Interests

Please complete a declaration of competing interests, considering the following questions:

- Have you in the past five years received reimbursements, fees, funding, or salary from an organisation that may in any way gain or lose financially from the publication of this manuscript, either now or in the future?
- Do you hold any stocks or shares in an organisation that may in any way gain or lose financially from the publication of this manuscript, either now or in the future?
- Do you hold or are you currently applying for any patents relating to the content of the manuscript?
- Have you received reimbursements, fees, funding, or salary from an organization that holds or has applied for patents relating to the content of the manuscript?
- Do you have any other financial competing interests?
- Do you have any non-financial competing interests in relation to this paper?

If you can answer no to all of the above, write 'I declare that I have no competing interests' below. If your reply is yes to any, please give details below.

- No - No - No - No - No - No

I agree to the open peer review policy of the journal. I understand that my name will be included on my report to the authors and, if the manuscript is accepted for publication, my named report including any attachments I upload will be posted on the website along with the authors' responses. I agree for my report to be made available under an Open Access Creative Commons CC-BY license (http://creativecommons.org/licenses/by/4.0/). I understand that any comments which I do not wish to be included in my named report can be included as confidential comments to the editors, which will not be published.

Choose an item.

To further support our reviewers, we have joined with Publons, where you can gain additional credit to further highlight your hard work (see: https://publons.com/journal/530/gigascience). On publication of this paper, your review will be automatically added to Publons, you can then choose whether or not to claim your Publons credit. I understand this statement.

Yes Choose an item.
